# Supplementary material for: Effect of Physical Inactivity on the Oxidation of Saturated and Monounsaturated Dietary Fatty Acids: Results of a Randomized Trial
Source: PLoS Clin Trials. 2006 Sep 29;1(5):e27. doi: 10.1371/journal.pctr.0010027 (PMC1584255; doi:10.1371/journal.pctr.0010027)
Supplement: Trial Protocol Part A [file pctr.0010027.sd002.doc]

# TABLE OF CONTENTS

**Part A**

1 BACKGROUND AND objectives [29](#__RefHeading___Toc507931012)

1.1 Adaptation of humans to long term simulated weightlessness : [30](#__RefHeading___Toc507931013)

1.2 Evaluation of 2 countermeasures : [32](#__RefHeading___Toc507931014)

1.2.1 Muscular exercise [32](#__RefHeading___Toc507931015)

1.2.2 Biphosphonates [32](#__RefHeading___Toc507931016)

1.2.3 Harmonisation of physiological and medical measurements to evaluate the effects of countermeasures. [33](#__RefHeading___Toc507931017)

1.3 Medes background [33](#__RefHeading___Toc507931018)

2 STUDY GENERAL DESCRIPTION [34](#__RefHeading___Toc507931019)

2.1 Operational scenario [34](#__RefHeading___Toc507931020)

2.2 Integrated Test Regimen (ITR). Adaptation of ITR program to the bed rest study [34](#__RefHeading___Toc507931021)

2.2.1 Daily physical examination, anthropometry and metabolic assessment [35](#__RefHeading___Toc507931022)

2.2.2 Laboratory assessment [36](#__RefHeading___Toc507931023)

2.2.3 Physical fitness assessment [36](#__RefHeading___Toc507931024)

2.2.4 Operational tilt test [41](#__RefHeading___Toc507931025)

2.2.5 Cardiovascular tests [42](#__RefHeading___Toc507931026)

2.2.6 Bone tests [43](#__RefHeading___Toc507931027)

2.2.7 Functional neurological assessment [43](#__RefHeading___Toc507931028)

2.3 Selection and follow-up of volunteers [44](#__RefHeading___Toc507931029)

2.3.1 Subject's recruiting and medical selection [44](#__RefHeading___Toc507931030)

2.3.2 Psychological Preparation [48](#__RefHeading___Toc507931031)

2.3.3 Medical Follow-up during the Experiment [48](#__RefHeading___Toc507931032)

2.3.4 Physiotherapy during the experiment [50](#__RefHeading___Toc507931033)

2.3.5 Clinical follow-up after experiment completion [51](#__RefHeading___Toc507931034)

2.3.6 Psychological surveillance during the experiment [51](#__RefHeading___Toc507931035)

2.3.7 Psychological follow-up after the experiment [52](#__RefHeading___Toc507931036)

2.4 MEDES team [52](#__RefHeading___Toc507931037)

2.5 Housing conditions for volunteers [53](#__RefHeading___Toc507931038)

2.5.1 MEDES Clinical Research Facilty (Space Clinic) [53](#__RefHeading___Toc507931039)

2.5.2 Diet monitoring [55](#__RefHeading___Toc507931040)

2.5.3 Video monitoring [55](#__RefHeading___Toc507931041)

2.5.4 Light conditions [55](#__RefHeading___Toc507931042)

2.5.5 Transportations [55](#__RefHeading___Toc507931043)

2.5.6 Leisure time [55](#__RefHeading___Toc507931044)

2.5.7 Visits to the volunteers [56](#__RefHeading___Toc507931045)

2.6 Regulatory aspects [56](#__RefHeading___Toc507931046)

2.6.1 Agreements [56](#__RefHeading___Toc507931047)

2.6.2 Volunteer compensation [56](#__RefHeading___Toc507931048)

2.6.3 PI certification [56](#__RefHeading___Toc507931049)

3 HARDWARE [57](#__RefHeading___Toc507931050)

4 biological samples [58](#__RefHeading___Toc507931051)

4.1 Blood samples [58](#__RefHeading___Toc507931052)

4.2 Urine samples [58](#__RefHeading___Toc507931053)

4.3 Saliva samples [58](#__RefHeading___Toc507931054)

4.4 Stools samples [58](#__RefHeading___Toc507931055)

4.5 Storage and transportation [58](#__RefHeading___Toc507931056)

5 risks & hazards [62](#__RefHeading___Toc507931057)

5.1 Identified risks and lack of comfort associated with the situation of anti-orthostatic bed rest [62](#__RefHeading___Toc507931058)

5.2 Risks and lack of comfort due to the drugs taken by the subject during the experimentation [62](#__RefHeading___Toc507931059)

5.3 Risks and lack of comfort due to electrodes [63](#__RefHeading___Toc507931060)

5.4 Risks and lack of comfort due to blood samples [63](#__RefHeading___Toc507931061)

5.5 Risks due Radiations [63](#__RefHeading___Toc507931062)

# LIST OF ACRONYMS

| **ANS** | Autonomous Nervous System |
| --- | --- |
| **BDC** | Baseline Data Collection |
| **BW** | Body Weight |
| **CM** | Countermeasures |
| **CNES** | Centre National d'Etudes Spatiales |
| **Co-I** | Co-Investigator |
| **Inv** | Investigator |
| **ECG** | Electrocardiogram |
| **EE** | Energy Expenditure |
| **EI** | Energy Intake |
| **ESA** | European Space Agency |
| **Hb** | Hemoglobin |
| **HDBR** | Head-Down Bed-Rest |
| **HDT** | Head-Down Tilt |
| **HR** | Heart Rate |
| **HRV** | Heart Rate Variability |
| **Ht** | Hematocrit |
| **HUT** | Head-up Tilt |
| **Hz** | Hertz |
| **ISS** | International Space Station |
| **LBNP** | Lower Body Negative Pressure |
| **MAP** | Mean Arterial Pressure |
| **MEDES** | Institut de Médecine et de Physiologie Spatiales |
| **NASDA** | National Space Development Agency of Japan |
| **PI** | Principal Investigator |
| **PNS** | Para-Sympathetic system |
| **PV** | Plasma Volume |
| **SAP** | Systolic Arterial Pressure |
| **SBR** | Spontaneous Baroreflex Response |
| **SNS** | Sympathetic Nervous System |
| **STAND** | Stand Test |
| **TBW** | Total Body Water |

# INVESTIGATORS LIST

**Dr. MIZUNO Koh (PI : Ohshima)**

Address:

National Space Development Agency of Japan (NASDA)

Medical Research and Operations Office, Tsukuba Space Center (TKSC), 2-1-1 Sengen, Tsukuba-shi

305-8505 IBARAKI - Japon

Phone: +81 (298) 54.3952 / Fax: +81 (298) 50.2232

Mobile:

E-mail: mizuno.koh@nasda.go.jp

Function: **Co-I**

Protocol code: OHS-BS; OHS-BS add; OHS-ACTI; OHS-IMP; OHS-RADIO; OHS-URS; OHS-URS Ca; OHS-STOOLS Ca; OHS HEIGHT; OHS-LBS; ITR-DEXA; ITR-CYBEX; ITR-NMRI; OHS-SLEEP

**Dr. OHSHIMA Hiroshi (PI : Ohshima)**

Address:

National Space Development Agency of Japan (NASDA)

Medical Research and Operations Office, Tsukuba Space Center (TKSC), 2-1-1 Sengen, Tsukuba-shi

305-8505 IBARAKI - Japon

Phone: +81 (298) 54.3951 / Fax: +81 (298) 50.2232

Mobile:

E-mail: Ohshima.Hiroshi@nasda.go.jp

Function: **PI**; **Representative of NASDA (Co-Sponsor); Member of the Program Committee**

Protocol code: AREDIA; OHS-BS; OHS-BS add; OHS-ACTI; OHS-IMP; OHS-RADIO; OHS-URS; OHS-URS Ca; OHS-STOOLS Ca; OHS HEIGHT; OHS-LBS; ITR-DEXA; ITR-CYBEX; ITR-NMRI; OHS-SLEEP

**Dr. SEKIGUCHI Chiharu (PI : Ohshima)**

Address:

National Space Development Agency of Japan (NASDA)

Medical Research and Operations Office, Tsukuba Space Center (TKSC), 2-1-1 Sengen, Tsukuba-shi

305-8505 IBARAKI - Japon

Phone: +81 (298) 54-3950 / Fax: +81 (298) 50-2232 or 52-1687

Mobile:

E-mail: sekiguchi.chiharu@nasda.go.jp

Function: **Co-I**

Protocol code: OHS-IMP

**M. SUDOH Masamichi (PI : Ohshima)**

Address:

National Space Development Agency of Japan (NASDA)

Medical Research and Operations Office, Tsukuba Space Center (TKSC), 2-1-1 Sengen, Tsukuba-shi

305-8505 IBARAKI - Japon

Phone: +81 (298) 54-3952 / Fax: +81 (298) 50-2232

Mobile:

E-mail: sudoh.masamichi@nasda.go.jp

Function: **Co-I**

Protocol code: OHS-IMP

**Mlle WATANABE Yukiko (PI : Ohshima)**

Address:

National Space Development Agency of Japan (NASDA)

Medical Research and Operations Office, Tsukuba Space Center (TKSC), 2-1-1 Sengen,
Tsukuba-shi

305-8505 IBARAKI - Japon

Phone: +81 (298) 54-3947 / Fax: +81 (298) 50-2232

Mobile:

E-mail: watanabe.yukiko@nasda.go.jp

Function: **Co-I**

Protocol code: OHS-BS; OHS-BS add; OHS-ACTI; OHS-IMP; OHS-RADIO; OHS-URS; OHS-URS Ca; OHS-STOOLS Ca; OHS HEIGHT; OHS-LBS; ITR-DEXA; ITR-CYBEX; ITR-NMRI; OHS-SLEEP

**Pr. BENHAMOU Claude L (PI : Felsenberg)**

Address:

CHR Orléans La Source

Service de Rhumatologie, Hôpital Porte Madeleine, BP 6709

45067 ORLEANS Cedex 2 - France

Phone: +33 (02) 38 77 40 14 / Fax:

Mobile:

E-mail:

Function: **Co-I**

Protocol code: FEL-RADIO

**Me FEILCKE Birthe (PI : Felsenberg)**

Address:

University Hospital Benjamin Franklin

Free University Berlin, Osteoporosis Research Group, Hindenburgdamm 30

12200 BERLIN - Germany

Phone: +49 (30) 8445.3046 / Fax: +49 (30) 8440.9942

Mobile:

E-mail:

Function: **Co-I**

Protocol code: FEL-Ca intake

**Pr. FELSENBERG Dieter (PI : Felsenberg)**

Address:

University Hospital Benjamin Franklin

Free University Berlin, Osteoporosis Research Group, Hindenburgdamm 30

12200 BERLIN - Germany

Phone: +49 (30) 8445.3046 / Fax: +49 (30) 8440.9942

Mobile:

E-mail: felsenberg@ukbf.fu-berlin.de

Function: **PI**

Protocol code: AREDIA; FEL-QCT; FEL-pQCT; ITR-NMRI

**Pr. FRIEDRICHSEN Hans (PI : Felsenberg)**

Address:

Free University Berlin

Institute of Mineralogy, Malteserstrasse 74,

12249 BERLIN - Allemagne

Phone: / Fax: +49 (30) 838,70153

Mobile:

E-mail: hfriedri@zedat.fu-berlin.de

Function: **Co-I**

Protocol code: FEL-Ca intake

**Dr. GIEHL Michael (PI : Felsenberg)**

Address:

University Hospital Benjamin Franklin

Free University Berlin, Osteoporosis Research Group, Hindenburgdamm 30

12200 BERLIN - Germany

Phone: +49 (30) 8445.3056 / Fax: +49 (30) 8440.9942

Mobile:

E-mail: giehl@bigfoot.com

Function: **Co-I**

Protocol code: FEL-Ca intake

**Dr. GUNGA Haans-Christian (PI : Felsenberg)**

Address:

Free Univesisty of BERLIN

Department of Physiology, Arnimallee 22,

D-14195 BERLIN - Allemagne

Phone: +49 (30) 8445.1656 / Fax: +49 (30) 838 2507

Mobile:

E-mail: gunga@zedat.fu-berlin.de

Function: **Co-I**

Protocol code: FEL-US; FEL-BS

**Pr. KIRSCH Karl A (PI : Felsenberg)**

Address:

Free Univesisty of BERLIN

Department of Physiology, Arnimallee 22,

D-14195 BERLIN - Allemagne

Phone: +49 (30) 838 2532 / Fax: +49 (30) 838 2507

Mobile:

E-mail: kkirsch@zedat.fu-berlin.de

Function: **Co-I**

Protocol code: FEL-US; FEL-BS

**Dr. RITTWEGER Jörn (PI : Felsenberg)**

Address:

Free University Berlin

Department of Physiology, Arnimallee 22,

14195 BERLIN - Allemagne

Phone: +49 (30) 8445.1696 / Fax: +49 (30) 8440.9942

Mobile:

E-mail: ritmus@zedat.fu-berlin.de

Function: **Co-I**

Protocol code: AREDIA; FEL-QCT; FEL-pQCT; ITR-NMRI; FEL-BS; FEL-URS; FEL-RADIO; FEL-Ca intake; ITR-DEXA; ITR-NMRI; FEL-IMP

**Dr. EIKEN Ola (PI : Tesch)**

Address:

National Defence Research Establishment (FOA)

Aviation Medicine Dept., Karolinska Institute, Berzelius v13

S-171 77 STOCKHOLM - Suède

Phone: +46 (8) 728-6342/ Fax: +46 (8) 330-923

Mobile:

E-mail: ola.eiken@fyfa.ki.se

Function: **Co-I**

Protocol code: TESCH-HYPERBARY

**Mlle EKBERG Anneli (PI : Tesch)**

Address:

South Hospital

Department of Surgery,

S-118 83 STOCKHOLM - Suède

Phone: / Fax:

Mobile:

E-mail:

Function: **Co-I**

Protocol code: Biopsy; EX

**M. KOLEGARD Roger (PI : Tesch)**

Address:

National Defence Research Establishment (FOA)

Aviation Medicine Dept., Karolinska Institute, Berzelius v13

S-171 77 STOCKHOLM - Suède

Phone: +46 (8) 728-6342/ Fax: +46 (8) 330-923

Mobile:

E-mail:

Function: **Co-I**

Protocol code: TESCH-HYPERBARY

**M. LINDBORG Bertil (PI : Tesch)**

Address:

Karolinska Institute

Section of Environmental Physiology, Dept. of Physiology and Pharmacology,

S-171 77 STOCKHOLM - Suède

Phone: +46 (8) 728-6400/ Fax:

Mobile:

E-mail: bertil.lindborg@fyfa.ki.se

Function: **Co-I**

Protocol code: TESCH-HYPERBARY

**M. MEKJAVIC Igor B. (PI : Tesch)**

Address:

Jozef Stefan Iinstitute

Department of Automation, Biocybernetics & Robotics, Jamova 39

SI-1000 LJUBLJANA - Slovénie

Phone: +386 (61) 177-3358/ Fax: +386 (61) 442-433

Mobile: +386 (41) 696-558

E-mail: igor_mekjavic@email.com

Function: **Co-I**

Protocol code: TESCH-HYPERBARY

**M. NADER Gustavo A. (PI : Tesch)**

Address:

University of Illinois at Chicago; The

School of Kinesiology (M/C 194), 901 W. Roosevelt Road,

60608-1516IL CHICAGO - États-Unis

Phone: / Fax:

Mobile:

E-mail:

Function: **Co-I**

Protocol code: EX; ITR-CYBEX

**Pr. TESCH Per A. (PI : Tesch)**

Address:

Huddinge University Hospital

Medical Laboratory Sciences and Technology, Dept. Clinical Physiology, Huddinge Sjukhns

14186 HUDDINGE - Sweden

Phone: +46 (8) 5858 7226 / Fax: +46 (8) 774 8082

Mobile:

E-mail: teschpera@exchange.uams.edu

Function: **PI**

Protocol code: EX; ITR-CYBEX; Biopsy; ITR-NMRI

**M. TRAPPE Todd A. (PI : Tesch)**

Address:

DWR Center on Aging

Nutrition, Metabolism and Exercise Laboratory, 4301 W. Markham, Slot 806,

72205AR LITTLE ROCK - États-Unis

Phone: +1 (501) 526.5708 / Fax: +1 (501) 526.5710

Mobile:

E-mail: trappetodda@exchange.uams.edu

Function: **Co-I**

Protocol code: EX; ITR-CYBEX; Biopsy; ITR-NMRI

**M. TRIESCHMANN Jay T. (PI : Tesch)**

Address:

DWR Center on Aging

Nutrition, Metabolism and Exercise Laboratory, 4301 W. Markham, Slot 806,

72205AR LITTLE ROCK - États-Unis

Phone: +1 (501) 526.5714 / Fax: +1 (501) 526.5710

Mobile:

E-mail: trieschmannjayt@exhange.uams.edu

Function: **Co-I**

Protocol code: EX; ITR-CYBEX; Biopsy; ITR-NMRI

**Dr. CERCHIELLO Manfredo (PI : Pagani)**

Address:

Via Toscanini 3,

22071 CADORAGO (COMO) - Italie

Phone: +39 (031) 885.048 / Fax: +39 (031) 885.015

Mobile:

E-mail: manfredoc@libero.it

Function: **Co-I**

Protocol code: PAGANI

**Dr. CUTOLO de ROSIS Andrea (PI : Pagani)**

Address:

University of Milano

Medicina Interna I, CTNV, Ospedale L. Sacco, Via G.B. Grassi, 74

20157 MILAN - Italy

Phone: +39 (02) 3904.2748 / Fax: +39 (02) 3820.1396

Mobile:

E-mail: giovannam@fisiopat.sacco.unimi.it

Function: **Co-I**

Protocol code: PAGANI

**Dr. IELLAMO Ferdinando (PI : Pagani)**

Address:

Space Biomedicine Center

Dip. Med. Int., Univers. Di Roma 'Tor vergata', Via O. Raimondo

00173 ROMA - Italy

Phone: +39 (06) 725 94 219 / Fax: +39 (06) 725 94 263

Mobile:

E-mail: iellamo@med.uniroma2.it

Function: **Co-I**

Protocol code: PAGANI

**Pr. PAGANI Massimo (PI : Pagani)**

Address:

University of Milano

Medicina Interna I, CTNV, Ospedale L. Sacco, Via G.B. Grassi, 74

20157 MILAN - Italy

Phone: +39 (02) 3904.2748 / Fax: +39 (02) 3820.1396

Mobile: +39 (335) 819.6882

E-mail: massimop@fisiopat.sacco.unimi.it

Function: **PI**

Protocol code: PAGANI

**Dr. PIZZINELLI Paolo (PI : Pagani)**

Address:

University of Milano

Medicina Interna I, CTNV, Ospedale L. Sacco, Via G.B. Grassi, 74

20157 MILAN - Italy

Phone: +39 (02) 3904.2748 / Fax: +39 (02) 3820.1396

Mobile:

E-mail: giovannam@fisiopat.sacco.unimi.it

Function: **Co-I**

Protocol code: PAGANI

**Dr. ASTIER Catherine (PI : Marini)**

Address:

Université de Nice-Sophia-Antipolis

UMR CNRS 6548, Faculté des Sciences, Parc Valrose

06108 NICE cedex 2 - France

Phone: +33 (-04) 9207.6876 / Fax: +33 (04) 92 07 68 76

Mobile:

E-mail: astier@taloa.unice.fr

Function: **Co-I**

Protocol code: MAR-BS; Biopsy

**Mr. CARNINO Alain (PI : Marini)**

Address:

UPR CNRS 9041

Unité de Neurocybernétique Cellulaire, 280 boulevard de Sainte Marguerite,

13009 MARSEILLE - France

Phone: / Fax:

Mobile:

E-mail:

Function: **Co-I**

Protocol code: MAR-BS; Biopsy

**Mlle CHOPARD Angèle (PI : Marini)**

Address:

Université de Nice-Sophia-Antipolis

UMR CNRS 6548, Faculté des Sciences, Parc Valrose

06108 NICE cedex 2 - France

Phone: +33 (04) 92 07 68 76 / Fax: +33 (04) 92 07 68 76

Mobile:

E-mail: achopard@unice.fr

Function: **Co-I**

Protocol code: MAR-BS; Biopsy

**Me LAPEYSSONNIE Françoise (PI : Marini)**

Address:

Université de Nice-Sophia-Antipolis

UMR CNRS 6548, Faculté des Sciences, Parc Valrose

06108 NICE cedex 2 - France

Phone: +33 (-04) 9207.6876 / Fax: +33 (04) 92 07 68 76

Mobile:

E-mail:

Function: **Co-I**

Protocol code: MAR-BS; Biopsy

**Pr. MARINI Jean-François (PI : Marini)**

Address:

Université de Nice-Sophia-Antipolis

UMR CNRS 6548, Faculté des Sciences, Parc Valrose

06108 NICE cedex 2 - France

Phone: +33 (4) 92 07 68 90 / Fax: +33 (4) 92 07 68 90

Mobile: +33 (6) 82 81 47 09

E-mail: jfmarini@unice.fr

Function: **PI**

Protocol code: MAR-BS; Biopsy

**Mlle ROFFINO Sandrine (PI : Marini)**

Address:

UFR STAPS Nice

261, Route de Grenoble,

06205 NICE cedex 3 - France

Phone: / Fax: +33 (04) 92 29 65 36

Mobile:

E-mail: roffino@unice.fr

Function: **Co-I**

Protocol code: MAR-BS; Biopsy

**Mlle BAREILLE Marie-Pierre (PI : Bareille)**

Address:

Clinique Spatiale-MEDES

CHU Rangueil, 1 Avenue Jean Poulhès,

31403 TOULOUSE cedex 4 - France

Phone: +33 (05) 62 17 49 55 / Fax: +33 (05) 62 17 49 51

Mobile:

E-mail: Marie-Pierre.Bareille@medes.cnes.fr

Function: **PI**

Protocol code: BAREILLE

**Dr. DELVAUX Michel (PI : Bareille)**

Address:

Hôpitaux de Toulouse

CHU Rangueil, Service de Gastroentérologie et Nutrition, 1 Avenue J. Poulhes

31403 TOULOUSE cedex 4 - France

Phone: +33 (5) 61 32 24 52 / Fax:

Mobile:

E-mail: delvaux.m@chu-toulouse.fr

Function: **Co-I**

Protocol code: BAREILLE

**Mlle GANDIA Peggy (PI : Bareille)**

Address:

Hôpitaux de Toulouse

Laboratoire de Pharmacocinétique et Toxicologie Clinique

CHU Rangueil, 1 Avenue J. Poulhès

31403 TOULOUSE cedex 4 - France

Phone: +33 (5) 61 32 24 45 / Fax: +33 (5) 61 32 22 51

Mobile:

E-mail: gandia_chu@yahoo.fr

Function: **Co-I**

Protocol code: BAREILLE

**Pr. HOUIN Georges (PI : Bareille)**

Address:

Hôpitaux de Toulouse

Laboratoire de Pharmacocinétique et Toxicologie Clinique

CHU Rangueil, 1 Avenue J. Poulhès

31403 TOULOUSE cedex 4 - France

Phone: +33 (5) 61 32 24 66 / Fax: +33 (5) 61 32 22 51

Mobile:

E-mail: houin.g@chu-toulouse.fr

Function: **Co-I**

Protocol code: BAREILLE

**Dr. LAVIT Michel (PI : Bareille)**

Address:

Hôpitaux de Toulouse

Laboratoire de Pharmacocinétique et Toxicologie Clinique

CHU Rangueil, 1 Avenue J. Poulhès

31403 TOULOUSE cedex 4 - France

Phone: +33 (5) 61 32 31 62 / Fax: +33 (5) 61 32 22 51

Mobile:

E-mail: lavit.m@chu-toulouse.fr

Function: **Co-I**

Protocol code: BAREILLE

**Dr. PAVY-LE TRAON Anne (PI : Bareille)**

Address:

Clinique Spatiale-MEDES

CHU Rangueil, 1 Avenue Jean Poulhès,

31403 TOULOUSE cedex 4 - France

Phone: +33 (05) 62 17 49 52 / Fax: +33 (05) 62 17 49 51

Mobile: +33 (06) 74 40 29 62

E-mail: Anne.Pavy-LeTraon@medes.cnes.fr

Function: **Co-I**

Protocol code: BAREILLE

**Dr. SAIVIN Sylvie (PI : Bareille)**

Address:

Hôpitaux de Toulouse

Laboratoire de Pharmacocinétique et Toxicologie Clinique

CHU Rangueil, 1 Avenue J. Poulhès

31403 TOULOUSE cedex 4 - France

Phone: +33 (5) 61 32 24 66 / Fax: +33 (5) 61 32 22 51

Mobile:

E-mail: saivin.s@chu-toulouse.fr

Function: **Co-I**

Protocol code: BAREILLE

**Pr. BAUM Klaus (PI : Baum)**

Address:

Dürenerstrasse 411,

50858 COLOGNE - Germany

Phone: +49 (221) 278 1052 / Fax: +49 (221) 278 1054

Mobile:

E-mail: baum@professor-baum.de

Function: **PI**

Protocol code: BACKPAIN

**Pr. ESSFELD Dieter (PI : Baum)**

Address:

German Sport university Cologne

Carl-Diem-Weg 6,

50933 COLOGNE - Allemagne

Phone: +49 (221) 4982-219 / Fax: +49 (221) 4973-531

Mobile:

E-mail: essfeld@hrz.dshs-koeln.de

Function: **Co-I**

Protocol code: BACKPAIN

**M. LANGENOHL Mike (PI : Baum)**

Address:

German Sport university Cologne

Carl-Diem-Weg 6,

50933 COLOGNE - Allemagne

Phone: +49 (221) 4982-696 / Fax: +49 (221) 4982-673

Mobile:

E-mail: langenohl@hrz.dshs-koeln.de

Function: **Co-I**

Protocol code: BACKPAIN

**Dr. RUETHER Thomas (PI : Baum)**

Address:

German Sport university Cologne

Carl-Diem-Weg 6,

50933 COLOGNE - Allemagne

Phone: +49 (221) 4982-696 / Fax: +49 (221) 4982-673

Mobile:

E-mail: ruether@hrz.dshs-koeln.de

Function: **Co-I**

Protocol code: BACKPAIN

**Mr. APPEL Roy (PI : Blottner)**

Address:

Universitätkrankenhaus Eppendorf of Hamburg

Neurologische Klinik und Poliklinik, Martinistrasse 52,

D-20246 HAMBURG - Allemagne

Phone: +49 (40) 42803-3771 / Fax: +49 (40) 42803-5086

Mobile:

E-mail:

Function: **Co-I**

Protocol code: Biopsy

**Pr. BLOTTNER Dieter (PI : Blottner)**

Address:

Free University Berlin

Department of Anatomy 1, Neuronbiology Group, University Hospital Benjamin Franklin,

Königin-Luise-Strasse 15

14195 BERLIN-DAHLEM - Germany

Phone: +49 (30) 8445.1903 / Fax: +49 (30) 8445.1902

Mobile:

E-mail: blottner@zedat.fu-berlin.de

Function: **PI**

Protocol code: Biopsy

**Mlle LÜCK Gabriele H (PI : Blottner)**

Address:

Free University Berlin

Department of Anatomy 1, Neuronbiology Group, University Hospital Benjamin Franklin,

Königin-Luise-Strasse 15

14195 BERLIN-DAHLEM - Germany

Phone: +49-30.8445.1903 / Fax: +49-30.8445.1902

Mobile:

E-mail: luck@zedat.fu-berlin.de

Function: **Co-I**

Protocol code: Biopsy

**Mlle RINDERMANN Catrin (PI : Blottner)**

Address:

Free University Berlin

Department of Anatomy 1, Neuronbiology Group, University Hospital Benjamin Franklin,

Königin-Luise-Strasse 15

14195 BERLIN-DAHLEM - Germany

Phone: +49 (30) 8445.1903 / Fax: +49 (30) 8445.1902

Mobile:

E-mail:

Function: **PI**

Protocol code: Biopsy

**Mlle SCHOSER Benedikt G.H. (PI : Blottner)**

Address:

Universitätkrankenhaus Eppendorf of Hamburg

Neurologische Klinik und Poliklinik, Martinistrasse 52,

D-20246 HAMBURG - Allemagne

Phone: +49 (40) 42803-3771 / Fax: +49 (40) 42803-5086

Mobile:

E-mail: Schoser@uke.uni-hamburg.de

Function: **Co-I**

Protocol code: Biopsy

**Pr. ANTONUTTO Guglielmo (PI : Ferretti)**

Address:

Universita' degli Studi di Udine

Dipartimento di Scienze e Tecnologie Biomediche, P. Le Kolbe 4,

33100 UDINE - Italie

Phone: +39 (0432) 494.334 / Fax: +39 (0432) 494.301

Mobile:

E-mail: GAntonutto@makek.dstb.uniud.it

Function: **Co-I**

Protocol code: ITR-VO2; FER-Q

**Dr. AZABJI Marcel K (PI : Ferretti)**

Address:

Centre Médical Universitaire

Département de Physiologie

9, avenue de Champel,

CH-1211 GENEVE 4 - Suisse

Phone: +41 (22) 7025 375 / Fax: +41 (22) 7025 402

Mobile:

E-mail: marcel.azabji@medecine.unige.ch

Function: **Co-I**

Protocol code: ITR-VO2; FER-Q

**Dr. BINZONI Tiziano (PI : Ferretti)**

Address:

Hôpital Univeristaire de Genève

Dept. de Radiologie, Rue Micheli de Crest,

1211 GENEVE 4 - Suisse

Phone: +41 (22) 702 5358 / Fax: +41 (22) 702 5402

Mobile:

E-mail: binzoni@medecine.unige.ch

Function: **Co-I**

Protocol code: FER-A

**M. BLANC Stéphane (PI : Ferretti)**

Address:

University of Wisconsin - Madison

Department of Nutrional Sciences, 1415 Linden Drive,

53706-1571WI MADISON - États-Unis

Phone: +1 (608) 263.5477 / Fax: +1 (608) 262.5860

Mobile:

E-mail: blanc@nutrisci.wisc.edu

Function: **Co-I**

Protocol code: FER-BodyComp; FER-METABO; FER-BV

**Dr. CAPELLI Carlo (PI : Ferretti)**

Address:

Universita' degli Studi di Udine

Dipartimento di Scienze e Tecnologie Biomediche, P. Le Kolbe 4,

33100 UDINE - Italie

Phone: +39 (0432) 494.335 / Fax: +39 (0432) 494.301

Mobile:

E-mail: Carlo.Capelli@dstb.uniud.it

Function: **Co-I**

Protocol code: ITR-VO2; FER-Q

**Mlle CAUTERO Michela (PI : Ferretti)**

Address:

Universita' degli Studi di Udine

Dipartimento di Scienze e Tecnologie Biomediche, P. Le Kolbe 4,

33100 UDINE - Italie

Phone: +39 (0432) 494.332 / Fax: +39 (0432) 494.301

Mobile:

E-mail: mcautero@makek.dstb.uniud.it

Function: **Co-I**

Protocol code: ITR-VO2; FER-Q

**Dr. CUSTAUD Marc-Antoine (PI : Ferretti)**

Address:

Laboratoire de Physiologie de l'Environnement

Faculté de Médecine Grange-Blanche

8 Avenue Rockefeller,

69373 LYON Cedex 08 - France

Phone: +33 (04) 78 77 70 75 / Fax: +33 (04) 78 01 74 67

Mobile:

E-mail: mcustaud@club-internet.fr

Function: **Co-I**

Protocol code: FER-BV; FER-S

**Dr. D'ANTONA Giuseppe (PI : Ferretti)**

Address:

University of Pavia

Institute of Human Physiology, Via Forlanini 6,

27100 PAVIA - Italie

Phone: +39 (0382) 507,664 / Fax: +39 (0382) 507,256

Mobile:

E-mail:

Function: **Co-I**

Protocol code: ITR-VO2; FER-Q

**Pr. DI PRAMPERO Pietro E. (PI : Ferretti)**

Address:

Universita' degli Studi di Udine

Dipartimento di Scienze e Tecnologie Biomediche, P. Le Kolbe 4,

33100 UDINE - Italie

Phone: +39 (0432) 494.330 / Fax: +39 (0432) 494.301

Mobile:

E-mail: pprampero@makek.dstb.uniud.it

Function: **Co-I**

Protocol code: ITR-VO2; FER-Q

**Dr. FERRETTI Guido (PI : Ferretti)**

Address:

Centre Médical Universitaire

Département de Physiologie

9, avenue de Champel,

CH-1211 GENEVE 4 - Suisse

Phone: +41 (22) 7025 363 / Fax: +41 (22) 7025 402

Mobile:

E-mail: Guido.Ferretti@medecine.unige.ch

Function: **PI**

Protocol code: ITR-VO2; FER-Q

**Dr. FORTRAT Jacques-Olivier (PI : Ferretti)**

Address:

Centre Hospitalier d'Angers

Laboratoire d'Explorations Fonctionnelles Vasculaires, ,

49003 ANGERS Cedex 01 - France

Phone: +33 (02) 41 35 36 67 / Fax: +33 (02) 41 35 50 42

Mobile:

E-mail: Explovasc@chu-angers.fr

Function: **Co-I**

Protocol code: FER-S

**Me FUSI Simonetta (PI : Ferretti)**

Address:

Universita' degli Studi di Udine

Dipartimento di Scienze e Tecnologie Biomediche, P. Le Kolbe 4,

33100 UDINE - Italie

Phone: +39 (0432) 494.333 / Fax: +39 (0632) 494.301

Mobile:

E-mail: sfusi@makek.dstb.uniud.it

Function: **Co-I**

Protocol code: ITR-VO2; FER-Q

**Dr. GEELEN Ghislaine (PI : Ferretti)**

Address:

Laboratoire de Physiologie de l'Environnement

Faculté de Médecine Grange-Blanche

8 Avenue Rockefeller,

69373 LYON Cedex 08 - France

Phone: +33 (04) 78 77 70 75 / Fax: +33 (04) 78 01 74 67

Mobile:

E-mail:

Function: **Co-I**

Protocol code: FER-BV

**Pr. GHARIB Claude (PI : Ferretti)**

Address:

Laboratoire de Physiologie de l'Environnement

Faculté de Médecine Grange-Blanche

8 Avenue Rockefeller,

69373 LYON Cedex 08 - France

Phone: +33 (04) 78 77 70 75 / Fax: +33 (04) 78 01 74 67

Mobile: +33 (06) 08 42 90 09

E-mail: gharib@univ-lyon1.fr

Function: **I**

Protocol code: FER-BV; FER-S; FER-METABO; FER-BodyComp

**Dr. LADOR Frédéric (PI : Ferretti)**

Address:

Centre Médical Universitaire

Département de Physiologie

9, avenue de Champel,

CH-1211 GENEVE 4 - Suisse

Phone: +41 (22) 7025 365 / Fax: +41 (22) 7025 402

Mobile:

E-mail: Frederic.Lador@medecine.ungie.ch

Function: **Co-I**

Protocol code: ITR-VO2; FER-Q

**Dr. LAVILLE Martine (PI : Ferretti)**

Address:

Centre de Recherche en Nutrition Humaine de Lyon

Faculté de Médecine RTH Laennec

8, rue Guillaume Parradin,

69372 LYON cedex 08 - France

Phone: +33 (04) 78 00 98 98 / Fax: +33 (04) 78 77 87 62

Mobile:

E-mail: mlaville@laennec.univ-lyon1.fr

Function: **Co-I**

Protocol code: FER-BodyComp; FER-METABO

**Pr. LINNARSSON Dag (PI : Ferretti)**

Address:

Karolinska Institute

Section of Environmental Physiology, Dept. of Physiology and Pharmacology,

S-171 77 STOCKHOLM - Suède

Phone: +46 (8) 728 6890 / Fax: +46 (8) 339 702

Mobile:

E-mail: Dag.Linnarsson@fyfa.ki.se

Function: **Co-I**

Protocol code: ITR-VO2; FER-Q

**Dr. MAGANARIS Constantinos N (PI : Ferretti)**

Address:

Manchester Metropolitan University

Dept. of Exercise and Sport Science, Hassal Road,

ST7 2HLCheshire ALSAGER - Grande-Bretagne

Phone: +44 (161) 247.5585 / Fax: +44 (161) 247.6375

Mobile:

E-mail: c.constantinos@mmu.ac.uk

Function: **Co-I**

Protocol code: FER-LOCO

**Mlle MILLET Catherine (PI : Ferretti)**

Address:

Laboratoire de Physiologie de l'Environnement

Faculté de Médecine Grange-Blanche

8 Avenue Rockefeller,

69373 LYON Cedex 08 - France

Phone: +33 (04) 78 77 70 75 / Fax: +33 (04) 78 01 74 67

Mobile: +33 (06) 86 99 54 29

E-mail:

Function: **Co-I**

Protocol code: FER-BV

**Pr. MINETTI Alberto E (PI : Ferretti)**

Address:

Manchester Metropolitan University

Dept. of Exercise and Sport Science, Hassal Road,

ST7 2HLCheshire ALSAGER - Grande-Bretagne

Phone: +44 (161) 247.5585 / Fax: +44 (161) 247.6375

Mobile:

E-mail: a.e.minetti@mmu.ac.uk

Function: **Co-I**

Protocol code: FER-LOCO

**M. MOIA Christian (PI : Ferretti)**

Address:

Centre Médical Universitaire

Département de Physiologie

9, avenue de Champel,

CH-1211 GENEVE 4 - Suisse

Phone: +41 (22) 7025 345 / Fax: +41 (22) 7025 402

Mobile:

E-mail: Christian.Moia@medecine.unige.ch

Function: **Co-I**

Protocol code: ITR-VO2; FER-Q

**Dr. NARICI Marco V (PI : Ferretti)**

Address:

Manchester Metropolitan University

Dept. of Exercise and Sport Science, Hassal Road,

ST7 2HLCheshire ALSAGER - Grande-Bretagne

Phone: +44 (161) 247.5659 / Fax: +44 (161) 247.6375

Mobile:

E-mail: m.narici@mmu.ac.uk

Function: **Co-I**

Protocol code: FER-A

**Mlle NORMAND Sylvie (PI : Ferretti)**

Address:

Centre de Recherche en Nutrition Humaine de Lyon

Faculté de Médecine RTH Laennec

8, rue Guillaume Parradin,

69372 LYON cedex 08 - France

Phone: +33 (04) 78 00 98 98 / Fax: +33 (04) 78 77 87 62

Mobile:

E-mail: snormand@laennec.univ-lyon1.fr

Function: **Co-I**

Protocol code: FER-BodyComp; FER-METABO

**Pr. PENDERGAST Dave R (PI : Ferretti)**

Address:

State University of New-York at Buffalo

Department of Physiology, 124 Sherman Hall,

14214NY BUFFALO - États-Unis

Phone: +1 (716) 829-3830 / Fax: +1 (716) 8292 384

Mobile:

E-mail: dpenderg@acsu.buffalo.edu

Function: **Co-I**

Protocol code: ITR-VO2; FER-Q

**Dr. SANDERS Michael (PI : Ferretti)**

Address:

University of Copenhagen

Copenhagen Muscle Research Center, Rigshospitalet,

DK-2200 COPENHAGEN - Danemark

Phone: +45 (35) 457.614 / Fax: +45 (35) 457.634

Mobile:

E-mail: sanders@dadlnet.dk

Function: **Co-I**

Protocol code: FER-Smicro

**Pr. SCHOELLER Dale A (PI : Ferretti)**

Address:

University of Wisconsin - Madison

Department of Nutrional Sciences, 1415 Linden Drive,

53706-1571WI MADISON - États-Unis

Phone: +1 (608) 263.5477 / Fax: +1 (608) 262.5860

Mobile:

E-mail: schoeller@nutrisci.wisc.edu

Function: **Co-I**

Protocol code: FER-BodyComp; FER-METABO

**Me ZAMPARO Paola (PI : Ferretti)**

Address:

Universita' degli Studi di Udine

Dipartimento di Scienze e Tecnologie Biomediche, P. Le Kolbe 4,

33100 UDINE - Italie

Phone: / Fax: +39 (0432) 494.301

Mobile:

E-mail: PZamparo@makek.dstb.uniud.it

Function: **Co-I**

Protocol code: ITR-VO2; FER-Q

**Pr. ARBEILLE Philippe (PI : Louisy; Bareille)**

Address:

Unité de Médecine et Physiologie Spatiales

CHU Trousseau, Dept. Médecine Nucléaire et Ultrasons,

37044 TOURS Cedex - France

Phone: +33 (02) 47 47 59 39 / Fax: +33 (02) 47 47 59 13

Mobile: +33 (6) 80 10 54 88

E-mail: arbeille@med.univ-tours.fr

Function: **Co-I**

Protocol code: LOUI-PLETH-US; STD LOUISY; BAREILLE

**Dr. LOUISY Francis (PI : Louisy)**

Address:

37 rue de la Mairie, ,

91590 D'HUISON-LONGUEVILLE - France

Phone: +33 (01) 64 57 42 81 / Fax: +33 (01) 64 57 42 81

Mobile: +33 (06) 09 76 63 50

E-mail: louisy@club-internet.fr

Function: **PI**

Protocol code: LOUI-PLETH-US; STD LOUISY

**Dr. ROUMY Jérôme (PI : Louisy)**

Address:

Unité de Médecine et Physiologie Spatiales

CHU Trousseau, Dept. Médecine Nucléaire et Ultrasons,

37044 TOURS Cedex - France

Phone: +33 (02) 47 47 59 39 / Fax: +33 (02) 47 47 59 13

Mobile: +33 (06) 13 13 49 02

E-mail: roumy_j@med.univ-tours.fr

Function: **Co-I**

Protocol code: LOUI-PLETH-US; STD LOUISY; BAREILLE

**Dr. DUPUI Philippe (PI : Dupui)**

Address:

GSBMS

Université de Médecine

133, Route de Narbonne,

31062 TOULOUSE - France

Phone: +33 (05) 62 88 90 00 ext 532 / Fax: +33 (05) 62 17 20 10

Mobile:

E-mail: dupui@cict.fr

Function: **PI**

Protocol code: ITR-Equitest

# agencies representatives

**M. ELMANN-LARSEN Benny**

Address:

European Space Agency

ESA-ESTEC-GS, Keplerlaan 1, Postbus 299

NL-2201 AG NOORDWIJK - Pays-Bas

Phone: +31 (71) 565 3322 / Fax: +31 (71) 565 4993

Mobile: +31 (6) 27433207

E-mail: belmann@estec.esa.nl

Function: **Project Manager; Member of the coordination group**

Protocol code:

**Me GAUQUELIN-KOCH Guillemette**

Address:

Centre National d'Etudes Spatiales

CNES/DPI/E2U, 2 Place Maurice Quentin,

75039 PARIS cedex 01 - France

Phone: +33 (01) 44 76 78 87 / Fax: +33 (01) 44 76 78 59

Mobile:

E-mail: Guillemette.GauquelinKoch@cnes.fr

Function: **Deputy Project Manager; Member of the coordination group**

Protocol code:

**Dr. GUELL Antonio**

Address:

Centre National d'Etudes Spatiales

CNES/DPI/E2U, 18 Avenue Edouard Belin,

31401 TOULOUSE Cedex 4 - France

Phone: 05.61.28.25.77 abr. 1026 / Fax: 05.61.27.30.91

Mobile: 06.08.21.24.77

E-mail: Antonio.Guell@cnes.fr

Function: **Representative of CNES (Study Promoter); Member of the Program Committee**

Protocol code:

**Dr. OHSHIMA Hiroshi (PI : Ohshima)**

Address:

National Space Development Agency of Japan (NASDA)

Medical Research and Operations Office, Tsukuba Space Center (TKSC), 2-1-1 Sengen, Tsukuba-shi

305-8505 IBARAKI - Japon

Phone: +81 (298) 54.3951 / Fax: +81 (298) 50.2232

Mobile:

E-mail: Ohshima.Hiroshi@nasda.go.jp

Function: **PI**; **Representative of NASDA (Co-Sponsor); Member of the Program Committee**

Protocol code: AREDIA; OHS-BS; OHS-BS add; OHS-ACTI; OHS-IMP; OHS-RADIO; OHS-URS; OHS-URS Ca; OHS-STOOLS Ca; OHS HEIGHT; OHS-LBS; ITR-DEXA; ITR-CYBEX; ITR-NMRI; OHS-SLEEP

**Dr. SCHMITT Didier A**

Address:

European Space Agency

ESA-ESTEC-GS, Keplerlaan 1, Postbus 299

NL-2201 AG NOORDWIJK - Pays-Bas

Phone: 00 31 71 565 4888 N.Abr. 1105 / Fax: 00 31 71 565 3042

Mobile: 06.07.36.49.17

E-mail: dschmitt@estec.esa.nl

Function: **Representative of ESA (Co-Sponsor);** **Member of the Program Committee**

Protocol code:

**Me SUENSON Rosita**

Address:

European Space Agency

NL-2201 AZ NOORDWIJK - Pays-Bas

Phone: 00 31 71 565 5218 / Fax: 00 31 71 565 3661

Mobile:

E-mail: rsuenson@estec.esa.nl

Function: **Public Relations**

Protocol code:

# MEDES STAFF

**M. BRAAK Laurent**

Address:

Clinique Spatiale-MEDES

CHU Rangueil, 1 Avenue Jean Poulhès,

31403 TOULOUSE cedex 4 - France

Phone: (05) 62 17 49 75 / Fax: (05) 62 17 49 51

Mobile: (06) 74 40 29 64

E-mail: Laurent.Braak@medes.cnes.fr

Function: **MEDES Executive Director**

Protocol code:

**Dr. COSTES-SALON Marie-Claude**

Address:

Clinique Spatiale-MEDES

CHU Rangueil, 1 Avenue Jean Poulhès,

31403 TOULOUSE cedex 4 - France

Phone: (05) 62 17 49 66 / Fax: (05) 62 17 49 51

Mobile:

E-mail: Marie-Claude.Costes-Salon@medes.cnes.fr

Function: **MD, Medical selection and survey**

Protocol code:

**M. FERREIRA Franck**

Address:

Clinique Spatiale-MEDES

CHU Rangueil, 1 Avenue Jean Poulhès,

31403 TOULOUSE cedex 4 - France

Phone: (05) 62 17 49 56 / Fax: (05) 62 17 49 51

Mobile:

E-mail: Franck.Ferreira@medes.cnes.fr

Function: **Technical Support**

Protocol code:

**M. MAILLET Alain**

Address:

Clinique Spatiale-MEDES

CHU Rangueil, 1 Avenue Jean Poulhès,

31403 TOULOUSE cedex 4 - France

Phone: 05.62.17.49.53 / Fax: 05.62.17.49.51

Mobile: 06.87.70.29.31

E-mail: Alain.Maillet@medes.cnes.fr

Function: **Project Scientist; Member of the coordination group**

Protocol code:

**Dr. PAVY-LE TRAON Anne**

Address:

Clinique Spatiale-MEDES

CHU Rangueil, 1 Avenue Jean Poulhès,

31403 TOULOUSE cedex 4 - France

Phone: +33 (05) 62 17 49 52 / Fax: +33 (05) 62 17 49 51

Mobile: +33 (06) 74 40 29 62

E-mail: Anne.Pavy-LeTraon@medes.cnes.fr

Function: **Coordinating-Investigator; Member of the coordination group**

**Me VASSEUR-CLAUSEN Pascale**

Address:

Clinique Spatiale-MEDES

CHU Rangueil, 1 Avenue Jean Poulhès,

31403 TOULOUSE cedex 4 - France

Phone: (05) 62 17 49 58 / Fax: (05) 62 17 49 51

Mobile:

E-mail: Pascale.Vasseur@medes.cnes.fr

Function: **responsible for volunteer selection and paramedical staff; data collection and quality control**

# pSYCHOLOGICAL TEAM

**Me ROSNET Elisabeth**

Address:

Laboratoire de Psychologie Appliquée

UFRSTAPS, Batiment 6, Campus Moulin de la Housse

51687 REIMS cedex 2 - France

Phone: / Fax: +33 (3) 26 91 36 50

Mobile: +33 (6) 60 15 89 77

E-mail: elisabeth.rosnet@univ-reims.fr

Function: **PI**

Protocol code: Psychological Support

# BACKGROUND AND objectives

Long duration space-flights have been up to now performed within the Russian Space Program. The utilisation of the International Space Station will require flights of many astronauts of a duration from 3 to 6 months. Space environment induces physiological changes which can affect health and performances of astronauts. Living in space environment and specially in weightlessness induces physiological modifications, mainly on the sensori motor and cardio vascular systems, on the muscles and on bones. The bone remodelling which occur during long duration spacefligths are, with psychological factors and effects of radiation (ionising radiation), a limiting factor for long term manned missions, especially missions to Mars. It is therefore necessary to perform experiments during space-flights to improve knowledge on physiological adaptation to space environment and develop preventive methods (countermeasures), pharmacological or physical, to prevent these changes and improve the return to Earth. As a matter of fact, Russian cosmonauts practice daily physical exercise (treadmill, bicycle ergometer) up to 90 min per day.

Considering the limited number of flight opportunities, the difficulties related to the performance of in flight experiments (operational constraints for astronauts, limited capabilities of in-flight biomedical devices), ground based experiments simulating effects of weightlessness are used to better understand the mechanisms of physiological adaptation, design and validate the countermeasures.

The most commonly used ground-based simulations is the anti-orthostatic bed rest (or head-down tilt-HDT). Different positions have been tested. On average, a position of minus six degrees is used. Several factors of space environment are reproduced during head-down tilt experiments : fluid shift, inactivity, confinement.

This model can be used to simulate the effects of space environment mainly on :

- cardiovascular system,

- hydromineral balance and hormonal regulation,

- muscular system,

- bones and phospho calcic balance.

Psychological behaviour and possible changes in circadian rhythms can also be studied.

Within the frame of the utilisation of the International Space Station, the French ( CNES), European (ESA) and Japanese (NASDA) space agencies have decided to join their efforts to organise ground based simulations of international space station flights, using the anti orthostatic bed rest model.

The experimental campaigns will last 4 months : 3 months of bed rest to simulate the effects of a spacefligth, with a 15 days period before and after for the baseline data collection (before bed rest) and the recovery (after bed rest).

The first campaign, which are presented in this report, is planned in 2001 and 2002 with the following objectives :

- Study the adaptation of humans to long term simulated microgravity, specially the muscular modifications and the bone remodelling. Ten scientific teams propose scientific experiments sometimes including several research protocols. These scientific protocols were evaluated and selected by the ESA and NASDA advisory groups.
- Evaluate the effects of countermeasures :
  - Regular exercising using a resistive exercise device (Fly Wheel) to prevent muscular atrophy. The effects of this physical training on bone and cardiovascular functions will also be studied.
  - Administration of biphosphonates (unique infusion of a 60 mg dose of pamidronate (Aredia) to prevent bone modifications.
- Contribute to an international harmonisation and standardisation of the physiological measurements performed on the astronauts before, during and after flights, enabling the evaluation of countermeasures.

Twenty eight healthy volunteers, 25 to 45 years old, non smokers, will participate to this experiment , distributed in 3 groups : 10 control subjects, 9 subjects with regular muscular exercise, 9 subjects with biphosphonates administration. The experiment will be divided in two periods: the first one in 2001,the second one in 2002. 14 volunteers will participate in each period: 5 control subjects, 4 or 5 subjects with muscular exercise or biphosphonates. Each period will last 120 days : 15 days for baseline data collection, 90 days of -6° head-down bed (HDBR) rest, 15 days for recovery. The subjects will be asked to come back for long term recovery measurements 45, 90, 180 and 360 days after the end of the HDBR period + questionnaire with telephone call after 60 days and 2 years.

The experiments will take place in the Space Clinic located within Toulouse Rangueil Hospital.

## Adaptation of humans to long term simulated weightlessness :

The bed-rest model has been used for several years for a ground simulation of the effects of weightlessness, generating modifications in the following areas:

- cardiovascular system: The HDBR induces a 10 % decrease in plasma volume, a de-conditioning of the cardiovascular system, characterized by a higher heart rate at rest, an arterial orthostatic hypotension and a decrease in capacity to perform physical exercise. The hypotension disappears a few days after completion of the bed-rest. About 50% of the volunteers suffered from an ortho-static intolerance sufficient to justify the premature stop of orthostatic tests, after HDBR simulation experiments of various length (from 4 to 42 days) *(Pavy- Le Traon et al, Clinical Physiology, 1999, 19, 5, 360-368 ).* Different factors contribute to the occurrence of ortho-static hypotension: the decrease of the plasma volume, the modifications of the hormones regulating blood volume, and of the cardiovascular control by the autonomic nervous system (baroreflex changes in particular), the increased venous distensibility of the lower limbs. *(Eckberg et Fritsch, J Clin Pharmacol, 1991, 31, 951-955 ; Gharib et Hughson, Advances in Space Biology and Medicine, 1992, 113-130 ; Blomqvist et al., Gravitational Physiology, 1994, 1, 122-124 ; Hughson et Bondar, Handbook of Clinical Neurology, 1999, chapter 9).*
- metabolism: Head-down bed rest induces endocrine and metabolic changes that affect the body composition and the energy requirements necessary to maintain health (*Gretebeck RJ, et al. 1995. J. Appl. Physiol. 78(6) : 2207-2211; Blanc S et al., J. Clin. Endocrinol. Metab. 1998, 83 (12): 4289-4297;Blanc S et al. Am J Physiol 2000, 279(3):R891-8)*. Recent data suggest, but do not demonstrate, that fat metabolism is strongly altered in HDBR *(Blanc S et al., J Clin Endocrinol Metab 2000, 85(6): 2223-33)*  as in flight *(Lane Hw et al. Am. Clin. Nutr. 1997 65 : 4-12, ; Stein TP, et al. Am. J. Physiol. 1999, 276(6) : R1739-R1748)*. Because fat is the highest energy density tissue of the organism, a better understanding of its adaptation to simulated weightlessness is required to plan long-term space flight and make accurate estimates of the astronaut's energy requirements.
- bone system : during space flights, the global decrease of load influences the whole skeleton since the gravitational and reaction forces are negligible. Space flights may be used as accelerated model for osteoporosis affecting the weight-bearing bones (bones of the lower limbs, spinal column).

These modifications are characterized by a reduction of the bone density (decrease of the bone production and increase of the resorption) and a negative calcium balance. A prolonged confinement will generate the same type of modification, though with a slower appearance. A 17 weeks bed-rest experiment showed that the loss of bone density of the whole body varies with each subject between 0.5 and 2.58% (average 1.41%) and correlates with the result of the calcium balance. *(Rubin et al., J. Bone Joint Surg., 1984, 66, 397-402).* A previous 6 weeks confinement experience on 7 subjects showed no significant loss of bone density (but a tendency for reduction at the lumbar vertebra and lower limbs level) *(Uebelhart et al., Osteoporosis Int 2000, 11; 59-67).* A 3 months experimentation duration should generate significant modifications of the bone density. The recovery rate varies with each individual; it was generally accepted that the length of the recovery period is equal to that of the flight. Vico et al. recently showed that a full recovery was not observed in some cosmonauts (6 months space-flight duration) 6 months after the return to Earth, suggesting that the time needed for the recovery is longer the flight duration *(Lancet 2000, 355, 1607-1611)*. However, no data are available in literature on the recovery of prolonged immobilization osteoporosis in healthy volunteers.

- muscular system: space flights as well as prolonged bed-rests lead to an atrophy of the lower limb and para-vertebral muscles, which are concerned by the posture and the locomotion. After a 6 week HDBR experiment, the cross sectional area of the extensor muscles of the knee, evaluated by Nuclear Magnetic Resonance imaging (NMR) showed an average loss of 14 %+/-4%, that of the vastus lateralis of 18+/-4%. The voluntary knee extensor muscles contraction strength was reduced from 25 to 30 % *(Berg et al., J. Appl. Physiol., 1997, 82, 182-188)*. The recovery rate after a 3 months HDBR has never been precisely investigated. Leblanc et al *(J. Appl. Physiol, 73: 2172-2178, 1992)* studied the cross sectional area of the extensor muscles of the lower limbs after a confinement of 17 weeks and demonstrated that after 7 weeks, the values of these parameters were not restored to those observed before the HDBR.
- The loss of exercise capacity observed after the space flights or simulated weightlessness experiments are in relation with the muscles modifications and the cardiovascular adaptation problems.
- spine geometry and movements : space flights and bed-rest experiments generate lumbar pains due to the forced reduced amplitude of the spine movements. This back pain do not seem correlated with the increase of the spine’s length *(Baum et al; Eur J Med Res, 1999, 4, 389-393).*
- sleep and circadian rhythms : they may be modified, particularly by the confinement situation and isolation of the subjects from their social and usual life conditions.
- sensori-motor system : although changes induced by HDBR are less important than those observed after a space flight, some disorders in particular of gait and balance were observed during the first days following the HDBR *(Dupui et al., Aviat. Space Environ. Med , 1992, 63, 1004-1010)*.

In fine, numerous physiological modifications, caused by actual or simulated weightlessness, may influence the pharmacokinetics of the medicaments which could be delivered to the astronauts during space flights ( *Amidon et al., J. Clin Pharmacol, 1991, 31, 968-973 ; Cintrom et al, Doc NASA, 1987) .* A first 4 days HDBR experiment showed that the half life of the lidocain, test drug to assess hepatic metabolism, was reduced by 40% compared to the ambulatory period in sitting position. *(Saivin et al., J. Clin Pharmacol, 1995, 35, 697-704).*  A more recent experiment showed that the bio-availability of promethazine (and used for prevention of space motion sickness) taken orally, increased by 30% during HDBR, suggesting its possible influence on absorption mechanisms.

**These different domains previously listed will be studied during this long duration HDBR experiment :**

- bone and calcium balance : blood and urine bone markers, calcium metabolism, bone density (DEXA), bone architecture (spiralled scanner of the skull, peripheral micro CT scan), influence of the fluid shift on bone changes,
- muscular changes : muscular biopsies (vastus lateralis, soleus), muscular degradation (blood assays of muscular proteins), muscle strength (Cybex), muscular atrophy and structure changes (NMR),
- metabolic changes : Energy expenditure (indirect calorimetry), Substrate oxidation, Dietary fat oxidation, Body composition (impedancemetry, DEXA)
- cardio-vascular adaptation : plasma volume changes (dilution method, indocyanine green), hormonal assays, sympathetic nervous activity (heart rate and blood pressure variability, sympathetic nerves recordings by microneurography), cardiac and vascular adaptation (echo- doppler and plethysmography measurements),
- cardiovascular, respiratory and muscular adaptation to exercise including VO2 max measurements
- spine geometry and movements
- sleep study (actimetry and body temperature recordings)
- pharmaco-kinetics of acetaminophen to assess the influence of the simulated weightlessness on the digestive absorption.

*Detailed rationale and experiment description will be given for each protocol.*

## Evaluation of 2 countermeasures :

### Muscular exercise

There is still on-going discussion and studies to determine the best type and the less time consuming counter-measure to be applied during space flights. The objective of this experiment is to evaluate and to validate a new technique to prevent muscle atrophy and impairment of muscle function designed to be used on the International Space station. A mechanical non gravity dependent exercise device has been developed which uses inertia of fly-wheels to produce resistance (resistive exercise device).

Nine volunteers (exercise group) will perform bilateral resistance exercise on the adapted ergometer every 3 days (start on the 5^th^ day of HDBR). Each session will consist of four sets of 7 maximal concentric-eccentric knee extensor (total duration of each session about 30 min).

### Biphosphonates

Drugs that inhibit bone resorption like biphosphonates are attractive candidates for the prevention and the treatment of osteoporosis. Etidronate (Didronel) has been prescribed since years for prevention and treatment of post-menopausal osteoporosis without significant side effects, but with a potential negative effect on mineralisation. Nevertheless, the new generation of biphosphonates are more efficient anti-resorptive agents. These drugs are generally prescribed and successful in several disorders characterised by excessive bone resorption such as Paget's disease of bone, tumoral osteolysis with skeletal metastases or malignant hypercalcemia. Their use in osteoporosis is still under evaluation, but most studies show encouraging results. Positive effects of pamidronate have been reported in the treatment of osteoporosis associated with corticosteroid treatment (*Gallacher et al., 1992, 47, 932-936* ), with osteogenesis imperfecta *(Glorieux et al, New Engl J Med 339:947-952, 1998)*, and in women in post-menopause *(Fromm et al., Osteoporosis Int 1991, 1, 129-133 ; Papapoulos et al., Bone, 1992,13, 41-49 ; Peretz et al., Maturitas, 1996, 25, 69-75).* The use of new biphosphonates by oral route like Alendronate is not possible ; although its strong efficacy is well-established and it is clinically approved for osteoporosis, its adverse effect to make esophageal erosions specially in supine position do not allow to use it during a bed rest study.

Therefore, it was proposed to use one single infusion of pamidronate 14 days before the start of the bed rest period to obtain sufficient effects during bed rest. This infusion will be performed slowly (in 3 hours) to avoid the side effects like hyperthermia.

### Harmonisation of physiological and medical measurements to evaluate the effects of countermeasures.

There is presently, no international agreement on a common procedure to develop, evaluate and validate countermeasures, first on ground then in flight. In co-operation with the responsibles of medical operations, NASA has initiated this procedure by proposing a first battery of tests designed to evaluate accurately the effects of countermeasures (ITR for Integrated Test Regimen). Furthermore, astronauts could have to perform several times identical tests. Even though in some cases these tests could be complementary, in some cases, this situation id due to the lack of standardisation of the measurements.

During this study, the space agencies propose a first implementation of a battery of tests, adapted to scientific protocols and as close as possible to the battery proposed by NASA. (ITR). The longer term objective is to build up a data base enabling a standardised evaluation and validation of countermeasures.

Most of the tests proposed within the frame of ITR are already requested by the scientific investigators in their protocol (ex : DEXA measurements to assess bone density, Cybex to assess muscle strength, blood and urine dosages to evaluate the nutritional status, exercise capacity,…). Some tests, not required by the scientists, have been added to complete the ITR program. These tests are described in the corresponding paragraph.

## Medes background

MEDES has developed since more than ten years an outstanding experience in the performance of bed rest simulation experiments, in particular for long term studies. Before MEDES creation, a one month bed rest was performed in 1988-1989 in Toulouse hospital, with the participation of future MEDES staff. A second bed rest one month bed rest has been organised by MEDES in 1990-19991. Both studies were funded by CNES. In 1994, MEDES has performed a 42 days bed rest study funded by ESA and CNES, in cooperation with a dozen European scientific teams.

Since 1996, MEDES is operating a facility dedicated for these experiments, the “Space Clinic”. On an average, MEDES has organised in the “Space Clinic” two clinical studies per year, using the centrifugation or the bed rest model. Two bed rest studies have been performed within the frame of a collaboration between CNES and DLR and in 1998, MEDES has performed a bed rest study on females. For all these studies, the scientific protocols have been proposed and performed by external scientific teams, MEDES being in charge of the overall co-ordination and organisation of the study.

# STUDY GENERAL DESCRIPTION

## Operational scenario

The volunteers will be hospitalised during a 4-month period :

- 15 days for ambulatory control period (BDC: Baseline data collection),
- 90 days of (-6°) HDBR period with or without countermeasure (Exercise or biphosphonates),
- 15 days for recovery after HDBR.

Furthermore each subject will have to come back at MEDES for other follow-up periods (45, 90, 180 and 360 days after the end of the HDBR period).

Finally, each volunteer will stay 127 days within MEDES clinical research infrastructure. Each period will be performed according to the overall experimental protocol which is presented as Annex 1. Briefly:

BDC-16 - Arrival at MEDES

- Inclusion parameters confirmation

BDC-15/BDC-1 - Ambulatory period for baseline data collection

HDT1/HDT90 - (-6°) HDBR period, with three groups of volunteers:

Controls

Exercise

Biphosphonates

R+1/R+15 - Tilt Test (first standing)

- Recovery period

R+16 (morning) - Medical check-up

- Volunteer departure from MEDES

R+45, +90, +180, +360: Recovery data collection

Two volunteers will be included in the study each day. These two volunteers will follow the same experimental schedule, with the necessary delay imposed by equipment and investigators disponibility.

In total, twenty eight volunteers will participate in this study with two different campaigns of (14 per campaign).

## Integrated Test Regimen (ITR). Adaptation of ITR program to the bed rest study

As mentioned in background information, besides the scientific objectives, the aim of this experiment is also to use this bed rest model to evaluate and validate the countermeasures before their use in-flight and therefore to assess their effects by using a standardised battery of tests.

NASA has already proposed a battery of tests called ITR (Integrated Test Regimen) which includes some tests already performed within the frame of the medical follow-on of the astronauts and additional tests proposed to assess more accurately the effects of counter-measures. Nevertheless, this battery was never applied to bed rest experiments.

Within the frame of this prolonged bed-rest experiment presented in this document, the organizing agencies, ESA, NASDA et CNES requested MEDES to include the integration of some of these ITR on the experiment schedule. The list of these ITR tests as well as their content were selected on the base of proposals of NASA, NASDA and ESA, the selecting criteria being their pertinence with the long duration bed-rest context and their compatibility with the research protocols submitted by the different scientific teams participating in the experimentation. **Most of these tests are not specific to the ITR, but are common with several scientific protocols.**

During this experiment, some of the ITR tests will be applied in order to standardise the evaluation of the effects of prolonged head-down bed rest and the effects of the countermeasures (muscular exercise and biphosphonates). Nevertheless, in this experiment these tests are adapted according to the scientific protocols already selected. Some of the ITR tests also requested by the investigators for scientific objectives are planned here in order to avoid their duplication for ITR and scientific purposes. Some of the ITR tests are also usually performed within the frame of the medical follow up during bed rest studies.

The following table summarises the tests needed for this ITR program including :

- the name and the category of the tests,
- the timing proposed within the NASA Countermeasure Evaluation and Validation Program (CEVP) or described in the Astronaut Medical Evaluation Requirement Document which is the actual official document summarising the medical tests planned for the astronauts on the International Space Station including the comments of the agencies sponsoring this bed rest study,
- the timing of the ITR program during this bed rest study taking into account the measurements needed for the already selected scientific protocols.
- the description of the measurements to be performed during the bed rest study.

These tests will be performed on the three groups of subjects (controls, biphosphonates, muscular exercise).

**Only the tests requested specifically for ITR program will be fully described hereafter.** The other scientific or medical tests (of which the data will be used to set up an ITR database) will be only listed hereafter. Their complete description can be found in the corresponding paragraphs (scientific protocols or medical follow-up).

### Daily physical examination, anthropometry and metabolic assessment

Some parameters measured daily during the bed rest study and described in the daily medical follow-up can be included in the ITR program, like arterial blood pressure, heart rate and weight. Weekly measurements are proposed to be included in the ITR database.

Measurement of height, and assessment of body composition by impedancemetry proposed in Dr Oshima protocol with regular measurements during the first two weeks and then monthly during the bed rest are also proposed in the ITR program to assess the nutritional status.

Measurement of the daily fluid balance (fluid intake minus 24 hour urine output) as well as the daily caloric intake will be used in the ITR database as a part of the nutritional status assessment.

### Laboratory assessment

Blood samples and 24H urine pools will be regularly collected before bed rest (BDC-14), during bed rest (HDT 14, 30 ,60 ,90 ) and after bed rest. These assays will contribute to the assessment of the nutritional status. The nutritional assessment is required to determine adequacy of nutrient stores before the bed rest, to assess nutrient status during and after the bed rest. These assays will also contribute to the medical follow-up of the volunteers during the bed rest.

They include the following dosages :

- in plasma : complete blood cell count, Hte, hemoglobin, glucose, total proteins, albumin, creatinine, electrolytes (Na, Cl, K, Ca, P, Mg), cholesterol, triglycerides, transaminases (AST, ALT)
- in urines : Na, K, Ca, urea, creatinine

Some bone markers (assayed in plasma and urine) as well as DEXA measurements (described hereafter) requested also for scientific protocols (bone specialists) will also contribute to assess the nutritional status by determining changes in bone.

A more complete biological nutritional check-up will be performed on BDC-14, HDT 90 and R+14 including the following parameters :

- serum protein electrophoresis
- transferrin, tranferrin receptors, ferritin ,
- urinary methyl histidine,
- urinary phosphorus and magnesium (already requested in scientific protocols)
- ceruleoplasmin, transthyretin, retinol binding protein

In addition various blood and urinary chemistry and endocrinology parameters susceptible to be modified by real or simulated weightlessness will be assayed before (BDC-14), at the end of the bed rest (HDT90) and before departure (R+14). These measures are those fixed at the chapter covering the nutritional status and several others in addition:

- reticulocyte count, osmolality, complete lipid profile, LDH, CK, reactive protein C,

- insulin, growth hormone, cortisol, thyroid hormones (T3,T4,TSH).

The risks linked to this assessment are those linked to the blood samples described in the general risks of the experiment.

### Physical fitness assessment

#### Maximum heart rate test on a cyclo-ergometer.

The maximal oxygen consumption (or VO2 Max.), the ECG and the arterial blood pressure will be measured before and after the bed-rest, on a cyclo-ergometer based on a protocol of gradual increase of the maximal workload. The protocol used to assess the maximal oxygen consumption will be the protocol described in Dr Ferreti experiment.

The nominal test consist in a warm-up pedalling session of 5 minutes without load. Then the resistance will be brought up to 50 Watts during 5 minutes, 100 Watts during 5 minutes, 150 Watts during 5 minutes, etc..

Then, when the subject is felt to approach VO2 Max., the imposed resistance will be increased of 25 Watts every 5 minutes, and this until the subject reaches a major fatigue level, or the test is stopped by the physician. During this test the pedalling cadence will be optimally kept at 75 cycles per minute.

Between each imposed resistance threshold, the subject shall maintain a 5 minutes rest period, during which the lactates will be measured. These will be made at the auricular or digital level, at the first, third and fifth minute of this pause.

The individual V02 Max will then be determined by the measurement of a «threshold» reached with an oxygen consumption (which does not increase anymore) and the power level achieved

INSERER TABLEAU ITR 1

INSERER TABLEAU ITR 2

for a given resistance. If this threshold cannot be observed, reference will be made to other measurement criteria: 1°) the absence of any heart rate increase between two successive work levels, 2°) a ratio of the respiratory gas in excess of 1.1, 3°) lactates quantities measured at more than 10 mM.l ^–1^.

The test may be modified so as to be best adapted to the mass and physical fitness of the volunteer. More attention will be given to the tolerance displayed during this exercise after the bed-rest.

This test will be performed before bed rest (BDC-15) and after bed rest (BDC+4)

#### Sub-maximal heart rate test on cyclo-ergometer.

The maximal heart rate tests performed on cyclo-ergometer before and after bed-rest are completed by sub-maximal heart rate tests before, during and after the bed-rest period as proposed during space flights. These tests will be performed in sitting upright position before and after the bed rest and in supine position during the bed rest. The workload applied to the volunteer will be determined as a function of the VO2 Max. measured before bed-rest (previously described). The workload (measured in Watt) applied will be that the subject developed when at 25%, 50%, and 75 % of his VO2 Max. The tolerance to effort will be evaluated indirectly by measuring his heart rate. At the same time, the arterial blood pressure, the imposed resistance level and the effort perception, will be recorded. No measurement of the respiratory gases will be made during these tests.

The selected protocol is:

0-2 minutes : rest

2-7 minutes: pedalling at a resistance level corresponding to 25% of VO2 Max.

7-12 minutes: pedalling at a resistance level corresponding to 50% of VO2 Max.

12-17 minutes: pedalling at a resistance level corresponding to 75% of VO2 Max.

7-22 minutes: progressive and quiet stoppage of the imposed resistance ( 25-50 W )

The purpose of this protocol is to evaluate the subject’s tolerance to exercise during this experiment with a protocol similar than in flight The tests will be performed at BDC-10, HDT13, 31, 61, 86 and R+12.

*Medical risks*

The risks of intensive muscular exercise are described in Dr Ferreti experiment (VO2 max measurement). The same kind of risks exist, even if less, with the sub-maximal heart rate test.

If these risks (in particular cardio-vascular complications : infarct, rhythm disorders) have to be mentioned, a complete medical selection examination of the volunteers is the best way to reduce their probability of onset. Strict security measures will be taken: precise stop criteria during the exercise performance are determined. The electrocardiogram and/or the arterial pressure will be continuously monitored. The stop criteria will be the following :

1. sudden fall of the heart rate of more than 15 beats per minute,
2. sudden and persistent fall of the arterial pressure (25 mm Hg for the systolic, 15 mm Hg for the diastolic),
3. significant arrhythmia
   1. one or more brady-arrhythmias
   2. one or more tachy-arrhythmias
   3. evident sign of heart blockage other than the first degree one
   4. premature ventricular contractions (PVC) which meet any of the following criteria :

six or more PVC’s per minute,

PVC’s that are closely coupled,

PVC’s that fall on the T wave of the preceding beat,

PVC’s that occur in couplets or runs,

PVC’s that are multi-formed,

1. Systolic arterial pressure < 80 mm Hg,
2. Evidence of myocardial ischemia on the ECG
3. Severe nausea, moist skin, sweat, light headed , dizziness or tingling sensation,
4. Pre-syncope symptoms, vaso-vagal fainting,
5. Patient’s request.

Trained personnel with resuscitation equipment will be always present where the tests are performed.

When measuring the data for the expiratory gases, bacterial, viral or fungic infections will be prevented prevent by using one individual sterile mouthpiece for each subject.

#### Test with the isokinetic dynamometer.

The performance of certain muscular groups will be also assessed before and after bed-rest. The maximal and sub-maximal muscular contraction will be determined. For this, a Cybex.6000 dynamometer will be used. The measured parameters are: the maximum contraction strength, the maximal force torque, the angular velocity, the time necessary to reach maximal explosive speed and the clinical endurance time (one articulation is tested at different speeds)

*Protocol :*

All the tests will be carried out in concentric mode by means of a Cybex 6000. The measures are taken in a sitting (pre/post bed rest) or in supine position (bed rest):

1. ankle(s): plantar flexion and extension with an isokinetic and self regulated angular speed of 30, 60 degrees/sec .4 repetitions.
2. knee(s) : flexion and extension at 30, 90 , 180 degrees/sec .4 repetitions
3. concentric extension endurance of the knee at 180 degrees/sec 30 repetitions

One test will be performed only before and after the bed-rest in standing position: evaluation of the trunk muscles based on a 60° flexion/extension with 4 repetitions.

*Risks :*

There are two kinds of associated risks:

-locally, where muscles accidents may occur during the muscular performance tests, from elongation to laceration.

-general, with the cardiovascular risks generated by the performance of important muscular efforts. These risks are related to those previously described during the maximal and sub-maximal heart rate tests.

### Operational tilt test

*Objectives*

Tilt tests are used pre- and post-bed rest to assess the effects of "simulated weightlessness" conditions on orthostatic tolerance during up right posture. Moreover, this test will be allowed to evaluate counter-measures against the effects of orthostatic hypotension post bed rest.

*Measurements parameters*

The volunteer is instrumented and placed on the tilt table.

The test consists of supine position and 80° upright tilt (10 min.) data collection for the following variables: Aortic Doppler ultrasound for determination of cardiac output and total peripheral resistance, an ECG and heart-rate, beat-to-beat non-invasive blood pressure (Finapres) and minute-to-minute automatic blood pressure measurements.

The test is carried out as follows: supine position at 0° during 30 minutes, period during which the necessary sensors are installed and during which the reference measurements are performed, +80° head up tilt position (reached in approximately 10 sec) during 10 minutes. In case of intolerance, the volunteer is brought back to the lying position, the test of vertical position is then planned more progressively.

A short questionnaire is then completed by the volunteer at the end of the test.

The tilt test will be performed before bed rest (BDC-15) and after bed rest (R+1). After bed rest, the subjects will stand up for the first time during the tilt test.

*Risks*

The medical risks will exist in particular at the end of bed rest when the volunteers will stand for the first time after the prolonged bed rest, mainly:

- orthostatic hypotension inducing pre-syncope or syncope symptoms
- vaso-vagal fainting
- excessive tachycardia
- cardiac rhythm disorders

In order to limit medical consequences, test termination criteria have been determined. If any of following instances occur while the volunteer is standing, he is laid down and monitoring continued until the subject is stable:

1. A sudden drop in heart rate than 15 beats per minute
2. A sudden drop in blood pressure (systolic fall >25 mm Hg or diastolic fall >15 mm Hg)
3. significant cardiac arythmias :
   1. bradyarhythmias ( example : heart rate < 40 for people whose resting heart rate is > 50)
   2. tachy arhymthmias (3 or more beats in a row of supraventicular or ventricular tachycardia
   3. Evidence of heart block other than first degree
   4. PVC’s which meet any of the following criteria :
      1. six or more PVC’s per minute
      2. PVC’s that are closely coupled
      3. PVC’s that fall on the T wave of the preceding beat
      4. PVC’s that occur in couplets or runs
      5. PVC’s that are multi-formed
4. An absolute systolic blood pressure < 80 mm Hg,
5. Severe, nausea, clammy skin, perfuse sweating, pallor, light-headedness, dizziness,
6. Volunteer request at any time

### Cardiovascular tests

An increased incidence of cardiac rhythm disorders has been reported during space-flights.

Besides regular ECG, ambulatory (24 H) Holter monitor will be performed before and during the bed rest ( BDC -13, -3, HDT 15, 32, 62, 85).

Echocardiography and Doppler measurements of different arteries (upper and lower limbs, mean cerebral artery) and veins (upper and lower limbs) will be realised for scientific purpose (Dr Louisy experiment). Some of these measurements could be included in the ITR program after analysis if evidence of their interest in the evaluation of countermeasures effects (in particular assessment of the changes in vascular resistance).

### Bone tests

*Objectives*

Bone densitometry (DEXA) is used pre- and post- flight to track individual skeletal integrity (loss and recovery), and is a specific requirement of nutritional assessment.

*Measurement parameters*

The bone densitometry (dual energy X-ray absorptiometry) is evaluated on proximal femur (hip), lumbar spine L1-L4 (lower back), calcaneus (heel) and whole body (including skull). Percent fat and lean body mass will also be measured.

*Risks*

The examination is totally painless and non invasive, and delivers an x-ray exposure 10 times less than that observed for a lung radiography. A total of 12 DEXAs will be carried out during the whole experiment. The total of X-ray exposure is 235 μSievert (25 μSievert *12).

### Functional neurological assessment

The purpose of this activity is to perform functional neurological assessment that provide an objective test of the volunteer’s neurosensory re-adaptation to standing position following prolonged simulated weightlessness. This balance test is performed pre- and post bed rest.

The equipment needed to perform the Equitest is not available in Toulouse. The balance analysis will be performed before and after bed rest according to the following protocol.

Balance function is tested both in static and dynamic conditions.

1. Static balance (orthostatic position on a fixed base) will be analysed by a force platform SATEL® (France). Three strain gauges allow the measurement of the displacements of the subject’s foot pressure center on the oxy horizontal plane during a period of 51.2 seconds (sampling frequency: 40 Hz). Several parameters measure postural stability eyes open and closed. The length of the statokinesigram (LS) is total linear displacement of the foot pressure centre on the oxy plane, the area of the statokinesigram (AS) contains 90% of the successive positions of the foot pressure centre. The AS allows a better estimation of the static stability than LS parameter. The mean x position (Xm) indicates the mean projection of the gravity centre according to the x axis (lateral sway). The mean y position (Ym) indicates the mean projection of the gravity centre according to the y axis (anteroposterior sway). The length versus area (LVA) is the ratio : measured length/calculated length from the area ; the ratio value must be near of the units, its variations give an indirect measure of the whole subject energy expenditure during the test. The variance versus y (VVY) is the ratio : measured variance/calculated variance from the length of the y displacements. The VVY variations afford an indirect evaluation of the muscular tone of the posterior muscles group of the legs.
2. Dynamic balance (upright position on a rocking base) : The subjects are examined while standing on a seesay platform derived from the platforms of Freeman et al. and Dietz et al.; the seesaw platform is placed onto the force platform. The seesaw, consisting of a platform with a cylindrical curved base (weight : 2 Kg ; height with the platform horizontal : 6 cm; radius of base curvature : 55 cm), is in contact with the force platform horizontal : 6 cm; radius of base curvature : 55 cm), is in contact with the force platform along a generatrix of the cylinder (pivot). Angular tilting of the seesaw is monitored by the strain gauges of the force platform. The seesaw platform make the subject unable to stand still. From the signal output, a microcomputer calculates the dynamic balance parameter i.e. the sum in milli-meters, during 25.6 sec., of the absolute values of the elementary horizontal displacement (stabilogram) of the seesaw pivot on the ground (length of the pivot displacement = LPD) ; this parameter indicated the skill in dynamic balance. Then a Fast Fourier Transform (FFT) processing allows a spectral analysis of the stabilograms and give the total energy (TE, cm^2^) and the energy distribution into 3 frequency bands (0-0.5 Hz : Iow-frequency energy (LFE), 0.5-2 Hz : medium frequency energy (MFE), 220 Hz: high frequency energy (HFE)) ; this energy is expressed in cm2 and in percentage of TE. These last parameters inform about the balance strategy implemented. According to the position of the subjects on the platform, anteroposterior or lateral sways were successively recorded eyes open then eyes closed ; during the tests the subject was instructed to maintain the seesaw as horizontal as possible.

In addition gait will be also analysed:

1. Gait analysis : A kymographic method allows the measurement of the simultaneous horizontal displacements of both feet along a 6 m walkway. Recordings of longitudinal displacement of both feet are performed by linking each foot to a length-voltage transducer (potentiometer) by means of threads. The subject wears disposable plastic slippers fitted over the bare foot with straps. The slippers are connected to the transducer by a thread fastened to the head of the second metatarsal. The length of the displacements of each foot versus time is measured by the potentiometer output. The spatial and temporal gait parameters of the right and the left foot are processed by a microcomputer from several successive walk cycles. The parameters measured are the stride and the step length (m) as well as the cycle, the stance, the swing, and the double support durations (s). From these lengths and durations several parameters are calculated: the total cadence (strides.min^-1^) and the walking speed (m.min^-1^). The subject is instructed to start on the preferred foot first and reach the end of the 6 m walkway with sustained walk cadence.

*General protocol:*

The experimental procedure began by gait analysis (total duration about 5 min) and continued with balance analysis (total duration about 10 min.). Each subject underwent 2 practice sessions during the ambulatory period to become familiar with the procedure of gait and balance examinations. The subjects will be tested before the bed rest during the basal data collection period and during the recovery period.

*Risks:*

These techniques are fully without trauma potential. The only risk is that of falling from the platform (Each has a height of 6 cm.). During the experience, 2 persons are permanently within reach on each side to prevent any fall particularly in the recovery period.

Other tests like NMR measurements of lower limbs and lumbar muscles are not yet included in the ITR program but could also be interesting to be included in a database to assess the effects of the countermeasures.

## Selection and follow-up of volunteers

### Subject's recruiting and medical selection

#### Recruiting mode

Call for candidates will be made via Internet on the MEDES and ESA sites and by press announcement.

A minimum of 70 subjects will be pre-selected on the base of their files, in order to finally select 34 subjects (28 + 6 back-up) for the two research programs (14 +3 subjects in 2001, 14 +3 subjects in 2002).

#### Selection organization

The selection will be carried out in two phases:

- One preliminary selection on the base of the application files, comprising first a questionnaire on the subject’s way of life, his education and professional experience; and second a medical questionnaire on the personal and family medical history.
- A selection session comprising clinical, paramedical and psychological examinations, carried out where the investigation will be undertaken (Space Clinic).

***Pre-selection based on the application file***

*Number of subjects*

The purpose of this pre-selection is to select, on the base of their application file, 70 healthy volunteers who meet the requirements of the criteria listed below:

The pre-selected subjects will receive an information and consent document, so that they be fully informed and in a position to make their decision to participate or not in the experiment (after having carefully read the information document).

*Acceptance criteria*

- Healthy male volunteer , citizen of the European Community.
- Age 25 to 45,
- Non smokers,
- No alcohol, no drug dependence and no medical treatment,
- Height 165 cm to 185 cm,
- No overweight nor excessive thinness. BMI (weight Kg/ height m^2^) between 20 and 27,
- No personal nor family past record of chronic or acute disease which could affect the physiological data and/or create a risk for the subject during the experiment,
- Subject to be covered by a Social Security system,
- Free of any engagement during four consecutive months.

*Specific exclusion criteria*

- Having given blood (more than 300ml) in a period of three months or less before the start of the experiment,
- Subject already participating in a clinical research experimentation,
- Poor tolerance to blood sampling,
- Past record of orthostatic intolerance,
- Cardiac rhythm disorders,
- Allergies,
- Intensive sport training,
- Fractures or tendon laceration since less than one year,
- Chronic back pains,
- Past records of thrombophlebitis,
- Presence of metallic implants,
- Special food diet,
- Sleep disorders :
  - Lark and owl type,
  - Subject sleeping more than 10 hours or less than 5 hours,
- Photosensitive epilepsy.

#### Selection description

The purpose of these medical and psychological examinations is to select the subjects most apt to participate in a long duration bed-rest campaign. They will be carried out by physicians and psychologists, on the research location (Space Clinic), as well as in the specialized departments of the Rangueil Hospital in Toulouse.

These tests will last 48 hours.

- Research location : Space Clinic*,* CHU Rangueil, Avenue du Pr. Poulhès, 31403, Toulouse cedex4.
- Biomedical research agreements : Ministerial Agreement for undertaking biomedical research without direct profit concerning: the physiology, the ergonomy and the physio-pathology of healthy men in weightlessness (14027 S) ; the use of medicaments in the domain of space medicine on adults (N°14027 M).

The purpose of the first step is to select 17 subjects (14 subjects + 3 replacements) for the first experimentation campaign in 2001. The second selection process will start at the end of the first period.

##### Medical tests description

The medical check-up will include:

*On the research location (Space Clinic):*

- clinical examination,
- questionnaire to verify that no exclusion criteria is present,
- 12 leads electrocardiogram,
- measurement of the arterial blood pressure and heart rate first in a lying position, then in a standing position after 10 minutes (Stand Test), and a test on a tilt table (+80°) during 15 minutes (Tilt Test). These tests will check the absence of orthostatic hypotension.
- questionnaire on the Horne and Ostberg “matidinality–vesperality” index to evaluate the quality of the subjects’ sleep.
- ophthalmologic examination: visual acuity, and fundus examination
- biochemical, hematological, serological analysis et and search for toxic substances,
- Blood biochemical analysis:
  Urea, glucose, electrolytes (Na^+^, K^+^, CI^-^, P, HCO3^-^, Ca^++^), proteins, cholesterol, triglycerids, creatinine, total and free bilirubin, uric acid, transaminases (SGOT and SGPT), γGT, alkaline phosphatases.
  These assays will be carried out at the Biochemical Laboratory of the Rangueil Hospital.
- Hematological analysis:
  Globular count, platelet count, leukocyte formula, hemoglobin, haematocrit, VS, TP, TCA, fibrinogen and phlebitis markers (anti thrombin III, S-protein, C-protein, resistance to activated C-protein)
  These assays will be carried out at the Hematology Laboratory of the Rangueil Hospital.
- Thyroid hormone analysis:
  TSH, T3, T4.assays
- Serological analysis:
  Search for the hepatitis markers (B,C) and serological HIV (Volunteers are informed before the test).
  These assays will be carried out by the Virology Laboratory of the Rangueil Hospital.
- Urine analyses:
  made on strips: haematuria, leucocyturia, glucose, ketonuria, proteinuria, bilirubine, urobilonogenesis, nitrites, pH, density.
- Search for toxic components:
  The following components will be searched in the urine: barbiturics, benzodiazepins, opium derivatives, cocaine, amphetamines and cannabis.
  These assays will be carried out at the Laboratory for Pharmaco-Kinetics and Toxicology of the Rangueil Hospital.

*In the Specialized Departments (Rangueil Hospital, Toulouse):*

- Echo-doppler measurements of the lower limbs in order to eliminate the venous deficiencies,
- Chest radiography front and side,
- A dental panoramic radiography,
- Abdomen radiography to check the absence of lithiasis,
- Abdomen echography,
- DEXA measurement of the bone density which should be no more than 1 standard deviation above or below the age/sex-matched mean,
- A measurement of maximal oxygen consumption (VO2 max.)
- In doubt on a given pathology, the investigator may request additional investigations.

##### Psychiatric and psychological evaluation

The purpose of these examinations are twofold: the first one is to sort out the subjects to detect persons suffering from psychopathological tendencies (select-out). The second one is to detect the candidates who have the best profile to participate in the experiment (select-in).

The selection protocol includes psychological tests applied to the candidates in a group, a projective test, and an interview. The tests were selected because of their capacities to reveal the psychological characteristics required for a good resistance to social desirability. Also among the tests’ selection criteria is the existence of a certified version in both French and English.

*Tests passed when in group* :

- Gordon Personality Inventory (GPPI, Gordon, 1982)
- Defense Mechanisms Inventory (DMI, Gleser and Ihilevitch, 1969)
- Eysenck Personality Inventory (EPI, 1971)
- Bortner Stress Comportment Adaptation Questionnaire (1969)
- Spielberger Inventory of the Anxiety State-Trait (Spielberger et al. 1970)
- Rathus Assertivity Scale (Rathus, 1973)
- Inter-personal Values Inventory (SIV, Gordon, 1975)
- Personal Values Inventory (SPV, Gordon, 1967)

*Projective Test* : Wagner Hand Test

*Individual structured interview*

The time required per subject for these tests is approximately 2h for the group session, 15 minutes for the projective test and 30 minutes for the interview.

##### Subject Aptitude

Candidates will be declared apt to participate in the experiment at the following conditions:

- After verification that the information document has been carefully read, candidates will have the possibility to ask any additional question concerning the research project. After having obtained satisfactory answers to these questions, they will have to sign an Information and Consent Form.
- After checking the results of the tests, verification will be made that all inclusion criteria are present and that there are no non-inclusion criteria.

##### Reasons of exclusion during the experiment

**Participants are free to withdraw from the program if they so wish, at any time and for whatever reasons. An exclusion will be effective after the joint agreement of the coordinating investigator and the promoter’s representative, in accordance with the rules described in the participant’s Information and Consent Form.**

- 1. Non compliance with the protocol, refusal to co-operate.
  2. Undesirable effects, occurrence of a pathology limiting the experimentation,
  3. Impossibility to measure the data as requested by the protocol.
  4. If excluded during the experiment, the volunteer shall receive a indemnity “pro rata temporis”, according to the indemnity plan described in the informed consent form. Excluded participants will benefit from the same medical monitoring as the others.

### Psychological Preparation

The importance of preparing the subjects has often been demonstrated. The proposed work concerns all the subjects as well as the accompanying personnel and possibly the investigators.

The preparation phase should be undertaken before the experiment’s start, and preferably at least 5 days before ( needed by the subjects to fully integrate the message they receive).

**Psycho-physiological and somatic preparation**

Learning the relaxation and breathing techniques. The instruction lasts approximately 1h30. the subjects will be able to use these techniques during the bed-rest,

**Comportment and cognitive preparation:**

- Preparation session to bed-rest  (approximately 1h30)
- Information on the reactions to isolation.
- Learning the communication and expression techniques to permit an early expression of the difficulties before these become too important, and how to communicate without aggressing the others,
- Planning a time schedule and occupations. Each participant should find himself a personal goal to fulfill during the bed-rest period: learn a foreign language, write a document, develop a computer program, etc…
- How to best organize one’s time, integrating the personal activities within the experimental constraints: meals, sample taking, etc…
- Management of one’s personal objectives during the experiment.

**Presentation and explanations concerning the tools which will be used during the bed-rest : 30 minutes**

All the above represent approximately 5 working hours for the preparation of the subjects.

### Medical Follow-up during the Experiment

The medical follow-up includes:

- The verification of inclusion criteria the day of arrival in the Space clinic: On the day of arrival, an interview and a clinical examination will be performed in order to check the permanence of the inclusion criteria (absence of disease and drug intake able to interfere with the inclusion criteria since the selection process)
- Daily medical supervision during the whole experiment,
- Specific medical supervision tests and monitoring of functional tests, like orthostatic and exercise stress tests,
- Medical check-up at the end of the experiment.

#### Daily medical supervision

The daily medical supervision includes the following measurements:

- The arterial blood pressure (systolic, mean and diastolic) and the heart rate will be measured by means of an automated sphygmomanometer (Dinamap^®^), daily (6h30 am) and (6h30 pm),
- The weight will be measured with a scale designed to weigh persons in bed rest position. The weight will be measured early the morning, after emptying the bladder, before breakfast,
- The body temperature will be measured with a tympanic thermometer, morning and evening,
- The hydric intake and the 24H diuresis (hydric balance) will be measured daily.

#### Specific medical supervision

- Because of the potential risk of thrombo-phlebitis, besides the a accurate questionnaire and a clinical examination, a prevention by physiotherapy will be applied. No pharmacological prevention will be used because of the potential risks of low molecular weight heparin and its possible interference with bone changes. Echo-Doppler measurements of lower limbs veins will be performed each month, and in any case of clinical suspicion.
- To prevent or relieve back pains, physiotherapeutic care will be applied, only after the 9th day of the bed rest to avoid interference with protocols studying back pain. Before this date, massages of back muscles will be performed only in case judged necessary
- To prevent the risk of renal stone, sufficient liquid will be taken (30 ml/Kg/day).
- Abdominal X-ray will be performed before bed rest and controlled before departure to check the absence of calcium lithiasis.
- Blood samples will be collected before bed rest (-14), during bed rest (day 7,14, 30, 60, 90), after bed rest (+7, +14, +45, +90, +180, +360). The parameters measured will be:
  - - glucose,
    - proteins,
    - chloride,
    - phosphore,
    - creatinin,
    - Na, K , Ca
    - cholesterol,
    - triglycerids,
    - alanine amino-transferase, aspartate amino-transferase,
    - Complete blood cells count, reticulocyte, Hb, Ht,
    - TP (prothrombin rate), TCA, Fibrinogen
- Monthly urine pellet will be collected,
- ECG - 12 lead will be performed before bed rest and every 30 days during the bed rest
- 24 H Holter ECG will be performed before bed rest and during the bed rest at D15 and every 30 days
- to prevent the risk of fracture, bone mineral density will be followed and analysed during the experiment. The volunteer will be excluded from this experiment if changes in bone mineral density are large enough to induce a fracture risk before the end of the study.

#### Monitoring of functional tests

Blood pressure, heart rate, SaO_2_ and clinical tolerance will be monitored during the following tests :

- Orthostatic tests (stand and tilt tests),
- VO2 max measurement, sub-maximal heart rate test,
- Baroreflex and cardiac performance during exercise, gas exchange kinetics, cardiac output and oxygen transport,
- Equilibrium assessment during the recovery period,
- Muscular biopsies.

Stop criteria are defined for each test and will be applied by an physician from Medes present with the necessary resuscitation equipment.

At the end of the bed-rest, the volunteers will stand for the first time during the operational head up tilt test proposed within the frame of the ITR program. The head up tilt test is a reference test to assess orthostatic tolerance.

The test will be carried out as follows:

- lying position at 0° during 30 minutes, period during which the necessary sensors will be installed.
- +80° head up tilt position (reached in approximately 10 sec) during 10 minutes. In case of intolerance, the volunteer is brought back to the lying position, the test of vertical position is then planned more progressively.

After this test the subject is allowed to be in sitting and then in standing position, each step duration being dictated by the observed volunteer’s tolerance and monitoring of blood pressure and heart rate.

During this test, the ECG is permanently monitored as well as the finger arterial blood pressure (photoplethysmographic method, Finapres). The blood pressure and the heart rate are also continuously measured every minute on the brachial artery (Dinamap).

The tolerance to the change of position and to the maintaining in standing position is assessed by clinical and cardiovascular parameters.

The stop criteria will be the following ones :

1. syncope,
2. clinical signs of orthostatic intolerance (pallor, sweating, feeling of fainting, dizziness...),
3. quick and persistent decrease in systolic blood pressure higher or equal to 25 mm Hg,
4. brutal variation in heart rate of at least 15 beats/min minute after minute in stable period (i.e. excluding phases of changes of position),
5. important tachycardia (> 160 beats/min), significant cardiac rhythm disorders.

At the end of the bed rest this tilt test will be done in the presence of a resuscitation physician, of one physician and a nurse of the clinic’s staff with all the necessary resuscitation equipment.

All the orthostatic exercise stress tests planned in the early recovery period (3 first days) will be performed in the presence of a resuscitation physician.

#### Medical check up at the end of the experiment

On the day of departure, each volunteer will undergo a clinical examination including measurements of blood pressure and heart rate in supine position and after two, and five minutes in upright position to check orthostatic tolerance (stand test).

The same biological tests (blood biochemistry and haematology) than those performed during the selection will be performed. Plasma samples will be also stored to perform virology tests in case of any problem after the end of the experiment.

Before departure recommendations for physical training will be advised to the volunteer. An information will be given to the attending physician of the volunteer.

### Physiotherapy during the experiment

Every day, subjects will be massaged either by physiotherapists or by students of the Physiotherapy School. The aim of these massages is to prevent the muscular pain and the occurrence of thrombo-phlebitis. These massages which last 30 minutes will be planned in order to avoid interference with the scientific protocols.

They will be performed on the back (during 10 minutes) to relieve any muscular pain, and on the lower limbs to improve the venous back-flow (10 minutes for each limb). Daily passive mobilisation of joints will be also performed daily. As previously mentioned , massage of back muscles will be started after the 9^th^ day of bed rest.

Diaphragmatic breathing sessions will be performed every day, the objective of which is to improve venous back-flow. After adequate training, the volunteers will be able to practice several times in the day.

Every day, the subjects will be recommended to perform ankle circumduction exercises.

### Clinical follow-up after experiment completion

After completion of the experiment, a surveillance will be maintained, either at the clinic (4 times), or by telephone (2 times).

The volunteers will be invited to come back to the clinic for an interview, a clinical examination and some medical and scientific investigations whose data will permit to assess the recovery of the volunteers:

- R+45 (30 days after departure): Questionnaire, clinical examination including blood pressure and heart rate measurements in supine and standing positions, weight and height measurements, bone density and fat/lean body mass assessed by DEXA, body composition assessed by impedancemetry, muscular strength (Cybex), blood samples (previously listed), sub-maximal heart rate test,
- R+ 60 (approximately 2 months after the end of the bed rest period): questionnaire and telephone call,
- R+ 90 (approximately 3 months after the end of the bed rest period): the same tests as planned for R+45 will be performed. Others scientific measurements like heel Radiography, MRI of lower limbs (to assess recovery of muscular atrophy) will contribute to evaluate the recovery of the subjects.
- R+180 (approximately 6 months after the end of the bed rest period): same tests as R+90.
- R+360 (approximately 12 months after the end of the bed rest period): Questionnaire and clinical examination, DEXA measurements will permit to assess bone density recovery and MRI and Cybex to assess muscular changes recovery.
- R+720 (approximately 2 years after the end of the bed rest period): questionnaire and telephone call.

### Psychological surveillance during the experiment

The purpose of the tests proposed within the frame of surveillance is to detect as early as possible any possible difficulty, in order to find a prompt solution and so avoid to important a deterioration of the subject’s mood.

**Protocol for the subjects**

- Twice per week (every 3 days) (duration: approximately 15 minutes)
  - Rating the anxiety level (Scale Y1 of the State Trait Anxiety Inventory of Spielberger, 1970)
  - Adaptation questionnaire (AQ, Cazes et al., 1989)
  - Global Health Questionnaire (QSG)
- Subjects will be requested to write a diary. The reason for this is let volunteers cast as objective an eye as possible on the experience they are submitted to by means of a daily report.
- Systematically every 15 days, interview with a psychologist.
- Once a week, or every 15 days (approximately 5 minutes), volunteers will have to fill a form describing the various stress signs observed (auto-evaluation of their stress),
- If possible group dynamics sessions will be organised weeks 2, 5, 8 and 11. They will last 1 hour .
- Additional interviews with the psychologist may be organised if requested.

**Protocol for the accompanying personnel**

- Two different nurses will make each subject fill a stress sign form, every week when they are not too busy, and every 15 days when the experiment program becomes more demanding.
- All significant events shall be recorded on a special book.

### Psychological follow-up after the experiment

The goal of the debriefing after bed rest is to evaluate psychological state in order to prevent eventual after-effects.

- During the first days after standing-up, anxiety, adaptation to this unusual situation and the way volunteer feel will be investigated using different questionnaires,
- An other questionnaire will evaluate how the volunteer coped with the experiment,
- The volunteer will have to write a free report upon the important facts of the experiment,
- An individual and a group interview will be organised,
- Two or three members of the medical staff will rate adaptation with a brief questionnaire
  (5 questions),
- A collective session about the observations done by the medical staff will be organised,
- Some of these measurements (state of anxiety questionnaire, global health questionnaire and interview) will be done again during the control visits (+30, +90, +180, +360).

A psychological support will be organised if the volunteer have some difficulties to return to “normal” life.

## MEDES team

#### Permanent staff

The permanent staff of MEDES involved in the experiment includes:

- One physician being the coordinating investigator of the study,
- Two physicians in charge of the medical survey of the volunteers,
- One project scientist responsible for the scientific coordination of the protocols including scientific data management and data sharing,
- One pharmacist responsible for the pharmacy and regulatory aspects,
- One physiologist responsible for paramedical team organisation, logistics and biological samples dispatching,
- One dietician responsible for diet monitoring and nutritional balance,
- One technician responsible for reception, installation of biomedical devices.

#### Medical and Paramedical teams

The medical and paramedical teams attached to the Space Clinic will be comprised of 3 physicians, 5 nurses and 6 assistant nurses (ASH).

During the day, these teams will be insure the medical and psychological surveillance, the healthcare and the material comfort of the subjects. There will be permanently present at the clinic a minimum of 1 physician, 2 nurses and 2 auxiliary nurses. This personnel could be increased during the high activity periods.

During the night, the surveillance will be ensured by 1 physician and another person (nurse or assistant, or other non medical clinic personnel). Physicians will be temporarily assigned for the medical follow-up.

#### Critical phase and resuscitation needs

As mentioned previously, one physician specialist in resuscitation will be present in early recovery period and a psychological team will be in charge of the psychological selection and support.

The coordinating investigator or his deputy will be on site or contactable by phone.

All the medical and paramedical staff will be trained to emergency (first aid care). In case of medical problem, the volunteer could be hospitalised in Toulouse Rangueil hospital according to the agreement between Medes and hospital intensive care and emergency department.

A complete information on the research protocols and their potential risks will be given to the hospital resuscitation department.

## Housing conditions for volunteers

### MEDES Clinical Research Facilty (Space Clinic)

The MEDES-Space Clinic opened in June 1996, it is a 1000 m^2^ multipurpose facility located within the Toulouse Rangueil Hospital. The experimental facility offers all the necessary biomedical equipment in order to assure a good and secure work. Thus, the MEDES-CRF obtained the formal approval from Ministry of Health and French Drugs Agency to perform biomedical experiments on healthy volunteers.

It is a strict controlled environment, in which there are 3 main areas:

1. office area (white part, from 22 to 30) and the main entrance (E),
2. A multi-purpose laboratory zone (dotted area: L1-L3, is 120 m^2^ ) with entrance fitted for large objects (2.50m x 2.50 m),
3. The experimental zone with highly controlled environmental conditions (red hatched area) such as: temperature (rooms controlled and monitored within 20-25 ±0.5 °C); light (natural or artificial lighting, light intensity between 0 and 500 Lux); acoustics (isolation from outside, -60 dB): rooms 1-6 are high quality chambers; rooms 7-8 are psychomotor test laboratories; rooms 9-14 & 15-16 are the logistic zone including nursery and biochemical laboratories; modular rooms 17-20 standing for 4 chambers, 4 laboratories or one bigger laboratories up to 64 m^2^; room 21 is a specific laboratory where orthostatic tests (tilt test, stand test and lower body negative pressure test) can be performed.

MEDES Clinical Research Infrastructure

MEDES Clinical Research Infrastructure (MEDES-CRI) is 1 000 m^2^ large, it includes three main areas : 1- Office area (white part, from 22 to 30) and the main entrance (E);
2- Multipurpose laboratory zone (dotted area : L1-L3, is a 120 m^2^ or 1,300 square feet) with entrance fitted for large size objects (2,50 m x 2,50 m or 8.20 to 8.20 feet); 3- The experimental zone with controlled environment[[1]](#footnote-2)1) (hatched area) : rooms 1-6, high quality chambers; rooms 7-8, psychomotor test laboratories; logistics zone (rooms 9-14 and 15-16); modular rooms (17-20: standing for 4 chambers, 4 laboratories or one bigger laboratory); room 21 is a specific laboratory where orthostatic tests (tilt and lower body negative pressure tests or Coriolis stress tests - on rotating chair - can be performed).

The facility configuration allows to suit several imperatives as required by the different protocols (modular zones can be equipped as rooms, laboratories or training areas).

### Diet monitoring

The other overall conditions of this protocol will be the followings :

- liquid intake will be at least 30 ml/Kg/day (in order to minimise renal stone risk) with a maximum 2.5 l/day; At the end of the HDBR period (recovery period; water intake will not be limited to a maximum in order to help the volunteers to restore their fluid volume.
- Caloric intake: For HDT, it is suggested to use 110% of the WHO based BMR, and add 10% for dietary induced thermogesis. BMR is calculated as follows: (15.3 x Bodyweight + 679 ) kcal. For a 70 kg person this will be 2117.5 kcal. During ambulatory control periods, the level should be 140% BMR + 10%, again for a 70 kg person, this will be around 2695 kcal.
- sodium intake around 6 g/day.
- Intake of beverages including tea, coffee or cola will not be allowed.

### Video monitoring

During the whole duration of the experiment, volunteers will remain in the Space Clinic located in Rangueil Hospital (Toulouse). During the -6° Head-Down Tilt (HDT) period, the position will be permanently maintained and controlled by video monitoring.

### Light conditions

The volunteer will follow a "normal" day/night cycle (wake-up around 6h30 am and light will be turn off at 11h00 pm). There will be no artificial light or cycle conditions.

### Transportations

Transfers outside the clinic, to specialised hospital departments (radio, MRI, scanner) will be carried out by ambulances.

During these transfers, the subjects will be assisted by one of the para-medical personnel, and during the 3 months bed rest period and the first days of recovery, by a physician with resuscitation equipment.

### Leisure time

During the entire experiment, the volunteers will have to perform scientific tests, following the overall planning as described in Annex. Unless, this thigh schedule, there will have free time in between the different sessions or during the HDT period when no scientific sessions will be planned.

During their free time, the volunteers will be free to have leisure activities (reading, games), computer activities. A nap in supine position (after lunch, maximum 1h duration) will be allowed during the pre and post HDBR periods. The volunteers will be allowed to make phone calls to their family after scientific sessions performance (6h00 pm- 11h00 pm period).

During the ambulatory BDC and recovery periods, the volunteers will be authorised to go outside the Clinic, staying in the neighbours of the Clinic and with one member of the MEDES paramedical staff.

### Visits to the volunteers

No visit will be allowed during the study. A team of psychologists wil be in charge to ensure psychological selection and support.

## Regulatory aspects

### Agreements

The experiment will be performed within the Space Clinic, location authorised for biomedical research related to space environment on healthy volunteers, without direct individual benefit.

- Agreement for biomedical research in physiology and physio-pathology n° 14027S
- Agreement for biomedical research on drugs n° 14027M

### Volunteer compensation

The volunteers will receive a compensation of 75 000 FF given on 3 years. They will not be allowed to perform biomedical research studies during this period (exclusion period) . In case of withdrawal from the experiment, the compensation will be given to the subject in proportion to the time spent according to the following plan :

- 400FF per day during the 15 ambulatory first days (baseline data collection)
- 600FF per day during the 90 days of HDBR
- 400FF per day during the 15 days of the recovery period
- 400FF per day for the 6 days of long term recovery measurements (1 day (+45,+360), 2 days (+90, +180).

An additional amount will be provided after completion of the HDBR period (5000FF) and after the last day of measurement (+360) (1600FF).

### PI certification

According to the law on biomedical research, each principal protocol has to be performed under the responsibility of a physician, who will be the principal investigator for the Ethics Committee.

To be allowed to perform biomedical research in France, foreign physicians will have to provide the following documents :

- Certificate of accreditation of medical doctor by the member state, according to the agreement on the European Economic Community,
- Certification on his honour that he practises legally medical activity in his country and he is not forbidden to practise medicine by any authority.

MEDES will also register the physicians to a medical administrative organisation (“Conseil de l’Ordre des Médecins”) for the experiment duration with a notification of provision of service in France (date and brief description of the biomedical research to be performed).

# HARDWARE

During the two campaigns of the experiment, different kind of equipments will be used: PI's own equipments (see individual protocols in Part B of this document, MEDES equipments listed hereafter and large equipments from Toulouse hospital (i.e. Cybex 6000, NMRI system, QCT scanner, RADIO, DEXA)

**List of MEDES equipment**

| **Blood drawing kit :**  Neofly® needles, Ohmeda, (Trappes, France)  Vitaflon® catheters, Ohmeda, (Trappes, France)  Vacutainer® tubes, Becton Dickinson, (Meylan, France) |
| --- |
| **Jouan GR412 Cool centrifuge**, Jouan, (St Herblain, France) |
| **Jouan Hematocrit A12 centrifuge**, Jouan, (St Herblain, France) |
| **HEMOCUE® β Hemoglobin photometer**, Hemocue AB, (Angelholm, Sweden) |
| **Jouan VX350 Freeze**r (-80°C) Jouan, (St Herblain, France) |
| **Dinamap 1846 SX**, Critikon Inc. (Tampa, USA) |
| **Cardiovit AT-3 ECG device** Kontron Inst. (Montigny le Bretonneux, France) |
| **Lohmeir M608 ECG/BP Monitor**, (Munchen, Germany) |
| **Arjo 232300 HDT weighing system** Arjo Hospital Equipment AB, (Eslov, Sweden) |
| **Refrigerator** (4°C) |
| **Freezer** (-20°C) |
| **24-h video monitoring** |

# biological samples

## Blood samples

Blood samples will be drawn from an antecubital vein with butterfly needle (or catheter when multiple successive samples) according to the overall planning as figured in Annex. The summary (including name of PIs) of total blood volume to be drawn from each volunteer is presented in the 3 following tables (one per group).

Immediately after blood collection, the tubes will be centrifuged in a cooled centrifuge: time, speed and temperature as requested by the PI's. Then, plasma or serum will be distributed in plastic tubes to be stored in freezer either –20°C or –80°C temperature as requested by PI's until transportation to the PI's laboratory for analyses.

## Urine samples

During the experimental campaign, 24-h urine outputs collections will be performed according to the requests of PI's (Drs. Felsenberg, Ohshima and Ferretti) and as figured in the overall planning. For each 24-h period, it will be collected from the second void of the morning to the first void the following day. During that period of 24 hours, urine will be kept at 4°C.

Once the 24-h collection period will be terminated, samples will be made (according to volume requested by the PI's for assays) in plastic bottles and stored at –20°C until transportation to the PI's laboratory for analyses.

## Saliva samples

During the experimental campaign, saliva samples will be collected during Dr. Pagani & Dr. Bareille protocols according to the requests of PI's and as figured in the overall planning. Samples will be stored at –20°C until transportation to the PI's laboratory for analyses.

## Stools samples

During the experimental campaign, 96-h stool's collections will be performed according to the requests of PI's (Dr Ohshima) and as figured in the overall planning. Each 96-h period sample will be stored at –20°C until transportation to the PI's laboratory for analyses.

## Storage and transportation

Most of the biological samples will be kept in the Clinic until the end of each campaign with exception of biopsy samples which will be transferred to PI's laboratories right after the biopsies for analyses.

At the end of the campaigns, the biological samples will be sent to the different PI's laboratories to be analysed. Each transportation will be done with adequate means to preserve the samples during the whole transport duration (ice, dry ice,…).

**Table: Total volume of blood samples for Control group and differences between the 3 groups**

**Table: Total volume of blood samples for Exercise group**

**Table: Total volume of blood samples for Biphosphonate group**

# risks & hazards

## Identified risks and lack of comfort associated with the situation of anti-orthostatic bed rest

- During the first days of bed rest there may appear an impression of “full” head, sometimes with headaches. These phenomena are transitory and will regress in 2 or 3 days,
- Possibility of back pain and skin irritations due to bed rest.
- Thrombophlebitis risk: this risk is low for healthy subjects without particular prior indication, also considering that the subject is not obliged to total immobility and that medical surveillance is constant (clinical attendance, plethysmography...),
- The risk that kidney stones may appear is 1/10 000. To prevent this problem it is recommended to drink water more than 30 ml/kg/day.
- Reduction of the muscular mass: prolonged bed rest at –6° was found to induce a muscular mass reduction of about 2-3% by week could be 25% on 4 weeks.
- Rising after an anti-orthostatic bed confinement at -6° may cause a low arterial blood pressure. The head up tilt test will therefore be progressive and medically checked (Arterial blood pressure, heart rate) until the standing position is fully tolerated. Precision on this is given hereafter (cf. Tilt Test protocol),
- Aptitude to effort: after an anti-orthostatic bed rest at –6° exists a decrease in the capability to produce physical efforts. The main reason is the lack of activity during the whole test period. After rising, the volunteer’s aptitude will be restored within the following days
- Loss of bone : the bone density modifications are very variable. After 6 months of flight the bone density decrease has been observed about 23 % (tibia) and 3% (radius forearm bone).

## Risks and lack of comfort due to the drugs taken by the subject during the experimentation

- The side effects of Pamidronate (Aredia®, Novartis) are the followings:

- The most frequent observed side effects are transient fever (1-2°C), usually 2 days after administration, lasting 24-48 hours and without clinical effect. A flu associated with discomfort, fatigue, rigidity and vasomotor reactions are also frequent. Slow infusion (3 hours) of this drug would minimize these side effects.

- Sometimes, its administration can produce:

- - - local reactions at the injection site : pain, flush, indurations, swelling, phlebitis,
    - side effects concerning locomotor systems : transient bone pains, muscle pain, joint pain or generalized pains,
    - gastro-intestinal side effects : nausea, vomiting,
    - effects on central nervous system side effects: headaches
    - blood effects: lymphocytopenia which does not have a long duration and without any clinical effect

- Rarely are reported : muscle cramps, abdominal pains, diarrhea or constipation, anorexia, confusion, dizziness, sleep disorders, anemia, leucopenia, hypo or hypertension.

- Paracetamol or acetaminophen (Dafalgan ®, Doliprane®) : skin rashes and other allergic reactions occur occasionally. Exceptionally thrombocytopenia have been reported.
- Xylocaïne ® : used to do a local anesthesia for the biopsy. Side effects apparent after local anesthesia may be due to the anesthetics or to errors in technique. Allergic reactions to local anesthetics are rare. The systemic toxicity of local anesthetics mainly involves the central nervous system (nervousness, dizziness, excitement, vomiting, nausea,…) and cardiovascular system (myocardial depression and peripheral vasodilatation resulting in hypotension and bradycardia).
- Others substances used like markers in the different protocols. The risks and inconveniences will be explained within each protocol.

## Risks and lack of comfort due to electrodes

The installation of electrodes on the skin (ECG, EMG...) may result in local irritation problems. These usually regress fairly rapidly.

## Risks and lack of comfort due to blood samples

A very slight haematoma and/or infection risk at the venous puncture points may occur. The usual precautions will be taken by the nurses to avoid this problem.

In order to avoid the multiplicity of punctures, and to avoid any nuisances created by the blood sampling, venous catheters will be used, which may create some lack of comfort. To avoid the infection risks, the same catheter will not be kept in place more than 48 hours.

A total quantity of 869 ml, or 812 ml or 739 ml of blood sample (respectively for control, drug and exercise groups) shall be withdrawn during the whole experiment (from inclusion visit to the last measurements). 699 ml or 642 ml or 569 ml (respectively for control, drug and exercise groups) will be collected during the 3 months of bed rest.

## Risks due Radiations

The total exposure to x-rays will be 10 807 μSievert on 18 months which is less than the allowable limit for human health (20 000 μSievert/year).

| **Inclusion Visit** | **During the 120 days** | **Control 6 months after (+180)** | **Control 12 months after (+360)** |
| --- | --- | --- | --- |
| 2524 μSievert | 7441.2 μSievert | 836.8 μSievert | 5 μSievert |

1. ? controlled environment means: natural/artificial light (with modular intensity from 0 to 500 lux), air conditioned (temperature from 20 to 26 ± 0.5 °C, humidity: 45-55%), acoustic isolation

   (-60 dB from outside) and possibility to isolate the whole experimental infrastructure from outside (doors and windows closed) thus creating confinement conditions for the study participants. [↑](#footnote-ref-2)
