# Supplementary material for: Effect of Physical Inactivity on the Oxidation of Saturated and Monounsaturated Dietary Fatty Acids: Results of a Randomized Trial
Source: PLoS Clin Trials. 2006 Sep 29;1(5):e27. doi: 10.1371/journal.pctr.0010027 (PMC1584255; doi:10.1371/journal.pctr.0010027)
Supplement: Trial Protocol Part B [file pctr.0010027.sd003.doc]

# TABLE OF CONTENTS

Part B

6 scientific protocols [1](#__RefHeading___Toc508430894)

**6.1 Effects of 90 days 6 degree head-down tilt bed rest on musculo-skeletal system, renal stone risk, and back pain.** [**3**](#__RefHeading___Toc508430895)

6.1.1 Scientific Background [3](#__RefHeading___Toc508430896)

6.1.2 Objectives and hypothesis [5](#__RefHeading___Toc508430897)

6.1.3 Specific inclusion and non-inclusion criteria for test subjects [6](#__RefHeading___Toc508430898)

6.1.4 Precise description of the experimental protocol [7](#__RefHeading___Toc508430899)

6.1.5 Protocol summary (one page), written on a separate sheet [13](#__RefHeading___Toc508430900)

6.1.6 Investigators [14](#__RefHeading___Toc508430901)

**6.2 Effects of long duration bed rest on sleep and core body temperature rhythm with and**

**without exercise.** [**19**](#__RefHeading___Toc508430902)

6.2.1 Scientific Background [19](#__RefHeading___Toc508430903)

6.2.2 Objectives and hypothesis [21](#__RefHeading___Toc508430904)

6.2.3 Specific inclusion and non-inclusion criteria for test subjects [22](#__RefHeading___Toc508430905)

6.2.4 Precise description of the experimental protocol [22](#__RefHeading___Toc508430906)

6.2.5 Protocol summary (one page), written on a separate sheet [25](#__RefHeading___Toc508430908)

6.2.6 Investigators [25](#__RefHeading___Toc508430909)

**6.3 Bone loss and muscle hyptrophy under head-down tilt and resistance training and**

**bisphoshponates.** [**26**](#__RefHeading___Toc508430910)

6.3.1 Scientific Background [26](#__RefHeading___Toc508430911)

6.3.2 Objectives and hypothesis [28](#__RefHeading___Toc508430912)

6.3.3 Specific inclusion and non-inclusion criteria for test subjects [29](#__RefHeading___Toc508430913)

6.3.4 Precise description of the experimental protocol [30](#__RefHeading___Toc508430914)

6.3.5 Protocol summary [36](#__RefHeading___Toc508430915)

6.3.6 Investigators [36](#__RefHeading___Toc508430916)

**6.4 Effects of resistance exercise, using fly-wheel technology, on muscle size and function in healthy bedridden subjects.** [**37**](#__RefHeading___Toc508430917)

6.4.1 Scientific Background [37](#__RefHeading___Toc508430918)

6.4.2 Objectives and hypothesis [44](#__RefHeading___Toc508430919)

6.4.3 Specific inclusion and non-inclusion criteria for test subjects [45](#__RefHeading___Toc508430920)

6.4.4 Precise description of the experimental protocol [45](#__RefHeading___Toc508430921)

6.4.5 Protocol summary (one page), written on a separate sheet [49](#__RefHeading___Toc508430922)

6.4.6 Investigators [49](#__RefHeading___Toc508430923)

**6.5 Validation of physical and pharmacological countermeasures to maintain optimal stress responses in astronauts** [**50**](#__RefHeading___Toc508430924)

6.5.1 Scientific Background [50](#__RefHeading___Toc508430925)

6.5.2 Objectives and hypothesis [53](#__RefHeading___Toc508430926)

6.5.3 Specific inclusion and non-inclusion criteria for test subjects [54](#__RefHeading___Toc508430927)

6.5.4 Precise description of the experimental protocol [55](#__RefHeading___Toc508430928)

6.5.5 Protocol summary [60](#__RefHeading___Toc508430929)

6.5.6 Investigators [60](#__RefHeading___Toc508430930)

**6.6 Effects of a long-term bed rest on muscle integrity and cytoskeletal proteins, with and**

**without muscle exercise countermeasures.** [**61**](#__RefHeading___Toc508430931)

6.6.1 Scientific Background [61](#__RefHeading___Toc508430932)

6.6.2 Objectives and hypothesis [65](#__RefHeading___Toc508430933)

6.6.3 Specific inclusion and non-inclusion criteria for test subjects [66](#__RefHeading___Toc508430934)

6.6.4 Precise description of the experimental protocol [66](#__RefHeading___Toc508430935)

6.6.5 Protocol summary (one page), written on a separate sheet [68](#__RefHeading___Toc508430936)

6.6.6 Investigators [69](#__RefHeading___Toc508430937)

**6.7 Effects of posture on drug absorption mechanism : Pharmacokinetics of acetaminophen**

**used as a probe to study oral absorption in simulated weightlessness.** [**70**](#__RefHeading___Toc508430938)

6.7.1 Scientific Background [70](#__RefHeading___Toc508430939)

6.7.2 Objectives and hypothesis [73](#__RefHeading___Toc508430940)

6.7.3 Specific inclusion and non-inclusion criteria for test subjects [74](#__RefHeading___Toc508430941)

6.7.4 Precise description of the experimental protocol [74](#__RefHeading___Toc508430942)

6.7.5 Protocol summary [79](#__RefHeading___Toc508430943)

6.7.6 Investigators [80](#__RefHeading___Toc508430944)

**6.8 Validation of measures against backpain during bed rest** [**81**](#__RefHeading___Toc508430945)

6.8.1 Scientific Background [81](#__RefHeading___Toc508430946)

6.8.2 Objectives and hypothesis [82](#__RefHeading___Toc508430947)

6.8.3 Specific inclusion and non-inclusion criteria for test subjects [82](#__RefHeading___Toc508430948)

6.8.4 Precise description of the experimental protocol [82](#__RefHeading___Toc508430949)

6.8.5 Protocol summary (one page), written on a separate sheet [85](#__RefHeading___Toc508430951)

6.8.6 Investigators [85](#__RefHeading___Toc508430952)

**6.9 Molecular plasticity of human skeletal muscle compartments after bed rest.** [**86**](#__RefHeading___Toc508430953)

6.9.1 Scientific Background [86](#__RefHeading___Toc508430954)

6.9.2 Objectives and hypothesis [89](#__RefHeading___Toc508430955)

6.9.3 Specific inclusion and non-inclusion criteria for test subjects [90](#__RefHeading___Toc508430956)

6.9.4 Precise description of the experimental protocol [90](#__RefHeading___Toc508430957)

6.9.5 2 Protocol summary [93](#__RefHeading___Toc508430958)

6.9.6 Investigators [93](#__RefHeading___Toc508430959)

**6.10 Respiratory, circulatory, muscular and metabolism consequences of inactivity in humans.** [**94**](#__RefHeading___Toc508430960)

6.10.1 Scientific Background [94](#__RefHeading___Toc508430961)

6.10.2 Objectives and hypothesis [95](#__RefHeading___Toc508430962)

6.10.3 Specific inclusion and non-inclusion criteria for test subjects [96](#__RefHeading___Toc508430963)

6.10.4 Precise description of the experimental protocol [97](#__RefHeading___Toc508430964)

6.10.5 Protocol summary (one page), written on a separate sheet [102](#__RefHeading___Toc508430965)

6.10.6 Body composition and Lipid metabolism (Annex of point 3 of Protocol 6.10) [103](#__RefHeading___Toc508430966)

6.10.7 Investigators [109](#__RefHeading___Toc508430970)

**6.11 Assessment of lower and upper limbs vascular hemodynamics during long term bed rest.** [**110**](#__RefHeading___Toc508430971)

6.11.1 Scientific Background [110](#__RefHeading___Toc508430972)

6.11.2 Objectives and hypothesis [114](#__RefHeading___Toc508430973)

6.11.3 Specific inclusion and non-inclusion criteria for test subjects [114](#__RefHeading___Toc508430974)

6.11.4 Precise description of the experimental protocol [115](#__RefHeading___Toc508430975)

6.11.5 Hazards, side effects, possible inconveniences, discomfort and constraints to subjects [123](#__RefHeading___Toc508430976)

6.11.6 Protocol summary (one page), written on a separate sheet [124](#__RefHeading___Toc508430977)

6.11.7 Investigators [125](#__RefHeading___Toc508430978)

# scientific protocols

Here after are presented the individual scientific protocols. Curriculum vitae of each Principal Investigator (PI), Investigator (Inv) or Co-Investigator (CO-I) are presented in the Annex Part of the document.

The following Table summarized for each protocol part on which group of volunteers it will be applied.

## Effect of 90 days 6 degree head-down tilt bed rest on musculo-skeletal system, renal stone risk, and back pain.

### Scientific Background

#### Problem presentation

**On bone loss and renal stone risk during space flight**

The potential for significant loss of bone is one of the most important medical concerns of International Space Station (ISS) medical operation ([1, 2] in the next item). Loss of bone mass is hazardous to astronauts on long duration flights, not only because hypercalciuria might lead to the formation of renal stone during flight, but axial skeletal fractures may occur on return to the Earth’s gravity. Besides, osteoporosis may greatly impact their quality of life after they retired.

Disuse bone loss has not yet been prevented sufficiently by any of exercise countermeasures. Biochemical intervention could have an important role; however, more research is needed regarding the candidate drugs and their regimens.

As the research opportunities in space are relatively limited, there is a need to use ground-based model (-6 degree head-down tilt bed rest) to validate the countermeasures to prevent bone loss and renal stone.

**On body composition changes during space flight**

The effects of weightlessness include body weight loss, caused by loss of body fluid, which occurs after fluid shift at the early stage of space flight, and disuse atrophy of muscles. Significant loss of weight is not a negligible problem on health care for the crew. As for the muscle atrophy, it may adversely affect not only crew health but crew performance during the missions. There is a need to develop a simple in-flight device with which we can monitor changes in body composition that reflect changes in muscle volume. In this experiment, we would like to study the usefulness of a commercially available device based on the BIA (bioelectrical impedance analysis), which is expected to meet this need.

**On back pain during space flight and bed rest**

Low back discomfort was one of the more frequently described medical problems observed during space flights and bed rest studies. However, there were few reports of the incidences of back pain or descriptions of the pain patterns in bed rest subjects.

#### Literature data including references

**On pharmacological countermeasures for bone loss and renal stone**

Various biochemical attempts to prevent bone loss and the negative calcium balance during long-term bed rest have been done. These therapeutic attemps include phosphate supplements, oral calcium and phosphate, synthetic salmon calcitonin, but little or no protection was afforded by these three methods during bed rest [3].

The bisphosphonates have received attention over the past three decades because of their potential therapeutic usefulness in osteoporosis. There were also trials to use ethidronate in weightlessness induced osteoporosis. In 1972, Dr. D. Lockwood *et al.* attempted continuous administration of low and high doses Ehidronate during 20 weeks bed rest and they met with limited success, expressed by urine hydroxyproline [4]. In 1992, Dr. A. Grigoriev *et al*. also tried exercise countermeasure together with and without Ethidronate in his 360 days bed rest study with continuous administration of 900mg/day, the results of which indicated the potential of the drug in preventing the disuse osteoporosis [5].

As was proved later that dosages of Ethidronate above 800 mg/day impair the normal skeletal mineralisation [6], it may be possible that the dosages of Ethidronate attempted in the previous bed rest studies were too high. Though there have been still arguments regarding optimal doses and regimens (continuous versus intermittent) [7], the evidence-based and recommended clinical regiment for osteoporosis [8] is to take 400 mg of ethidronate in Europe, in order to suppress bone resorption for a two-week period, followed by a drug-free period of 3 months, during which bone formation may proceed at a normal rate ; and repeat this cycle. As there were no significant adverse effects at this dose for more than four years, this regimen have been repeatedly tried in animal and human studies. In an animal study [9], it is demonstrated that the intermittent regimen not only maintain the mass of bone but preserve the mechanical property of bone. Dr. Fujita reported that, in the patients of post menopausal and senile osteoporosis, the lumbar vertebral mineral density significantly (p<0.001) increased by 1.4+/- 0.5% [mean+/- S.E.] by one cycle of 400 mg of Ethidronate administration from the pretreatment levels [10]. This intermittent regimen will be studied in this protocol in order to clarify the persisting protective effect of the drug on bone metabolism and calcium balance in healthy young male during immobilization.

[1] A. LeBlanc. *et al*.: Bone Mineral and lean tissue loss after long duration spaceflight. J. Bone Miner. Res. 1996 11: S323.

[2] S. M. Smith *et al*.: Calcium metabolism before, during, and after a 3-mo spaceflight : kinetic and biochemical changes. Am. J. Physiology 1999 July 277 (1 Pt 2): R1-10.

[3] V. S. Schneider and J. McDonald: Skeletal Calcium Homeostases and Countermeasures to prevent disuse osteoporosis. Calcif Tissue Int 1984 36: S151-154.

[4] D. R. Lockwood *et al*.: Effect of the disphosphonate EHDP on bone mineral metabolism during prolonged bed rest. J Clin Endocrinol Metab 1975 41: 533.

[5] A. Grigoriev *et al.*: Effect of exercise and bisphosphonate on mineral balance and bone density during 360 day antiorthostatic hypokinesia. J Bone Miner Res 1992 Dec 7 Suppl 2: S449-55.

[6] S. Adami and N. Zamberlan : Adverse effects of bisphosphonates. A comparative review. Drug Saf Mar, 1996 14(3): 158-70.

[7] N. Watts: Treatment of osteoporosis with bisphosphonates. Rheum Dis Clin North Am 1994 Aug 20(3): 717-34.

[8] N. Watts *et al.*: Intermittent cyclical ethidronate treatment of postmenopausal osteoporosis. N Engl J Med 1990 Jul 12 ;323(2) :73-9.

[9] Katsumata et al: Intermittent cyclical ethidronate treatment maitains the mass, structure and the mechanical property of bone in ovariectomized rats. J Bone Miner Res 1995 Jun; 10(6) :921-31.

[10] T. Fujita: Double-blind muticenter comparative study with alfacalcidol of ethidronate disodium (EHDP) in involutional osteoporosis: Clin Eval (Rinsho Hyouka) 1993 21 : 261-303.

**On bioelectrical impedance method for body composition monitoring**

Bioelectrical impedance method enabled the monitoring of body fat and weight with nearly the same level of accuracy achievable as dual energy X-ray absorptiometry (DEXA) and hydrodensitometry (underwater weighing) [1, 2]. With the device based on this method, we can monitor the changes both in body fluid distribution and in the body % fat which is expected to have high correlation with muscle volume.

[1] Evaluation of dual energy X-ray absorptiometry as a method of measurement of body fat. Eur J Clin Nutr 1993 Mar;47(3):216-28.

[2] Reliability and validity of bioelectrical impedance in determining body composition. J Appl Physiol 1998 Feb;64(2):529-34.

**On Back pain**

There was a brief review of the medical records of orbital crew members reported by Dr. Wing. Sixty-eight percent of the astronauts had reported to the post-flight medical examiner that they had experienced back pain during the mission [1]. Dr. Styf described the back pain intensity of subjects of bed rest study, but the duration of bed rest was only 3 days [2]. Therefore, we would like to investigate the back pain patterns of the subjects of 90 days bed rest study.

[1] P. Wing et al.: Back pain and spinal changes in microgravity. Orthop Clin N Am 1991; 22:255-262.

[2] JR Styf, et al. Height increase, Neuromascular function and back pain during 6 degree head down tilt with traction. Avait Space Environ Med 68: 24-29, 1997

#### Protocol justification according to current scientific knowledge

The intermittent regimen of ethidronate has been proven to be effective for post-menopausal osteoporosis, but has never been tried for immobilization-induced osteoporosis in humans of the same generation as the astronauts.

The results of this experiment will be deeply related to the future health care program for the ISS astronauts, and cannot be replaced by animal study.

#### The interest of the duration of the head-down bed rest has also to be justified, in particular for bone changes.

Ninety-day is not only one cycle period in ethidronate regimen but a realistic period as a space mission for ISS astronauts. It is long enough to compare the results between control group and pharmacological group, for the densities of weight-bearing bones significantly change in 90 days immobilization as reported in previous study.

#### Rationale for drug (if used) and dosage selection

Subjects will receive Aredia infusion (Novartis), 60 mg 14 days before the beginning of the bed rest period. At the beginning, we planned to have test Ethidronate administration. Ethidronate is clinically approved for osteoporosis, and is widely used

in Japan. Its efficacy is being well-established as the supporting evidences have

accumulated, It is also a well-tolerated drug with quite rare and minor effects.

It is a pity that most of the trials using Ethidronate in Japan are not translated in English.

We met with an opinion from Dr. Felsenberg that Ethidronate is an old-fashion drug with week efficacy. When we consider the number of evidences, published in international journals, that support newer generation bisphosphonate, we decided to replace Ethidronate with other bisphosphonate.

Dr. Felsenberg strongly recommended Ibandronate, but we think its efficacy is still doubtful. Another candidate for us was Alendronate as its strong efficacy is well-established and it is clinically approved for osteoporosis. However, its adverse effect to make esophageal erosions made us gave up Alendronate administration for bed rest subjects.

Finally, we come to think of intravenous Pamidronate administartion. Although it is not approved for osteoporosis for economical reason in Japan nor in France, its antiresorptive effect is well-established in Paget's disease and hypercalcemia in malignant cancers in many countries and severe osteoporosis in osteogenesis imperfecta and its adverse effects are minor ones. Dr. Felsenberg compromised with our idea, and thus, we reached an agreement.

References

1) The effect of pamidronate on lumbar spine bone density and pain in osteoporosis secondary to systemic mastrocytosis, by Marshall A , et al, J of Rheumatology 36:1997, 393-396

2) Glorieux GH, et al: Cyclic administration of pamidronate in children with severe osteogenesis imperfecta. New Engl J Med 339:947-952, 1998.

### Objectives and hypothesis

#### Main objective of the protocol

(1) To determine the efficacy of pharmacological protocol to prevent disuse bone loss and renal stone risk, as assessed by the parameters in the next item.

(2) To monitor the changes of muscle volume during 90 days bed rest and to prove the usefulness of a specific device in monitoring muscle mass change during bed rest by the comparison study with more validated method (DEXA).

#### Identification of main parameters

For bone metabolism analyses

- Bone mineral densitometry of lumbar spine, neck of femur, whole body (including skull), heel and forearm (These measurements are included in ITR measurements and described elsewhere.)
- Calcium balance
- Metabolic bone markers
- Bone regulating hormones
- Renal stone risks

For disuse muscle atrophy analyses

- body weight, height, impedance values of each body section (upper and lower extremities of both sides and trunk)
- Isokinetic muscle strength (using Cybex)
- MRI measurements

Parameters for secondary object (see 6;2.5)

- Back pain score and height

#### Tested hypothesis

(1) Administration of ethidronate for 2 weeks before bed rest will prevent or inhibit:

- bone loss,
- bone resorption acceleration, and
- urinary calcium increase associated with renal stone risk increase

induced by 90 days bed rest.

(2) The impedance values of the subjects will well correlate with their lean body mass values measured by DEXA for the time course of bed rest.

#### Expected results

1. Subjects who received pamidronate will not experience bone resorption acceleraration (monitored by sensitive indicators) and bone mineral density losses (monitored by osteodensitometer) seen in the control group. In the pharmacological group, bone may be maintained, or may show ameliorated losses as compared to the control group. The persistent negative calcium balance during bed rest documented in the previous studies will be significantly more positive.
2. In the pharmacological group, renal stone risk profile will not change significantly, while that in the control group may show tendency to stone formation.
3. If the above-written hypothesis (2) is correct, the device attempted in this study can be a good candidate to be used for in-flight monitoring of body composition.

#### Secondary objectives ( if necessary)

We would also like to elucidate the low back pain patterns of subjects of 90 days bed rest study. Subjects will be asked to fill in the questionnaires (see Table 4) on back pain parameters such as location, quality, severity, and moment of low back pain; and the patterns of low back pain will be assessed.

### Specific inclusion and non-inclusion criteria for test subjects

Selection tests will includes:

- Class III physical (in Federal Aviation Regulations/Aeronautical Information Manual, Page: F-160)
- DEXA scanning for bone mineral density
- HIV/Hepatitis screening
- Drug screen by questionnaire

Inclusion criteria

Healthy males between the ages of 25 and 40 who respond to a request for research subjects from general population in Europe

Non-inclusion criteria

- - Failure to pass a Class III airman medical certificate of Federal Aviation Regulations (FAR)
  - Bone mineral density more than 1.0 standard deviations above or below the age/sex-matched mean.
  - HIV, hepatitis
  - Acute disease other than acute upper respiratory infections and minor skin diseases (example: dermatophytosis)
  - Chronic diseases other than minor skin diseases (example: acne)
  - Long-term corticosteroid treatment
  - Family history of thrombosis
  - Gastritis, Hiatal hernia
  - History of low back surgery
  - History of renal stones
  - Medications that by their action interfere with the interpretation of the results (examples: fluoride, estrogen, testosteron)
  - Metal implants
  - Pacemaker or other implanted medical device
  - Prior neurological disorder, musculoskeletal injury or cardiovascular disease
  - Thyroid disturbances

NOTE: Subjects should be randomly assigned to the three groups so that there is no statistically significant difference among the three groups in age, weight, height, body mass index, percent fat and bone mineral density of the whole body. P value of each pair should exceed 0.05.

### Precise description of the experimental protocol

#### Tests description (methods, duration, time of day, protocol conditions, etc.)

Twenty eight subjects will participate in the 90 days bed rest study. They will be divided in three groups (control group, exercise group, and pharmacological group).

Attached table shows overall time schedule of data collection activities.

***For the subjects in control and pharmacological groups***

Subjects of pharmacological group will receive pamidronate (AREDIA®, Novartis). They will receive 60 mg of Aredia by one single infusion (duration 3 hours at BDC-14) before bed rest.

***For the subjects in all three groups***

Urine and venous blood biochemical data will be collected before, during and after bed rest. Calcium balance will be evaluated by intake from food and output in urine and feces. Imaging measurements with Dual Energy X-ray Absorptiometry (DEXA) and Magnetic Resonance Imaging (MRI) will be collected before during and after bed rest. Isokinetic muscle strength will be measured by Cybex-Norm dynamometer. Back pain during bed rest will be assessed by low back pain record. Height will be measured using tape measure.

Similar clip electrodes as in the ECG measurements will be attached to the right and left wrist and ankle of the subjects at the supine position. Measurements will be performed for one minute. Whole procedure from preparation to removal of the device may require five minute per one subject.

Body weight will be measured after urination every morning on the same day as impedance measurements. Height will be measured using tape measure at the same time.

#### Measured parameters

For all three groups:

- Bone mineral densitometry of lumbar spine, neck of femur, whole body (including skull), heel and forearm.
- Metabolic bone markers
- Bone regulating hormones
- Renal stone risks
- Ca balance evaluation
- Muscle volume
- Isokinetic Muscle strength
- Back pain record and height
- Impedance values, body weight

#### Equipment used

- Blood and Urine sampling
  - - - Only the general laboratory equipment & supplies are to be employed in the procedure such as sample tube and freezing container, blood centrifuge apparatus, freezer, syringes, needles, test-tubes, sterilizer, cotton and dry-ice, Urine collection container
- Measurement for imaging

1. Dual Energy X-ray Absorptiometry (DEXA)

Bone mineral content of lumbar spine, neck of femur, whole body (including skull), heel and forearm will be measured with a Lunar DPX-L (Lunar France). Percent fat and lean body mass will also be measured. These measurements are included as ITR measurements.

1. Abdominal X ray for checking renal stone
2. Magnetic Resonance Imaging (MRI)

Lower leg muscle and lumber spine will be measured with a Magneton Impact Expert 1T (Siemens SA medical division). Any activities that require muscle strain such as physical training and muscle strength measurements must not be performed within 24 hours before MRI measurement.

- Measurements of isokinetic muscle strength (Cybex-Norm dynamometer)
  - Right knee, ankle and trunk testing for isokinetic muscle strength will be conducted with a Cybex 6000 (Medimex) only at concentric mode.
  - Angular velocity and repetitions for each measurement are shown as follows:
  - Trunk: Flexion/Extension: 60 degree/sec.
  - Ankle: Planter Flexion/Extension: 0, 30, 60 degree/sec
  - Knee: Flexion/Extension: 0, 30, 90, 180 degree/sec
  - Endurance of concentric knee extension: 180 degree/sec: 30 reps
  - Isokinetic muscle strength measurements must be conducted after MRI, DEXA.
- Measurements of body composition by bio-electrical impedance method. Modified commercially available H/W made by Tanita Co., Ltd will be used. Clip electrodes will be attached to the right and left wrist and ankle of the subjects at the supine position. See Attachment 4. Measurements will be performed for one minute and whole procedure from preparation to removal of the device may require five minute per one section. Measured parameters are percent fat, fat mass, lean body mass, and estimated muscle mass.
- Measurement for height: Standard tape measure

#### Description of the test termination criteria of the protocol

Termination will be considered if :

- Life-threatening events or serious side effects occur in the pharmacological group.
- Clinical data indicate any abnormality that result in serious consideration of study termination
- Physician in MEDES decide that the subject should withdraw from the experiment.
- The subject express his will to withdraw from the research

Any incidents that result in serious consideration of study termination will be communicated from MEDES physician to NASDA PI in a timely fashion.

#### Specific medical monitoring (ECG, blood pressure monitoring, etc.) (if necessary)

Specific medical monitoring will be defined as ITR and will be described in other documents.

It is recommended that water intake should be more than 30 ml/kg/day to prevent renal stone formation by hypercalciurea.

#### Other specific requirements (dietary, conditions of the experiment sessions, etc.)

- DEXA measurements quality control

Human phantom scanning should be performed during the experimental period including baseline and recovery period. To ensure the reliability, We would like to request DEXA raw data to be conveyed to Japan electronically (hopefully by e-mail as attached files, alternatively by special delivery in a floppy disc), immediately after the measurement. According to these raw data, we may request re-measurement.

- MRI

Any activities that require muscle strain such as physical training and muscle strength measurements must not be performed within 24 hours before MRI measurement.

- Isokinetic muscle strength

Isokinetic muscle strength measurements must be conducted after MRI and DEXA.

#### Biological samples handling, preservation conditions, transport

##### Measured parameters

#### *Blood Samples*

At each of the scheduled time points (see Table. 1 in the appendix 1), venous blood will be collected conventionally before breakfast. All blood draws should be performed at the same time of day*.* At each section, 15 ml of blood will be needed before BR (-14,-1), during BR (3,7,14,30,60,90) and after BR (+3,+7,+14,+45,+90, +180, +360). Additionally, 3 ml of blood will be drawn during bed rest (HDT2), and after bed rest (R+2. For blood biochemistry analysis, serum will be separated by centrifuge. The total volume of blood drawn for the entire study will be approximately 171 ml. Measured parameters are listed in the next page (and also in Table 2).

NOTE : Asterisks are attached to the parameters that are measured as the ITR measurements on some specific days.

Blood samples are analysed for:

　a) Biochemistry

1. Erythrocyte* and leukocyte count*, Hemoglobin*, Hematocrit*
2. Glucose*
3. Electrolyte (Na*, Cl*, Ca*, P*, K*, and Mg)
4. Total Protein* and Albumin*
5. Creatinine*
6. Markers of bone metabolism

　・Bone formation markers

1. Intact Osteocalcin (Bone Gla Protein)
2. Alkaline phosphatase (osteo-type)
3. PICP (Procollagen type 1 calboxy-terminal propeptide)

・Bone resorption marker

1. Beta-CTX (Type 1 collagen cross-linked C telopeptide)

c) Calcitropic hormone

1. Intact-PTH
2. 1α25-Dihydroxyvitamin D (Calcitriol)

Blood samples are analysed for:

　 a) Biochemistry

- 1. Erythrocyte* and leukocyte count*, Hemoglobin*, Hematocrit*
  2. Glucose*
  3. Electrolyte (Na*, Cl*, Ca*, P*, K*, and Mg)
  4. Total Protein* and Albumin*
  5. Creatinine*

#### *Urine samples*

At each of the scheduled time points (see Table. 1 in the appendix 1), we would like to stock as much volume of twenty-four hours urine as possible. The methods for urine storage and measurements is up to MEDES Hospital. Urine volume for 24 hours will be measured first and samples will be analyzed for the following parameters.

Urine samples are analyzed for:

1. Urine volume (24-hour urine)*
2. Urine pellet (WBC, RBC, Epitheliocyte, Mucus, Crystals and Casts) by

microscopy

1. Biochemistry
2. Electrolytes (Na, Cl, Ca, P, K, and Mg)
3. Creatinine
4. Urine Urea Nitrogen
5. Uric Acid,
6. Sulfate
7. Markers of bone metabolism*
8. NTX (Type I collagen cross-linked N-telopeptide)
9. Beta-CTX (Type I collagen cross-linked C-telopeptide)
10. Free and total Deoxypyridinoline using Pyrilinks-D-kit
11. Renal stone risk
12. Osteopontin
13. Citric acid
14. Oxalate acid

- For Ca balance study

Food monitoring is included as ITR measurements. Calcium excretion in 24 hours urine and feces will be measured before bed rest (from -15 to –12; from
 -4 to –1), during bed rest (from 4 to 7; from 27 to 30; from 57 to 60, from 87 to 90), and after bed rest (from +4 to +7, from +11 to +14).

##### Methods, equipment and place of analysis

See table 2 and 3 for the information on the methods of biochemical analyses. Every measurements will be conducted in MEDES, Toulouse.

The method for height measurement:

(1) During the bed rest study, height from bottom of the feet to head in spine position will be measured by at 5 P.M. by tape measure.

(2) To assess normal diurnal changes prior to bed rest study, spinal length will be measured before bed rest study at 6 A.M and at 5 P.M. The measurements at 7 A.M. will be taken in supine position before the stand up following one night of horizontal sleep. The 5 P.M. height measurement will be taken in standing position.

#### (3) After the bed rest period, height will be measured in standing position at 5 P.M. ( or in the afternoon after R+ 14).

##### Labelling, storage and packaging of the samples

- Labelling

Labelling of the sample tubes is up to MEDES. If needed, please use the symbols listed in Tables 2 and 3. Every subject should be given a corresponding number to preserve his anonymity.

Example: Serum-Subject No.-Day-Ca-Aliquot No.

- Storage

Blood osteocalcin (6;4.7.1 b), 1) is especially sensitive to temperature. We would like the serum samples to be stored frozen at –80^o^C within 15 min from serum sampling and please avoid repetition of dissolving and frozing.

Flow of serum preparation

Allow blood to clot at room temperature.

🡪 3,000 rpm at 4 ^o^C 10 min.

🡪 Collect the supernatant and stock in a -80 ^o^C freezer if needed.

Flow of urine preparation

Clollect urine in a container for 24 hours

🡪 Volume measurement.

🡪 Divide the sample and put into several tubes, stock in a -80 ^o^C

freezer if needed.

#### Data utilisation and analysis, specify

##### Parameters analysed

Data from control subjects will be used to define the amount of bone loss that will be considered indicative of inhibition of bone loss.

All data for each subject will be normalized, with their baseline values taken as 100%. The normalized data will be plotted against the number of days after the start of bed rest or recovery.

##### Statistical tests

#### *Correlation tests.*

##### Software used

Standard software.

#### Hazards, side effects, possible inconveniences, discomfort and constraints to subjects

##### Hazards and protection to minimize risks

Total exposure to X-rays is summarised in a dedicated table of chapter 5.

The subjects’ genital organs will be protected from X-ray exposure for renal stone.

##### Side effects

The side effects of Pamidronate (Aredia®, Novartis) are the followings:

- The most frequent observed side effects are transient fever (1-2°C), usually 2 days after administration, lasting 24-48 hours and without clinical effect. A flu together with discomfort, fatigue, rigidity and vasomotor reactions are also frequent. Slow infusion (3 hours) of this drug would minimize these side effects.
- Sometimes, its administration can produce:
  - local reactions at the injection site : pain, flush, indurations, swelling, phlebitis,
  - side effects concerning locomotor systems : transient bone pains, muscle, joint or generalized pains,
  - gastro-intestinal effects : nausea, vomiting,
  - effects on central nervous system : headaches,
  - blood effects : lymphocytopenia which does not have a long duration and without any clinical effect.
- Rarely are reported : muscle cramps, abdominal pains, diarrhea or constipation, anorexia, confusion, dizziness, sleep disorders, anemia, leucopenia, hypo or hypertension.

##### Possible inconveniences and discomfort

The subject might feel discomfort from venous blood sampling by needle and timed micturition. The amount of blood collection is no more than minimal risk, and the amount is listed in Table 2 (appendix 2). After the isokinetic muscle testing, the subject might feel fatigue and/or mild muscle soreness.

As the device produces microelectricity, micro-current (50kHz, 800 microampere, far below sensation threshold) flows in the body. However, any adverse effects have been reported and its safety is well established.

Rash (contact dermatitis) by the electrodes and/or electrode paste may happen to some subjects (severity, minimal; probability, low).

### Protocol summary (one page), written on a separate sheet

Objectives, brief protocol description, main medical risks

Objectives

The priority of NASDA is to establish health care program for astronauts, a part of which is the pharmacological countermeasure against bone loss in space. Bisphosphonate, a drug known to inhibit bone resorption will be administered to healthy young male subjects (of the pharmacological group) who will perform 90 days -6 degree head-down tilt bed rest in order to clarify its protective effect on bone metabolism, calcium balance and renal stone formation during immobilization. Back pain during bed rest will also be assessed by questionnaire for the subjects. Additionally, usefulness of a commercially available device to monitor body composition will be studied in order to develop a simple in-flight device (using all three groups).

Brief protocol description

The subjects for the pharmacological group will receive 60 mg of Aredia (Novartis) at BDC-14 via a 3-h infusion.

Fifteen ml of blood samples will be collected, 14 days and 7 days before bed rest, on days 3, 7, 14, 30, 60, 90 during bed rest, and on days 3, 7, 14, 45, 90, 180 and 360 during recovery period. Additionally, 3 ml of blood will be drawn on the first day during bed rest, and one day after bed rest. Total blood volume will be 174 ml during the study period.

24 hours urine samples will be collected before bed rest (from -15 to –13; from –10 to –7; from -4 to –1), during bed rest (from 1 to 7; from 27 to 30; from 57 to 60, from 87 to 90), and after bed rest (from +1 to +7, from +11 to +14) for the parameters listed in table 3 of Appendix 2. Additionally, calcium excretion in 24 hours urine and feces will be measured at the same dates.

Blood, urine and feces samples will be examined for biochemical parameters of bone turnover, calcium homeostasis and renal stone. Abdominal X ray for checking renal stone will be performed before bed rest (during selection) and 3 and 30 days after bed rest period. Bone mineral content of lumbar spine, neck of femur, whole body (including skull), heel and forearm together with percent fat and lean body mass will be measured (ITR-DEXA) with DEXA (Dual Energy X-ray Absorptiometry) before bed rest (during selection and BDC-14), during (30, 60 89) and after bed rest (R+7, +14, +45, +90, +180, +360). Lower leg muscle and lumbar spine will be measured with MRI (Magnetic Resonance Imaging). These Imaging measurements (ITR-NMRI) will be done, 9 days before bed rest and on days 29, 89 during bed rest and on days 14, 90, 180 and 360 during recovery period. Isokinetic muscle strength will be measured by Cybex-Norm dynamometer on the same days as imaging measurements; These measurements (ITR-CYBEX) will be done, 12 and 5 days before bed rest and on days 30, 60, 90 during bed rest and on days 2, 8, 14, 45, 90, 180 and 360 during recovery period.

Additional measurements include evaluation of back pain during bed rest, and body composition by bio-electrical impedance method (see table 1).

Main medical risks

Side effects of the drug

The side effects of Pamidronate (Aredia®, Novartis) are the followings:

- The most frequent observed side effects are transient fever (1-2°C), usually 2 days after administration, lasting 24-48 hours and without clinical effect. A flu together with discomfort, fatigue, rigidity and vasomotor reactions are also frequent. Slow infusion (3 hours) of this drug would minimize these side effects.
- Sometimes, its administration can produce:
  - local reactions at the injection site : pain, flush, indurations, swelling, phlebitis,
  - side effects concerning locomotor systems : transient bone pains, muscle, joint or generalized pains,
  - gastro-intestinal effects : nausea, vomiting,
  - effects on central nervous system : headaches,
  - blood effects : lymphocytopenia which does not have a long duration and without any clinical effect.
- Rarely are reported : muscle cramps, abdominal pains, diarrhea or constipation, anorexia, confusion, dizziness, sleep disorders, anemia, leucopenia, hypo or hypertension.

### Investigators

**Principal Investigator:** H. Ohshima, MD

**Co-investigators:**

Chiharu Sekiguchi, MD (responsible for bio-impedance)

Koh Mizuno, MD (responsible for sleep analyses)

Masamichi Sudoh, PhD (responsible for bio-impedance)

Yukiko Watanabe, MS

INSERER TABLE1

INSERER TABLE2

INSERER TABLE 3

INSERER TABLE 4

## Effects of long duration bed rest on sleep and core body temperature rhythm with and without exercise.

### Scientific Background

#### Problem presentation

Sleep disturbances in space have been commonly reported. The possible reasons for these disturbances are shorter daily schedule cycle than 24 hours, isolated environment, busy work of the mission, and microgravity. Poor sleep can be a cause not only of human error lead to sever accidents, but also of impairment in mood and psychological status. Under the bed rest condition, which is one of simulated situations of microgravity, sleep disturbances have been also reported as longer nocturnal sleep onset latencies and poorer subjective rated sleep.

Physical exercise has been demonstrated to promote improved sleep by the results of epidemiological and experimental studies. However, a few studies examining the effect of physical exercise on sleep during bed rest up to 35 days showed that physical exercise during bed rest has negative effects on sleep. For the work/rest schedule in the International Space Station program, physical exercise as a countermeasure against bone and muscle atrophy is being planned for 5 to 6 days per week. Therefore, it is necessary to reconfirm the effect of exercise countermeasure on sleep by ground-based study (bed rest), although bed rest situation has several different features from the cases of space flight.

#### Literature data including references

Sleep disturbances during space mission have been commonly reported. The reason of sleep disturbances is thought to be a combination of several factors such as the initial excitement of space flight, noise disturbance, the effects of weightlessness, and cramped quarters available for sleep (1). Though some of these factors have been eliminated in later space missions, poor sleep and subsequent fatigue were still recognized as one of the significant problems during space flight (2, 3). In case of space shuttle missions, one of the major factors to deteriorate sleep during space missions is the shorter duration of daily cycle less than 24 hours, in other words, scheduled phase advance of sleep/wake cycle. To eliminate this phase advance effects, Monk et al examined sleep in specially scheduled shuttle mission for minimizing disruptions in circadian rhythms and sleep. In this study, poor sleep characterized by decreased total sleep time and amount of delta sleep were still observed (4). Recently, Czeisler et al examined the effects of melatonin administration as a countermeasure against sleep disturbance and scheduled phase advance during shuttle flight (in STS-90 & 95). The results of this study have not been published yet.

It has also been reported that sleep disturbance accompanied by a loss of motivation and concentration, increased fatigability and sensitivity to physical or emotional stressors are observed during bed rest (5,6,7). This sleep disturbance was characterized by increased daytime napping, longer sleep onset latency at night, and decreased amount of slow wave sleep (8). Campbell (9) demonstrated that usual monophasic sleep ( ～8 hours) was replaced by short fragmented sleep bouts (～8 hours) throughout the 24 hour day under 60 hours bed rest condition without any time cue (Zeitgeber). One possible reason for sleep disturbance at night during bed rest is to have too much daytime napping. In case the subjects’ sleep was scheduled only in night time (having daytime nap was not allowed), only minor problems of longer nocturnal sleep onset latencies and poorer subjectively rated sleep but with no reliable changes in any of the other sleep parameters were observed during 17 days bed rest study (10). Since one of the major influences of bed rest is immobilization and inactivity, decrements in circadian amplitude in body temperature (10), heart rate and urine catecholamines (11) were confirmed during bed rest study.

To our knowledge, there have been only two studies to examine the effect of physical exercise on sleep during bed rest. Since physical exercise has been considered to promote good sleep characterized by longer total sleep time, increased amount of slow wave sleep, and decreased amount of REM sleep (12), it was expected that physical exercise possibly counteracted against sleep disturbance during bed rest. However, the results of those studies did not support the hypothesis that poor sleep during bed rest is recovered by performing physical exercise. Ryback and Lewis measured polysom-nographic recordings during 5 weeks bed rest in two groups, exercise group and control group (13). In this study, no significant effect of exercise was observed, while slow wave sleep in control group increased significantly compared to pre-bed rest value. DeRoshia and Greenleaf examined the effects of two type of exercise, isotonic pedalling and isokinetic exercise, during 30 days bed rest on mood state including subjective sleep sensation (14). Unexpectedly, adverse effect of isotonic pedalling on sleep was shown as a significant increase in subjective feeling of trouble-falling-asleep. Since prescribed exercise intensity (up to 90% of VO2peak), volume (30 min x twice/day), and frequency (6 days/week) was intensive, the authors explained that this result might reflect overtraining or excess total workload.

The potential effect of exercise to shift human circadian phase has been suggested (12, 15). This effect of exercise seems to depend on timing for performing exercise as well as the phase-shifting effect of bright light exposure. There has been no study to examine this effect under bed rest condition. In the study of Ryback and Lewis (13), there was no description regarding timing for performing exercise. And in the study of DeRoshia and Greenleaf (14), 30 minutes exercise was performed twice per day (morning and afternoon).

1. Graeber RC. Sleep in space. In: Roussel B, Jouvet M, editors. Actes du 27 eme seminaire du GRD le sommeil et ses implications militaries. Lyon: A.C.E.M.L. 1988: 59-69.
2. Berry CA. Preliminary clinical report of the medical aspects of Apollos VII and VIII. Aerospace Med 40: 245-254. 1969.
3. Santy PA, Kapanka H, Davis JR, Stewart DF. Analysis of sleep on shuttle missions. Aviat. Space Environ. Med. 59: 1094-1097, 1988.
4. Monk TH, Buysse DJ, Billy BD, Kennedy KS, Willrich LM. Sleep and circadian rhythms in four orbiting astronauts. J Biol Rhythms. 13(3):188-201, 1998.
5. Artishuk VN, Litsov AN, Stupnitskiy VP, and Yakushkov YuV. Effect of 30-day hypokinesia on the dynamics of higher nervous activity and sleep of an operator. Kosm Biol Aviakosm Med 8:75-79, 1974.
6. Myasnikov VI. Characteristics of the sleep of men in simulated space flights. Aviat Space Environ Med 46: 401-408, 1975.
7. Winget CM, DeRoshia CW. Psychosocial and chronophysiological effects of inactivity and immobilization. In: Sandler H, Vernikos J, eds. Inactivity: physiological effects. New York: Academic Press, pp. 123-147, 1986.
8. Ohta T. Sleep-wake rhythm of normal subjects under the entrained, absolute bed-rest condition for one week. Seishin Shinkeigaku Zasshi. 85: 302-30, 1983.
9. Campbell SS. Duration and placement of sleep in a “disentrained” environment. Psycophysiol. 21: 106-113, 1984.
10. Monk TH, Buysse DJ, Billy BD, Kennedy KS, Kupfer DJ. The effects on human sleep and circadian rhythms of 17 days of continuous bedrest in the absence of daylight. Sleep 20:858-64, 1997.
11. Samel A, Wegmann HM, Vejvoda M. Response of the circadian system to 6 degree head-down tilt bed rest. Aviat Space Environ Med. 64: 50-54, 1993.
12. O’connor PJ, Youngstedt SD. Influence of exercise on human sleep. In: Holloszy JO (ed.), Exercise and sport Sciences Reviews Vol. 23. Williams & Willkins, Baltimore, 1995: 105-134.
13. Ryback RS, Lewis OF. Effects of prolonged bed rest on EEG sleep patterns in young healthy volunteers. Electroenceph Clin Neurophysiol 31:395-399, 1971.
14. DeRoshia CW, Greenleaf JE. Performance and mood-state parameters during 30-day 6 degree head down bed rest with exercise training. Aviat Space Environ Med. 64：522-527, 1993
15. Van Reeth O, Sturis J, Byrne MM, Blackman JD, L’Hermite-Beleriaux M, Leproult R, Oliner C, Refetoff S, Turek FW, Van Cauter E. Nocturnal exercise phase delays circadian rhythms of melatonin and thyrotropin in normal men. Am J Physiol 266: E964-974, 1994.

#### Protocol justification according to current scientific knowledge

There have been only two studies to examine the effect of physical exercise on sleep. One study was conducted during 35 days bed rest with the measurement of polysomnography, which was published in 1971, and the other study was conducted during 30 days bed rest with the measurement of subjective questionnaire, which was published in 1993. Since 90 days of bed rest duration is much longer than previous studies, meaningful data accumulation and new findings in this research field are expected.

The objective evaluation of sleep/wake is planned to conduct by Actigraphy, which is to estimate the state of sleep or awake by measuring acceleration of a wrist of none-dominant hand. Although polysomnographic recordings including EEG, EOG, and EMG are necessary to classify sleep stages consisting of stages wake, 1, 2, 3, 4, and REM, continuous measurement for more than several days is difficult due to the skin irritation caused by paste with electrodes, and natural sleep architecture might be influenced by attaching a lot of electrodes. By using Actigraphy, objective prolonged data with minimum stress on the subjects can be obtained. Reliability of this procedure for identifying sleep/wake has been well established (1). Besides, from the results of 17 days bed rest study without physical exercise, reported reliable changes in the sleep parameters were longer nocturnal sleep onset latencies, which can be evaluated by Actigraphy.

In case of normal daily life on the ground, a variety of factors act to mask proper circadian rhythm. One study succeeded in proving the phase shifting effect of exercise on endocrine circadian rhythm was conducted under rigorously controlled situation called “constant routine technique”. As for a rhythm of core body temperature, an elevation in body temperature caused by muscle contractions in routine physical activities is one major masking factor and possibly to affect sleep/wake status. From this point of view, the situation of bed rest ( and microgravity ) may be able to demonstrate the effects of exercise on circadian phase shifting and sleep/wake status more obviously without the masking effects caused by physical activities.

#### The interest of the duration of the head-down bed rest has also to be justified, in particular for bone changes.

The duration of the bed rest planned in this project is longer than that in any previous studies to examine sleep. The present research proposal is not related to bone metabolism.

#### Rationale for drug (if used) and dosage selection

Drug dosage is not concerned with this proposal.

### Objectives and hypothesis

#### Main objective of the protocol

The objective of the present protocol is to determine the effect of long duration bed rest with or without physical exercise on Actigraphically evaluated sleep and core body temperature rhythm. The effects of timing of performing exercise on sleep and core body temperature rhythm is also planned to determine.

#### Identification of main parameters

1. Evaluation of the pre-bed rest characteristics of the subjects related to sleep by questionnaires
2. Continuous sleep/wake evaluation by Actigraphy before, during, and after bed rest
3. 72 hours continuous measurement of rectal temperature before, during, and after bed rest
4. Evaluation of subjective rated sleep every 15 days before, during, and after bed rest

#### Tested hypothesis

- Increased diurnal napping and decreased nocturnal total sleep time would be observed during long duration bed rest without bright light.
- Observed core body temperature rhythm during bed rest would be of lower amplitude and phase-delayed compared with pre-bed rest value.
- Conducting timed physical exercise affects amplitude and phase of core body temperature rhythm and sleep/wake status, possibly counteracting against adverse effects during bed rest.

#### Expected results

- - 1. Sleep of the subjects under long duration bed rest without physical exercise would be fragmented and shorter sleep duration during night would be observed.
    2. Core body temperature rhythm during bed rest without physical exercise would be of lower amplitude and phase delayed.
    3. Performing physical exercise would affect core body temperature to prevent lowering amplitude of body temperature rhythm and to shift phase of body temperature rhythm by changing the timing for performing exercise.

Those data, obtained under such long duration bed rest with or without physical exercise, would be an new findings in the scientific field of sleep and chronobiological physiology and be useful findings for arranging the schedule for physical exercise during long duration space flight in aspect of preventing internal desynchronization.

#### Secondary objectives (if necessary)

None

### Specific inclusion and non-inclusion criteria for test subjects

Inclusion and non-inclusion criteria for test subjects of this research proposal are as follows:

- 1. Subjects who are extremely morning (lark) or evening (owl) type must be excluded. This evaluation is done by the modified standard questionnaire established by Horne and Ostberg.
  2. Subjects who sleep normally less than 5 hours or more than 10 hours must be excluded. This evaluation is done by interview or questionnaire, or if possible, the measurements of Actigraph for one week during selection of the subjects would be done.
  3. Subjects must be free from sleep disorders verified by the Pittsburgh Sleep Quality Index (global sleep disturbance index of less than 5).
  4. Subjects who have recent experience of shiftwork (within 1 year) or travel across time zone more than 5 hours (within 3 month) must be excluded.

### Precise description of the experimental protocol

#### Tests description (methods, duration, time of day, protocol conditions, etc.)

1. Subjects:
   Since we prepare only ten Actigraph, ten subjects consisting 4 subjects of the exercise group and 6 subjects of the control or the pharmacological group will be treated during one bed rest experimental series. Total 16 subjects consisting 8 subjects in the exercise group and 8 subjects in the control and pharmacological group will be analysed.
2. Methods:
   During the bed rest period, the subjects will have constantly-timed meals (breakfast, lunch and dinner), not deviated for 15 minutes. The room light will be switched off from 23h00 to 7h00, and illumination of the light at the face of the subjects must be less than 1000 lux and 10 lux during the lights on and off, respectively. The subjects will be allowed to have nap during daytime. For the subjects in the exercise group, we request the subjects to perform exercise in three different timing (morning: the center of exercise period will be at 10:30; afternoon: the center of exercise period will be at 14:30; and evening: the center of exercise period will be at 18:30), which will be changed every 30 days throughout the bed rest period. For example, one subject will perform exercise in the morning from the beginning to 30^th^ day, in the afternoon from 31^st^ day to 60^th^ day, and in the evening from 61^st^ to 90^th^ day. The order for changing the timing of performing exercise is randomised among the subjects.

Following measurements will be performed:

- - 1. Morningness-Eveningness questionnaire and Pittsburgh Sleep Quality Index will be answered once before bed rest to identify the sleep characteristics of the subjects.
    2. Actigraphy: Wrist activity of the subjects will be measured by the Actigraph to estimate sleep/wake state before, during and after bed rest. The subjects will wear Actigraph on their wrist of none dominant hands continuously from 2 weeks before bed rest to 2 weeks after bed rest. Battery exchange and data retrieving of the Actigraph will be conducted every 2 weeks.
    3. Rectal temperature: 72 hours continuous measurement of rectal temperature will be performed once before and after bed rest, and four times during bed rest. One day with no exercise must be included in this 72 hours measurement for the subjects in the exercise group. Preferable constitution of this 72 hours (3 days) is that two exercise days are followed by one no exercise day. The timing of the measurement after bed rest will be between +5 and +7. The probe will be inserted 10 cm into their rectum, and be temporarily removed when the subjects will evacuate the bowels.
    4. Modified Pittsburgh Sleep Quality Index: Pittsburgh Sleep Quality Index modified to evaluate sleep of past 2 weeks will be answered on each day when rectal temperature measurement will be completed during and after bed rest.

#### Measured parameters

- - - 1. Answers of questionnaire to identify the characteristics related to the subjects’ sleep
      2. Sleep parameters from the results of Actigraphy
      3. Rectal temperature continuously measured for 72 hours at 1minute interval 4) Subjective rated sleep evaluated by modified Pittsburgh Sleep Quality Index consisting of seven component scores.

#### Equipment used - Hardware description (trademark, model, …), specify if experimental equipment is commercially available or self built,

- - - - Software description.
      - Actigraph (AMI, Ultra or Advanced)
      - Interface of Actigraph including an AC adaptor and cable
      - Software for Actigraph (AMI, Act and Action W)
      - Thermometer for measuring rectal temperature including an AC adaptor and a probe (Anritsu, AM-7002)
      - Software for the thermometer (Anritsu, AMS-7006WIN)

#### Description of the test termination criteria of the protocol

### If the subjects would have severe continuous pain or discomfort with anus due to inserting rectal probe, the rectal probe would be removed until recovery.

#### Specific medical monitoring (ECG, blood pressure monitoring, etc.)(if necessary)

None

#### Other specific requirements (dietary, conditions of the experiment sessions, etc.)

The room light will be switched off from 23h00 to 7h00, and illumination of the light at the face of the subjects must be less than 1000 lux and 10 lux during the lights on and off, respectively. The subjects will be allowed to have nap during daytime. For the subjects in exercise group, we request the subjects to perform exercise in three different timing (morning: the center of exercise period will be at 10:30; afternoon: the center of exercise period will be at 14:30; and evening: the center of exercise period will be at 18:30), which will be changed every 30 days throughout the bed rest period. For example, one subject will perform exercise in the morning from the beginning to 30^th^ day, in the afternoon from 31^st^ day to 60^th^ day, and in the evening from 61^st^ to 90^th^ day. The order for changing the timing of performing exercise is randomised among the subjects.

#### Biological samples handling, preservation conditions, transport

No biological sampling will be done.

- Measured parameters

*Not applicable*

- Methods, equipment and place of analysis

*Not applicable*

- Labelling, storage and packaging of the samples

*Not applicable*

#### Data utilisation and analysis, specify

*Parameters analysed*

- Morningness-Eveningness score
- Scores of Pittsburgh Sleep Quality Index : Once before and after bed rest and every 15 days during bed rest
- Sleep parameters calculated by the Actigraph data: Sleep onset latency, Total sleep time per daytime, night time and 24 hours, Sleep Efficiency Index in the night time, and so on
- Parameters from the results of rectal temperature data: Cycle length, Amplitude, and Acrophase of the temperature rhythm

*Statistical tests*

ANOVA will be used to detect the effects of exercise and time course during bed rest. Software used Microsoft Excel and/or some software will be used.

#### Hazards, side effects, possible inconveniences, discomfort and constraints to subjects

The possible discomfort due to wearing Actigraph is skin eruptions or irritations. To prevent this discomfort, the wrist of the subjects who will be susceptible to skin eruptions should be covered with some cotton under the device. If this discomfort would be happened, ointment would be supplied.

Discomfort or pain with mucous membrane around anus might be happened by insertion of the rectal probe to measure 72 hours continuous rectal temperature. To insert the probe smoothly, some plant oil or white vaseline will be prepared. If the subjects would have severe continuous pain or discomfort with anus, the rectal probe would be removed until recovery.

### Protocol summary (one page), written on a separate sheet

*Objectives, brief protocol description, main medical risks*

The objective of this study is to determine the effects of exercise and timing for performing exercise upon sleep and body temperature rhythms during long duration bed rest.

The subjects will consist of totally 8 subjects in exercise group and 12 subjects in control and pharmacological group.

Continuous measurement of Actigraphy from 2 weeks before bed rest to 2 weeks after bed rest through whole the bed rest period will be performed to estimate subjects’ sleep/wake status. Accompanied with this Actigraphy, 72 hours continuous measurement of rectal temperature will be done once before and after bed rest and four times during bed rest to identify the characteristics of core body temperature rhythm. Subjective rated sleep evaluation by using modified Pittsburgh Sleep Quality Index will be done on the day to complete rectal temperature measurement to assess subjective sleep feeling during past 15 days.

During the bed rest period, the room light will be switched off from 23h00 to 7h00, and illumination of the light at the face of the subjects must be less than 1000 lux and 10 lux during the lights on and off, respectively. The subjects will be allowed to have nap during daytime if they will have time. The subjects in exercise group will perform exercise in three different timing (morning: the center of exercise period will be at 10:30; afternoon: the center of exercise period will be at 14:30; and evening: the center of exercise period will be at 18:30), which will be changed every 30 days throughout the bed rest period. For example, one subject will perform exercise in the morning from the beginning to 30^th^ day, in the afternoon from 31^st^ day to 60^th^ day, and in the evening from 61^st^ to 90^th^ day. The order for changing the timing of performing exercise is randomised among the subjects.

Possible medical risks specified in this study are 1) skin eruptions or irritations due to wearing the Actigraph on their wrist, and 2) discomfort with anus due to 72 hours continuous measurement of rectal temperature. Countermeasures for these problems will be prepared.

### Investigators

**Principal investigator :**Hiroshi Ohshima, MD, PhD

**Co-investigator**

Koh Mizuno, PhD (responsible for sleep analyses)

## Bone loss and muscle hyptrophy under head-down tilt and resistance training and bisphoshponates.

### Scientific Background

#### Problem presentation

Both space flight and immobilisation elicit a considerable loss of muscle force which occurs mainly in the lower body. Contrasting with this, Schneider and co-workers found that bone mineral density (BMD) increased at the skull after 9-15 days of space flight (11). Similar findings were repeated by Miyamoto et al. (7) in bed-rest studies with -6° head down tilt.

The finding, that bone loss occurs almost exclusively in the lower extremity has often been ascribed to a reduced force generation: during space flight, the lower extremities undergo severe disuse, whereas the arms are used in almost the same way as on earth. In stead of, or in addition to the former explanation, an alternative hypothesis has been put forward. Basis for this opinion is the observation, that femoral vein ligation inhibits the reduction of bone gain in the hindlimb suspended rat (2). In fact, compared to terrestric conditions, a cranial fluid shift occurs in weightlessness. According to the cited opinion, this may contribute to the bone apposition during space flight and head down tilt.

Another possible argument arises from net Ca++-excretion.. It is undoubted, that bones – besides providing stiffness – are important in Ca++-homeostasis of the milieu interieur (5). Thus, by an unknown mechanism, Ca++ might be stored in certain places (e.g. the skull) during removal from other places.

Modelling adapts bones to increased forces, as e.g. after re-entry into the gravitational field. During adulthood in lamellar bone, modelling (i.e. apposition) takes place predominantly at the periostal surface, whereas remodelling (removal) is endosteal. The general observation thus is that with increasing age, long bones expand their outer circumference, while preserving or decreasing the bone cross sectional area (3). Thus, loss and re-gain of bone mineral (during and after space flight ) in the long bones will accelerate the physiological effects of aging (10). An analogous effect pertains to cancellous bone: Any trabeculum, that has been damaged without chance of repair will be removed and its loss is unrecoverable (8). Subsequent losses and gains of bone mineral in spongy bones thus lead to a reduced number of trabeculi and a rarefication of the network, even if bone mineral content returns to its previous value.

#### Literature data including references

The bone loss in the lower extremity amounts to about -10% and seems to cease after 120 days (9) (for survey see van Loon et al. (13)). The increase of bone mineral density (BMD) at the skull seems to be about 3% after 9-15 days of space flight (11), as measured with the Hologic QDR-2000.

Regarding countermeasures, resistance training attenuates muscular atrophy in hind limb suspended rats (6), and it maintains skeletal muscle protein synthesis in simulated weightlessness in man (4). It seems probably, hence, that lower extremity bone loss can be attenuated, if not prevented by resistance training.

A model calculation shows, that in the os calcis, a loss of 5% with in 14 days seems to be realistic (12). The skeleton of the lower body contains 500 g Ca++. A loss of 5% thus amounts to 25 g. The maximum Ca++ excretion rate of the human kidney, however, is only 540 mg per day, and still 100 mg Ca++ are resorbed via the guts (1). The increase of skull BMD thus might be very well be due to an increased storage of Ca++.

References

1. Agus, Z.S. and S. Goldfarb. Renal regulation of calcium balance. In: ***The kidney, physiology and pathophysiology***, edited by D.W. Seldin and G. Giebisch. New York: Raven Press, 1985, p. 1323-1336.

2. Bergula, AP, W Huang, and JA Frangos. Femoral vein ligation increases bone mass in the hindlimb suspended rat [In Process Citation]. *Bone* 24: 171-177, 1999.

3. Epker, BN and HM Frost. Periosteal appositional bone growth from age two to age seventy in man. A tetracycline evaluation. *Anat Rec* 154: 573-577, 1966.

4. Ferrando, AA, KD Tipton, MM Bamman, and RR Wolfe. Resistance exercise maintains skeletal muscle protein synthesis during bed rest. *J Appl Physiol* 82: 807-810, 1997.

5. Heaney, R.P. Calcium. In: *Principles of bone biology*, edited by J.P. Bilezikian, L.G. Raisz, and G.A. Rodan. San Diego: Academic Press, 1996,

6. Linderman, JK, KL Gosselink, FW Booth, VR Mukku, and RE Grindeland. Resistance exercise and growth hormone as countermeasures for skeletal muscle atrophy in hindlimb-suspended rats. *Am J Physiol* 267: R365-R3711994.

7. Miyamoto, A, T Shigematsu, T Fukinaga, K Kawakami, C Mukai, and C Sekiguchi. Medical baseline data collectioon on bone and muscle change with space flight. *Bone* 22: 79S-82S, 1998.

8. Mosekilde, L. Consequences of the remodelling process for vertebral trabecular bone structure: a scanning electron microscopy study (uncoupling of unloaded structures). *Bone Miner* 10: 13-35, 1990.

9. Organov, V, A Rakhmanov, VE Novikov, ST Zatsepin, SS Rodionova, and C Cann. The state of human bone tissue during space flight. *Acta Astronautica* 23: 129-133, 1991.

10. Rittweger, J. and Kirsch, K. Bone and muscle - aging and space. 20. 1999. Annual International Gravitational Physiology Meeting. 10-6-1999. (GENERIC) Ref Type: Conference Proceeding

11. Schneider, V., V. Organov, A. LeBlanc, A. Rakhmanov, A. Bakulin, A. Grigoriev, and L. Varonin. Space flight bone loss and change in fat and lean body mass. *J Bone Miner Res* 117: S1221992.(Abstract)

12. Tilton, FE, JJC Degioanni, and VS Schneider. Long-term follow-up of Skylab bone demineralization. *Aviation Space and Environmental Medicine* 51: 1209-1213, 1980.

13. van Loon, J.J.W.A., J.P. Veldhuijzen, and E.H. Burger. Bone and space flight: an overview. In: *Biological and medical research in space*, edited by D. Moore, P. Bie, and H. Oser. Berlin: Springer, 1996, p. 259-299.

#### Protocol justification according to current scientific knowledge

The problem of muscle weakness and bone loss is one of the major obstacles to manned space flight. While frailty upon return to the gravitational field of Earth might be considered a passagere nuisance, it will put severe problems in mission to other planets. The present protocol tests hypotheses, relevant for effective countermeasures.

Loss of muscle and bone function, however, does not occur exclusively after space flight , but also during immobilisation, a condition much more 'earthbound' and common. In this respect, the present protocol will yield beneficial knowledge for many injured and elderly, since from a patho-physiological point of view, the mechanisms of immobilization and its countermeasures can more easily be investigated in young and otherwise healthy subjects.

The protocol is based on the current scientific knowledge. All interventions and tests have been intensively studied in other fields of application. The scientific team, in particular the MEDES staff has extensive expertise in the management of mid and long term head-down tilt. The flywheel training principle has been evaluated in numerous subjects by Dr. Tesch. The test procedures foreseen are either clinical routine (blood samples, CT, DXA, MRI) or without recognizable risk (ultrasound, Body Impedance, injection of stable, non-radioactive Ca^++^-isotopes, jump test), both with validated outcome.

Finally, and most important, there is no permanent damage or disadvantage expected for the subjects, neither for the treatment groups nor for the control group. Hence, the protocol's relevance for mankind and the humans justifies the expected minor risks.

#### The interest of the duration of the head-down bed rest

Bones adapt to the forces they experience. This process of adaptation, however, takes its time. Loss of bone mineral due to immobilisation, and probably also due to space flight , seems to slow down after 6-8 weeks and probably ceases after 90-120 days. In consequence, if the effects of long-term space missions (> 1 year) on bone are to be simulated, 90 days is the least duration of the experiment. The re-gain of bone mineral after re-entry to earth or after remobilisation takes even longer. Clinical experience, but also investigations of astronauts after space flight demonstrate that this requires months or even years. This again demonstrates the necessity to develop efficient countermeasures for inter-planetar missions.

Bed-rest causes immobilization of the lower limbs, which occurs during space flight . The circulatory effects of microgravity impose additional requirements. Under terrestrial conditions, the only ways to simulate the reported fluid shifts is by water immersion or by 6% head down tilt. The hypotheses to be tested try to quantify the respective combined effects of immobilization and of the cranial fluid shift. Since immersion for 90 days is not practicable, 6% head down tilt for 90 days is the way of choice to simulate long-term effects of microgravity.

#### Rationale for drug (if used) and dosage selection

Bisphosphonates are the most effective anti-resorptive drug to reduce bone loss in

- Post-menopausal osteoporosis
- Glucocorticoid induced osteoporosis, and
- Paget’s disease

In the lack of vaildated intravenous applications for the prevention of bone loss, an oral application of bisphosphonates is inevitable. The choice of the drug which will be used in the experiment is described in Dr. Ohshima's protocol.

### Objectives and hypothesis

#### Main objective of the protocol

It is the main objective of the protocol to analyse simultaneously the effects of immobilisation and head down tilt, and to test which of the proposed countermeasures solves which of the expected problems. The latter consist in the application of a well established type of high resistance training and in the ingestion of a bone anti-resorptive drug in different groups of subjects. Hence, the present protocol in first instance promotes the development of specific countermeasures for space flight adversive effects, and in second instance it aims at the clarification of the pathophysiological causes of these negative effects.

#### Identification of main parameters

The protocol focusses on those parts of the muskulo-skeletal system that undergo the greatest changes, i.e. the bones of the lower leg and particularly the os calcis (for bone loss) and the skull (for bone gain).

The main parameters to be assessed are

1. Muscle size & function:The method of choice for the assessment of muscle size is MRI. Cross section images shall be taken from the calf muscle to assess the muscles cross-sectional areas and volume. Muscle function of the calf muscles will be tested during a standardized procedure of vertical jumps on a ground reaction platform. Parameters of interest are maximum force and power, both during eccentric and concentric contraction.
2. Bone mineral content & structureThe method of choice to assess content *and* structure is quantitative computer tomography (QCT). QCT scans shall be obtained from the bones of primary interest (lower calf) and the skull. Numerical estimation of the trabecular meshwork shall be performed on basis of conventional x-ray images of the os calcis. As a control site, the distal forearm of the non-dominant hand shall also be investigated by QCT. For the control bone sites, and for the os calcis, in which it is difficult to define a standard section level, the bone mineral content will be assessed by dual x-ray adsorptiometry (DXA). The lumbar spine as another control site shall also be measured by DXA.
3. Fluid volume shiftMeasurements of the relative body composition (total body water, lean body mass, bdoy fat) will be carried out with the non-invasive body impedance method according Baumgartner (1988) applying the Body Impedance Analyser (BIA), Data Input (Frankfurt a. M., Germany). On the days of measurement, the total body weight shall be assessed at 10 a.m. The fluid distribution in the superficial tissues will be determined by a non-invasive ultrasound equipment which has frequently been used before (Kirsch et al. 1993; Gunga et al. 1994, 1995). For the measurements, an ultrasonic pulse echo equipment will be used (CL3DL Krautkrنmer and Co, Cologne, Germany). The hormonal responses of the organism to fluid redistribution and changes in vascularisation shall be monitored by the measurement of the vascular endothelial growth factor (VEGF), erythropoietin (EPO), and thrombopoietin (TPO).
4. Calcium metabolism & hormonal controlCalcium absorption from the bowels, calcium removal from and deposition to the bone shall be assessed by the double-labeling technique: one stable (non-radioactive) calcium isotope is injected intravenously, another is ingested orally. Ca-diet and excretion via the kidney must be assessed during the day of test. In total, three tests are to be performed: control, the expected day of maximum removal (~HDT8) and of maximum bone apposition (~R14).

#### Tested hypothesis

It is the objective of the proposed project to test a set of hypotheses, relative to the items which have been detailed above.

#### Expected results

Hypothesis 1: It is expected, that resistance training will attenuate or even prevent the loss of muscular mass and function. In particular, the calf muscles cross sectional area and volume as assessed by MRI will be unchanged by bed-rest in the exercise group, but will undergo a consistent reduction in the control and the bisphosphonate group. These changes will recover within 90 days after termination of bed-rest.

Hypothesis 2: It is expected, that application of bisphosphonates will reduce or prevent the loss of bone mineral. Hence, bone mineral content and structure of the lower limb will not change in the exercise and bisphosphonate groups, but will depict a significant reduction and deterioration during bed-rest in the control group. These changes will recover after 360 days after termination of bed-rest. No changes are expected at the distal forearm and at the lumbar spine.

Hypothesis 3: The effects of immobilisation / head down tilt, assessed on HDT8 will be ass follows: Calcium excretion will reach the kidney's saturation level (500 mg/day) only in the control group. Net removal from the bone will increase significantly and exceed the likewise increased Ca^++^ deposition in the bone. The enteral calcium absorption will be significantly depressed.After termination of bed-rest, the effects detailed above will be reverted in the control group, with near maximum enteral absorption (700 mg/day) and minimum renal calcium excretion. PTH levels will be low during bed-rest, and elevated during recovery.

Hypothesis 4: It is expected, that the loss of bone mineral in the diaphyses of the tibia in the control group will be mainly on the endosteal surface. Hence, at the termination of bed-rest an increase in inner diameter without changes of the outer diameter is expected. After recovery, bone formation will take place mainly on the periostal surface, i.e. an increase in outer diameter is expected, without changes of the endosteal diameter.

Hypothesis 5: It is expected, that the loss of bone mineral in the spongy bones of the lower leg in the control group will reduce the number of trabeculi. Hence, at the termination of bed-rest, a rarefication of the trabecular network in the distal tibia and the os calcis is expected. This rarefication will correlate with the decrease of bone mineral content. During recovery, the number of trabeculi will be unchanged, whereas bone mineral content will reach its original value within 360 days.

### Specific inclusion and non-inclusion criteria for test subjects

Subjects to be included must fulfil the following criteria:

male

aged between 25 and 40

physical activity 1-2 times / week for at least 30 minutes; no top athletes (1st or 2^nd^ league in their sports)

normal kidney function (checked by serum creatinine or preferentially creatinine clearance).

Volunteers can not participate if they

are weightlifters, body builders etc

have had a fracture within the last 24 months,

have had a rupture of a tendon, ligament or muscle fiber within the last 12 months,

have a metal implant in their body,

have an artificial cardiac pacemaker.

### Precise description of the experimental protocol

#### Tests description (methods, duration, time of day, protocol conditions, etc.)

1. Vertical jumps:The subjects stand on a force plate (without shoes and stockings). Two electrodes are applied with 2 cm distance to the center of the vastus lateralis, tibialis anterior, and lateral gastrocnemius muscles of the non-dominant leg. Two additional electrodes are placed to record the ECG. The subjects' position is upright, with the hands attached to the hips. After 10 seconds of standing still, the subjects consecutively perform three vertical jumps, the hands keeping on the hips. The tentative is to jump as high as possible (first priority) and to keep the contact time as brief as possible (second priority). At the end of the test, the subject has to stand still for another 10 seconds. If not indicated else in the protocol, this test is performed between 10 and 12 a.m.. The force plate will be calibrated before and after each recording session with a 20 kg mass.
2. MRI of the non-dominant lower leg:The adjustment of the MR device, the positioning of the subject, archiving of image data, evaluation of scans, documentation of results, and the transfer of data will documented in Table 5.
3. Radiograph os calcis:The realization of calcaneus X-ray images is performed following a standardized procedure :The focal calcaneus distance is settled at 1m. Calcaneus is placed on contact with the cassette. X-ray tube voltage is fixed at 46 kV. As absorption and diffusion phenomena are dependent on X-ray tube voltage, this parameter is fixed to have the same characteristics of optical density on the films. Exposition conditions are fixed to 16 mA.s. Kodak monocoated films are used. The screen and the film are put in a cassette which permits to maintain the contact between the screen and the film. The screen is used as an amplifier and thus improves the film sensibility. MIN-RG Kodak films of 18×24 cm size are used. These films are usually devoted to mammography.Development is automatically realized with the same equipment at 34° in a developing bath. Utilization of an aluminium standard scale form. This scale, supplied by our center, must be radiographed just after (or just beside) calcaneus in order to normalize the grey scale and to avoid grossly bad exposures. To have a sufficient resolution, the scale has 16 steps. With the X-ray voltage used and the aluminium scale, the step height must be of 1.25 mm.A standard sight of spatial resolution is used. This sight, supplied by our center, must be radiographed just after calcaneus in order to check the modulation transfer function of the films. These function characterizes the system capacity to reproduce the small details. It is the attenuation contrast measure in relation with the spatial frequency. In order to assess the measure variability, the first three patients must have two calcaneus radiographs in a delay of 1 hour.Radiographs are numerized with an Agfa Duoscan scanner. The scanner resolution is fixed to 100 µm. Options like brightness, contrast, colour and optical density will be inactivated. The film and the scanner are considered as a single system. This system is modelized with three characteristics : grey level curve, attenuation contrast and background noise.The region of interest (ROI) is defined by marking anatomic points that can easily be identified on the calcaneus image. This ROI of 2.7×2.7 cm is located in a part of the calcaneus that contains only trabecular bone. The ROI is numerized with 256 grey levels. The histogram of the grey levels allows us to distinguish bad and good radiographs. A reproducibility study showed that a small shift of the ROI in horizontal and vertical directions weakly influence the fractal analysis results.
4. pQCT of non-dominant calf and of non-dominant forearm:The dominant arm is the side, that the subject uses for writing. The dominant leg is the leg, that the subject uses intuitively for jumping.During the first investigation, the length of the tibia will be assessed as the distance between the medial malleolus and the medial joint cleft of the knee. Next, the subjects will be seated in the pQCT device with the tibia positioned horizontally. The foot will be fixed, with the tibio-talar joint positioned to an angle of 120°. In total, four pQCT section images will be obtained from the leg:1) The first three sections will be at 4%, 14% and 33% of the tibia length, starting from the tibio-talar joint. The tibio-talar joint cleft will be identified by scout-viewing. Images will be taken with a voxel size of 0.4 mm (parameter voxel size in the integrated software) in the transverse direction and 2.4 mm in the longitudinal (x-ray beam width). The CT speed will be set to 20 mm/s.2) The fourth section will be at 66% of the tibia length. The medial joint cleft will be identified in scout-viewing mode. 34% of the tibia length distal from the cleft a cross section image will be obtained with voxel size of 0.8 mm and a CT speed of 20 mm/s.3) As a control bone site, the distal forearm will be measured at 4% and 60% levels of the ulna. The length of the ulna will be measured in the first investigation as the distance between the distal joint cleft and the processus styloideus ulnae. The hand will be positioned on the integrated fixation device, with the arm extended in the elbow. The distal joint will be searched in scout-viewing, and the reference line will be defined semi-automatically by the integrated software. Both images of the arm will be taken with a voxel size of 0.4 mm and a CT speed of 20 mm/s.**Important**: All images will be evaluated for movement artefacts while the subject is still positioned. If movement artefacts are present (recognised by reddisch blurs), the contaminated sections must be repeated.
5. QCT of the skull:The adjustment of the CT device, the positioning of the subject, archiving of image data, evaluation of scans, documentation of results, and the transfer of data will documented in Table 5.
6. DXA os calcis and lumbar spine:Measurements of the os calcis and the lumbar spine (L1-L4) will be according to the manufacturer's recommendations. For the lumbar spine, the subjects are in supine position, with a support of the lower legs so that the upper legs are vertical. The vertebral column is posed in the center of the measurement in straight position. For the sake of orientation, scanning will be from the edge of the os sacrum to the 12^th^ rib.
7. Double labeling with stable Ca^++^ isotopes & 24 hour urine collection :Starting at least 3 days prior to the experiment and lasting 5 days after it, the alimentary Ca^++^ diet is adjusted between 600 and 800 mg / day. The experiment starts at 8.00 a.m., when at first the urine bladder is voided and urine collection is started in 8 hour portions for 5 days. Then the baseline blood sample (sample No 0) is drawn (2 ml), and 50 mg of tracer1 are injected intravenously in 2 minutes. Subsequent samples (sample No 1- 5) are drawn exactly 1, 2, 4, 8, and 12 hours after termination of the tracer injection. Immediately after the injection, the subjects have a standard breakfast, comprising 2 rolls, 10 g butter, 20 g marmelade, and 2 cups of coffee or tea. The second label, tracer2, will be mixed in the last half of the first cup of beverage. The next meal will not be served before 1 p.m.. In the meantime, the subjects may drink water ad libitum.The exact dosages will be detailed in Amendment 1.
8. Body Impedance Analysis & tissue thickness:The Body Impedance Analyser operates with four electrodes: two electrodes have to be placed at the right hand and two others at the right foot. The local fluid distribution in the superficial tissues of the pretibial region (50% of tibia length) and of the front (between the two eyebrows) will determined by a non-invasive ultrasound equipment. These analyses are performed in supine position between 8 a.m and 10 a.m. At the same time the body weight of the subjects has to be assessed.

#### Measured parameters

1. Vertical jumps:Measured parameters of primary interest are the maximum power and force in the eccentric and concentric phases of jumping. In addition, the following parameters will be obtained to guarantee the quality of the recording: range of motion, height of maximum power, electro-motor delay times, heart rate response.
2. MRI of the non-dominant lower leg:Primary outcome parameters are the volume of the muscles at the lower leg, and the cross sectional area at 66% of the tibia length, measured from the tibio-talar joint.
3. Radiograph os calcis:To take into account the anisotropy of the bone architecture transposed on the texture of the trabecular bone image, we perform an oriented texture analysis. For each direction studied, we analyse several lines with the same orientation. The result in one direction is the average of the values of each line. This unidirectional evaluation is repeated in 36 directions. The results are transferred onto a polar diagram to portray a visual representation of the anisotropy of the texture. The final result for an image, the $H$ mean, is the mean value of $H$ obtained by averaging the results of the 36 directions. The $H$ mean values represents this final unique figured result for each image.
4. pQCT measurements:In the integrated software of the XCT2000, the measures of bone mineral density are normalized to the density of fat tissue. Accordingly, the density of muscle tissue is about 70 mg/cm³, whereas completely mineralised bone has a density of about 1200 mg/cm³.In the pQCT images, regions of Interest (ROIs) for the bones (tibia, fibula, radius, ulna) will be defined semi-automatically by the software. In all sections and bones, the bone mineral content and the perimeter will be obtained with the separation procedure in contour mode 1 and the threshold set to 710 mg/cm³. For calculation of the area moment of resistance, the threshold will be set to 280. The latter calculation will not be performed in the 4%-level sections.For the assessment of the muscle CSA (at 66% of tibia length), the ROI will be defined within the subcutaneous tissue, but outside the muscle. Within this ROI, the total area of the muscle will be determined with the threshold set at 35 mg/cm3 (contour-mode 1). Next, the total areas of the fibula and the tibia, assessed as above, will be subtracted. The section of the 4% level of the non-dominant forearm will be analysed in the same way as the 4% level section of the calf, and the 60% forearm section will be analysed like the 66% calf section.
5. QCT of the skull:Measurements include bone mineral density (mg /cm³), bone mineral content and bone thickness in different parts of the skull. The evaluation will be performed by a computer programme that has been manufactured by the Institute of Medical Physics, Erlangen University.
6. DXA os calcis and lumbar spine:For the definition of the region of interest (ROI), the broadth will be adopted from the manufacturer's recommendations, while the length will be adjusted manually, so that the upper part of the os sacrum and the lower part of the 12^th^ rib is included.Primary parameters of interest are the bone mineral content and the bone mineral density of L2.
7. Double labeling with stable Ca^++^ isotopes & 24 hour urine collection:In all blood samples (6 per test) and 8 hr urine collections (15 per test) obtained, the total calcium will be assessed after precipitation with ammonium oxalate by atomic absorption spectrometry. The intra-venous and oral tracer excess of all blood and urine samples will be assessed by thermal mass ionisation spectrometry (TIMS). The kinetics of the data will be fitted to a 3-compartment model. From these data, the true fractional absorption α and the Ca^++^ flow to the bone compartment, V_o+_ and from the bone compartment V_o-_ will be calculated.
8. Body Impedance Analysis & Tissue thickness:The Body Impedance Analyser yields the relative portions of water, fat and lean tissue. Multiplication will yield the body composition according to total body water, fat mass and lean body mass.

#### Equipment used

1. Vertical jump measurements:The jumps will be performed on a 'Leonardo' force plate (Novotec, Pforzheim, Germany). The ground reaction forces of the left and right will be recorded digitally using a PowerLab & Chart 4.0 software connected to the SCSI interface of a Personal computer. From the recordings, the heart rate response and the electro-motor delay times can be measured in the Chart software. The other parameters will be extracted by a self-manufactured programme (JumpAn).
2. MRI measurements:1.0 Tesla machine, as existing in Rangeuil University hospital
3. Radiographs of os calcis:Any x-ray machine available in Rangeuil University hospital, as long as it is the same for all radiographs.
4. pQCT measurements:The pQCT measurements will be obtained using the XCT2000 machine (Stratec, Pforzheim, Germany) with the standard positioning device for foot and hand. Analyses will be performed with the actual software version (5.40 or higher) pertaining to the XCT2000.
5. QCT of the skull:The measurments shall be obtained with the Siemens Somatom Plus 4 machine, that is existing in Rangeuil University Hospital.
6. DXA measurements:The measurements shall be performed with a machine, that MEDES will have installed by the beginning of the study.
7. Double labeling with stable Ca^++^ isotopes & 24 hour urine collectionTracers will be obtained from Oak Ridge National Laboratory (Oak Ridge, TN, USA) as calcium carbonate and converted to calcium chloride by the Rangueil University Hospital Pharmacy. Tests for pyrogenity and sterility will be performed before use. Atomic absorption spectroscopy will be conveyed with a Beckham Klina Flame. Thermal ionisation mass spectroscopy will be performed with a Finigan MAT Thermion (Bremen, Germany). Non-linear fitting of the tracer excess will be conveyed with the Revol software, developped and described by 'Berlin' colleagues (Koeppe P, Hamann C: A program for nonlinear regression analysis to be used on desktop computers. Comput. Progr. Biomed. (12) 1980:121-128).
8. Body impedance analysis:Measurements of body composition (total body water, lean body mass, bdoy fat) will be carried out with the non-invasive body impedance method according Baumgartner (1988) applying the Body Impedance Analyser (BIA), Data Input (Frankfurt a. M., Germany).
9. Ttissue thickness:An ultrasonic pulse echo equipment will be used (CL3DL Krautkrنmer and Co, Cologne, Germany). The CL3DL operates on 10 MHz. The probe (0 = 1.0 cm) transmitts a brief burst of ultrasonic energy that propagates through different materials and is received by the same probe (A-mode). The probe is fitted into a teflon ring which stabilizes the system. The weight of the probe and the teflon ring together is 6.5 g.

#### Description of the test termination criteria of the protocol

The only test / investigation in which conditions may occur that will cause a termination is the jumping test. All other investigations are without any perceivable risk or active contribution of the subject.

Hence, the following criteria for the termination of the jump test are defined:

Whenever the subjects feel dizzy or are afraid to fall, they may skip the test. Dizziness might for instance be reported during the 10 seconds of standing still before jumping. Then, the subject is free to perform the test or not. They should, however, be aware that astronauts may be confronted with a similar task upon re-entry into a gravitational field. If the subjects struggle in the course of the jumping test, and hence the recorded signal does not fulfil the standard criteria (see above), the subjects are free to repeat the test at maximum twice.

#### Specific medical monitoring (ECG, blood pressure monitoring, etc.)

- not applicable -

#### Other specific requirements (dietary, conditions of the experiment sessions, etc.)

1. substitution of 400 international units Vitamin D3 per day
2. calcium intake of 800 to 1000 mg per day

#### Biological samples handling, preservation conditions, transport

##### Measured parameters

1. Erythropoietin (EPO),
2. Thrombopoietin (TPO),
3. Vascular endothelial growth factor (VEGF)
4. Parathyrin (PTH),
5. Alkaline phosphatase (AP),
6. N-terminal cross links (NTX),
7. Blood samples taken for double-labeling of Ca^++^-compartments

##### Methods, equipment and place of analysis

1. EPO: Commercial ELISA kits calibrated against the WHO standard preparation of EPO will be used for assay of EPO from IBL (Hamburg, Germany). The analysis will be performed in Berlin.
2. *TPO:* Commercial ELISA kits calibrated against the WHO standard preparation of TPO will be used for assay of TPO from IBL (Hamburg, Germany). The analysis will be performed in Berlin.
3. *VEGF:* The concentration of serum immunoreactive VEGF_165_ will be measured by enzyme-linked immunoassay (ELISA; R&D Systems, Minneapolis, MN, USA). Antibodies are directed against human recombinant VEGF_165_. The immobilized antibody is monoclonal, while the second horseradish peroxidase-coupled antibody is polyclonal. The intra- and interassay coefficients of variance have been determined to be 5% and 7.5%, respectively, and the lower detection limit to be 9 pg/ml in the laboratory of one of the authors (Heits et al., 1997). In healthy adult Caucasian persons, serum VEGF amounts to 239 pg.ml^-1^ (± 163, N=60). The analysis will be performed in Berlin.
4. Parathyrin (PTH). The analysis will be performed in Berlin.
5. Alkaline phosphatase (AP), photometrical measuremnt; the analysis will be performed in Berlin.
6. N-terminal cross links (NTX), ELISA, The analysis will be performed in Berlin.
7. Blood samples taken for double-labeling of Ca^++^-compartments. The analysis will be performed in Berlin.

##### Labelling, storage and packaging of the samples

Blood samples of 8 ml shall be drawn for the analysis of parameters a) - f). The tube shall be labeled as follows:

Blood Sample (Hormones) OsResBis.<SubjectNumber>.<StudyDay>Date

Subject Number is the Randomization-Number of the subject, StudyDay is the day within the study, e.g. HDT3, Date is the actual date.

For the double labeling of the calcium compartments, 6 blood samples with 2 ml plus 5 days of 8 hour-wise urine collection is required. The blood sample shall be stored in Ca++-free tubes, labeled as follows:

Blood Sample (Ca double labeling) OsResBis.<SubjectNumber>.<StudyDay>.<SampleNo>Date, time

SampleNo is the running sample number, i.e. 1 for the first sample of that day and 6 for the last. Time is the time at wich the sample is drawn.

The volume of the 8 h urine collections shall be assessed, and 10 ml samples shall be stored in a Ca++-free tube, labeled as follows:

Urine sample (Ca double labeling)
OsResBis.<SubjectNumber>.<StudyDay>>.<SampleNo>
Date, time

#### Data utilisation and analysis, specify

##### Parameters analysed

Based on the parameters detailed under 6.3.2.2 and 6.34.2, effects of head-down tilt and immobilisation will be tested between the treatment and control groups, according to the hypotheses specified above. In brief, these are: muscle size and function (force & power), bone mass & structure in the heel and skull, and Ca^++^-metabolism.

##### Statistical tests

For all parameters tested, the MANOVA or the t-test will be applied if the assumption of a normal distribution is justified, and the Mann Whitney or Wilcoxon test if not. The questions put to the data are two-fold: 1) Is there a reduction (or increase) of the parameter during the head down tilt period, and does it recover? AND 2) Is there a difference between the treatment groups and the control group?

The first question will be put within the groups, the second between the groups. Hence, for the first question the paired t-test or Wilcoxon test will be performed (with Bonferroni's correction for multiple comparison), and for the second question, the MANOVA procedure or the Mann-Whitney test will be conveyed.

##### Software used

Analyses will be performed with the SPSS/PC software.

#### Hazards, side effects, possible inconveniences, discomfort and constraints to subjects

Hazards or adverse effects arise from the following interventions:

1. Blood samples / infusion of stable Ca^++^ isotopes. With every venous puncture or venous injection, there is a minor risk of bleeding, hematoma or, very scarcely, of infection. Upon injection of Ca^++^, there may be a short sensation of heat, which is not dangerous at all. In some very rare instances, cardiac arrhythmias may occur. It is excluded that these potential arrhythmias are threatening, because the Ca^++^ dose injected is very low.
2. During the jumping test, subjects may faint, particularly in the first days of recovery after head down tilt. The risk in this circumstance is minimised if the subjects skip the test when they feel dizzy. In some very rare instances, the subjects may struggle and damage their ankle.
3. MRI-imaging is without any known risk or inconvenience (if the exclusion criteria are considered: metal implants and cardiac pacemaker).
4. The body impedance analysis is without any known risk or inconvenience.
5. The ultrasonic measurements are without any known risk or inconvenience.

The radiological measurements imply exposition to radiation. The dosage amounts is summarised in chapter 5.

### Protocol summary

During space flight, bone mass and muscle function decay, particularly in the lower part of the body, while fluid and (to some extent) bone mass seem to be shifted to the upper half. At present, these changes are a major obstacle for manned space flight .

The present protocol promotes the development of specific countermeasures for space flight adversive effects. Moreover, it aims at the clarification of the patho-physiological causes of these negative effects, which play an important role in immobilised patients.

The protocol is designed to analyse simultaneously the effects of immobilisation and head down tilt, and to test which of the proposed countermeasures. The latter consist in the application of a well established type of high resistance training and in the ingestion of a bone anti-resorptive drug in different groups of subjects.

In order to simulate these effects under well controlled, terrestrial conditions with complete medical service, -6% head down tilt is performed in 28 male healthy volunteers between 25 and 40 years of age is performed for 90 days, with additional 14 days before and after the head down tilt, dedicated for medical assessment.

The parameters of interest are

1. the muscles' size, assessed by MRI, and force and power, assessed on a force plate in a standardised jumping protocol,
2. the bone mineral content and bone structure of heel, leg, lumbar spine, skull, and arm, assessed by quantitative computer tomography, dual energy adsiorptiometry and conventional radiographs,
3. the cranial fluid volume shift and its related hormonal changes, assessed by ultrasound and body impedance methods, and ELISA on blood samples
4. the calcium metabolism, assessed by intravenous and oral labeling with stable (non radioactive) isotopes, and the related hormonal changes.

It is expected, that the resistance training will preserve muscle and bone mass and structure as it is at the beginning of head down tilt. Drug application preserve bone alone, but will have no effect on muscle. The control group will have the well known effects in muscle and bone, known from own experience and reported in the literature. These effects will be reversed 6-12 months after head down tilt. The present protocol, hence, allows also the study of re-conditioning of the muskulo-skeletal system.

### Investigators

Principal Investigator : Dieter Felsenberg, Prof.

Co-Investigators

Claude Laurent Benhamou, Prof.

Hans Friedrichsen, Prof

Michael Giehl, Dr.

Hann-Christian Gunga, PhD.

Karl A. Kirsch, Prof.

Per A. Tesch, PhD

Joِrn Rittweger, Dr.

## Effects of resistance exercise, using fly-wheel technology, on muscle size and function in healthy bedridden subjects.

### Scientific Background

#### Problem presentation

Skeletal muscle function is compromised in response to long-duration orbital flight. Hence, in-flight methods to counteract this effect are warranted. We have developed a mechanical, non-gravity dependent exercise system, which uses the inertia of fly-wheels, to produce resistance. It meets NASA requirements of a resistive exercise device designed for use on the International Space Station (ISS).

This project will study the efficacy of this exercise system to prevent muscle atrophy and impairment of muscle function which occur during spaceflight. In an effort to simulate weightlessness, healthy men will perform 90 days bedrest. Ten subjects will not train (BR), whereas ten volunteers will perform bilateral resistance exercise (BRE), on the ergometer 2-3 times weekly. Each session consists of four sets of 7 maximal concentric-eccentric knee extensor muscle actions. Before and after BR and BRE the cross-sectional area of individual knee extensor muscles will be measured using magnetic resonance imaging (MRI). In muscle biopsies, obtained from the vastus lateralis and soleus muscles, myosin heavy chain composition and enzyme activities will be assessed. Also, muscle force and electromyographic activity, will be measured during submaximal or maximal knee extensions to assess several aspects of muscle function.

Thus, we should be able to study the effects of resistance training on size, integrity and function of skeletal muscle subjected to a spaceflight analog. If the exercise countermeasure paradigm proves to be effective in preventing muscle atrophy and impaired muscle function in response to 90 days bedrest, we propose fly-wheel technology to be used as an exercise countermeasure on the ISS.

Another purpose is to study the effects of bedrest alone and in combination with resistance exercise on vascular distensibility of the arteries and arterioles of the upper and lower extremity. Orthostatic tolerance is a significant problem after prolonged bedrest and spaceflight, and arterial or arteriolar distensibility may play a role in orthostatic tolerance. It appears reasonable to assume the proposed bedrest regimen will increase arterial/arteriolar distensibility. Resistance exercise might off-set this effect.

#### Literature data including references

Since the first manned space flights it has become evident that space crew suffer from muscle atrophy and deteriorated musculo-skeletal function upon return to earth (28, 31, 35, 55, 61, 62, 66, 73, 102). Although the specificity or the magnitude or rate of these changes have not been disclosed in detail, several studies, using simulation models of weightlessness have suggested that the decrease in maximal voluntary strength is substantial during the first weeks or months (98). The mechanism(s) behind this functional change is not fully understood but it is evident that muscle atrophy only cannot explain the decrease in voluntary strength. Thus, it appears that neural adaptations (11, 13, 28, 74, 92) and/or changes related to the contractile machinery (11, 31, 44, 64, 83, 107, 108, 109, 111) are also responsible for the impaired function, because several studies are consistent in that they show muscle atrophy to be less than the decrease in muscle strength (2, 6, 14, 54). It is also evident from these studies that weightbearing muscles are more affected than non-weightbearing muscles (48, 54, 55, 67, 103). Hence, lower-limb muscles; i.e. knee or ankle extensor muscles, show more pronounced reduction in strength than upper-body muscle groups, and the extensor muscles of the knee show greater decrease in strength or size than the knee flexor muscle group. The weekly rate of whole-muscle or fiber atrophy of the quadricep muscle during the course of 1-6 weeks of unloading or bedrest has been reported to about 2-3% (2, 6, 14, 54, 57, 79). At odds with these findings are results on astronauts examined pre and post 5-11 days spaceflight (35), showing a decrease in muscle fiber size ranging 11-36%, but only a modest decrease in knee extensor strength (55). The magnitude of reduction in strength appeared to fit with the 6% decrease in muscle cross-sectional area reported after 8 d spaceflight (66). Currently, there is no information available describing molecular or muscle fiber-type specific adaptations in humans in response to space flight or simulations of space flight of extended duration.

Even though a reduction in Type I myosin heavy-chain (MHC) content has been suggested to occur in response to 5-11 d space flight (114), the bulk of existing data show no alteration in MHC composition or the proportions of fiber types in response to several weeks of unloading or bedrest (9, 14, 54, 64), despite significant muscle atrophy and impaired function. Likewise, there is no apparent difference in the atrophic response between slow and fast twitch fibers of weight-bearing muscles (11, 14, 54). Further support suggesting fiber-type specific changes to be non-existing or of limited functional importance in response to up to 6 weeks of non-weight bearing are to be found in several reports showing no alteration of the in-vivo force-velocity relationship; at least not at angular velocities up to about one third of the maximum angular velocity (2, 6, 14, 32, 34, 54). Hence, decreases in isometric, concentric and eccentric strength appear to be of similar magnitude.

Although maximum voluntary force seems to be a valid measure to reflect muscle function, work capacity or endurance of muscle might be equally important for several work tasks performed by crew in orbit or upon return to earth. Animal studies have suggested unaltered or increased fatigability of unloaded muscles (84, 85, 112), and four weeks of unloading or bedrest produced decreased work capacity and increased fatigability accompanied by decreased oxidative enzyme content (9, 57).

Regardless of the mechanism(s) being responsible for the decrease in strength, countermeasures to this effect are imperative on the future ISS. The most attractive solution to this problem seems to be the implementation of some kind of exercise program (28) for space stationed crew. Collectively, the results from numerous human studies show that resistance type exercise (1, 33, 51, 52, 53, 40, 41, 89, 96), but not aerobic endurance exercise (56 63, 105) promotes important increases in muscle size and strength. It therefore seems likely that resistance exercise would offer an effective stimuli to maintain muscle mass and strength in weightlessness. Heavy resistance training programs produce increased muscle cross-sectional area due to an increase in myofibrillar potein content (42, 96, 97, 113). In response to short-term training programs however appreciable increases in muscular strength and strength related performances are possible with no or minor muscle hypertrophy (26, 77, 79, 89, 90), suggesting neural factors in part to be responsible for training-induced increases in strength (89). Resistance training will not induce any important or favorable metabolic adaptations (95, 96). It appears, for example that heavy-resistance training, emphasizing high-load, low-repetition exercises, does not produce capillary neoformation. Hence, when pronounced hypertrophy of individual muscle fibers occurs, the capillary density may rather decrease (96). Likewise, secondary to exercise-induced muscle hypertrophy, oxidative enzyme content is reduced (96). Because of these responses, resistance training either at 1g, or in space is not likely to promote enhanced endurance.

To optimize the effects of resistance training studies it is prudent to say that such exercise should emphasize the performance of coupled concentric and eccentric muscle actions. For example, several studies have shown coupled concentric and eccentric resistance exercise to promote greater increases in strength and muscle size (26, 33, 22, 51, 53, 60) than resistance training using concentric actions only. This effect appears only in part, to be due to the inherent ability of skeletal muscle to produce more force during lengthening than shortening. Regardless of the mechanism responsible for the superior response to training comprising eccentric actions compared to training using concentric actions only, a more prolonged increase in the rate of protein synthesis occurs with eccentric actions (113). Also, from a functional point of view, coupled concentric and eccentric actions should be performed because normal locomotory activities typically comprise both types of actions.

Resistance exercise programs designed for use in a weightlessness environment must consider several physiological aspects. As indicated, an exercise task effective in promoting increases in strength and muscle size, should comprise both concentric and eccentric muscle actions and the exercise should produce relatively high muscle tension (1). Moreover, in an environment where there is a shortage of food, water and oxygen, energy expenditure must be kept low (7). Secondly, there are certain technical aspects that must be taken into account when designing exercise equipment to be used in space. Because of the lack of gravity barbells or dumb-bells, for example, can not be used. It is therefore necessary to replace the gravitational pull from a lifted weight by an alternative force or power source. Ergometers available that use dampers or friction to produce force often provide concentric resistance only. So-called isokinetic dynamometers, which accomplish eccentric resistance as well, unfortunately require electrical power on the order of 2-4 kW. Because the reported energy budget for the ISS is about 70 kW and only 10-15 kW is dedicated for scientific experiments, use of such electrically powered equipment for daily exercise seems remote.

Other mechanically powered solutions e.g., elastic cords, spring-loaded equipment or modified treadmills will probably not provide sufficient resistance (99), or do they not allow force or work to be controlled or monitored.

We have developed a mechanical, non-gravity dependent resistive exercise device (12, 14, 99) for in-flight use. It uses the inertia of rotating fly-wheels provide resistance.

Long-term bedrest is also associated with a decrease in bone density (69). The rate of decrease is however, smaller than the corresponding decrease in muscle cross-sectional area. Several studies are available showing resistance training programs are effective in increasing bone mineral density and that this effect is correlated with the strength of the anatomically related muscles (58, 86, 88, 93). The results of these studies support old observations that weight trained individuals appear to have greater bone mineral density than control subjects (30). Although it is not the intent of this study to assess changes in bone density, we believe the fly-wheel ergometer could also serve as a countermeasure to prevent bone loss as well.

There is a bulk of information available as a result of research performed on lower mammals flown in space (20, 23, 61, 82) or being exposed to unloading (20, 22, 40, 43, 44, 47, 49, 111, 112). Unfortunately, such information is not readily applicable when important decisions are to be made concerning exercise prescriptions for crew in orbit. If the exercise countermeasure paradigm proves to be effective in preventing muscle atrophy and impaired muscle function in response to 120 days of bedrest, we propose fly-wheel technology to be considered and evaluated as an exercise countermeasure on the ISS.

It is well established that prolonged bedrest brings about orthostatic intolerance (5, 45), the cause of which, in all probability, is multifactorial and may include impaired intrinsic myocardial function (90), cardiac atrophy (70) and diminished arterial baroreflex function (27). Also, increased venous distensibility resulting in increased pooling of blood in dependent parts of the body may contribute to bedrest-induced orthostatic intolerance (91).

It appears that the question of to what extent prolonged bedrest affects the distensibility of arteries and arterioles, which may also affect orthostatic tolerance, has received less attention in the past. We have recently conducted a number of experimental studies regarding the distensibility of the vasculature in human limbs. To bring about increases in the distending pressure across vessel walls of the limb we have employed a modified version of a technique originally described elsewhere (50). Briefly, the vascular distending pressures in the limb are manipulated by enclosing the body in a pressure chamber, with exception of an arm or a lower leg which remains exposed to normal atmospheric pressure. As chamber pressure is elevated, all tissues enclosed in the chamber including the heart and the central blood vessels, are pressurized in direct proportion to the applied pressure. The elevated arterial pressure is transmitted to the arteries of the unexposed test limb. Venous pressure in the test limb rises until it just exceeds the elevated pressure in the central (downstream) veins and an anterograde blood flow is resumed. In this manner the pressure across the walls of both the arteries and the veins of the test limb can be increased and maintained elevated as desired. Also, pressure-induced changes in vessel diameters and blood flow in the unexposed test limb can readily be studied by use of ultrasonographic/Doppler techniques.

By use of these techniques we have found that elevation of the distending pressure in arm vessels by >120-150 mmHg results in distension not only of veins but also of arteries and, judging from the increments in blood flow as well as the rate of formation of tissue edema, also in smaller precapillary resistance vessels (36, 38, 39, 59). In another study we explored the possibility of adapting local blood vessels to increased transmural pressure by ”pressure training” the vessels of one arm (37, 39). It appears that the distensibility of arteries/arterioles can be considerably reduced by pressure training the vessels. Thus, a pressure-training regimen consisting of three 40 min pressure sessions/week for 5 weeks, during which the vascular distending pressure was gradually increase from +65 mmHg during the first week to +105 mmHg during the last week, resulted in 50% reduction of brachial artery distensibility and about 25% reductions of the edema formation and the increase in blood flow that are normally observed in response to a vascular pressure provocation of +180 mmHg.

To investigate the possibility that the hydrostatic pressure components acting along the blood vessels in the upright posture are large enough to influence the distensibility of arteries and/or arterioles we also compared the pressure responses in leg vessels to those in arm vessels. In the arm, but not in the leg, blood flow increased markedly at the highest levels of distending pressure (i.e. when the distending pressure was elevated by more than 120-150 mmHg). The brachial and radial arteries were more pressure distensible than the posterior tibial artery. Thus, it seems that arteries and smaller precapillary vessels are more pressure resistant in the leg.

It can be assumed, that regional arterial/arteriolar compliances in humans are distributed in such a way that the hydrostatic forces acting in the upright posture are partially counteracted. These results confirm and extend previous findings by others (24, 25).

Since arteries and arterioles are more pressure resistant in the leg than in the arm and since the distensibility of such vessels changes in response to intermittent exposures to moderate pressure stimuli, it seems reasonable to hypothesize that arterial/arteriolar distensibility in the limbs will increase after 120 days of bed rest and that such increase will be especially pronounced in leg vessels. If, or to what extent, a countermeasure consisting of resistance exercise is capable of preventing a bedrest-induced increase in arterial/arteriolar distensibility is as yet an open question. It can be expected that the proposed high-resistance exercise regimen will produce acute increments in arterial pressure which are of the same magnitude and duration as those shown to decrease arterial/arteriolar distensibility in the above mentioned vascular pressure-training regimen. It is therefore conceivable that high-resitance exercise will counteract a bedrest-induced increase in arterial/arteriolar distensibility. On the other hand, muscle exercise has been shown to increase arterial distensibility, at least when distensibility is not determined during pressure provocation but merely defined as the vessel diameter excursions during the course of a normal pulse pressure wave (110). This study will explore further the impact of resistance exercise on vascular distensibility.

**References**

1. ATHA. J. Strengthening muscle. 1981. Exerc Sport Sci Rev 9, 1-78.

2. ADAMS, G.R., HATHER, B.M. & DUDLEY, G.A. 1994. Effect of short-term unweighting on human skeletal muscle strength and size. Aviat Space Environ Med 65, 1116-1121.

3. BAMMAN, M.M., HUNTER, G.R., STEVENS, B.R., GUILLIAMS M.E. & M.C. GREENISEN. 1997. Resistance exercise prevents plantar flexion deconditioning during bed rest. Med Sci Sports Exercise 29, 1462-1468.

4. BAMMAN, M.M., HUNTER, G.R., CLARKE, M.S.F., FEEDBACK, D.L., R.J. TALMADGE, STEVENS, B.R., LIEBERMAN, S.A. & M.C. GREENISEN. 1998. Impact of resistance exercise prevents plantar flexion deconditioning during bed rest on skeletal muscle sarcopenia and myosin isoform distribution. J Appl Physiol 84, 157-163.

5. BASSETT FREY, M.A., J.B. CHARLES & D.E. HOUSTON. 1990. Weightlessness and response to orthostatic stress. In: Circulatory response to the upright posture (Ed. J.J. SMITH). CRC Press. pp 65-120.

6. BERG, H.E., DUDLEY, G.A., H€GGMARK, T., OHLSƒN, H. & TESCH, P.A. 1991. Effects of lower limb unloading on skeletal muscle mass and function in humans. J Appl Physiol 70, 1882-1885.

7. BERG, H.E. & P.A. TESCH. 1992. Designing methods for musculoskeletal conditioning in weightlessness. The Physiologist 35, Suppl. 96-98.

8. BERG, H.E., DUDLEY, G.A. & TESCH, P.A. 1993. Muscle involvement in two models of closed chain leg resistance exercise estimated by MR-imaging. Med Sci Sports Exerc, Suppl 25, 181.

9. BERG, H.E., DUDLEY, G.A., HATHER, B. & TESCH, P.A. 1993. Work capacity and metabolic and morphologic characteristics of the human quadriceps muscle in response to unloading. Clin Physiol 13, 337-347.

10 BERG, H.E., TEDNER, B. & TESCH, P.A. 1993. Changes in lower limb muscle cross-sectional area and tissue fluid volume after transition from standing to supine. Acta Physiol Scand 148, 379-385.

11. BERG, H.E. Effects of unloading on skeletal muscle mass and function in man. Karolinska Institutet (Thesis). 1996.

12. BERG, H.E. & TESCH, P.A. 1994. A gravity-independent ergometer to be used for resistance training in space. Aviat Space Environ Med 65, 752-756.

13. BERG, H.E. & TESCH, P.A. 1996. Changes in muscle function in response to 10 days of lower limb unloading in humans. Acta Physiol Scand. 157, 63-70.

14. BERG, H.E., LARSSON, L., & TESCH, P.A. 1997. Lower limb skeletal muscle function after 6 wk bed rest. J Appl Physiol 82, 182-188.

15. BERG, H.E. & TESCH, P.A. 1998. Force and power characteristics of a resistive exercise device for use in space. Acta Astronautica 42, 1-8.

16. BERGER, R.A. 1962. Effect of varied weight training programs on strength. Res Q 33, 168-181.

17. BERRY, P., BERRY, I. & MANELFE, C. 1993. Magnetic resonance imaging evaluation of lower limb muscles during bed rest - a microgravity simulation model. Aviat Space Environ Med 64, 212-218.

18. BIOLO, G., MAGGI, S.P., WILLIAMS, B.D., TIPTON, K.D., & WOLFE, R.R. 1995. Increased rates of muscle protein turnover and amino acid transport after resistance exercise in humans. Am J Physiol 268, E:514-520.

19.BORG, G.V.A. 1982. Psychophysical bases of perceived exertion. Med Sci Sports Exerc14, 377-381.

20. BOOTH, F.W. & GOLLNICK, P.D. 1983. Effects of disuse on the structure and function of skeletal muscle. Med Sci Sports Exerc 15, 415-420.

21. BOOTH, F.W. & KIRBY, C.R. 1992. Changes in skeletal muscle gene expression consequent to altered weight bearing. Am J Physiol 262, R329-R332.

22. BOOTH, F.W. & SEIDER, M.J. 1979. Recovery of skeletal muscle after 3 mo of hindlimb immobilization in rats. J Appl Physiol 47, 435-439.

23. CAIOZZO, V.J., BAKER, M.J., HERRICK, R.E., MING TAO & BALDWIN, K.M. 1994. Effect of spaceflight on skeletal muscle: mechanical properties and myosin isoform content of a slow muscle. J Appl Physiol 76, 1764-1773.

24. COLES, D.R. 1957. Heat elimination from the toes during exposure of the foot to subatmospheric pressures. J Physiol 131, 277-289.

25. COLES, D.R. & C.G. PATTERSON. 1957. The capacity and distensibility of the blood vessels of the human hand. J Physiol 135, 163-70.

26. COLLIANDER, E.B. & TESCH, P.A. 1990. Effects of eccentric and concentric muscle actions in resistance training. Acta Physiol Scand 140, 31-39.

27. CONVERTINO, V.A., D.F. DOERR, D.L. ECKBERG, J.M. FRITSCH & J. VERNIKOS-DANELLIS. 1990. Head-down bed rest impairs vagal baroreflex responses and provokes orthostatic hypotension. J Appl Physiol 68, 1458-1464.

28. CONVERTINO, V.A. 1996. Exercise as a countermeasure for physiological adaptation to prolonged spaceflight. Med Sci Sports Exerc 28, 999-1014.

29. CONVERTINO, V.A., DOERR, D.F., MATHES, K.L., STEIN, S.L. & BUCHANAN, P. 1989. Changes in volume muscle compartment, and compliance of the lower extremities in man following 30 days of exposure to simulated microgravity. Aviat Space Environ Med 60, 653-658.

30. DALƒN, N., LAFTMAN, P., OHLSƒN, H. & STROMBERG, L. 1985. The effect of athletic activity on the bone mass in human diaphyseal bone. Orthopedics. 8, 1139-1141.

31. DESPLANCHES, D. 1997. Structural and functional adaptations of skeletal muscle to weightlessness. Int J Sports Med.18, (Suppl. 4) s.259-264.

32. DUDLEY, G.A., DUVOISIN, M.R., CONVERTINO, V.A. & BUCHANAN, P. 1989. Alterations of the in vivo torque-velocity relationship of human skeletal muscle following 30 days exposure to simulated microgravity. Aviat Space Environ Med 60, 659-663.

33. DUDLEY, G.A., TESCH, P.A., B.J. MILLER, B.J. & BUCHANAN, P. 1991. Importance of eccentric actions in performance adaptations to resistance training. Aviat. Space Environ Med 62, 543-550.

34. DUDLEY, G.A., DUVOISIN, M.R., ADAMS, G.R., MEYER, R.A., BELEW, A.H. & BUCHANAN, P. 1992. Adaptations to unilateral lower limb suspension in humans. Aviat Space Environ Med 63, 678-683.

35. EDGERTON, V.R., ZHOU, M.Y., OHIRA, Y., KLITGAARD, H., JIANG, B., BELL, G., HARRIS, B., SALTIN, B., GOLLNICK, P.D., ROY, R.R., DAY, M.K. & GREENISEN, M. 1995. Human fiber size and enzymatic properties after 5 and 11 days of spaceflight. J Appl Physiol 78, 1733-1739.

36. EIKEN, O. & R. K…LEGپRD. Comparison between the upper and lower exremity with regard to vascular distensibility and the susceptibility of pain induced by high intravasular pressures. Abstract submitted to 21th Annual International Gravitational Physiology Meeting.

37. EIKEN, O. & R. K…LEGپRD. Pain in the arms induced by markedly increased intravascular pressure decreases after repeated exposures to moderately increased pressures. J. Gravit. Physiol. (in press).

38. EIKEN, O. & R. K…LEGپRD. Arm pain caused by elevation of distending pressure in local intravascular segments - on the site of origin and relationships of pain and distension of arteries and veins. Aviat Space Environ Med. (Submitted)

39. EIKEN, O. & R. K…LEGپRD. 1999. Arm pain induced by increased intravascular pressure: effects of repeated exposures. Aviat Space Environ Med 70, 398.

40. FELL, R.D., GLADDEN, L.B., STEFFEN, J.M. & MUSACCHIA, X.J. 1985. Fatigue and contraction of slow and fast muscles in hypokinetic/hypodynamic rats. J Appl Physiol 58, 65-69.

41. FERRANDO, A.A., STUART, C.A., BRUNDER, D.G. & HILLMAN, G.R. 1995. Magnetic resonance imaging quantitation of changes in muscle volume during 7 days of strict bed rest. Aviat Space Environ Med 66, 976-981.

42. FERRANDO, A.A., TIPTON, K.D., BAMMAN, M.M. & WOLFE, R.R. 1997. Resistance exercise maintains skeletal muscle protein synthesis during bed rest. J Appl Physiol 82, 1110-1115.

43. FITTS, R.H., MCDONALD, K.S. & SCHLUTER, J.M. 1991. The determinants of skeletal muscle force and power: their adaptability with changes in activity pattern. J Biomechanics 24 (Suppl 1), 111-122.

44. FITTS, R.H., METZGER, J.M., RILEY, D.A. & UNSWORTH, B.R. 1986. Models of disuse: a comparison of hindlimb suspension and immobilization. J Appl Physiol 60, 1946-1953.

45. FORTNEY, S.M., V.S. SCNEIDER & J.E. GREENLEAF. 1996. The physiology of bed rest. In: Handbook of Physiology, Sect. IV, Environmental Physiology, Vol. II (EDS.: M.J. FREGLY, C.M. BLATTEIS). Oxford Univ. Press. pp: 889-939.

46. GAMRIN, L., BERG, H.E., ESSƒN, P., TESCH, P.A., HULTMAN, E., GARLICK, P.J., MCNURLAN, M.A. & WERNERMAN, J. 1998. The effect of unloading on protein synthesis in human skeletal muscle. Acta Physiol Scand 163, 369-377.

47. GARDETTO, P.R., SCHLUTER, J.M. & FITTS, R.H. 1989. Contractile function of single muscle fibers after hindlimb suspension. J Appl Physiol 66, 2739-2749.

48. GOGIA, P.P., SCHNEIDER, V.S., LEBLANC, A.D., KREBS, J., KASSON, C. & PIENTOK, C. 1988. Bed rest effect on extremity muscle torque in healthy men. Arch Phys Med Rehabil 69, 1030-1032.

49. GOLDSPINK, D.F., MORTON, A.J., LOUGHNA, P. & GOLDSPINK, G. 1986. The effect of hypokinesia and hypodynamia on protein turnover and the growth of four skeletal muscles of the rat. Pflںgers Arch 407, 333-340.

50. GREENFIELD, A.D.M. & G.C. PATTERSON. 1954. Reactions of the blood vessels of the human forearm to increases in transmural pressure. J Physiol 125, 508-524.

51. H€KKINEN, K. & KOMI, P.V. 1981. Effect of different combined concentric and eccentric muscle work regimens on maximal strength development. J Hum Move Stud 7, 33-44.

52. H€KKINEN, K., ALƒN, M., & KOMI, P.V. 1985. Changes in isometric force- and relaxation time, electromyographic and muscle fibre characteristics of human skeletal muscle during strength training and detraining. Acta Physiol Scand 125, 573-585.

53. HATHER, B.M., TESCH, P.A., BUCHANAN, P. & DUDLEY, G.A. 1991. Influence of eccentric actions on skeletal muscle adaptations to resistance training. Acta Physiol Scand 143, 177-185.

54. HATHER, B.M., ADAMS, G.R., TESCH, P.A. & DUDLEY, G.A. 1992. Skeletal muscle responses to lower limb suspension in humans. J Appl Physiol 72, 1493-1498.

55. HAYES, J.C., MCBRINE, J.J., ROPER, M.L., STRICKLIN, M.D., SICONOLFI, S.F. & GREENISEN, M.C. 1992. Effects of space flight on skeletal muscle performance. FASEB J 65, A1770.

56. HICKSON, R.C. 1980. Interference of strength development by simultaneously training for strength and endurance. Eur J Appl Occup Physiol 45, 255-269.

57. HIKIDA, R.S., GOLLNICK, P.D., DUDLEY, G.A., CONVERTINO, V.A. & BUCHANAN, P. 1989. Structural and metabolic characteristics of human skeletal muscle following 30 days of simulated microgravity. Aviat Space Environ Med 60, 664-670.

58. JOHNSON, R.B. 1998. The bearable lightness of being: bones, muscles, and spaceflight. Anat Rec 253, 24-27.

59. K…LEGپRD, R., H. …RNHAGEN, N. HOLMSTR…M, B.LINDBORG, O. EIKEN. 1998. Pressure distension in veins and arteries in the human arm. Aviat Space Environ Med 69, 222.

60. KOMI, P.V. & BUSKIRK, E.R. 1972. Effect of eccentric and concentric muscle conditioning on tension and electrical activity of human muscle. Ergonomics 15, 417-434.

61. KOZLOVSKAYA, I.B., BARMIN, V.A., STEPANTSOV, V.I. & KHARITONOV, N.M. 1990. Results of studies of motor functions in long-term space flights. The Physiologist 33, S1-S3.

62. KOZLOVSKAYA, I.B., KREIDICH, Y.V., OGANOV, V.S. & KOSERENKO, O.P. 1981. Pathophysiology of motor functions in prolonged manned space flights. Acta Astronautica 8, 1059-1072.

63. KRAEMER, W.J., PATTON, J.F., GORDON, S.E., HARMAN, E.A., DESCHENES, M.R., REYNOLDS, K., NEWTON, R.U., TRIPLETT, N.T. & DZIADOS, J.E. 1995. Compatibility of high-intensity strength and endurance training on hormonal and skeletal muscle adaptations. J Appl Physiol 78, 976-989.

64. LARSSON, L., LI, X., BERG, H.E. & FRONTERA, W.R. 1996. Effects of removal of weight-bearing function on contractility and myosin isoform composition in single human skeletal muscle cells. Pflںgers Arch 432, 320-328.

65. LINK, T.M., MAJUMDAR, S., AUGAT, P., LIN, J.C., NEWITT, D., LANE, N.E., & GENANT, H.K. 1998. Proximal femur: assessment for osteoporosis with T2 decay characteristics at MR imaging. Radiology 209, 531-536.

66. LEBLANC, A.D., ROWE, R., SCHNEIDER, V., EVANS, H. & HEDRICK, T. 1995. Regional muscle loss after short duration spaceflight. Aviat Space Environ Med 66, 1151-1154.

67. LEBLANC, A.D., GOGIA, P., SCHNEIDER, V.S., KREBS, J.M., SCHONFELD, E. & EVANS, H.J. 1988. Calf muscle area and strength changes after five weeks of horizontal bed rest. Am J Sports Med 16, 624-629.

68. LEBLANC, A.D., SCHNEIDER, V.S., EVANS, H.J., PIENTOK, C., ROWE, R. & SPECTOR, E. 1992. Regional changes in muscle mass following 17 weeks of bed rest. J Appl Physiol 73, 2172-2178.

69. LEBLANC, A.D., SCHNEIDER, V.S., EVANS, H.J., ENGELBRETSON, D.A. & KREBS, J.M. 1990. Bone mineral loss and recovery after 17 weeks of bed rest. J Bone Mineral Res, 5, 843-850.

70. LEVINE, B.D., ZUCKERMAN, J.H. & PAWELCZYK, J.A. 1997. Cardiac atrophy after bed-rest deconditioning, a nonneural mechanism for orthostatic intolerance. Circulation 96: 517-525.

71. MARTIN, T.P., EDGERTON, V.R. & GRINDELAND, R.E. 1988. Influence of spaceflight on rat skeletal muscle. J Appl Physiol 65, 2318-2325.

72. MARTIN, A.D. & MCCULLOCH, R.G. 1987. Bone dynamics: stress, strain and fracture. J Sports Sci 5, 155-163.

73. MCCALL, G.E., C. GOULET, G.E. BOORMAN, J.A. HODGSON, R.R. ROY, M.C. GREENISEN & EDGERTON, V.R. 1997. Maintenance of plantar flexor muscle function in human during a 17-day space flight. Med Sci Sports Exerc 29, S28.

74. MCCOMAS, A.J. 1994. Human neuromuscular adaptations that accompany changes in activity. Med Sci Sports Exerc 26, 1498-1509.

75. MERTON, P.A. 1955. Voluntary strength and fatigue. J Physiol 123, 553-564.

76. MUSACCHIA, X.J., STEFFEN, J.M. & FELL, R.D. 1988. Disuse of skeletal muscle: Animal models. Exerc Sport Sci Rev 16, 61-87.

77. NARICI, M.V., ROI, G.S., LANDONI, L., MINETTI, A.E. & CERRETELLI, P. 1989. Changes in force, cross-sectional area and neural activation during strength training and detraining of the human quadriceps. Eur J Appl Physiol Occup Physiol 59, 310-319.

78. NARICI, M.V., KAYSER, B,. BARATTINI, P. & CERRETELLI, P. 1997. Changes in electrically evoked skeletal muscle contractions during 17-day spaceflight and bed rest. Int J Sports Med 18, (Suppl. 4) s.290-292.

79. PLOUTZ, L.L., TESCH, P.A., BIRO, R.L. & DUDLEY, G.A. 1994. Effect of resistance training on muscle use during exercise. J Appl Physiol 76, 1675-1681.

80. PLOUTZ-SNYDER, L.L., TESCH, P.A., CRITTENDEN, D.J. & DUDLEY, G.A. 1995. Effect of unweighting on skeletal muscle use during exercise. J Appl Physiol 79, 168-175.

81. PLOUTZ-SNYDER, L.L., TESCH, P.A. & G.A. DUDLEY. 1998. Increased vulnerability to exercise induced dysfunction and muscle injury after concentric training. Arch Phys Med Rehab 79, 58-61.

82. RILEY, D.A., ELLIS, S., GIOMETTI, C.S., HOH, J.F.Y., ILYINA-KAKUEVA, E.I., OGANOV, V.S., SLOCUM, G.R., BAIN, J.L.W. & SEDLAK, F.R. 1992. Muscle sarcomere lesions and thrombosis after spaceflight and suspension unloading. J Appl Physiol 73, Suppl. 33S-43S.

83. RILEY, D.A., BAIN, J.L.W, THOMPSON, J.L, FITTS, R.H., WIDRICK, J. neJ., TRAPPE, S.W., TRAPPE, T.A. & COSTILL, D.L. 1998. Disproportionate loss of thin filaments in human soleus muscle after 17-day bed rest. Muscle & Nerve 21, 1280-1289.

84. ROBINSON, G.A., ENOKA, R.M. & STUART, D.G. 1991. Immobilization-induced changes in motor unit force and fatigability in the cat. Muscle Nerve 14, 563-573.

85. ROY, R.R., BALDWIN, K.M. & EDGERTON, V.R. 1991. The plasticity of skeletal muscle: effects of neuromuscular activity. Exerc Sports Sci Rev 19, 269-312.

86. RUBIN, C.T. & LANYON, L.E. 1984. Regulation of bone formation by applied dynamic loads. J Bone Jt Surg Am 66, 397-401.

87. RYAN, A.S., TREUTH, M.S., RUBIN, M.A., MILLER, J.P., NICKLAS, B.J., LANDIS, D.M., PRATLEY, R.E., LIBANATI, C.R., GUNDBERG, C.M., & HURLEY, B.F. 1994. Effects of strength training on bone mineral density: hormonal and bone turnover relationships. J Appl Physiol 77, 1678-1684.

88. RYAN, A.S., TREUTH, M.S., HUNTER, G.R. & ELAHI, D. 1998. Resistive training maintains bone mineral density in postmenopausal women. Calcif Tissue Int 62, 295-299.

89. SALE, D.G. 1991. Neural adaptation to strength training. In: The Encyclopedia of Sports Medicine: "Strength and Power in Sports" (Ed. Komi, P.V.). Blackwell Scientific Publications, Oxford. pp. 249-265.

90. Saltin, B., & G. BLOMQVIST, J.H. MITCHELL, R.L. JOHNSON, JR, K. WIDENTAHL & C.B. CHAPMAN. 1968. Response to exercise after bed rest and after training. A longitudinal study of adaptive changes in oxygen transport and body composition. Circulation 38 (Suppl 7): VII-1-VII-78.

91. SAVILOV, A.A., V.I. LOBACHGIK & A.M. BABIN. 1990. Cardiovascular function of man exposed to LBNP tests. The Physiologist 33, S128-S132.

92. SCHULTZE, K.E., GALLAGHER, P.M. & S.W. TRAPPE. A minimalistic model of resistance training effects on skeletal muscle function during unloading. Med Sci Sports Exerc, 31 No 5 (Suppl), S 329.

93. TAAFFE, D.R., ROBINSON, T.L., SNOW, C.M. & MARCUS, R. 1997. High-impact exercise promotes bone gain in well-trained female athletes. J Bone Miner Res, 12, 255-260.

94. TESCH, P.A., LINDBORG, B.P.O. & COLLIANDER, E.B. 1990. Evaluation of a dynamometer measuring torque during uni- and bilateral concentric and eccentric muscle actions. Clin Physiol 10, 1-9.

95. TESCH, P.A., THORSSON, A. & COLLIANDER, E.B. 1990. Effects of eccentric and concentric resistance training on skeletal muscle substrates, enzyme activities and capillary supply. Acta Physiol Scand 140, 575-580.

96. TESCH, P.A. 1991. Short- and long-term histochemical and biochemical adaptations to heavy resistance exercise. In: The Encyclopedia of Sports Medicine: "Strength and Power in Sports" (Ed. Komi, P.V.). Blackwell Scientific Publications, Oxford. pp. 239-248.

97. TESCH, P.A. 1991. Training for bodybuilding. In: The Encyclopedia of Sports Medicine: "Strength and Power in Sports" (Ed. Komi, P.V.). Blackwell Scientific Publications, Oxford. pp. 370-380.

98. TESCH, P.A., PLOUTZ. L.L. & DUDLEY, G.A. 1994. Effects of 5 weeks of lower limb suspension on muscle size and strength. J Gravit Physiol 1, 59-60.

99. TESCH, P.A. & BERG, H.E. 1997. Resistance training in space. Int J Sports Med 18, (Suppl. 4) s.322-324.

100. TESCH, P.A. & BERG, H.E. 1996. Effects of 16 days bed rest on quadricep muscle function and mass. In: Life and Microgravity Sciences Spacelab Mission: Human research pilot study. Six month report. NASA Technical Memorandum 110395; p. 26.

101. TESCH, P.A. & BERG, H.E. 1998. Effects of spaceflight on muscle. J Gravit Physiol. 51, P-19-22.

102. THOMASON, D.B. & BOOTH, F.W. 1990. Atrophy of the soleus muscle by hindlimb unweighting. J Appl Physiol 68, 1-12.

103. THORNTON, W.E. & RUMMEL, J.A. 1977. Muscular deconditioning and its prevention in space flight. In: Biomedical Results from Skylab (Eds. Johnston, R.S. & Dietlein, L.F.) National Aeronautics and Space Administration (NASA), Washington, DC. pp. 191-197.

104. TRAPPE, S.W., TRAPPE, T.A., COSTILL, D.L. & FITTS, R.H. 1995. ASGSB Bulletin 9, 44.

105. TRAPPE, S.W., COSTILL, D.L., GOODPASTER, B.H. & PEARSON, D.R. 1996. Calf muscle strength in former elite distance runners. Scand J Med Sci Sports 6, 205-211.

106. VIITASALO, J.H.T. & KOMI, P.V. 1977. Signal characteristics of EMG during fatigue. Eur J Appl Physiol 37, 111-121.

107. WIDRICK, J.J., ROMATOWSKI, J.G., BAIN, J.L., TRAPPE, S.W., TRAPPE, T.A., THOMPSON, J.L., COSTILL, D.L., RILEY, D.A. FITTS, R.H. 1997. Effects of 17 days of bed rest on peak isometric force and unloaded shortening velocity of human soleus fibers. Am J Physiol 273, C1690-1699.

108. WIDRICK, J.J., NORENBERG, K.M., ROMATOWSKI, J.G., BLASER, C.A., KARHANEK, M., SHERWOOD, J., TRAPPE, T.T., TRAPPE, S.W., COSTILL, D.L. & FITTS, R.H. 1998. Force-velocity-power and force-pCa relationships of human soleus fibers after 17 days of bed rest. J Appl Physiol 85, 1949-1956.

109. WIDRICK, J.J., NUTH, S.W., NORENBERG, K.M., ROMATOWSKI, J.G., BAIN, J.L., RILEY, D.A., KARHANEK, M.,TRAPPE, S.W., TRAPPE, T.A., COSTILL, D.L. & FITTS, R.H. 1999. Effect of 17 day spaceflight on contractile properties of human soleus muscle fibres. J Physiol 99, 915-930.

110. WIJNEN, J.A.G., H. KUIPERS, M.J. KOOL, A.P.G. HOEKS, M.A. van BAAK, H.J.A. STRUYKER BOUDIER, F.T.J. VERSTAPPEN & L.M.A.B. Van BORTEL. 1991. Vessel Wall properties of larger arteries in trained and sedentary subjects. Bas Res Cardiol 86 (suppl 1), 25-29.

111. WINIARSKI, A.M., ROY, R.R., ALFORD, E.K., CHIANG, P.C. & EDGERTON, V.R. 1987. Mechanical properties of rat skeletal muscle after hind limb suspension. Exp Neurol 96, 650-660.

112. WITZMANN, F.A., KIM, D.H. & FITTS, R.H. 1983. Effect of hindlimb immobilization on the fatigability of skeletal muscle. J Appl Physiol 54, 1242-1248.

113. WONG, T.S. & BOOTH, F.W. 1990. Protein metabolism in rat tibialis anterior muscle after simulated chronic eccentric exercise. J Appl Physiol 69, 1718-1724.

1. ZHOU, M-Y., KLITGAARD, H., SALTIN, B., ROY, R.R., EDGERTON, V.R. & GOLLNICK, P. D. 1995. Myosin heavy chain isoforms of human muscle after short term space flight. J Appl Physiol 78, 1740-1744.

#### Protocol justification according to current scientific knowledge

Although changes in muscle size and strength will occur within a couple of weeks, the interest in this particular study is to examine changes which will occur in response to *extended* missions on the International Space Station (see 6.4.1.4). The information gained from this study with regard to skeletal muscle adaptations in response to resistive exercise is novel. **Also, no previous studies have examined the effects of long-duration muscle unloading only or resistance exercise of unloaded muscle on vascular distensibility.**

#### Justification of study duration

Although changes in muscle size and strength will occur within a couple of weeks, the interest in this particular study is to examine changes which will occur in response to *extended* missions on the International Space Station.

#### Rationale for drug (if used) and dosage selection

N/A

### Objectives and hypothesis

#### Main objective of the protocol

Although it should be acknowledged that studies intended to answer what mechanisms are responsible for the adaptations taking place as a result of spaceflight are important, we believe there is sufficient data to support testing in man, the hypothesis that resistance exercise is an effective countermeasure in space. Therefore, we intend to study the effects of resistance training in subjects performing 90 days of an analog to spaceflight - bedrest. An ergometer, which will function regardless of gravity will be used to serve this purpose. More specifically, the main objective of this research is to study the efficacy of a resistive exercise paradigm, using non-gravity dependent technology and designed for use in space, on muscle integrity, size and strength of unloaded muscle**. In addition, and because orthostatic tolerance is a serious concern after spaceflight, and the mechanisms behind this phenomenon are not fully understood another aim of this study is to examine the effects of 90 days bedrest on vascular distensibility with or without resistance exercise.**

#### Identification of main parameters

The following main parameters will be measured: 1. Maximal and submaximal dynamic and isometric muscle force of the knee extensor and plantar flexor muscle groups. 2. Surface electromyography (EMG)of quadriceps and calf muscles (see 1). 3. Muscle cross-sectional of lower-limb muscles (thigh and calf). 4. Muscle protein composition, muscle fiber type composition and muscle enzyme content of soleus and vastus lateralis muscles. 5. Vascular distensibility, assessed by employing a hyperbaric chamber “provocation model”; blood vessel diameter, blood volume flow (i.e. vessel diameter and limb mean flow-velocity) and forearm/lower leg volume. 6. Heart rate and blood pressure and 7. Rate of perceived discomfort/pain.

#### Tested hypothesis

We will test the hypothesis that resistance type training ameliorates the decrease in muscle strength and mass demonstrated in response to spaceflight or simulations of spaceflight. We will also test the hypothesis that bedrest increases vascular distensibility and furthermore that resistance training performed during bedrest will off-set this effect.

#### Expected results

We are confident that BR only, will produce decreases in muscle strength and other indices of deteriorated muscle function. We expect atrophy of the quadriceps muscle to be significant, but of somewhat less magnitude than the decrease in strength in response to BR. The rate of atrophy of the soleus and quadriceps muscles will be of similar order of magnitude. We believe maximal EMG activity, as a measure of neural drive, will decrease and the rate of increase in EMG in response to a given submaximal load, as a measure of fatigability, to be potentiated following bedrest. This study will disclose whether soleus and quadriceps muscles respond differently to bedrest with regard to potential changes in protein composition, fiber type composition and enzyme content. We hypothesize that the proposed resistance exercise regimen will ameliorate and most likely eliminate the negative effects of bedrest on voluntary strength during knee extension. Because the quadriceps muscle is involved in the proposed exercise we believe this muscle, will maintain size and integrity after resistance exercise performed during bedrest. We are however unsure whether resistance exercise will diminish the increased fatigue susceptibility and decreased oxidative enzyme activity, which we believe will occur in response to bedrest. **We expect vascular distensibility will increase as a result of bedrest. We believe this effect is abolished if resistive exercise is performed during bedrest. These experiments will enhance our understanding how unloaded skeletal muscle and the peripheral vascular system responds to episodes of resistive exercise.** This information is crucial in designing countermeasures programs for long-duration spaceflights. In addition, the results of these experiments have important clinical applications.

#### Secondary objectives

A second aim is to validate the feasibility of the particular gravity-independent resistive exercise device and paradigm employed in this study.

### Specific inclusion and non-inclusion criteria for test subjects

Standard physical examination. Healthy, middle-age men with no previous knee or ankle joint pathologies or vascular diseases. No allergic reaction to local anaesthesia. No history of claustrophobia.

### Precise description of the experimental protocol

#### Tests description (methods, duration, time of day, protocol conditions, etc.)

Bedrest: Subjects will perform bedrest. A 6° head-down tilt position will be maintained and the subjects are requested to comply with the general experiment requirements.

Fly-wheel ergometer

For the purpose of training, an exercise configuration using fly-wheel technology and constructed for the bilateral squat and heel raise will be used. It will be modified and constructed such that it will fit onto a sturdy "bed" and employed for exercise at a 6° head down tilt position. In any fly-wheel exercise configuration a sturdy strap is fixed to the main steel axle, which holds the fly-wheels of the device. By a pushing or pulling action the applied force initiates unwinding of the strap thereby imparting energy into the fly-wheel. Kinetic energy will increase as a function of the applied force and thus the rotational speed of the spinning fly-wheel. The mechanical work produced during the pushing or concentric muscle action is then utilized in the following braking or eccentric movement when the pull of the fly-wheel recoils the strap. Using the inertia of a rotating fly-wheel, not only the magnitude but also the feel, typical of loading using traditional weights is produced, and fly-wheels may produce forces exceeding 3000N. The smooth resistance produced by a weight-stack or barbell due to the inborn inertia of its mass, is resembled by the living force of the spinning fly-wheel.

The frame of the device including the foot stance is crafted in steel and aluminum. It has a padded seat that slides on two main rails. Two plastic fly-wheels (1.95 kg each) are used. The trainee is positioned on the slide, to which the strap is anchored through a pulley, and fixed using padded shoulder support,. While sliding along the rails, the subject pushes horizontally against the footplate. Strap length is adjusted to comply with individual body stature differences. From a starting position of about 80°knee angle, the subject initiates fly-wheel rotation through the pull of the strap. The strap will begin to unwind, and energy is imparted to the fly-wheel. Once the pushing concentric phase has been completed at almost full knee extension, the strap will start rewinding by virtue of the kinetic energy of the rotating fly-wheel and thus, returning the subject towards the footplate. While attempting to resist the force produced by the pull of the fly-wheel recoiling the strap, an eccentric muscle action is now performed. Once the fly-wheel has come to a stop, the next cycle is initiated. The heel raise is performed using a similar strategy and and extended strap length but allowing movement about the ankle joint only.

Resistance training protocol

Subjects will perform resistance exercise (squats and heel raises) during bed rest (BRE), on the inertial ergometer two (uneven weeks) or three (even weeks) times weekly. Each session consists of four sets of 7 maximal coupled concentric-eccentric muscle actions using two fly-wheels. Sets are interspersed by 2 min rest periods. Exercise will be preceded by a 5 min warm-up consisting of three sets of 6 submaximal actions with progressively increased effort. Thus, each exercise session, including warm-up, will be completed in less than 30 min. Force, power and work will be measured or calculated during each exercise session. Subjects will also receive instant performance feed-back while exercising on the ergometer. Using strain-gauge technique and an electrogoniometer, force and joint angle will be displyed on a PC display. An A/D converting system (MuscleLab™, Ergotest AS, Langesand, Norway) samples and integrates the signals for feed-back and training log.

Strength testing

Unilateral strength for both limbs will be measured in a seated knee extension fly-wheel ergometer. Maximal isometric force (maximal voluntary contraction; MVC) is assessed at 90° and 120° knee angle, respectively and subsequently force-rise time is measured at 120°, from an initial force level of 20% of MVC. Three trials will be allowed to assess MVC and force-rise time. Torque will also be measured during maximal voluntary concentric and eccentric knee extensions and using the fly-wheel ergometer, by requesting subjects to perform one bout of 7 consecutive maximal coupled actions. Heel raise force will be assessed in the isometric mode only. The order (right vs. left limb) for each of the different tests employed will be random among subjects. Bilateral strength will also be measured during squat and heel raises in the bed fly-wheel ergometer using this protocol.

Electromyography (EMG)

EMG will be recorded during maximal knee extension exercise. Also, using visual feedback (force is shown on a PC display) the subject will be asked to maintain a constant force level, which corresponds to 40% of MVC (pre-bedrest value), for 60 seconds. EMG is recorded simultaneously. Knee extensor EMG activity from the three superficial aspects of quadriceps femoris is recorded using disposable bi-polar Ag-Ag/Cl surface EMG electrodes (Multi Bio Sensors Inc., El Paso, TX.) affixed over m. vastus medialis, m. vastus lateralis and m. rectus femoris. Electrode position is retained by using cutaneous ink marks and transparent plastic sheets marked for electrodes and anatomical landmarks, proximal patella and anterior iliac crest. Individual amplification level of each EMG electrode channel will be maintained across sessions. After being filtered with low and high cut-off frequencies (9.5 and 510 Hz) the raw EMG signal is full-wave rectified and passed through a smoothing window of 44 ms time constant. Torque and EMG signals are digitized at 100 Hz and processed using MuscleLab™ (Ergotest AS, Langesund, Norway) for the MP100WS. The mean value of activity recorded from the three electrode sites are used for comparison. EMG values are also normalized to pre-bedrest using the baseline measurements.

Imaging techniques and analyses

Before and after BR or BRE the cross-sectional area (CSA) of individual muscles of the thigh will be measured using magnetic resonance imaging (MRI; Siemens Somatom Impact 1.5 T, Erlangen, Germany) at standard settings (TR/TE = 700/17 ms). After data transfer, CSA measurement and calculation are performed using software (NIH Image 1.60) on the Macintosh. Bilateral scans will be obtained after 1 h of bed­rest to avoid the influence of potential fluid shifts (10, 11).

Subjects are positioned with a foot restraint for fixation. Serial scans are obtained from the knee joint to the femoral head and from the ankle to the knee joint, respectively, as estimated from frontal or sagittal scout images. Using computerized planimetry, areas of interest (total limb, muscle plus bone, bone or individual muscle bellies; mm. vastus lateralis, vastus medialis, vastus intermedius, rectus femoris, biceps femoris, semitendinosus, semimembranosus, adductor magnus, adductor longus and gracilis and soleus and gastrocnemius) will be identified from the displayed image and manually circumscribed and then automatically computed. Similarly, muscle CSA of the soleus and gastrocnemius muscles will be assessed. Subcutaneous fat and total muscle CSA are also calculated using measured areas.

Muscle biopsies and analyses

Percutaneous muscle biopsies will be obtained from the right m. vastus lateralis and soleus respectively using the Bergstrom needle technique. Tissue samples will be divided for various analyses and subsequently frozen and stored. Freze-dried samples, dissected free from connective tissue, blood and fat, will be homogenized and enzyme activities of citrate synthase and phosphofructokinase will be determined using fluorometry. In homogenized thin serial sections, myosin heavy chain composition (MHC) will be determined using one-dimensional sodium dodecyl sulphate-polyacrylamide gel electrophoresis; separating gels being silver stained and scanned in a soft-laser densitometer. Type I, IIA, and IIB MHC composition is expressed as the amount relative to total MHC in each sample.

Vascular distensibility

Vascular distensibility will be determined in all subjects by means of a vascular pressure provocation employing a hyperbaric chamber model (see below). For the individual subject, a provocation is performed for one leg and one arm prior to BR, within 7 days as well as 4 weeks after BR. If maintainance of a normal wall stiffness in the arteries/arterioles of the leg is dependent on the regular hydostatic pressure loading afforded by the upright posture, then the proposed BR regimen is likely to considerably increase arterial/arteriolar distensibility. In light of previous studies it can be assumed that such changes in arterial/arteriolar distensibility readily can be detected by the proposed techniques.

Method and protocol for vascular pressure provocation

The experiments are carried out with the subject sitting in a pressure chamber with one arm (here termed the ”test arm”) slipped through a hole in the chamber door, or with the subject lying in the chamber with the lower portion of a leg (”test leg”) protruding through the hole in the door. The test arm/leg is supported at the level of the heart by means of a stand. The chamber opening is hermetically sealed slightly distally of the axilla or proximally of the knee by use of a short self-sealing rubber sleeve for the limb. When the arm is tested the subject is provided with a harness around the thorax to prevent movement of the arm and/or activity of trunk and arm muscles when the pressure in the chamber is elevated. Likewise, when the leg is tested movement/muscle activity during pressurization is avoided by use of a saddle-like support for the subject. During the experiments ambient temperature is maintained at 25-28 oC at the site of the test limb.

Each experiment commences with a 10 min baseline period with normal atmospheric pressure in the chamber. Thereafter chamber pressure is increased every 2.5 min in steps of 30 mmHg up to a supraatmospheric pressure of 180 mmHg for the arm and to 240 mmHg for the leg; the pressure provocation is discontinued at lower pressure levels if discomfort/pain becomes severe. Thereafter chamber pressure is rapidly released and a 5 min recovery period at atmospheric pressure ensues. Recordings of heart rate, arterial pressure, vessel diameters, blood-volume flow, tissue impedance and rating of preceived pain are obained during the last 1.5 min at each pressure level

Cardiovascular recording techniques during vascular pressure provocation

Diameters of blood vessels in the test limb are measured using an ultrasonographic technique. Measurements are conducted in B-mode image during end-diastole (determined from the ECG) as wall-to-wall distance in sagital section using a 6.0-11.0 MHz linear array transducer (Aspen, Acuson, CA, USA). For the arm, diameters are measured in the brachial artery, brachial vein and the cephalic vein 5-10 cm proximal to the cubital fossa, and in the radial artery 5 cm proximal to the wrist. For the leg, diameters are measured in the tibial posterior artery 5 cm proximal to the ankle and in the great sapheneous vein 15 cm proximal to the ankle.

Blood volume flow is measured in the brachial and tibial arteries by simultaneous measurements of vessel diameter and mean flow-velocity employing an ultrasound/Doppler technique (6.0-11.0 MHz tansducer; Aspen Acuson). Volume flow is then calculated by multiplying vessel crossectional area by the time integral of the mean flow-velocity.

Heart rate is derived from electrocardiographic recordings using a bipolar precordial lead. Systolic, mean and diastolic arterial pressures (SAP, MAP, DAP) are measured in the index finger of an arm inside the chamber using a volume-clamp technique (Portapres, Model 2.0, TNO Biomedical Instrumentation, Amsterdam, Netherlands). The level of the heart is used as the point of reference for the arterial pressure measurements. Arterial distending pressure is calculated by adding chamber pressure to DAP, whereas venous distension pressure is approximated as equal to chamber pressure.

Changes in forearm/lower leg volume are estimated from impedance plethysmographic recordings using a tetrapolar constant-current impedance system (Minnesota Impedance Cardiograph Model 304A, Instrumentation For Medicine Inc) with four pairs of standard disposable ECG-electrodes placed on the test limb.

Rate of perceived discomfort/pain is assessed by the subject using a ratio scale.

#### Measured parameters

See 6.4.2.2 and 6.4.4.1

#### Description of the test termination criteria of the protocol

See General protocol

#### Specific medical monitoring

ECG and blood pressure are monitored during hyperbaric chamber experiments.

#### Other specific requirements (dietary, conditions of the experiment sessions, etc.)

N/A

#### Biological samples handling, preservation conditions, transport

- Biopsy samples will be divided and processed on site to be shared by several investigators.
- Biopsies will be frozen and labeled and stored and subsequently transported to analyzed at selected laboratories.

#### Hazards, side effects, possible inconveniences, discomfort and constraints to subjects

Subject constraints and potential side effects associated with the bedrest study are described in the general bedrest protocol. The potential risks of involvement in this particular study include: Initially, there might be muscle tightness, soreness, and fatigue following resistance exercise. This potential effect typically subsides after a few sessions. Acute resistance exercise very rarely results in a pulled muscle. Resistance exercise will be supervised ; intensity will be prpogressively increased and always preceded by warm-up. The muscle biopsy procedure may produce light dull pain and delayed soreness that may persist over a couple of days. There is a small risk of bleeding, infection, and scarring of the skin. Temporary numbness of the skin near the biopsy site occurs rarely. Collectively, we have performed thousands of muscle biopsies with no serious complications. Our standrad precautions will minimize any hazardous effects. There is a small risk of a claustrophobic reaction during the MRI scans.

We have conducted four studies (comprising >200 pressure exposures) employing the method to determine blood vessel distensibility described in the present proposal. To ensure that the chamber pressure is not by mistake elevated above safe levels the chamber door is provided with a water lock which releases once pressure reaches 250 mmHg. Increasing the vascular distending pressure in this manner in the leg induces slight discomfort but no pain nor any adverse after effects. Elevation of distending pressure in arm vessels by >120 mmHg is usually accompanied by a pain which abates promptly upon release of pressure.

After the exposure the subject can sometimes experience a slight tenderness over the brachial vessels and/or slight petechial haemorrhages (cf ”G-measles”), both of which will subside within a few days.

### Protocol summary (one page), written on a separate sheet

*Objectives, brief protocol description, main medical risks*

Skeletal muscle function is compromised in response to long-duration orbital flight. Hence, in-flight methods to counteract this effect are warranted. We have developed a mechanical, non-gravity dependent exercise system, which uses the inertia of fly-wheels, to produce resistance. It has been designed for use on the International Space Station (ISS).

This project will study the efficacy of this exercise system, to prevent muscle atrophy and impairment of muscle function which occur during spaceflight. In an effort to simulate weightlessness, healthy men will perform 90 days bedrest. Ten subjects will not train (BR), whereas ten volunteers will perform bilateral resistance exercise (BRE), on the ergometer 2-3 times weekly. Each session consists of four sets of 7 maximal concentric-eccentric knee extensor muscle actions. Before and after BR and BRE the cross-sectional area of individual knee extensor muscles will be measured using magnetic resonance imaging (MRI). In muscle biopsies, obtained from the vastus lateralis and soleus muscles, myosin heavy chain composition and enzyme activities will be assessed. Also, muscle force and electromyographic activity, will be measured during submaximal or maximal knee extensions to assess several aspects of muscle function.

Thus, we should be able to study the effects of resistance training on size, integrity and function of skeletal muscle subjected to a spaceflight analog. If the exercise countermeasure paradigm proves to be effective in preventing muscle atrophy and impaired muscle function in response to 90 days bedrest, we propose fly-wheel technology to be used as an exercise countermeasure on the ISS.

Another purpose is to study the effects of bedrest alone and in combination with resistance exercise on vascular distensibility of the arteries and arterioles of the upper and lower extremity. Orthostatic tolerance is a significant problem after prolonged bedrest and spaceflight, and arterial or arteriolar distensibility may play a role in orthostatic tolerance. It appears reasonable to assume the proposed bedrest regimen will increase arterial/arteriolar distensibility and that resistance exercise will off-set this effect. This project uses established and safely proven techniques. Thus, there are no major medical risks associated with the protocol of this study.

### Investigators

**Principal Investigator:** Pr. P.A. Tesch

**Co-Investigators:**

Dr. O. Eiken

M. T.A. Trappe

Dr. A. Ekberg

M. B. Alkner

M. J.T.Trieschmann

M. G. Nader

M. R. Kölegard

M. B. Lindborg

M. I.B. Mekjavic

## Validation of physical and pharmacological countermeasures to maintain optimal stress responses in astronauts

### Scientific Background

#### Problem presentation

Although space research has clearly evidenced a remarkable tolerance of higher organisms to microgravity even for extended periods of time, the cardiovascular system appears particularly exposed to important stresses both during flights and upon return to ground. Neurohumoral control mechanisms, mostly through the intervention of the autonomic nervous system, are playing a key role in this complex process.

#### Literature data including references

Although space flight is associated to an array of changes in crucial physiological control mechanisms, space research has clearly evidenced a remarkable tolerance of higher organisms to microgravity even for extended periods of time.

The cardiovascular system appears exposed to important stresses both during flights and upon return to ground. During flights, the occurrence of rapid changes in intrathoracic volumes lead to the activation of cardiopulmonary reflexes. On the contrary, upon return to ground, orthostatic intolerance is likely to cause important dysfunction (1-3).

Physical exercise is an important component of the routine work during flight, and could have salutary effects on various microgravity induced disturbances, inclusive of muscle-skeletal impairment and post-flight orthostatic intolerance (4). Accordingly it is considered as a potentially important countermeasure to prevent post microgravity disturbances of cardiovascular regulation.

Neurohumoral control mechanisms, mostly through the intervention of the autonomic nervous system, are playing a key role in this complex process.

Following the seminal study by Akselrod (5) in the last 15 years we have developed a technique (6,7), which employs continuous ECG, arterial pressure and respiration, recordings to obtain quantitative indices of the autonomic balance regulating the SA node, of the sympathetic vasomotor regulation and of baroreflex control of the SA node, as well as of their interrelation with respiration. This technique is based on autoregressive spectral analysis of beat by beat variability, to extract the oscillatory components hidden in the signals. It is important to recall that, as recently remarked by an ad hoc Task Force (8), both parametric and non-parametric spectral analysis techniques provide similar results, when properly employed. However, only a normalization procedure of data obtained with non parametric algorithms, according to a method we originally proposed in 1986 (6), provides an adequate assessment of induced changes in sympatho-vagal balance, as occurs with graded tilt or with intravenous infusions of vasoactive substances. Importantly a tight correlation between oscillatory components of efferent sympathetic nerve activity and similar components of RR interval and systolic arterial pressure variability is present using non parametric methodologies (9), while such a relationship is much weaker with parametric approaches.

We have already employed this approach in two ESA campaigns (11,12), and found it useful to assess in simulated microgravity attendant changes in autonomic cardiovascular control. An additional advantage of this approach is the capacity to be employed in dynamic, real life conditions, using radiotelemetric techniques, at a distance (13) There are no data of this type on real, as opposed to simulated microgravity, such as occurs during space flights.

The opportunity offered by ESA, which is planning organize additional simulated μG experiments, offers a crucial opportunity to plan studies on regulation of blood pressure and to test models of countermeasures for post μG orthostatic hypotension, focusing on changes in neural humoral regulation of the cardiovascular system, in humans.

In addition the exposure to the stress of microgravity might alter hormonal and immunological regulatory systems. In particular hormones of the hypothalamus- pituitary –adrenal (HPA) axis, such as corticotrophin and cortisol, seem to be altered (14-16). This has a potential to alter immunofunction, as assessed by subpopulations lymphocyte studies, or cytokine production, based on saliva samples.

Various drugs, are considered as a possible preventive countermeasures to prevent, or minimize the effects of autonomic disturbances, and the shift of fluids that occurs with the prolonged exposure to microgravity.

This study was designed, bearing in mind the specific aspects of the next ESA prolonged bed rest campaign, that is based on three groups of subjects: no countermeasures, physical countermeasures, and pharmacological countermeasures.The present, bed rest based, solicited proposal applications is a natural, and important, complement to project ST-107, to which the proponent is participating, planning to assess the effects of stress of space flight in Astronauts, during a shuttle flight tentatively scheduled for early 2001.

In conclusion, taking advantage of an ESA sponsored bed rest facility and of the recent related solicited research proposal, we planned the present experiment in order to expand our base knowledge about the integrated adaptation of stress responses and processes to μG.

The information on countermeasures that we will obtain will permit a better planning of future flying mission, inclusive the ST 107 to which these investigators are participating under a combined ESA/ASI sponsorship.

*References:*

1. Convertino VA, Doerr DF, Stein SL. Changes in size and compliance of the calf after 30 days of simulated microgravity. J Appl Physiol 1989; 66: 1509-1512
2. Convertino VA, Doerr DF, Eckberg DL, Fritsch JM, Vernikos-Danellis J. Head-down bed rest impairs vagal baroreflex responses and provokes orthostatic hypotension. J Appl Physiol 1990; 68: 1458-1464
3. Hughson RL, Maillet A, Gharib C, Fortrat JO, Yamamoto Y, Pavy-Le Traon A, Riviere D, Guell A. Reduced spontaneous baroreflex response slope during lower body negative pressure after 28 days of head-down bed-rest. J Appl Physiol 1994; 77: 69-77.
4. Levine BD, Buckey JC, Fritsch JM, Yancy CS, Watenpaugh DE, Snell PG, Lane LD, Eckberg DL, Blomquist CG. Physical fitness and cardiovascular regulation: mechanisms of orthostatic intolerance. J Appl Physiol 1991; 70: 112-122.
5. Akselrod S, Gordon D, Ubel FA, Shannon DC, Barger AC, Cohen RJ: Power spectrum analysis of heart rate fluctuation: a quantitative probe of beat-to-beat cardiovascular control. Science 1981;213:220-222.
6. Pagani M, Lombardi F, Guzzetti S, Rimoldi O. Furlan R, Pizzinelli P, Sandrone G, Malfatto G, Dell'Orto S, Piccaluga E, Turiel M, Baselli G, Cerutti S, Malliani A: Power spectral analysys of heart rate and arterial pressure variabilities as a marker of sympathovagal interaction in man and consciuos dog. Circ Res 1986;59:178-193
7. Malliani A, Pagani M, Lombardi F, Cerutti S. Cardiovascular neural regulation explored in the frequency domain. *Research Advances Series*, Circulation 1991;84:482-492.
8. Task Force of the European Society of Cardiology and the North American Society of Pacing and Electrophysiology. Heart Rate Variability. Standards of Measurements, Physiological Interpretation, and Clinical Use. Circulation 1996;93:1043-1065.
9. Malliani A, Pagani M, Furlan R, Guzzetti S, Lucini D, Montano N, Cerutti S, Mela GS. Individual recognition by heart rate variability of two different autonomic profiles related to posture. Circulation 1997 (in press).
10. Pagani M, Montano N, Porta A, Abboud F, Malliani A, Somers V. The relationship between spectral components of cardiovascular variabilities and direct measures of muscle sympathetic nerve activity in humans. Circulation 1997;95:1441-1448.
11. Pagani M, Iellamo F, Lucini D, Pizzinelli P, Castrucci F, Peruzzi G, Malliani A. Adaptational changes in the neural control of cardiorespiratory function in a confined environment: the CNEC#3 experiment. Acta Astronautica 1995;36:449-461.
12. Pagani M, Iellamo F, Castrucci F, Pizzinelli P, Lucini D, Peruzzi G, Malliani A. Time and frequency domain analysis of neurovegetative control of cardiovascular function during simulated microgravity conditions as induced by prolonged bed-rest in head-down position. ESA-CNES 1994 long-term bed-rest study. Final Report. Toulouse, France, October 1996 p.31 (Abstract N. 3.3).
13. Pagani M, Lucini D, De Bernardi F, Pozzi A, Pizzinelli P, Malliani A: Mental stress. J Ambulat Monit 1992;5:235-244
14. Vernikos J, Dallman MF, Keil LC, O’Hara D, Convertino VA. Gender differences in endocrine responses to posture and 7 days –6 degrees head-down bed rest. Am Physiol 1993;265:E153-61.
15. Macho L, Coll, Acta Astronautica, 1991;23:117-21.
16. Grigoriev AI, ADV Space Bioc Med 1992b;2:43-82.
17. Lucini D, Pagani M, Mela GS, Malliani A: Sympathetic restraint of baroreflex control of heart period in normotensive and hypertensive subjects. Clinical Science 1994;86:547-556.
18. Porta A, Baselli G, Guzzetti S, Magatelli R, Montano N, Cogliati C, Malliani A, Cerutti S. Synchronisation analysis of heart period variability signal based on corrected conditional entropy. Computers in Cardiology 1997, 7-10 September, Lund, Sweden.
19. Mitchell JH, Kaufman MP, Iwamoto GA: The exercise pressor reflex: its cardiovascular effects, afferent mechanisms and central pathways. Ann Rev Physiol 1983; 45:229-242.
20. Pagani M., Lucini D., Rimoldi O., Furlan R., Piazza S., Biancardi L.: Effects of physical and mental exercise on heart rate variability. In: Malik M., Camm A.J eds. Heart Rate Variability. Armonk, NY, Futura Publishing Company, Inc., 1995:245-266.
21. Saul JP, Albrecht P, Berger RD, Cohen RJ. Analysis of long term heart rate variability: methods 1/f scaling and implications. Computers in cardiology, Silver Springs: IEEE Computers Society Press, 1988:419-22.
22. Pagani M, Somers V, Furlan R, Dell' Orto S, Conway J, Baselli G, Cerruti S, Sleight P, Malliani A. Changes in autonomic regulation induced by physical training in mild hypertension. Hypertension 1988; 12: 600-610.

#### Protocol justification according to current scientific knowledge

This project is based and extends previous work performed by the proposer with similar techniques in ground simulation experiments, in two different settings. A first series of experiments has been performed in Germany in isolation experiments (11) and secondly in a series of experiments performed in prolonged head-down bed-rest in France (12). Although other laboratories have addressed similar issues, the uniqueness of our studies derives from the technique we employed. We addressed heart rate and blood pressure variability, using non-parametric autoregressive spectral analysis of beat by beat, RR interval and blood pressure variability (5, 6). This specific approach appeared capable of showing tight correlation between hemodynamic and MSNA spontaneous oscillation, in humans, (10) at variance with previous studies employing parametric methods. This technique demonstrated important influence of isolation on the performance of the autonomic nervous system at the end of the isolation period, and also demonstrated important changes in the orthostatic defense mechanisms during and few days after the end of the microgravity simulation period. It also demonstrated a clear effect of microgravity simulation on the capacity of the system to respond to standardized physical and mental stressors. In addition we will address the effects of μG on the overall information content of the variability signals, using a new method of non linear analysis of the variability signals, that we recently described (i.e. conditional entropy (18). Finally, we will use a new forecasting approach, recently described by our group (9). The co-investigators that will address hormonal and immunological function have an extensive experience of these specific fields.

In addition extensive experience on the effects of exercise and on the development of analytical tools are represented. Finally, a crucial expertise on bioengineering, computer signal analysis and modeling is strategically available.

#### The interest of the duration of the head down bed rest

Head Down Bed Rest has been extensively employed as a mean to mimic major aspect of real microgravity, however prior experiments have been usually performed with relatively short duration protocols.

The predicted duration of forthcoming space activities, such as those that will occur in the space stations, or with future space flights (such as Mars missions), is of the order of such months. Accordingly, it is crucial to gather better knowledge not only on the pathophysiology of long exposure to microgravity, but also on the methods to best counterbalance the occurrence of its effects. Although not directly examined in the present protocol, bone changes might be of great importance; to be considered together with musculoskeletal changes the present protocol, indeed, examines, on a part of the experiment, autonomic effects of exercise.

#### Rationale for drug and dosage selection

Not applicable.

### Objectives and hypothesis

#### Main objective of the protocol

To assess initial and long term, autonomic adaptation to prolonged simulated microgravity, as obtained with head down bed rest (HDBR) in humans, using non-parametric spectral analysis of cardiovascular variability and of respiration. We will additionally address the interaction of various autonomic cardiovascular control mechanisms, considering pre HDBR, HDBR and post HDBR periods.

Specifically we will consider sympatho-vagal interaction modulating the SA node, sympathetic modulation of vasomotion, the arterial pressure heart period baroreflex and total heart rate variability. Finally we will consider non linear dynamics of heart rate variability by way of a new approach to non linear components of the variability signal, i.e. conditional entropy, which provides a normalized measure of overall information content of the signal also on short term data segments (of a few hundreds of beats) (18).

We will also address the capacity of physical training and of pharmacological treatment (still to be defined by ESA) to counteract orthostatic intolerance, and to assess the autonomic mechanisms of this effect.

We will also assess the possible changes in autonomic responsiveness to physical and mental stressors, focusing in particular on light dynamic exercise and standardized mental arithmetic. In addition we will address the changes in responsiveness to orthostatic stimulation before and after exposure to μG.

We will also assess the HPA axis and the citokine profile (TH1/TH2), before doing and after μG, in order to test possible changes in hormonal and immunological functions.

#### Identification of main parameters

*Recorded variables*.

During the tests we will continuously measure the following: 1) RR interval from an ECG lead signal by an high impedance probe, 2) Systolic (SAP), Diastolic (DAP) and Mean arterial pressure (MAP) by Finapres (or Portapres) and 3) Respiration by a piezoelectric pneumobelt, or Respiration Inductance Plethysmography (RIP). Alternatively we will obtain these signals by the available local instrumentation. For the subsequent frequency domain analysis, the analogue signals, after appropriate amplification and filtering, will be sampled at 300 Hz per channel by an A/D board and a portable PC and stored on optical disks.

*Frequency domain analysis*.

Details of the off-line analysis have been already published (15-17). Schematically, from tachogram sections, of adequate length and stationarity (usually 200-500 beats) the computer program automatically calculates simple statistics and the best autoregressive estimate of the power spectral density. The power and frequency of every spectral component are printed out and presented both in absolute units (i.e. [msec²]) and normalized units (i.e. [nu]). Normalized units are computed by dividing the absolute power of a given high-frequency (HF) or low frequency (LF) component by total power (i.e. variance) after having subtracted from it the power of the component with a center frequency near 0 Hz (less than 0.03 Hz) and multiplying this ratio by 100. The analysis of these extremely low-frequency components, which nevertheless may contain important information, requires a different methodology (21) and is not within the scope of the present study. Unless otherwise specified, spectral power will be presented in normalized units (6). Spectral analysis will also be performed on the respiratory signal using a similar procedure. From the simultaneous analysis of arterial pressure and RR interval variability an index (α) can be derived, which is a measure of the overall gain of the arterial pressure-heart period relationship in correspondence of LF and HF oscillatory components (22); an average index is obtained with the formula:

α= [(PR-R/PSAP) LF 1/2+(PR-R/PSAP) HF 1/2] /2

where PR-R and PSAP represent the spectral powers of the RR interval and of the systolic arterial pressure components, respectively. The validity of this calculation requires that the coherence between the two variability signals is greater than 0.5. (22).

Free saliva cortisol and the citokine profile (IL1, IL2,TNFα) will be obtained with ELISA techniques

Corrected entropy will be computed according to (18), from short (about 300-500 beat) data series.

Statistical tests that will be employed comprise the computation of medians and semi interquartile (more appropriate in the case of few subjects, as in the present case), as well as non-parametric ANOVA for repeated measures.

#### Tested hypothesis

1. Exposure to microgravity modifies autonomic (and possibly immuno-hormonal) control mechanisms at rest.
2. The responsiveness to laboratory stressors (physical and mental exercise) is different in subjects that have been exposed to microgravity.
3. These effects are different in subjects that perform exercise as a countermeasure.

#### Expected results

In spite of extensive research on the effects of microgravity on autonomic and hormonal control mechanisms, still considerable uncertainty is present on this matter. This is likely to result from the complexity of the issue and from the difficulty of analysing cardiovascular control with non-invasive approaches. As an example, although it is commonly assumed that carotid baroreceptor function is depressed after exposure to microgravity (1-3), a selective enhancement of aortic baroreflexes has also been reported (17). Since integrated cardiovascular control depends on the summation of various reflex mechanisms (7), the effects of microgravity should probably be addressed with comprehensive (9) as opposed to analytical, approaches. In addition reported hormonal immunological changes are still scanty. Accordingly the proposed comprehensive approach will provide important new information on overall cardiovascular control mechanisms, and on the changes produced by prolonged exposure to microgravity irrespective of the potential confounder of diverging, yet simultaneously operative mechanisms. This approach will provide comprehensive evidence on the mechanisms of action of important potential countermeasures such as physical exercise (19).

#### Secondary objectives

We will also provide new information on the changes in autonomic control in response to stressors such as physical and mental exercise (20) which might further help in selecting tailored countermeasures.

### Specific inclusion and non-inclusion criteria for test subjects

Those employed by ESA and the project apply.

### Precise description of the experimental protocol

#### Tests description (methods, duration, time of day, protocol conditions, etc.)

This study considers periods before, during and after HDBR. In these periods we propose to evaluate in humans the performance of the autonomic nervous system, by employing spectral analysis of cardiovascular variability and of respiration.

Accordingly we will consider:

- resting conditions
- physical stress of moderate dynamic exercise
- mental stress

as standard model components of everyday activity.

In addition we will consider the response to orthostatic stress, as a laboratory model capable to test the gravitational defense mechanisms, relevant to microgravity research. Before and after μG exposure (head down bed rest) we will consider a detailed assessment of autonomic cardiovascular control profile at rest, and the response to orthostatic stress. In addition we will also test the response to moderate exercise as performed with a bicycle ergometer. In addition the response to standardized mental stress test as performed with mental arithmetic will be assessed in all experimental sessions.

During permanence in microgravity, we will assess the progressive changes in resting autonomic conditions and the responsiveness to standardized physical and mental stressors (as obtained with bicycle ergometer and mental arithmetic).

Hormonal/immunological functions will be addressed with saliva samples at the same time windows.

*Phases:*

1. Pre HDBR: (BDC-10, -2) this phase of the study will be mainly devoted to the tuning of the recording procedures to the specific requests of the bed rest scenario, and to the performance of initial short-term experiment, and training of the personnel.
2. During HDBR: (HDT 3, 12, 25, 53, 82, 89) during the bed rest period, it would be important to have as many recordings as possible. We plan to have at least three recordings: early, middle, late HDT.
3. Post HDBR: after exposure to μG, it will be important to perform a full recording as soon as possible (R+2), followed thereafter by recordings at R+9, in order to monitor the recovery process. In a prior study (ref 12) we observed in fact that after as much as 8 days into the recovery period clear alterations in autonomic mechanisms were still present.

*Dates/duration*

The study should be planned according to the schedule of the HDBR. It will be linked with the heart rate variability part of Dr. Ferretti protocol

*Place of training/test/baseline Data Collection*

Training, tests and baseline data collection will take place at MEDES.

#### Procedures

Subjects will be studied at rest and during the following exercise procedures: mental exercise (arithmetic), and light bicycle exercise.

- *Mental arithmetic* consists in having subjects subtract, aloud, a two digit number (usually 13) from a four digit number (usually 2017), with an investigator pressing.
- *Light bicycle exercise* will be performed for 8 minutes at 75 watts, on a horizontal bicycle ergometer before and after HDBR. This protocol will be integrated with other physical exercise protocols. A comment should be dedicated to the potential influence of this small amount of exercise in the non-countermeasures group. According to our previous experience with moderate handgrip (ref 12), this influence should be minimal, if any.
- *Active standing* (performed only Pre and Post HDBR) will consists in having the subjects reach the upright posture, without being aided, and maintain it for approximately 5-7 minutes
- *Immunology*: saliva samples will be obtained in each experimental session, in order to assess TH1/TH2 cytokine production as well as free cortisol. IL2, IL-12, IFN and IL4, IL-10 will be assessed with ELISA and Fluorescence techniques.

Before and after bed rest

|  |  | Timing of experimental procedures |
| --- | --- | --- |
| **Preparation & saliva samples** | |  |
|  | *Setting of electronics,*  *electrode placement* | 5 min |
| **Recording** |  |  |
|  | *Stand* | 5 min |
|  | *Recovery* | 3 min |
|  | *Rest* | 5 min |
|  | *Mental arithmetic* | 5 min |
|  | *Rest* | 3 min |
|  | *Bicycle exercise* | 8min |
| **Removal of electrodes** | | 3 min |
|  |  |  |
| TOTAL |  | 37 min |

During best rest

| **Preparation & saliva samples** | | |
| --- | --- | --- |
|  | *Setting of electronics,*  *electrode placement* | 5 min |
| **Recording** |  |  |
|  | *Rest* | 5 min |
|  | *Mental arithmetic* | 5 min |
|  | *Recovery* | 3 min |
| **Removal of electrodes** | | 3 min |
|  |  |  |
| TOTAL |  | 21 min |

#### Details of Laboratory conditions

In our experiment there is no specific request for conditions such as air composition, humidity, temperature control, and illumination.

Note that the following tasks: stand, mental arithmetic, moderate exercise will have to be harmonized with the general protocol. Stand will be (obviously) performed only before and after HDBR.

#### Measured parameters

During the tests we will continuously measure the following: 1) RR interval from an ECG lead signal by an high impedance probe, 2) Systolic (SAP), Diastolic (DAP) and Mean arterial pressure (MAP) by Finapres (or Portapres) and 3) Respiration by a piezoelectric pneumobelt, or Respiration Inductance Plethysmography (RIP). Alternatively we will obtain these signals by the available local instrumentation.

#### Equipment used (hardware and software description)

During the tests we will continuously measure the following: 1) RR interval from an ECG lead signal by an high impedance probe, 2) Systolic (SAP), Diastolic (DAP) and Mean arterial pressure (MAP) by Finapres (or Portapres) and 3) Respiration by a piezoelectric pneumobelt, or Respiration Inductance Plethysmography (RIP). Alternatively we will obtain these signals by the available local instrumentation.

#### Description of the test termination criteria of the protocol

These tests have been extensively used in several laboratories with no known ill effects on either volunteers or patients. In any case tests will be terminated in the very unlikely occurrence of experimental subjects’ request.

#### Specific medical monitoring (ECG, blood pressure monitoring, etc.)

This study is planning to use the local systems or to rely for the rest of the data acquisition on standard portable PC according to subsequent arrangements. In the case the specific requirement for ECG, respiratory and blood pressure recording could not be met by the local instrumentation (a very unlikely opportunity), we plan to bring over the specific hardware for this purpose.

#### Other specific requirements (dietary, condition of the experiment sessions, etc.)

N/A

#### Biological samples handling, preservation conditions, transport

Dry reagent immunoassay with increased automation has been recently developed to determine important analyses. No reagent preparation or sample pre-treatment is necessary. The sample volume is small, and the analysis is usually completed in few minutes. A miniature analytical element has been developed for the quantitative analysis of salivary and urinary cortisol. Saliva collection will be performed with a plastic vial, that will be immediately stored at least at –20°C. Vials will be subsequently sent in dry ice to Milano, where analysis will be performed.

#### Data utilization and analysis

For the subsequent frequency domain analysis, the analogue signals, after appropriate amplification and filtering, will be sampled at 300 Hz per channel by an A/D board and a portable PC and stored on optical disks.

*Frequency domain analysis*.

Details of the off-line analysis have been already published (15-17). Schematically, from tachogram sections, of adequate length and stationarity (usually 200-500 beats) the computer program automatically calculates simple statistics and the best autoregressive estimate of the power spectral density. The power and frequency of every spectral component are printed out and presented both in absolute units (i.e. [msec²]) and normalized units (i.e. [nu]). Normalized units are computed by dividing the absolute power of a given high-frequency (HF) or low frequency (LF) component by total power (i.e. variance) after having subtracted from it the power of the component with a center frequency near 0 Hz (less than 0.03 Hz) and multiplying this ratio by 100. The analysis of these extremely low-frequency components, which nevertheless may contain important information, requires a different methodology (21) and is not within the scope of the present study. Unless otherwise specified, spectral power will be presented in normalized units (6). Spectral analysis will also be performed on the respiratory signal using a similar procedure.

From the simultaneous analysis of arterial pressure and RR interval variability an index (α) can be derived, which is a measure of the overall gain of the arterial pressure-heart period relationship in correspondence of LF and HF oscillatory components (22); an average index is obtained with the formula:

α= [(PR-R/PSAP) LF 1/2+(PR-R/PSAP) HF 1/2] /2

where PR-R and PSAP represent the spectral powers of the RR interval and of the systolic arterial pressure components, respectively. The validity of this calculation requires that the coherence between the two variability signals is greater than 0.5. (22).

Free saliva cortisol and the citokine profile (IL1, IL2,TNFα) will be obtained with ELISA techniques

Corrected entropy will be computed according to (18), from short (about 300-500 beat) data series.

Statistical tests that will be employed comprise the computation of medians and semi interquartile (more appropriate in the case of few subjects, as in the present case), as well as non-parametric ANOVA for repeated measures.

#### Hazards, side effects, possible inconveniences, discomfort and constraints to subjects

Proposed experimental work is meeting all local human subject requirements for protocol using human subjects. We don’t foresee any hazardous conditions; the only potential risk being the fainting of unaided subjects upon exposure to standing-up in the post μG period.

##### Potential hazard: Skin irritation

a. Potential hazard cause

Due to the application of adhesive ECG electrodes.

b. Effects of the hazard

The adhesive on the ECG electrodes may cause minor skin irritation in some subjects.

c. Assessment: Severity & probability

Severity categories: Minimal

Probability categories: Low

d. Protection to minimize risks

Application of 0.5 - 1% hydrocortisone cream to the affected site.

##### Potential hazard: Electric shock

a. Potential hazard cause

Application of electrical current to the subject from a short in the ECG electronics or through the ECG harnes.

b. Effects of the hazard

Subject injury or death.

c. Assessment: Severity & probability

Severity categories: Reasonable

Probability categories: Extremely low

d. Protection to minimize risks

All electronics are conforming with IEC standards for electromedical equipment at the time of manufacture. In a layman’s term this is regular hospital standard for *e.g.* intensive care.

e. Safety verification methods

A ground check will be performed prior to use.

##### Potential hazard: Orthostatic hypotension

a. Potential hazard cause

Impaired orthostatic tolerance after exposure microgravity.

b. Effects of the hazard

Fall of arterial blood pressure, nausea, fainting.

c. Assessment: Severity & probability

Severity categories: Reasonable

Probability categories: Very low

d. Protection to minimize risks

During the resting stand up procedures the duration of upright posture is limited to less than 10 minutes. There will be continuous monitoring of heart rate and arterial blood pressure. Subjects will be free to rapidly regain the supine posture in case of symptoms of orthostatic intolerance (nausea, pallor, sudden increases or decreases in heart rate, sudden decreases of arterial blood pressure).

##### Mental exercise

Proposed mental arithmetic test requires only a moderate level of attention and does not require any specific preparation. This test has been performed with no difficulty by healthy subjects and by patients, with different kinds of disease, without any evidence of discomfort

##### Bicycle exercise

Proposed workloads of less than 100 watt during sitting will require more than minimum effort, but will not result in fatigue or muscle soreness for the planned durations.

### Protocol summary

Although space research has clearly evidenced a remarkable tolerance of higher organisms to microgravity even for extended periods of time, the cardiovascular system appears particularly exposed to important stresses both during flights and upon return to ground. Neurohumoral control mechanisms, mostly through the intervention of the autonomic nervous system, are playing a key role in this complex process.

A previous non-invasive study we assessed initial, and long term, autonomic adaptation to prolonged simulated microgravity, in humans. We observed important changes in makers of autonomic modulation that were assessed by spectral analysis of ECG derived RR interval, arterial pressure and respiration. This technique provides a quantitative assessment of the autonomic balance regulating the SA node, of the sympathetic vasomotor regulation and of baroreflex control of the SA node, as well as of their interrelation with respiration. This approach has recently been endorsed by an ad hoc Task Force (7), and is the only one that is also supported by direct validation experiments based on microneurographic recordings of sympathetic activity in humans.

We observed not only changes in rest values of autonomic markers, but also a complex modification of responses to standardized stimuli**.**

In this simulated microgravity study, we will expand prior observations, by considering as well hormonal and immunological homeostasis. In addition we will assess a whether physical (exercise) and pharmacological countermeasures will be able to counter act the microgravity induced alterations of autonomic control.

This study will provide the first comprehensive evaluation of prolonged microgravity on simultaneous markers of autonomic, hormonal and immunological homeostasis, in addition it will provide an assessment of the potentially beneficial effects of physical and pharmacological countermeasures.

### Investigators

**Principal Investigators:** Pr. Massimo Pagani

**Co-Investigators**:

Dr. Cerchiello M.

Dr. Custolo de Rosis A.

Dr. Iellamo F.

Dr. Pizzinelli P.

## Effects of a long-term bed rest on muscle integrity and cytoskeletal proteins, with and without muscle exercise countermeasures.

### Scientific Background

#### Problem presentation

The modifications of skeletal muscle morphology and functions likely to happen during long duration spaceflight in absence of countermeasures, are considered to represent a significant problem for some important reasons : the resulting muscle atrophy and weakness can increase the fatigability of the astronauts, decrease their work capacity, and impair their well-being and health.

Furthermore, the modifications of the normally imposed stimuli on the neuromuscular system resulting from spaceflights constitute an experimental situation which is promising for the improvement of our knowledge of muscle plasticity.

It is well established that bed rest is a good way to simulate the physiological effects of microgravity, and especially the loss of mass and strength of skeletal muscles. It was classically demonstrated that muscle atrophy is the most important aetiology of this loss of strength and mass of the muscles either during spaceflight (Cherepakhin and Pervushin, 1971; Thornton and Rummel, 1977; Koslovskaia et al., 1981) or bed rest (Grogoriev and Koslovskaia, 1988; Hikida et al., 1989; Leblanc et al., 1988).

Modifications of protein turnover are one of the main mechanisms involved in muscle atrophy in animals and humans (Steffen and Musacchia, 1984, 1986; Goldspink et al., 1986; Ferrando et al., 1996). As neuromuscular activity is an important stimulus able to modify muscle protein turnover, it is not surprising that general conditions of confinement or hypokinesia result in muscle atrophy.

Skeletal muscle fibers, because of force generation and external loading, must withstand mechanical constraints exerted on their longitudinal and transversal axis (20, 36). The intensity, frequency, and duration of muscle loading depend on the muscle functions. These variable mechanical constraints cause structural differences in the myotendinous junction (MTJ) (38); Zamora and Marini, 1988; Roffino et al., 1998; Carnino et al., 2000) and in Z lines (39) on the longitudinal axis of slow (type I) and fast (type II) muscle fibers. But little is known about functional consequences on the transversal cytoskeletal organization. Besides its functional role during force transmission, the cytoskeleton, managing the forces generated or supported in the transversal and longitudinal axis of the muscle fiber, is able to protect the muscle fiber from contraction induced damages.

There are many requirements for countermeasures to space deconditioning (Nicogossian et al., 1994; Greenleaf et al.,1989 and 1997; Convertino, 1996). Pharmacological or nutritional manipulations, and physical exercise could play an important role in the program of countermeasures. No single training method nor exercise devices can produce amelioration in both the musculo-skeletal, cardiovascular, neuro-humoral functions, fluid-electrolyte metabolism, physical fitness and work capacity in flight, as well as in the readaptation processes upon re-entry on Earth. Nevertheless, concerning specifically the skeletal muscle deterioration, heavy resistance exercise of some sort must be used in the aim to prevent, to decrease or to compensate the impairment of muscle structure and functions during extended human spaceflight. This kind of exercise was recently proposed as a countermeasure ( Berg and Tesch, 1994; Prou and Marini, 1997) and shown to be efficient during bed rest (Germain et al, 1995; Bamman et al., 1998; Ferrando et al., 1997). In this way the relatively time consuming exercise programs can be partly avoided (Bonde-Petersen et al., 1991).

Mechanical stimuli appears to be an important factor in regulating muscle mass (Goldberg et al., 1975), and the stretching of the muscle fiber is also known to stimulate protein turnover (Vandenburgh and Kaufman, 1980; Loughna et al., 1986). In so far, physical exercise appears to compensate partly the changes resulting from hypokinesia, but it must be also reminded that exercise workloads and over-training may negatively affect the physiological, psychological and behavioral response to exercise in space. Muscle impairment consecutive to long duration spaceflights associated with physical exercise may thus promote muscle damage occurrence (Prou and Marini, 1997; Riley et al., 1998).

#### Literature data

- Berg H.E., Tesch P.A. (1994). A gravity independant ergometer to be used for resistance training in space. Aviat Space Environ Med 65:752-756.
- Bonde-Petersen F., Tallarida G., Marini J.F. (1991). Study on muscular exercise as countermeasure for EMSI. ESA Study 9144/90/F/BZ. Final Report.
- Carnino A., Roffino S., Chopard A., Marini J.F. (2000). Effects of a 14-day spaceflight on soleus myotendinous junction ultrastructure in Rhesus Monkey. J Gravit. Physiol. 7(1):S65-S68.
- Cherepakhin M.A., Pervushin V.I. (1971). Space flight effect on the neuromuscular system of cosmonauts. Space Biol. Med. 4 (6) : 64-69.
- Chopard A., Pons F., Marini J.F. (2000). Cytoskeletal protein contents before and after hindlimb suspension in a fast and slow rat skeletal muscle. Am. J. Physiol. (in press).
- Chopard A., Leclerc L., Pons F., Leger J.J. and J.F. Marini. (2000) Effect of 14-day spaceflight on myosin heavy chain expression in biceps and triceps muscles of Rhesus monkey. J. Gravit. Physiol. 7(1):S47-S49.
- Convertino V.A. (1996). Exercise as a countermeasure for physiological adaptation to prolonged spaceflight. Med Sci Sports Exerc 28: 999-1014.
- Ferrando A.A., Lane H.W., Stuart C.A., Davis-Street J., Wolfe R.R. (1996). Prolonged bed rest decreases skeletal muscle and whole body protein synthesis. Am J Physiol ; 270(4 Pt 1):E627-E633.
- Ferrando A.A., Tipton K.D., Bamman M.M., Wolfe R.R. (1997). Resistance exercise maintains skeletal muscle protein synthesis during bed rest. J Appl Physiol ; 82(3):807-810.
- Germain P., Güell A., Marini J.F. (1995). Muscle strength during bed rest with and without muscle exercise as a countermeasure. Eur J Appl Physiol 71: 342-348.
- Goldberg A.L., Etlinger J.D., Goldspink, D.F., Jableki C. (1975). Mechanism of work- induced hypertrophy of skeletal muscle. Med. Sci. Sports. 7: 248-261.
- Goldspink D.F., Mortin A.J., Loughana P., Goldspink G. (1986). The effect of hypokinesia and hypodynamia on protein turnover and the growth of four skeletal muscles of the rat. Pfluegers Arc. 407 : 333-340.
- Greenleaf J.E., Bernauer E.M., Ertl A.C., Trowbridge T.S., Wade C.E. (1989). Work capacity during 30 days of bed rest with isotonic and isokinetic exercise training. J Appl Physiol 67: 1820-1826.
- Greenleaf J.E., Van Beaumont W., Convertino V.A., Starr J.C. (1983). Handgrip and general muscular strength and endurance during prolonged bed rest with isometric and isotonic leg exercise training. Aviat Space Environ Med 54: 696-700.
- Grigoriev A.I. Kozlovskiaia I.B. (1988). Physiological responses of skeletomuscular system to muscle exercises under long-term hypokinetic conditions. The Physiologist 31(1) Suppl. : 93-97.
- Hikida R.S., Gollnick P.D., Dudley G.A., Convertino V.A., Buchanan P. (1989). Structural and metabolic characteristics of human skeletal muscle following 30 days of simulated microgravity. Aviat Space Environ Med 60(7): 664-670.
- Kozlovskaya I.B., Kreidich Y.U.V., Rakhmanov A.S. (1981). Mechanisms of the effects of weightlessness on the motor system of man. The physiologist, Vol. 24 (6) Suppl.
- LeBlanc A.D., Schneider V.S., Evans H.J., Pientok C., Rowe R., Spector E. (1992). Regional changes in muscle mass following 17 weeks of bed rest. J Appl Physiol 73(5): 2172-2178.
- Loughna P., Goldspink K.G., Goldspink, D.F. (1986). Effect of inactivity and passive strech on protein turnover in phasic and postural rat muscles. J. Appl. Physiol. 61 (1): 173-179.
- Monti R.J., Roy R.R., Hodgson J.A., Edgerton V.R. (1999). Transmission of forces within mammalian skeletal muscles. J. Biomech. 32: 371-380.
- Nicogossian A.E. (1994). Countermeasures to space deconditionning, in Nicogossian A.E., Leach-Hunton C., Pool S. (eds.). Space Physiology and Medecine. Philadelphia, London, Lea & Febiger, pp 447-467.
- Prou E., Marini J.F. (1997). Muscle research in space. Increased muscle susceptibility to exercise-induced muscle damage after a prolonged bed rest. Int. J. Sports Med. 18: 317-320.
- Riley D.A., Bain J.L., Thompson J.L., Fitts R.H., Widrick J.J., Trappe S.W., Trappe T.A., Costill D.L. (1998). Disproportionate loss of thin filaments in human soleus muscle after 17-day bed rest. Muscle Nerve ; 21(10): 1280-1289.
- Roffino S., Carnino A., Charpiot P., Marini J.F. (1998). Increase in rat soleus myotendinous interface after a 14-day spaceflight. C. R. Acad. Sc. (III) 321: 557-564.
- Steffen J.M., Musacchia X.J. (1984). Effect of hypokinesia and hypodynamia on protein, RNA, and DNA in rat hindlimb muscles. Am. J. Physiol. 247: R728-732.
- Steffen J.M., Musacchia X.J. (1986). Spaceflight effects on adult rat muscle protein nucleic acids and amino acids. J. Physiol. 251(6) part 2.
- Thornton W.E., Rummel J.A. (1977). Muscular deconditioning and its prevention in space flight. In : Biomedical results from Skylab, edited by Johnson, R.S., Dietlein, L.F. Washington, DC : NASA, 191-197, (NASA SP-377).
- Tidball J.G. (1991). Force transmission across muscle cell membranes. J. Biomech. 24 Suppl 1: 43-52.
- Trotter J.A., Baca J.M. (1987). A stereological comparison of the muscle-tendon junctions of fast and slow fibers in the chicken. Anat. Rec. 218: 256-266.
- Vandenburgh H., Kaufman S. (1980). Protein degradation in embryonic skeletal muscle. Effect of medium, cell type, inhibitors, and passive stretch. J Biol Chem. 255(12):5826-5833.
- Vigoreaux J.O. (1994). The muscle Z band : lessons in stress management. J. Musc. Res. Cell Motil. 15: 237-255.
- Zamora A.J., Marini J.F. (1988). Tendon and myotendinous junction in an overload skeletal muscle of the rat. Anat. Embryol. (Berl.) 179: 89-96.
- Berg H.E., Tesch P.A. (1994). A gravity independant ergometer to be used for resistance training in space. Aviat Space Environ Med 65:752-756.
- Bonde-Petersen F., Tallarida G., Marini J.F. (1991). Study on muscular exercise as countermeasure for EMSI. ESA Study 9144/90/F/BZ. Final Report.
- Carnino A., Roffino S., Chopard A., Marini J.F. (2000). Effects of a 14-day spaceflight on soleus myotendinous junction ultrastructure in Rhesus Monkey. J Gravit. Physiol. 7(1):S65-S68.
- Cherepakhin M.A., Pervushin V.I. (1971). Space flight effect on the neuromuscular system of cosmonauts. Space Biol. Med. 4 (6) : 64-69.
- Chopard A., Pons F., Marini J.F. (2000). Cytoskeletal protein contents before and after hindlimb suspension in a fast and slow rat skeletal muscle. Am. J. Physiol. (in press).
- Chopard A., Leclerc L., Pons F., Leger J.J. and J.F. Marini. (2000) Effect of 14-day spaceflight on myosin heavy chain expression in biceps and triceps muscles of Rhesus monkey. J. Gravit. Physiol. 7(1):S47-S49.
- Convertino V.A. (1996). Exercise as a countermeasure for physiological adaptation to prolonged spaceflight. Med Sci Sports Exerc 28: 999-1014.
- Ferrando A.A., Lane H.W., Stuart C.A., Davis-Street J., Wolfe R.R. (1996). Prolonged bed rest decreases skeletal muscle and whole body protein synthesis. Am J Physiol ; 270(4 Pt 1):E627-E633.
- Ferrando A.A., Tipton K.D., Bamman M.M., Wolfe R.R. (1997). Resistance exercise maintains skeletal muscle protein synthesis during bed rest. J Appl Physiol ; 82(3):807-810.
- Germain P., Güell A., Marini J.F. (1995). Muscle strength during bed rest with and without muscle exercise as a countermeasure. Eur J Appl Physiol 71: 342-348.
- Goldberg A.L., Etlinger J.D., Goldspink, D.F., Jableki C. (1975). Mechanism of work- induced hypertrophy of skeletal muscle. Med. Sci. Sports. 7: 248-261.
- Goldspink D.F., Mortin A.J., Loughana P., Goldspink G. (1986). The effect of hypokinesia and hypodynamia on protein turnover and the growth of four skeletal muscles of the rat. Pfluegers Arc. 407 : 333-340.
- Greenleaf J.E., Bernauer E.M., Ertl A.C., Trowbridge T.S., Wade C.E. (1989). Work capacity during 30 days of bed rest with isotonic and isokinetic exercise training. J Appl Physiol 67: 1820-1826.
- Greenleaf J.E., Van Beaumont W., Convertino V.A., Starr J.C. (1983). Handgrip and general muscular strength and endurance during prolonged bed rest with isometric and isotonic leg exercise training. Aviat Space Environ Med 54: 696-700.
- Grigoriev A.I. Kozlovskiaia I.B. (1988). Physiological responses of skeletomuscular system to muscle exercises under long-term hypokinetic conditions. The Physiologist 31(1) Suppl. : 93-97.
- Hikida R.S., Gollnick P.D., Dudley G.A., Convertino V.A., Buchanan P. (1989). Structural and metabolic characteristics of human skeletal muscle following 30 days of simulated microgravity. Aviat Space Environ Med 60(7): 664-670.
- Kozlovskaya I.B., Kreidich Y.U.V., Rakhmanov A.S. (1981). Mechanisms of the effects of weightlessness on the motor system of man. The physiologist, Vol. 24 (6) Suppl.
- LeBlanc A.D., Schneider V.S., Evans H.J., Pientok C., Rowe R., Spector E. (1992). Regional changes in muscle mass following 17 weeks of bed rest. J Appl Physiol 73(5): 2172-2178.
- Loughna P., Goldspink K.G., Goldspink, D.F. (1986). Effect of inactivity and passive strech on protein turnover in phasic and postural rat muscles. J. Appl. Physiol. 61 (1): 173-179.
- Monti R.J., Roy R.R., Hodgson J.A., Edgerton V.R. (1999). Transmission of forces within mammalian skeletal muscles. J. Biomech. 32: 371-380.
- Nicogossian A.E. (1994). Countermeasures to space deconditionning, in Nicogossian A.E., Leach-Hunton C., Pool S. (eds.). Space Physiology and Medecine. Philadelphia, London, Lea & Febiger, pp 447-467.
- Prou E., Marini J.F. (1997). Muscle research in space. Increased muscle susceptibility to exercise-induced muscle damage after a prolonged bed rest. Int. J. Sports Med. 18: 317-320.
- Riley D.A., Bain J.L., Thompson J.L., Fitts R.H., Widrick J.J., Trappe S.W., Trappe T.A., Costill D.L. (1998). Disproportionate loss of thin filaments in human soleus muscle after 17-day bed rest. Muscle Nerve ; 21(10): 1280-1289.
- Roffino S., Carnino A., Charpiot P., Marini J.F. (1998). Increase in rat soleus myotendinous interface after a 14-day spaceflight. C. R. Acad. Sc. (III) 321: 557-564.
- Steffen J.M., Musacchia X.J. (1984). Effect of hypokinesia and hypodynamia on protein, RNA, and DNA in rat hindlimb muscles. Am. J. Physiol. 247: R728-732.
- Steffen J.M., Musacchia X.J. (1986). Spaceflight effects on adult rat muscle protein nucleic acids and amino acids. J. Physiol. 251(6) part 2.
- Thornton W.E., Rummel J.A. (1977). Muscular deconditioning and its prevention in space flight. In : Biomedical results from Skylab, edited by Johnson, R.S., Dietlein, L.F. Washington, DC : NASA, 191-197, (NASA SP-377).
- Tidball J.G. (1991). Force transmission across muscle cell membranes. J. Biomech. 24 Suppl 1: 43-52.
- Trotter J.A., Baca J.M. (1987). A stereological comparison of the muscle-tendon junctions of fast and slow fibers in the chicken. Anat. Rec. 218: 256-266.
- Vandenburgh H., Kaufman S. (1980). Protein degradation in embryonic skeletal muscle. Effect of medium, cell type, inhibitors, and passive stretch. J Biol Chem. 255(12):5826-5833.
- Vigoreaux J.O. (1994). The muscle Z band : lessons in stress management. J. Musc. Res. Cell Motil. 15: 237-255.
- Zamora A.J., Marini J.F. (1988). Tendon and myotendinous junction in an overload skeletal muscle of the rat. Anat. Embryol. (Berl.) 179: 89-96.

#### Protocol justification according to current scientific knowledge

In order to determine the best procedure to induce the major changes expected from the use of muscular exercise, a good understanding of the effects of mechanical unloading on muscles and muscle fibers during spaceflights or bed rest are of great importance.

In these perspectives, according to current scientific knowledge indicated in §6.6.1.1 & § 6.6.1.2, we propose to study the adaptative phenomenons which, at the cellular level, induce muscle fiber adaptation to mechanical unloading with or without muscle exercise or pharmacological - nutritional manipulations. This research proposal is designed to a better understanding of the effects of modified mechanical impact or strain on human muscular cells, with respect to transduction signaling pathways involved in the early stages of muscle plasticity mechanisms (including apoptotic pathway) and particularly the crucial role played by the cytoskeleton in the maintenance of muscle integrity. We propose to correlate this study with an investigation of muscle susceptibility to exercise-induced damage before, during, and after a long term bed rest , with and without exercise and pharmacological manipulations.

#### The interest of the duration of the head-down bed rest has also to be justified, in particular for bone changes.

Since bed rest was previously shown to correspond to the best ground situation which permits to evaluate the effects of muscle unloading on humans and because spaceflights of several months are to date currently envisaged, it is now particularly relevant to determine the impacts at muscle cellular level of a long term duration on unloaded phasic and postural muscles or submitted to a putative compensatory exercise measures. Particularly, the changes in cytoskeletal protein contents or expression pattern and localization for such long duration or the molecular processes involved in the disorganization and the reorganization of the muscle fibers resulting from atrophy are not known, and their study will bring information on the relationship between muscle fiber atrophy and possible weakening or degeneration.

### Objectives and hypothesis

#### Main objective of the protocol

As indicated, we propose to study, at the cellular level the adaptative phenomenons, inducing muscle fiber adaptation to mechanical unloading with or without muscle exercise, with respect to the role of the cytoskeleton in muscle integrity maintenance.

#### Identification of main parameters

In order to study the relationship between muscle fiber atrophy and possible weakening or degeneration, we will first consider the changes in cytoskeletal protein contents or expression pattern and localization. Then, we will investigate the molecular processes involved in the disorganization and the reorganization of the muscle fibers resulting from atrophy consecutive to a reduction of the load imposed on muscle during long-term bed rest, and we will also examine these processes during the subsequent activity recovery.

We have planned to investigate the induced changes onto both a slow (SOL) and a faster muscle type (VL) at 3 subcellular levels:

- the sarcomeric and myofibrillar filamentous system looking to the elastic, the Z-line, and the A-band components.
- the membrane-integrated proteins related to muscle fiber cytoskeleton, i.e. proteins from DGC and costameres.
- a muscular enzymatic system- i.e calpain 3, as well as one of a known cytoskeletal calpain substrate : filamin (located at the Z line periphery) will also be taken in consideration.

Moreover, we will examine the DNA status of atrophic fibers as the activation and proliferation of satellite cells, in the aim to determine the contribution of these apoptosis and fusion events to the specific atrophy and repairing mechanisms.

#### Tested hypothesis

We would like to confirm the contribution of human muscle specific cytoskeletal key elements and of programmed cell death mechanisms in unloading induced atrophy. Moreover, by following relevant indicators of muscle integrity, we will evaluate the pertinence of an heavy resistance exercise applied in a prophylactic way in the aim to decrease or to prevent muscular microgravity-associated disorders.

#### Expected results

For the first time realized on human biopsies, this study will allow us :

- To determine the changes in cytoskeletal protein contents resulting from a reduction of the load imposed on muscle (electrophoresis, immunoblotting, densitometry)
- To study the effects of the modifications of mechanical constraints upon the remodeling and the expression of the membrane proteins complexes involved in muscle fiber cohesion (ultra-immuno-microscopy).
- To determine whether or not the muscular exercises used for testing and for muscle exercise or pharmacological treatments as countermeasures are deleterious on muscle after a long duration bed rest
  - to compare muscle susceptibility to exercise-induced damage before and after a prolonged bed rest.
  - to study the innocuousness of heavy resistance exercise with respect to these parameters.

This study will need to be done during a prolonged exposure to simulated microgravity, and blood concentration of Creatine Kinase (CK), Myosin Heavy Chain (MHC), Troponin and eventually Titin were chosen as indicators of cellular impairment (RIA and ELISA).

To relate cytoskeletal proteins changes with other structural, physiological, mechanical and biochemical changes.

#### Secondary objectives

The obtained results will allow us to improve the design of the muscular exercise programs and exercisers dedicated to counteract the deleterious effects of spaceflights in muscle structure and function.

### Specific inclusion and non-inclusion criteria for test subjects

No particular test needed

### Precise description of the experimental protocol

#### Tests description

Cf P. Tesch's project for description of muscle testing and exercise programs

#### Measured parameters

On muscle biopsies : the contents in sarcomeric and myofibrillar filamentous system (elastic, the Z-line, and the A-band proteins) components, and in the membrane-integrated proteins related to muscle fiber cytoskeleton ( proteins from DGC and costamers), and the activity of a muscular enzymatic system (i.e calpain 3), as well as one of a known cytoskeletal calpain substrate (filamin), and, finally, the DNA status of atrophic fibers will be examined (electrophoresis and immunocytochemistry).

On blood samples : blood concentration of Creatine Kinase (CK), Myosin Heavy Chain (MHC), Troponin and eventually Titin will be examined (RIA and ELISA).

#### Methods, equipment and place of analysis

BLOOD SAMPLES (1ml each, for the 3 groups) :

In the aim to evaluate muscle impairment by the dosage of muscle enzymes and structural proteins on exercise, pharmacological and control groups, the sampling timing is adjusted onto the physical tests (during the pre- and post-bed rest periods) and the exercise sessions defined.

- The method used is described in the general purposes of the global LTBR protocol.
- The freezing of the samples will immediately be done in the Space Clinic at MEDES.
- The analysis of the samples will be done at Nice, after dry ice transport.

MUSCLE BIOPSIES :

- The needle biopsy procedure is described in P. Tech's project.
- The needed sample is of 50 mg and frozen slices will be shared from it for both Marini's and Tesch's immunolabelling experiments, which will allow to reduce the total size of the needed biopsies.

The analysis scheme is listed below :

The muscle biopsies needed to be frozen in isopentane and liquid nitrogen. We will manipulate the samples in our laboratory.

We will then perform cytoskeletal proteins analysis (onto 10 mg of muscle) and will measure by dosage the total protein content, according to a protocol we have determined in the laboratory. We will repeat this extraction 3 times to compare the results.

After electrophoretic migration of the extracts, we will proceed in an immunodetection by chimiluminescence, after transfer of the gel onto a PVDF membrane.

We will then quantify the corresponding signals for several members of 3 distinct muscle subcompartments, i.e. dystrophin, and its associated proteins, desmin, filamin, actin, myosin, vinculin, alpha-actinin, titin, calpain 3

If enough tissue is available, we envisage to extend the immunofluorescence on sections to the labelling of nucleus by the TUNEL method (apoptosis detection) and to the labelling of the proteins which were seen to be affected by the LTBR, after our quantitative measurements.

#### Labelling, storage and packaging of the samples

The samples will be stored at -80°C and transported in dry ice.

#### Statistical tests and software used

The cytoskeletal proteins expression will be measured on both gels and autoradiographic films after immunoblotting by using a scanner densitometer with a resolution of 1200 ppi. The digitized images will be quantitatively analyzed using a specific software, Phoretix 1D (Phoretix International, Newcastle upon tyne, UK). The optical density (OD) of a protein band in a sample will be expressed as relative optical density (ODr) according to the previously calibrated amount of protein loaded into the corresponding well.

The quantification of blood parameters will be realized by ELISA or RIA, with systematic blank and control wells in order to take into account non specific background or absorption. In each case, the measurements of each protein followed will be realized in triplicate, and the results will be expressed as mean ± standard deviation (SD). Finally, for each protein relative content in both muscle biopsies and blood samples, the two groups (with and without exercise) will be compared by Mann Whitney test. *P* values ≤ 0.05 will be considered statistically significant.

#### Hazards, side effects, possible inconveniences, discomfort and constraints to subjects

The possible, but reduced and of low probability discomforts resulting from needle biopsies are indicated in P. Tesch's project.

### Protocol summary (one page), written on a separate sheet

It is now well established that bed rest is a good way to stimulate the physiological effects of microgravity, and especially the loss of skeletal muscles mass and strength. It was demonstrated that muscle atrophy is the most important aetiology of this loss of both muscular strength and mass during spaceflight or bed rest. In that context, physical exercise was shown to compensate partly the changes resulting from hypokinesia, while it also could in another way promote muscle damages occurrence. Thus, it is of a particular interest to evaluate the effects of a long term bed rest with and without exercise or pharmacological treatments as coutermeasures. In this perspective, we propose to follow the adaptative phenomenons, inducing muscle fiber adaptation to mechanical unloading with and without muscle exercise. Muscle specific cytoskeleton assumes an essential role in fiber cohesion, cell integrity maintenance and force transmission by linking together several muscular compartments. We have previously developed a quantitative method suitable for human muscle biopsies analysis, and we have demonstrated that cytoskeletal proteins expression levels could largely contribute to muscle fiber adaptative changes resulting from modified functional demands. We thus propose to follow the specific muscle changes induced by the modifications of mechanical constraints in a long term bed rest with and without muscle exercise on both muscle biopsies and blood samples. For each subject, we will examine the expression, localization and/or degradation of several pertinent proteic indicators of muscle cytoskeletal integrity by using specific and quantitative immunodetection methods. This study will also permit to compare muscle susceptibility to putative exercise-induced damages before and after a prolonged bed rest, and to evaluate the innocuousness of heavy resistance exercise in the same conditions. The results of these experiments which are with no medical risk will thus not only contribute to enlarge our knowledge of muscle physiology and plasticity, but will overall permit an efficient exercise prescription adapted to an optimized maintenance of muscle functions during long-term exposure to microgravity.

#### Muscle exercise

Cf projects from others PIs.

Need to adapt our protocol to these constraints

#### Blood sampling

Blood samples will be collected (1 ml each) for :

-a baseline reference : a first sample for each subject will be taken before all the tests planned during the pre-bed rest period (BDC-15)

-the study of the effects of any muscular test or exercise on muscles during initial (pre-bed rest) and recovery muscles ( post-bed rest) periods:

at BDC –13, the sampling will be done just before the first muscular test and 2, 24, and 48 hours after (i.e. respectively at BDC –13, -12,-11) for the samplings of second pre-bed rest period (BDC-7) and the recovery period (R+1, R+8), they will be done just before the first physical test and 2, 24, 48 and 72 hours after (respectively BDC-7,-6,-5,-4 ; R+1,+2,+3, +4 ; and R+8,+9,+10,+11)

* One additional sample will be taken at the end of the first recovery day, 2 hours after the last physical test planned

- muscle homeostasis and/or alteration related to atrophy, during the bed rest period for the groups with and without exercise, the sampling will be done at HDT 16, 58, 78 on the same scheme : just before exercise and 2, 24, 48 hours after.

As indicated before we intend to determine whether or not the muscular exercises used for testing and for muscle exercise as a countermeasure are deleterious on muscle after a long duration bed rest.

We will compare muscle susceptibility to exercise-induced damage before and after a prolonged bed rest, and study the inocuity of heavy resistance exercise during bed rest. We will use serum concentration of Creatine Kinase (CK), Myosin Heavy Chain (MHC, types I and II), Troponin I and eventually titin as indicators of cellular impairment, using RIA and ELISA methods.

#### Muscle biopsies

Muscular biopsies will be taken on muscle vastus lateralis and in the soleus, as soon as possible before and after the bed rest.

Small open biopsies or needle biopsies (open would be better). Sample size about 30 to 50 mg. First biopsy: as long as possible before the bed rest or in the very last hours coming before the bed rest (in order to perturb as less as possible the other experiments). Second biopsy as short as possible before (if it is necessary to perturb as less as possible the other experiments) or after the end of the bed rest.

As indicated in chapter 1, we have planned to investigate the induced changes onto both a slow (SOL) and a faster muscle type (VL) at 3 subcellular levels:

- the sarcomeric and myofibrillar filamentous system looking to the elastic, the Z-line, and the A-band components.

- the membrane-integrated proteins related to muscle fiber cytoskeleton, i.e. proteins from DGC and costameres

- a muscular enzymatic system- i.e calpain 3, as well as one of a known cytoskeletal calpain substrate : filamin (located at the Z line periphery) will also be taken in consideration.

Moreover, we will examine the DNA status of atrophic fibers as well as the activation and proliferation of satellite cells in the aim to determine the contribution of apoptosis and fusion to these specific atrophy and repairing mechanisms.

Several polyclonal affinity purified antibodies directed against titin, filamin, C-protein and calpain 3, as well as monoclonal antibodies labelling dystrophin and its associated proteins, desmin, vinculin, alpha-actinin will be used for immunodetection by quantitive immunoblotting, immunofluorescence with confocal microscopy analysis. We will realize the immunocytochemical study on biopsies cryofixed by the physical method of fixation. Within the selected zone, serial sections (8 to 20µm thick) will be cut. The analysis of the immunological responses will relate to the types and the localization of the different proteins.

### Investigators

**Principal Investigators:** Pr. J.F. Marini

**Co-Investigators**:

Dr. Astier C.

Mr. Carino A.

Mlle Chopar A.

Me LAPEYSSONIE F.

Mlle Roffino S.

## Effects of posture on drug absorption mechanism : Pharmacokinetics of acetaminophen used as a probe to study oral absorption in simulated weightlessness.

### Scientific Background

#### Problem presentation

Drugs are necessary in Space to treat ailments that are common to humans on Earth as well as disturbances caused by the Space environment (pharmacological countermeasures). The pharmacokinetics of drugs is determined by the combination of several complex phenomena. Each step of this process may be influenced by physiological changes occurring in Space flight. The most important changes need to be identified and then predicted for the main drugs. The use of a drug as a probe will permit to estimate the changes in specific pharmacokinetics parameters. In a previous study, we showed that availability of promethazine after oral absorption was increased by 30% in simulated weightlessness (-6° head-down tilt) compared to sitting position. We propose to use acetaminophen (1g) as a probe in order to study the influence of simulated weightlessness on oral absorption. This can be performed four times during long duration Head Down Bed Rest (HDBR) (before the experiment and after 1 day, two weeks, and three months) in order to assess the possible changes in pharmacokinetics during a long duration flight. In order to precise influence of weightlessness on absorption this study will be completed by gastric emptying evaluation (echographic measurement) performed at the same time and by oro-caecal transit time measurement (breath hydrogen test).

#### Literature data including references

Although the oral route is the best accepted by patients and therefore the most usual route for drug administration, it is also the most complicated one in terms of the physiological steps involved. Thus, only the amount reaching the circulation will be able to produce pharmacological effects. Absorption corresponds to the appearance of the drug and/or its metabolites in the circulation from the site of its administration. Absorption from the gastrointestinal tract is governed by several factors, three of which are more important : drug release in the stomach, dissolution of the drug in the gastro-intestinal fluids and gastric emptying. Gastric emptying is rate limiting in the absorption of many orally administered drugs because the intestine is the main site of drug absorption due to its large surface area and the long residence time. Consequently, some changes in gastric-emptying may delay the gastro-intestinal transit and may alter the oral bioavailability of drugs (Houin, 1990).

Gastric emptying is known to be strongly influenced by the orientation of the body. As compared with the left lateral decubitus position which delays gastric emptying and thereby drug absorption, the right lateral decubitus position increases the rate of gastric emptying (Burn-Murdoch, 1980). Acetaminophen absorption in healthy volunteers is faster during sitting position than in lying position. (Rumble, 1991). Nifedipine absorption is faster in standing or lying on the right side compared with lying on the left. (Renwick, 1992).

Both physiological and biochemical changes have been reported to occur during space-flight. These changes are important and may alter the pharmacokinetic properties of pharmacological agents administered in space. Each step of this process may be influenced by weightlessness whatever the route of administration : absorption, distribution, metabolism and elimination. (Lathers et al, 1989 ; Pavy-Le Traon et al, 1994 ; Saivin et al, 1997). In microgravity, gastric emptying may be influenced by the absence of drug or meal mass due to the lack of gravity effect. The absence of gravity could have a strong effect since in the gastric emptying process the likelihood that a certain particle in the stomach passes through the pylorus is a random-chance phenomenon. In microgravity, therefore, these alterations in gastric emptying could lead to delay the gastro-intestinal transit and to erratic plasma levels (Amidon et al, 1991). However, no study has investigated the effects of weightlessness on gastric emptying. Some studies have shown that physiological changes due to weightlessness may induce changes in pharmacokinetic behaviour of drugs and influence their dosage regimen. Inflight data obtained by salivary drug monitoring have shown changes in the distribution of scopolamine and a significant change in the disposition of the common pain-relief agent acetaminophen taken inflight, in both concentration and time course. The authors of this study emphasize however that their data are preliminary and yet incomplete (Cintron et al, 1987 (a) et (b)). In a previous study we showed changes in lidocaïne disposition during a four-day head-down-tilt (-6°): at the end of the experiment lidocaïne half-life was decreased by 40 % when compared with the ambulatory period (Saivin et al, 1995). In a recent study we showed that promethazine availability after oral absorption was increased by 30% in simulated weightlessness (-6° head-down tilt) compared to sitting position. This statistically significant difference can be explained by the effects of posture on drug absorption (DAR 96/CNES/0361, rapport LPTC 9524-MEDES/96/335).

Drugs are necessary in Space to treat ailments that are common to humans on Earth as well as disturbances caused by the Space environment (pharmacological countermeasures). In this context, the most important changes need to be identified and the use of a drug as a probe will permit to estimate the changes in specific pharmacokinetics parameters. Head-down bedrest models have been used for ground-based studies of the effects of weightlessness on cardiovascular function : this model is thought to well simulate physiologic changes, particularly cardiovascular adaptation syndrome occurring during microgravity (Lathers, 1991 ; Fortney et al, 1991 ; Vernikos et al, 1991 ; Gharib et Hughson, 1992). The cardiovascular function and body fluid regulation are modified during space flights. After hydrostatic pressure gradient withdrawal, weightlessness induces a headward fluid shift of blood volume and extracellular liquids. These modifications are also provoked by anti-orthostatic bed-rest. Due to the small number of space flight physiological experiments, ground simulations and anti-orthostatic bed-rest in particular, are widely used to evaluate weightlessness adaptative processes and to test space mission adapted countermeasure tools in order to help astronauts for their return to Earth.

Physiologic modifications during space flights are described in function of time : an acute phase during the first flight days followed by an adaptative phase, which may require days or weeks (Lathers, 1991). The cardiovascular modifications are among the most important during the first period. Because of the loss of hydrostatic pressure, a fluid shift of extra-and intravascular volumes of the upper part of the body occurs. This increase in blood volume limited to the upper part of the body is considered as a hypervolemia by baroreceptors. In 24 to 48 hours, neurohormonal adaptation induces an actual hypovolemia (Péquignot et al, 1985 ; Butler et al , 1991 ; Vernikos et al, 1992).

We propose to use acetaminophen (1g) as a probe in order to study the influence of simulated weightlessness on oral absorption and more particularly on gastric emptying. This can be performed four times during long duration head down bedrest (HDBR): one time before the experiment for baseline data collection (BDC) in sitting position, after one day of bedrest, after 2 weeks and after three months of bedrest. Acetaminophen has been widely used as a model drug for absorption studies. The pattern of absorption of oral acetaminophen has been shown to be closely associated with the pattern of gastric emptying (Kamali, 1992 ; Renwick, 1992 ; Paintaud, 1998). Moreover, acetaminophen is a commonly used pain relief in general population as well as crewmembers during space flights.

Saliva samples will be collected in order to study the correlation between plasma and saliva concentrations and to assess the usefulness of salivary drug monitoring for space medical operations in the future.

Moreover, in order to complete the assessment of gastric emptying, we will also measure it using an echographic method (Arbeille et al, 1988 ; Ahluwalia et al, 1994).

Only invasive methods are available to accurately study gastric motility. An indirect, non-invasive assessment of oro-caecal transit time can be performed using the breath hydrogen technique which is the most widely used to assess mouth to caecum transit. This test, commonly used in patients, relies on the fact that hydrogen is only produced by bacterial anaerobic fermentation which usually occurs in the colon (Levitt, 1969). Hydrogen, being highly diffusible, rapidly enters the blood stream and is exhaled in the breath within 1-2 min. Oral ingestion of poorly absorbable carbohydrate (lactulose for example) therefore result in a rise in breath hydrogen which occurs within a few minutes of the substrate entering the anaerobic region, usually the caecum (Bond and Levitt, 1975). Moreover, breath hydrogen production correlates very closely with end respiratory breath hydrogen concentration (Metz et al, 1976). End-expiratory gas can be collected and breath hydrogen measured by electrochemical method (Bartlett et al, 1980 ; Thompson et al, 1985 ; Spiller, 1994).

This technique is simple, inexpensive, non-invasive and requires no radiation exposure. Lactulose however, accelerates oro-caecal transit and slows gastric emptying (Miller et al, 1997). In order to avoid an interaction between acetaminophen gastric emptying and lactulose breath test we will perform this test four hours after dosing.

References :

- Ahluwalia NK, Thompson DG (1994) Illustrated Guide of Motility, D.L. Wingate & D. Kumar Eds, Livingstone, Edinburgh.
- Arbeille Ph, Valmalle R, Patat F, Pottier JM, Pleskoff L, Gharib C, Pourcelot L (1988) Follow up of the gastric emptying by ultrasounds after a stress (rotating chair). Interest of this method in space during space motion sickness. The Physiologist, 31 : 86-87.
- Amidon GL, DeBrincat GA, Najib N (1991) Effects of gravity on gastric emptying, intestinal transit, and drug absorption. J. Clin. Pharmacol., 31 : 968-973.
- Bartlett K, Dobson JV, Eastham E (1980) A new method for the detection of hydrogen in breath and its applicaton to acquired and inborn sugar malabsorption. Clin. Chim. Acta 108 : 189-194.
- Bond JH, Levitt MD (1975) Onvestigation of small bowel transit time in man utilizing pulmonary hydrogen (H2) measurements. J. Lab. Clin. Med. 85 : 546-555.
- Burn-Murdoch R, Fischer MA, Hunt JN (1980) Does lying on the right side increase the rate of gastric emptying ? J. Physiol., 302 : 395-398.
- Butler GC, Xing H, Northey DR, Hughson RL (1991) Reduced orthostatic tolerance following 4h head-down tilt. Eur. J. Appl.Physiol., 62 : 26-30.
- Charles JB, Lathers CM (1991) Cardiovascular adaptation to space flight. J. Clin. Pharmacol., 31 : 1010-1023.
- Cintron NM, Putcha L, Chen YM, Vanderploeg JM (1987 (a)) Inflight salivary pharmacokinetics of scopolamine and dextroamphetamine. In : Results of Life Sciences DSOs Conducted Aboard the Space Shuttle 1981-1986 : NASA TM 58280, 153-158.
- Cintron NM, Putcha L, Vanderploeg JM (1987 (b)) Inflight pharmacokinetics of acetaminophen in saliva. In : Results of Life Sciences DSOs Conducted Aboard the Space Shuttle 1981-1986 : NASA 19-23.
- Fortney SM, Hyatt KH, Davis JE, Vogel JM (1991) Changes in body fluid compartments during a 28 days bed-rest. Aviat. Space Environ. Med., 97-104.
- Gharib C, Hughson RL (1992) Fluid and electrolyte regulation in space. In : Bonting SL (eds) Advanced in Space Biology and Medicine. Vol.2. Jai Press Inc. Greenwich, 113-130.
- Houin G (1990) Pharmacocinétique, Ellipses, Paris.
- Kamali F, Edward C, Rawlins MD (1992) The effect of pirenzepine on gastric emptying and salivary flow rate : constraints on the use of saliva paracetamol concentrations for the determination of paracetamol pharmacokinetics. Br. J Clin. Pharmac., 33 : 309-312.
- Lathers CM, Charles JB, Bungo MW (1989) Pharmacology in space. Part 1 Influence of adaptative changes on pharmacokinetics Trends Pharmacol. Sci., 10 : 193-200.
- Levitt MD (1969) Production and excretion of hydrogen gas in man. New Engl. J. Med. 281: 122-127.
- Miller MA, Parkman HP, Urbain JL, Brown KL, Donahue DJ, Knight LC, Maurer AH, Fisher RS. Comparison of scintigraphy and lactulose breath hydrogen test for assessment of orocecal transit. Dig. Dis. and Sci. 42 : 10-18.
- Paintaud G, Thibault P, Queneau PE, Magnette J, Bérard M, Rumbach L, Bechtel PR, Carayon P (1998) Intraindividual variability of paracetamol absorption kinetics after a semi-solid meal in healthy volunteers. Eur. J. Clin. Pharmacol. 53 : 355-359.
- Pavy-Le Traon A, Savin S, Güell A, Soulez-Larivière C, Pujos M, Houin G (1994) The use of medicaments in Space. Therapeutic measures and potential impact of Pharmacokinetics due to weightlessness. ESA Journal; 18:33-50.
- Péquignot JM, Güell A, Gauquelin G, Jarsaillon E, Annat G, Bes A, Peyrin L, Gharib C (1985); Epinephrine, norepinephrine, and dopamine during a 4 days head-down bedrest; J. Appl. Physiol., 58 (1) : 157-163.
- Renwick AG, Ahsan CH, Challenor VF, Daniels R, Macklin BS, Waller DG, George CF (1992) The influence of posture on the pharmacokinetics of orally administered nifedipine. Br. J.clin.Pharmac., 34 : 332-336.
- Rumble RH, Roberts MS, Denton MJ (1991) Effects of posture and sleep on the pharmacokinetics of paracetamol (acetaminophen) and its metabolites. Clin. Pharmacokinet., 20 (2) : 167-173.
- Saivin S, Pavy-Le Traon A, Cornac A, Güell A, Houin G. (1995). Impact of a four-day head-down tilt (-6°) on lidocaine pharmacokinetics used as probe to evaluate hepatic blood flow. J.Clin.Pharmacol. 35:697-704.
- Saivin S, Pavy-Le Traon A, Soulez-Larivière C, Pujos M, Güell A, Houin G (1997) Pharmacology in space : Pharmacokinetics. In : Bonting SL (eds) Advanced in Space Biology and Medicine. Vol.6. Jai Press Inc. Greenwich, CT, 107-121.
- Spiller RC (1994) Illustrated Guide of Motility, D.L. Wingate & D. Kumar Eds, Livingstone, Edinburgh.
- Thompson DG, Binfield P, De Belder A, O’Brien J, Warren S, Wilson M (1985) Extraintestinal influences on exhaled breath hydrogen measurement during the investigation of gastrointestinal disease. Gut. 26: 1349-1352.
- Vernikos J, Dallman MF, Van Loon G, Klein LC, (1991) Drug effects on orthostatic intolerance induced by bed rest. J; Clin. Pharmacol., 31 : 974-984.
- Vernikos J, Keil L, Ertl AC, Wade CE, Greenleaf JE, O’Hara D, Ludwig D (1992) The value of the 4-day head-down bed rest model for screening countermeasures. Aviat. Space Environ. Med., 5 : 449.

#### Protocol justification according to current scientific knowledge

Both physiological and biochemical changes have been reported to occur during space-flight. These changes are important and may alter the pharmacokinetic properties of pharmacological agents administered in space. Some studies have shown that physiological changes due to weightlessness may induce changes in pharmacokinetic behaviour of drugs and influence their dosage regimen. Inflight data obtained by salivary drug monitoring have shown changes in the distribution of scopolamine and a significant change in the disposition of the common pain-relief agent acetaminophen taken inflight, in both concentration and time course. However, these data are preliminary and yet incomplete. In this context, the most important changes need to be identified and the use of a drug as a probe will permit to estimate the changes in specific pharmacokinetics parameters.

#### The interest of the duration of the head-down bed rest has also to be justified,

This experiment schedule could assess the possible changes in pharmacokinetics during future space missions: shuttle flights and International Space Station flights.

#### Rationale for drug and dosage selection

Acetaminophen has been widely used as a model drug for absorption studies. The pattern of absorption of oral acetaminophen has been shown to be closely associated with the pattern of gastric emptying. Moreover, acetaminophen is a commonly used pain relief in general population as well as crewmembers during space flights. Side effects of acetaminophen are rare and usually mild.

### Objectives and hypothesis

#### Main objective of the protocol

The first aim of the present study is to use acetaminophen (1g) as a probe, to assess the influence of simulated weightlessness on oral absorption and more particularly on gastric emptying. This can be performed four times during long duration HDT: one time before the experiment for baseline data collection in sitting position (D-11), the first day of bedrest (D1), after 2 weeks (D18) and after three months (D80) of bedrest. This experiment schedule could assess the possible changes in pharmacokinetics during shuttle flights and ISS flights.

To investigate further the gastric emptying time we will also measure it using an echographic method. Measurement of orocaecal transit time will be performed using the breath hydrogen technique which is the most widely used to assess mouth to caecum transit.

#### Identification of main parameters

Gastric emptying determined by acetaminophen absorption will be expressed by the following parameters : C_max_ (mg.l^-1^): maximum serum acetaminophen concentration, T_max_ (h) : time to reach maximum serum acetaminophen concentration, T_1/2_ determined as the ratio of the logarithm of 2 to the terminal slope k_e_, AUC_0-_ (mg.l^-1^.h): area under the serum acetaminophen concentration-time curve from 0 to infinity.

#### Tested hypothesis

Gastric emptying is known to be strongly influenced by the orientation of the body. Weightlessness could have a strong effect in the gastric emptying process. Exposure to long term bedrest will lead to changes in the disposition of acetaminophen (in both drug concentration and time course).

#### Expected results

Consequently, some changes may occur in the rate of drug absorption which may delay the gastro-intestinal transit time and also transit motility.

#### Secondary objectives

Saliva samples will be collected in order to study the correlation between plasma and saliva concentrations and to assess the usefulness of salivary drug monitoring for space medical operations in the future.

### Specific inclusion and non-inclusion criteria for test subjects

Nineteen male healthy volunteers (10 in the control group and 9 in the pharmacological group) will be included in the present study after they gave their written informed consent. An Ethic’s Committee approval will be obtained prior the start of the study in accordance with legal requirements.

The inclusion criteria will be the followings :

- Male healthy volunteers who will have given written informed consent to participate in the study,
- Age : 25 to 45 years old,
- Recognized as fitted subjects after selection tests.

The exclusion criteria will be the followings :

- Gastrointestinal disease, diarrhoea or constipation and particular diet.
- Allergy to acetaminophen.

### Precise description of the experimental protocol

#### Tests description (methods, duration, time of day, protocol conditions, etc.)

***Overall design :***

- Gastric emptying will be assessed using acetaminophen pharmacokinetics and echographic method on four occasions:
- one time before HDBR for Baseline Data Collection (BDC) in sitting position : D-11,
- at D1, at D18, at D80 during head down bedrest.
- Breath hydrogen test and echographic measurements will be performed the same days in order to assess mouth to caecum transit and gastric emptying.

BDC period HDBR -6°

| Screening | D-11  **PK** |  |  |  | D1  **PK** |  |  | D18  **PK** |  |  |  |  | D80  **PK** |  |
| --- | --- | --- | --- | --- | --- | --- | --- | --- | --- | --- | --- | --- | --- | --- |

Sitting position

*Protocol conditions :*

- After overnight fasting, the subjects will be given 1g acetaminophen with 20 ml of water and a test meal made of liquid yoghurt : 375g 370 ml, 300 cal, 68 % glucides, 18 % lipids, 12 % protides, pH = 3.7 to 4.2, 1000 osm per liter of water.
- During the head-down bed rest, the standing position will be strictly forbidden and the position will be monitored by a video camera.
- Acetominophen and lactulose will be provided by the Pharmacy Department of the Rangueil Hospital. Acetaminophen administered in the present study will have the same batch.
- If necessary, aspirin administration will be allowed.
- Contra-indicated concomitant medications : treatment with acetaminophen and any drug which has an effect or a putative effect on drug absorption or gatrointestinal motility (antiacids, gastric secretory inhibitors, gastrointestinal cytoprotective agents or peristaltic stimulants...).

*Schedule of the experiment :*

- Sitting position: D-11
- Basal echographic measurement,
- Blood and salivary sampling before the acetaminophen administration (T-10 min),
- Acetaminophen and test meal administration (T0),
- Echographic measurement at T30 min (maximal distension of the antrum), and each 30 min after this second measure until the antrum volume recovered completely,
- Blood sampling at : T15 min, T30 min, T40 min, T50 min, T60 min, T75 min, T90 min, T120 min, T240 min, T6 h, T8 h, T10 h,
- Urinary sampling at T+0-3h, T+3-6h, T+6-9h, T+9-12h,
- Salivary sampling : T30 min, T60 min, T90 min, T120 min, T240 min, T6 h, T8 h, T10 h,

After T240 min blood sampling, the lactulose breath test will begin :

- basal breath hydrogen values measurement each 5 min from T’-30 min to T’0,
- lactulose administration (T’0),
- Breath sampling each 5 min from T’0 till the hydrogen concentration rises by 10 ppm on three successive measures,

- In order to assess plasma volume, hematocrit and hemoglobin measurements will be performed on baseline blood samples (T-15 min) and after 1 hour (T 60 min), after 4 hours (T 240 min) and at the 10th hour.

- D1, D18, D80 head-down bedrest: D-11 same experimental protocol will be performed.

In all, **130 ml** of blood will be sampled.

*Methods :*

- The ultrasound method

The cross sectional area of the gastric antrum will be measured at two levels: S1 on the sagittal plane passing through the superior mesenteric vein, S2 on the sagittal plane crossing the limit between the antrum and the lesser curvature of the stomach. For the evaluation of the antrum volume, changes during the test we used the expression V - { (S1 + S2)/2 }. The echographic evaluation is a safety, accurate and reproducible method for the assessment of the gastric function.

Splanchnic venous flow will also be measured (portal vein diameter & velocity : one measure at each antrum measurement).

- Breath hydrogen test

This test, commonly used in patients, relies on the fact that hydrogen is only produced by bacterial anaerobic fermentation which usually occurs in the colon. Hydrogen, being highly diffusible, rapidly enters the blood stream and is exhaled in the breath within 1-2 min. Oral ingestion of poorly absorbable carbohydrate (lactulose for example) therefore result in a rise in breath hydrogen which occurs within a few minutes of the substrate entering the anaerobic region, usually the caecum. Moreover, breath hydrogen production correlates very closely with end respiratory breath hydrogen concentration. End-expiratory gas can be collected and breath hydrogen measured by electrochemical method. The subjects breathe out through a washable plastic mouthpiece into 1.7 m wide bore (1.2 cm diameter) tube, sampling gas from the proximal part of the tube using a three-way tap. After 4-5 steady breaths through the mouthpiece with their nasal passage gently occluded by pinching between finger and thumb, subjects should be asked to breath out and then hold their breath while 20 ml of gas is aspirated from the three-way tap (cf. 6.7.4.7.2 hydrogen measurements).

#### Measured parameters

- Plasma and salivary of acetaminophen concentrations will be measured using a enzym immunoassay method (réactif Dade-Berhing) on automate Cobas-Mira.

- The cross sectional area of the gastric antrum will be measured at two levels: S1 on the sagittal plane passing through the superior mesenteric vein, S2 on the sagittal plane crossing the limit between the antrum and the lesser curvature of the stomach.

Splanchnic venous flow will also be measured (portal vein diameter & velocity : one measure at each antrum measurement).

- End-expiratory gas will be collected and breath hydrogen measured by electrochemical method.

#### Equipment used

All the experimental equipment is commercially available :

* Blood samples : they will be collected through a canule inserted into a forearm vein. Blood will be sampled (2.5 ml) in Vacutainers ® tubes (Lithium heparin).

* Salivary samples : they will be collected using dental cotton wool cylinders (Starstedt).

* The ultrasound examinations will be always performed by the same specially trained investigator, with a high resolution real time ultrasound machine (ESAOTE challenge 2000) with a 3.5 Mhz probe

* Breath hydrogen concentrations will be measured by electrochemical method : electrochemical hydrogen meter (Biotech Company).

#### Description of the test termination criteria of the protocol

- Blood and salivary sampling before the acetaminophen administration (T-10 min) till 10h later.

- Breath sampling each 5 min from T’0 till the hydrogen concentration rises by 10 ppm on three successive measures,

- Echographic measurement at T30 min (maximal distension of the antrum), and each 30 min after this second measure until the antrum volume recovered completely,

#### Specific medical monitoring (ECG, blood pressure monitoring, etc.)

During the pharmacokinetic study, blood pressure and heart rate will be monitored each hour during the first 4 hours and then every 2 hours until the 10th hour.

#### Other specific requirements (dietary, conditions of the experiment sessions, etc.)

- After overnight fasting, the subjects will be given 1g acetaminophen with 20 ml of water and a test meal made of liquid yoghurt : 375g 370 ml, 300 cal, 68 % glucides, 18 % lipids, 12 % protides, pH= 3.7 to 4.2, 1000 osm per liter of water. Four hours after acetaminophen dosing (T240) they will be given lactulose.

- The other overall conditions will be the followings :

- meals will be standardised and food intake will be around 2000 Kcal.

- liquid intake at 2,5 l/day, sodium intake around 6g/day.

- intake of beverages including tea, coffee or cola and grapefruit juice will be not allowed.

- a diet low in poorly absorbable carbohydrate the night before the experiment.

- the subjects should brush their teeth thoroughly and rinse their mouth with chlorhexidine mouthwash every morning before the experiment to minimize the oral cavity bacterial flora.

- before the start of the study the volunteers will have to follow dietary requirements given at screenning by a dietician.

- On pharmacokinetics days, standard meal will be taken after the end of the lactulose breath test.

- No other specific conditions are requested for the present experiment

#### Biological samples handling, preservation conditions, transport

##### Measured parameters

* Blood samples : they will be collected through a canula inserted into a forearm vein. Blood will be sampled (2.5 ml) in Vacutainers® tubes (Lithium heparin) : they will be immediately centrifuged and plasma will be frozen at -20°C until assayed.

* Salivary samples : they will be collected using dental cotton wool cylinders (Starstedt). Each salivary sample will start 5 min. before each blood sample. They will be immediately centrifuged . (4°C during 20 min ; 4500 rpm) and frozen at - 20°C until assayed.

* Urinary samples : The volume of each micturition will be noted and a sample (15 ml) will be immediately frozen at -20°C until assayed.

##### Methods, equipment and place of analysis

Plasma and salivary of acetaminophen concentrations will be measured in the laboratory of Pharmacokinetics in Toulouse Hospital.

They will be measured using a enzym immunoassay method (réactif Dade-Berhing) sur automate Cobas. This method will be validated for salivary samples ; manufacturer standard method for blood samples

The hydrogen is measured by the electrochemical method. This consists of a hydrogen-sensitive polarographic cell. The gas sample (10-20 ml) is injected into the measurement chamber from which hydrogen diffuses through a metalized membrane at a rate proportional to its concentration. Hydrogen is oxidized at the membrane thus generating an electromotive force which can be detected and displayed on a digital readout scale as a concentration measured in parts per million. The equipment will be lent by the Gastroenterology Department (Rangueil Hospital).

##### Labelling, storage and packaging of the samples

Samples will be immediately centrifuged and frozen at -20°C until assayed.

#### Data utilisation and analysis, specify

##### Parameters analysed

Cf those appearing in the 6.7.4.8.2

##### Statistical tests

Pharmacokinetics calculations will be done using the Kinetica program (Innaphase, Champs sur Marne). Data analysis will be performed in the Laboratory of Pharmacokinetics in Toulouse Rangueil Hospital. An ANOVA analysis will be performed on the following parameters: C_max_, T_max_, AUC _0-t_, AUC _0- ∞_, T_1/2_,

- C_max_ (mg.l^-1^): maximum serum acetaminophen concentration observed,
- T_max_ (h): time to reach maximum serum acetaminophen concentration,
- AUC_0-_ (mg. l^-1^.h): area under the time concentration curve calculated by the trapezoidal method and extrapolated to infinity by the ratio of the last concentration to the slope of the terminal phase.
- T_1/2_: terminal half-life calculated by the ratio of ln2 to the slope of the terminal phase.

If possible salivary concentrations will be correlated with plasma concentrations.

Software used

Pharmacokinetics calculations will be done using the Kinetica program (Innaphase, Champs sur Marne).

#### Hazards, side effects, possible inconveniences, discomfort and constraints to subjects

Except the possible hazards, inconveniences or discomforts to the subjects during head-down bedrest, hazards due to this specific experiment are the following:

- Side-effects of acetaminophen are rare and usually mild though haematological reactions have been reported. Skin rashes and other allergic reactions occur occasionally, thrombocytopenia are exceptional.
- Blood sample : bleeding, haematoma formation and infection can occur but this hazards have a low probability and can be prevented by careful handling, compression after withdrawal of needle, alcohol swap and careful cleaning. A total of 130 ml of blood will be drawn during the study.
- Urine sample: no hazard.
- Salivary sample: no hazard
- The echographic evaluation of the gastric emptying is a safety method, breath hydrogen test as well.

### Protocol summary

Objective of the study:

The first aim of the present study is to assess the influence of simulated weightlessness on acetaminophen oral dosing. Head-down bedrest models have been used for ground-based studies of the effects of weightlessness: this model is thought to well simulate physiologic changes occurring during Space flights.

Drugs are necessary in Space to treat ailments that are common to humans on Earth as well as disturbances caused by the Space environment. The pharmacokinetics of drugs is determined by the combination of several complex phenomena. Each step of this process may be influenced by physiological changes occurring in Space flight. Gastric emptying is particularly one of the most important step for many orally administered drugs. This step allows drugs to get in the intestine which is the main site of drug absorption. Absorption corresponds to the appearance of the drug and/or its metabolites in the circulation from the site of its administration. Thus, only the amount reaching the circulation will be able to produce pharmacological effects. Consequently, some changes in gastric-emptying may delay the gastro-intestinal transit and may alter the oral bioavailability of drugs. On earth, gastric emptying is known to be strongly influenced by the orientation of the body. During Space flights, the absence of gravity could have a strong effect since in the gastric emptying process the likelihood that drugs or food in the stomach passe through the pylorus is a random-chance phenomenon. Some studies have shown that physiological changes due to weightlessness may induce changes in pharmacokinetic behaviour of drugs and influence their dosage regimen. However, no study has investigated the effects of weightlessness on gastric emptying.

We propose to use acetaminophen (1g) as a probe in order to study the influence of simulated weightlessness on oral absorption. Acetaminophen has been widely used as a model drug for absorption studies. The pattern of absorption of oral acetaminophen has been shown to be closely associated with the pattern of gastric emptying. Moreover, acetaminophen is a commonly used pain relief in general population as well as crewmembers during space flights.

Schedule of the study:

Gastric emptying will be investigated four times during long duration Head Down Bed Rest (HDBR):

- before the experiment (BDC-11) in sitting position,
- and after 1 day (HDT1), 18 days (HDT18) and 80 days (HDT80) in head down position.

Two methods will be used to investigate gastric emptying :

- Acetaminophen pharmacokinetics will be estimated by blood, saliva and urines samples,
- In order to precise influence of weightlessness on absorption this study will be completed by gastric emptying evaluation using echographic measurements.

A measurement of the oro-caecal transit time will be performed the same days using the breath hydrogen test.

Methods:

Blood samples : they will be collected through a canule inserted into a forearm vein.

The ultrasound method : doppler echographic techniques are non-invasive and based on the ultrasound capability to penetrate the various parts of the body to detect and display the organs, and to measure the velocity of blood within the heart and vessels. They use a probe or a small sensor fixed to the skin comparable to those used to visualize the fetus of a pregnant woman.

Breath hydrogen test is the most widely used to assess mouth to caecum transit. This test is commonly used in patients and relies on the fact that hydrogen is only produced by bacterial anaerobic fermentation which usually occurs in the colon. Hydrogen, being highly diffusible, rapidly enters the blood stream and is exhaled in the breath within 1-2 min. Oral ingestion of poorly absorbable carbohydrate (lactulose for example) therefore result in a rise in breath hydrogen which occurs within a few minutes of the substrate entering the anaerobic region, usually the caecum. Moreover, breath hydrogen production correlates very closely with end respiratory breath hydrogen concentration. End-expiratory gas can be collected and breath hydrogen measured by electrochemical method. You will just have to breathe out through a washable plastic mouthpiece into a tube, sampling gas from the proximal part of the tube using a three-way tap. After 4-5 steady breaths through the mouthpiece with their nasal passage gently occluded by pinching between finger and thumb, subjects should be asked to breath out and then hold their breath while 20 ml of gas is aspirated from the three-way tap.

Salivary samples : they will be collected using dental cotton wool cylinders.

Hazards, side effects, possible inconveniences, discomfort and constraints to subjects :

Except the possible hazards, inconveniences or discomforts to the subjects during head-down bedrest, hazards due to this specific experiment are the following:

- Side-effects of acetaminophen are rare and usually mild though haematological reactions have been reported. Skin rashes and other allergic reactions occur occasionally, thrombocytopenia are exceptional.
- Blood sample : bleeding, haematoma formation and infection can occur but this hazards have a low probability and can be prevented by careful handling, compression after withdrawal of needle, alcohol swap and careful cleaning. A total of 130 ml of blood will be drawn during the study (32.5 ml in each session).
- The echographic evaluation of the gastric emptying is a safety method, breath hydrogen test as well.
- The hydrogen breath test is a non-invasive test. Flatulence or diarrhea may occur after lactulose administration, but these adverse effects have been described in patients taking large doses of lactulose.
- Urine sample: no hazard.
- - Salivary sample: no hazard

### Investigators

**Principal Investigators:** Dr. M.P. Bareille

**Co-Investigators**:

Pr. Arbeille P.

Dr. Delvaux M.

Mlle Gandia P.

Pr. Houin G.

Dr. LAVIT M.

Dr. Pavy-Le Traon A.

Dr. Saivin S.

## Validation of measures against backpain during bed rest

### Scientific Background

#### Problem presentation

Astronauts and subjects of bed rest studies frequently experience back pain. During the last years we applied, both during bed rest and spaceflight, a new method allowing a continuous, ambulant monitoring of spine movements and curvatures (Baum et al. 1997, Baum and Essfeld 1999). The results of these studies showed that bed rest- and microgravity-induced back pain is associated with reduced amplitudes of spine movements,

1. the location and quality of pain are largely uniform: A dull pain occures in the lumbar region
2. about 10 cm lateral of the spinuos processes,
3. length changes do not correlate with pain intensity,
4. and increasing these amplitudes through exercise transiently reduces the severity of back pain.

These results suggest that the reduced amplitudes are the origin, not the consequence, of back pain. Therefore, we proposed regular, slow, large-amplitude movements as a countermeasure (Baum and Essfeld 1999). In more detail, a countermeasure might consist of extensions and flexions in the sagittal plane as well as maximal spine movements in the frontal plane over periods of about 4 minutes every two hours during daylight. To validate the effect of these countermeasures,

1. subjects will be devided into a control- and treatment group,
2. intensity, quality, and location will be assessed by means of a questionnaire, and
3. spine geometry and back muscle activity will be quantified continuously over 24 h periods.

#### Literature data including references

- Baum K., Hoy S., Essfeld D. (1997) Continuous monitoring of spine geometry: A new approach to study back pain in space. Int. J. Sports Med. 18, 331 - 333
- Baum K., Essfeld D. (1999) Origin of back pain during bed rest: A new hypothesis. Eur. J. Med. Res. 4, 389 – 393

#### Protocol justification according to current scientific knowledge

Although the frequent occurrence of back pain during bed rest and space flight is a well known phenomenon, the origin of pain and, therefore, effective countermeasures are still missing. With the present protocol, data will allow to give both a better understanding of the cause of back pain and the effectiveness of physical countermeasures.

#### The interest of the duration of the head-down bed rest

The present literature with respect to the occurrence of back pain exclusively focuses on short term effects lasting one to two weeks. During this phase, a continuous decline of pain sensations has been described. However, there exists no information whether the relief of pain is a long lasting effect or transiently in nature.

#### Rationale for drug (if used) and dosage selection

No drugs will be administered

### Objectives and hypothesis

#### Main objective of the protocol

The main objective of the protocol is to evaluate the origin of back pain and the validation of measures (regular, slow, large-amplitude movements of the spine) against backpain during bed rest.

#### Identification of main parameters

- 24 h profile of spine geometry
- flexibility index of spine
- activity of lower back muscles
- subjective rating of back pain

#### Tested hypothesis

Without countermeasure, back pain will occur in most subjects.

Back pain will be associated with reduced movement amplitudes and increased EMG-activities of the lower back.

Regular, slow, large-amplitude movements of the spine will significantly reduce, if not abolish, lower back pain.

#### Expected results

see 6.8.2.3

### Specific inclusion and non-inclusion criteria for test subjects

Subjects should be from chronic back pain

### Precise description of the experimental protocol

#### Tests description (methods, duration, time of day, protocol conditions, etc.)

- 24h-recording of geometry of the thoracic and lumbar spine and of the activity of the lower back
- muscles (iEMG). Time schedule: BDC-13, BDC-8, BDC-3, HDT 1-2-3-5-8-14-28-48-68-88, R+1, R+3, R+13
- evaluation of spine flexibility by standard positions (pre and post bed rest: BDC-13, BDC-8, BDC-3 and R+1, R+3, R+13)
- In the treatment group, the following countermeasure will be applied:
- The subjects will perform slow, large-amplitude movements in the sagittal and frontal plan as well as around the longitudinal axis
- The final position of each movement will be kept for 5 s. The treatments will be applied during daylight hours only. The overall duration of one treatment will be 4 minutes. The interval between treatments should be between two to three hours.

#### Measured parameters

- daily questionnaire concerning back pain (scale from 0 = no pain to 5 = extreme)
- continuous monitoring of geometry of the thoracic and lumbar spine by miniaturised ultrasound transmitters and receivers
- - continuous recording of the activity of the lower back muscles by a miniaturised EMG recording unit (iEMG)
- evaluation of spine flexibility by standard positions (pre and post bed rest)

#### Equipment used

### 1. miniaturized ultrasound transmitters and receivers (manufacturer: Dr. Manthey Orthoson, Weimar, Germany)

2. miniaturized EMG recording unit (manufacturer: biovision, Wehrheim, Germany)

#### Description of the test termination criteria of the protocol

Drug intervention against pain will be allowed if pain sensations equals or exceeds the range of 4 (= severe)

#### Specific medical monitoring (ECG, blood pressure monitoring, etc.)(if necessary)

Not needed

#### Other specific requirements (dietary, conditions of the experiment sessions, etc.)

Not needed

#### Biological samples handling, preservation conditions, transport

No biological samples will be drawn

#### Data utilisation and analysis

The location, quality and intensity of back pain will be evaluated by a questionnaire used in our previous studies. During the first ten days of bed rest, the questionnaire should be filled out daily. Thereafter, a modified questionnaire will be used once a week.

The geometry of the thoracic and lumbar spine will be monitored by a method described previously [2]. Briefly: the skin distance between miniaturised ultrasound transmitters and receivers (diameter 2 cm, height 0,6 cm) is measured with an accuracy of 1 mm on the basis of the constant ultrasound propagation velocity in soft tissues (1500 m/s in the range of physiological temperatures). The sensors are positioned in such a way that the distances between C7 and Th12/L1 as well as between TH12/L1 and L5/S1 can be determined on both sides of the spinous processes. Identical changes of the right and left distances indicate pure flexion or extension in the sagittal plane, changes in opposite direction correspond to movements in the frontal plane. The sensors are connected via thin cables with a central unit (Orthoson©) which records the data at a frequency of 1Hz for 24 hours. The central unit has the dimensions of 12.5 cm * 6 cm * 1 cm and a weight of 180g. During the experiments it will be worn in a breast pocket of a T-shirt. After the experiments, the data will be transferred to a PC and analysed as time series and in terms of amplitude histograms. The range of movement throughout the day is quantified as the central range of amplitudes covering 90 % of all values measured. Additionally, the maximal amplitude of movement of the thoracic and lumbar segment will be measured in standard positions before, immediately after, and three days after the bed rest. For that purpose the subjects have to perform a maximal flexion and extension of the spinal column while resting on all fours with the knee joint flexed to 90^0^.

As a measure of segmental flexibility in the sagittal plane, the following index of flexibility will be used:

(F – E) * 100

Index of flexibility :

F

*F = distance during maximal flexion in the standard position*

E = distance during maximal extension in the standard position

The activity of lower back muscles will be assessed by a miniaturized, commercially available EMG recording unit (Fa. Ernst GbR). The method provides a continuous recording of the integrated EMG signal over periods of 24 h.

#### Hazards, side effects, possible inconveniences, discomfort and constraints to subjects

Both devices neither disturb the spontaneous movement behaviour of subjects nor their sleep comfort. They will be applied in parallel for 24 h before bed rest, during bed rest at days 1, 2, 4, 7, in the middle and at the end of the bed rest period as well as during recovery.

### Protocol summary (one page), written on a separate sheet

Astronauts and subjects of bed rest studies frequently experience back pain. During the last years we applied, both during bed rest and space flight, a new method allowing a continuous, ambulant monitoring of spine movements and curvatures. The results of these studies showed that bed rest- and microgravity-induced back pain is associated with reduced amplitudes of spine movements, the location and quality of pain are largely uniform: A dull pain occurs in the lumbar region about 10 cm lateral of the spinuos processes, length changes do not correlate with pain intensity, and increasing these amplitudes through exercise transiently reduces the severity of back pain.

These results suggest that the reduced amplitudes are the origin, not the consequence, of back pain. Therefore, we proposed regular, slow, large-amplitude movements as a countermeasure. In more detail, a countermeasure might consist of extensions and flexions in the sagittal plane as well as maximal spine movements in the frontal plane over periods of about 4 minutes every two hours during daylight. To validate the effect of these countermeasures, subjects will be divided into a control- and treatment group, intensity, quality, and location will be assessed by means of a questionnaire, and spine geometry and back muscle activity will be quantified continuously over 24h periods.

The devices used in the present study neither disturb the spontaneous movement behaviour of subjects nor their sleep comfort. They will be applied in parallel for 24 h before bed rest (BDC-13, -8, -3), during bed rest at days (HDT1, 3, 2, 5, 8, 14, 28, 48, 68, 88) as well as during recovery (R+1, +3, 13).

### Investigators

**Principal Investigators:** Pr. K. Baum

**Co-Investigators**:

Pr. Essfeld D.

Mr. Langenohl M.

Dr. Ruether T.

## Molecular plasticity of human skeletal muscle compartments after bed rest.

### Scientific Background

#### Problem presentation

Human skeletal muscle atrophy is a well-known phenomenon in relevant muscle diseases (e.g., type-II fiber atrophy or amyotrophic lateral sclerosis) and immobility (”disuse atrophy”). Substantial atrophy is, therefore, a severe problem in immobili-sation due to prolonged clinical bed-rest and/or microgravity exposure in space flight. Either disuse and/or unloading of skeletal muscle results in atrophy particularly of limb extensor muscle groups (i.e., postural or ”anti-gravity” extensor muscles) within days and weeks following immobilization and/or unloading with dramatic structural and functional changes which may impair, if not prevent, cell and tissue repair mechanisms during muscle remobilization and recovery.

Although the consequences of muscle atrophy, a loss in force and power, and the major histopathological features, e.g., the decreased total fiber numbers, the switch in fiber-types (e.g. -I or -II) or groups, and the altered ultrastructural integrity of the contractile apparatus, are clear, the mechanisms of atrophy are barely understood. Fundamental insights into the cellular and molecular events of atrophying muscle may thus help to improve countermeasure design for impaired muscle functions during bed-rest or human space flights and remobilization/recovery thereafter.

Our main scientific approach aims at documenting compartmental expression patterns of two candidate molecules important for normal muscle structure and function, (i) nitric oxide synthase (NOS), which generates the gaseous signal NO, and (ii) matrix metalloproteinases (MMPs, or ”matrixins”), intimately involved in extracellular matrix remodeling and plasticity. NOS and MMP localization and expression will be investigated in defined cellular compartments of muscle fibers and endo-/perimysial cells surrounding individual fiber groups in normal and atrophic human limb muscle samples biopsied from volunteers subjected to prolonged bed-rest with and without excercise countermeasures. Amongst others, these two candidate molecules play major roles in normal human muscle structure, function and (patho)physiology. Both NOS and MMP have been used in immunocytochemical studies, and enzyme activities. The amount of protein and mRNA transcripts have been previously investigated in human muscle biopsies.

In addition, altered numbers of motor end plates will be quantitated in muscle sections from the biopsies obtained in order to document neuromuscular plasticity mechanisms that may parallel, if not be linked with NOS and MMP expression in atrophic muscle and its putative impact on exercise countermeasures.

#### Literature data including references

The following list consists of selected references listed according to key words relevant to this study and first author et. al., if more than two. References are listed consecutively by arabical numbers and publication year. The main purpose of each paper is given in brackets. Review papers are indicated.

Muscle biology, physiology and pathophysiology:

- Engel AG, Franzini-Armstrong C, eds. (1994) Myology, 2nd ed. McGraw-Hill, New York, Vol. 1 and 2. (General review and monography)

Muscle atrophy studied by human bed-rest studies (”simulated microgravity”):

- - Ferrando et al., (1996) Prolonged bed rest decreases skeletal muscle and whole body protein synthesis. Am J Physiol 270:E627-33. (Original paper)
  - Bloomfield SA (1997) Changes in musculoskeletal structure and function with prolonged bed-rest. Med Sci Sports Excercise 29:197-206. (Original paper)
  - LeBlanc, A, Rowe R, Evans H, West S, Shackleford L, Schneider V (1997) Muscle atrophy during long duration bed rest. Int J Sports Med 18:S283-S285. (Review)
  - Riley DA et al., (1998) Disproportionate loss of thin filaments in human soleus muscle after 17-day bed rest. Muscle&Nerve 21:1280-1289. (Filament loss in soleus muscle after bed-rest)
  - Koryak Y (1998) Effect of 120 days of bed-rest with and without counter-measures on the mechanical properties of the triceps surae muscle in young women. Eur J Appl Physiol 78: 128-135 (prolonged bed rest study in women)

Microgravity and skeletal muscle:

- Desplanches D (1997) Structural and functional adaptations of skeletal muscle to weightlessness. Int J Sports Med 18:S259-S264. (Review)
- Fitts RH, Riley D, Widrick JJ (2000) Microgravity and skeletal muscle. J Appl Physiol 89:823-839. (Latest review)

Muscle Plasticity and Countermeasures

- Booth FW and Thomason DB (1991) Molecular and cellular adaptations of muscle in response to excercise: perspectives of various controls. Physiol Rev 71:541-585. (Review)
- Booth FW, Criswell DS (1997) Molecular events underlying skeletal muscle atrophy and the development of effective countermeasures. Int J Sports Med 18: S265-S269. (Countermeasure protocols according to molecular aspects)
- Landauer JA, Burke MS (1998) A proposed cause for and prevention of bone and muscle wasting in microgravity. Aviat Space Environ Med 69:699-702. (Involvement of NO in muscle atrophy is discussed)
- 11a) Kingwell BA (2000) Nitric oxide as metabolic regulator during excercise: effects of training in health and disease. Clin Exp Pharmacol Physiol 27:239-250.

The NOS/NO-System in Muscle:

NO/NOS Reviews:

- Förstermann U et al. (1995) Isoforms of nitric oxide synthase: Properties, cellular distribution and expressional control. Biochem Pharmacol 50: 1321-1332. (Review)
- Wang Y et al., (1999) Neuronal NOS: Gene structure, mRNA diversity, and functional relevance. Crit Rev Neurobiol 13:21-43. (Molecular biology of NOS-I)
- Boucher JL et al., (2000) Nitric oxide biosynthesis, nitric oxide synthase inhibitors, and arginase competition for L-arginine utilization. Cell Mol Life Sci 55:1015-28. (Latest review)

Function and regulation of the muscular NOS/NO-System:

- Bode-Boger SM et al. (1994) Excercise increases systemic nitric oxide production in men. J Cardiovascular Risk 1: 173-178. (First human study of NO production under training conditions)
- Reiser PJ et al. (1997) Induction of neuronal type nitric oxide synthase in skeletal muscle by chronic electrical stimulation in vivo. J Appl Physiol 82:1250-5. (NOS induced by electric stimulation)
- Fujii Y et al. (1998) Regulation of nitric oxide production in response to skeletal muscle activation. J Appl Physiol 85:2330-6. (Excercise regulates the NOS/NO-system)
- Maddali S et al., (1998) Postexercise increase in nitric oxide synthase in football players with muscle cramps. Am J Sports Med 26:820-4. (Functional link between NO and muscle contractility)
- Tidball JG et al. (1998) Mechanical loading regulates NOS expression and activity in developing and adult skeletal muscle. Am J Physiol 275: C260-C266. (NOS as force-generating protein)
- Reid MB (1998) Role of nitric oxide in skeletal muscle: synthesis, distribution and functional importance. Acta Physiol Scand 162: 401-409. (Review, NO as key mediator of metabolism)
- Marechal G, Gailly P (1999) Effects of nitric oxide on the contraction of skeletal muscle. Cell Mol Life Sci 55:1088-1102. (Review)
- Murrant CL et al. (1999) Exogenous reactive oxygen and nitric oxide alter intracellular oxidant status of skeletal muscle fibers. Acta Physiol Scand 166:111-121. (Review, NO as intrafiber mediator of metabolism)

Expression of NOS isoforms (NOS-I-III) in human muscle (including basal levels of NO/NOS in normal human subjects):

- - - Nakane et al., (1993) Cloned human brain nitric oxide synthase is highly expressed in skeletal muscle. FEBS Lett 316:175-180. (First study on NOS expression in human muscle)
    - Kobzik et al., (1994) Nitric oxide in skeletal muscle. Nature 372:546-48 (NOS-I in human muscle localized to sarcolemma)
    - Kobzik et al., (1995) Endothelial type nitric oxide synthase in skeletal muscle fibers: mitochondrial relationships. Biochem Biophys Res Commun 211:375-81. (NOS-III in muscle)
    - Frandsen U et al., (1995) Localization of nitric oxide synthase in human skeletal muscle. Biochem Biophys Res Commun 227:88-93. (Follow-up study on NOS in muscle)
    - Park et al. (1996) Constitutive expression and structural diversity of inducible isoform of nitric oxide synthase in human tissues. Life Sci 59:219-25. (NOS-II in human muscle)
    - Grozdanovic Z et al. (1997) Absence of nitric oxide synthase I despite the presence of the dystrophin complex in human striated muscle. Histochem J 29:97-104. (NADPH-diaphorase histochemistry, a valuable and semiselective marker for NOS-activity)
    - Lin CS et al. (1998) Analysis of neuronal nitric oxide synthase isoform: Expression and identification of human nNOS. Biochem Biophys Res Commun 253:388-394. (muscle-specific NOSµ in muscle tissue)

Methods for NOS/NO detection in muscle tissue (rodent and human):

- Nakos G and Gossrau R (1994) When NADPH-diaphorase (NADPHd) works in the presence of formaldehyde, the enzyme appears to visualize selectively cells with constitutive NOS. Acta histochem 96:335-43. (catalytic NOS-activity via NADPHd-histochemical reaction is stabilized by fixation techniques)
- Springall DR et al. (1992) Immunological detection of nitric oxide synthase(s) in human tissues using heterologous antibodies suggesting different isoforms. Histochemistry 98:259-66. (Various NOS isoforms are immunolocalized in human tissues
- Lück G et al., (1998) In situ identification of neuronal nitric oxide synthase mRNA in mouse and rat skeletal muscle. Neurosci Lett 246:77-80. (First In Situ hybridization study of NOS-I in skeletal muscle)

NO/NOS in human muscle dystrophy:

- Brenman et al. (1995) Nitric oxide synthase complexed with dystrophin and absent from skeletal muscle sarcolemma in Duchenne muscular dystrophy. Cell 82: 743-52. (NOS is linked to sarcolemma via the DGC-complex and absent from DGC-deficient Duchenne patients)
- Chao et al., (1996) Selective loss of sarcolemmal nitric oxide synthase in Becker Muscular Dystrophy. J Exp Med 184:609-618.
- Yang C C et al., (1996) Increase of nitric oxide synthase and nitrotyrosine in inclusion-body myositis. NeuroReport 8:153-58.
- Schoser BGH et al. (1997) Partial loss of nitric oxide synthase associated with NADPH-dependent diaphorase/nitric oxide synthase-complex in amyotrophic lateral sclerosis and human type-II myofiber atrophy. Neurosci Lett 231:163-166.
- Ohkoshi N, Mizusawa H, Fujita T, Shoji S (1997) Histological determination of nitric oxide synthase (NOS) and NADPH-diaphorase in ragged-red fibers from patients with mitochondrial encephalomyopathies. Neurol Sci 149:151-56.
- Tews DS et al (1997) Expression of different isoforms of nitric oxide synthase in experimentally denervated and reinnervated skeletal muscle. J Neuropathol Exp Neurol 56: 1283-89.

Matrix Metalloproteinases (MMP) in muscle research:

Review:

- Nagase H (1996) Matrix metalloproteinases: In: Hooper NM (ed.) Zinc Metalloproteinases in Health and Disease, Taylor & Francis Ltd., London, pp.153-204.

MMPs in human dystrophic skeletal muscle:

- Springman EB et al. (1989) Multiple roles of activation of latent human fibroblast collagenase: evidence for the role of Cys73 active-site zinc complex in latency and a ”cystein switch” mechanism for activation. PNAS 87:364-8.
- Kheif S et al., (1998) Matrix metalloproteinases MMP-2 and MMP-9 in denervated muscle and injured nerve. Neuropathol Appl Neurobiol 24:309-319
- Schoser BGH, Blottner D (1999) Matrix metalloproteinases MMP-2, MMP-7, and MMP-9 in denervated human muscle. NeuroReport 10:1-3. (MMPs are upregulated around atrophic fibers of ALS and SMA patients)
- Schoser BGH, Blottner D, Stuerenberg, Expression of matrix metalloproteinases MMP-2, MMP-7 and MMP-9 in human inflammatory myopathies. ms. submitted. (MMPs are up-regulated in human myositis)

#### Protocol justification according to current scientific knowledge

The research project proposed on human muscle biopsies from a controlled long-term bed rest campaign with volunteers addresses major questions that are relevant to muscle atrophy following clinical bed-rest (immobilization) or space flights (microgravity) including countermeasure trials by e.g., exercise intervals. Though data obtained from animal research are valuable and necessary they cannot fully account for the mechanisms in human muscle atrophy and remobilization/ regeneration. Therefore, the investigations performed on human subjects may considerably add to the scientific knowledge on human muscle atrophy in health and disease and applied physiology.

The proposal has a clear scientific objective, it will be performed with well-accepted methods, and it offers a plausible data analysis plan. The methods are valid, practically feasible, and have the power to test the hypothesis put forward by the proposal. The biopsy protocol considers parameters comparable to previous bed-rest studies and to those that will be measured in later follow-up studies aimed at alternative countermeasure trials.

#### Duration of the head-down bed rest campaign

Histopathological changes in the expression pattern of NOS and MMPs in muscle are expected at individual fiber groups starting in the first weeks of bed-rest (subtle changes at individual fibers or groups) and are expected to be manifested within prolonged bed rest at numerous fiber groups and surrounding endomysial/perimysial cell compartments. Prolonged bed-rest allows for clear documentation of relevant markers in affected structures (i.e., maximal response) of atrophying muscle tissue. Controlled long-term bed-rest thus will be necessary to document (i) manifestated structural changes in NOS, MMP and (ii) altered amounts of motor endplate structures of affected fibers during ongoing atrophy processes. The data collection obtained by this prolonged bed-rest study will be used in long-term follow-up investigations on countermeasure attempts by e.g. exercise, to ameliorate fiber atrophy in human muscle. Preliminary data already exist from a countermeasure study in rodent skeletal muscle treated with NOS-inhibitors to ameliorate motor dysfunctions to overcome muscle impairments in health and disease (Ikeda et al., 1998). No such data are available for e.g. exercise countermeasure in human skeletal muscle.

#### Rationale for drug and dosage selection:

During biopsy protocol (punched needle biopsy of 3-5mm), anti-pain treatment (analgesia) will be done with a local anaesthetics (e.g. *Lidocain*), and, if nescessary, postbiopsy anti-pain treatment may be done with an oral drug (e.g. *Tramadol*). Dosages and durations are to be determined.

### Objectives and hypothesis

#### The objectives of the proposal are

- to investigate compartment-related changes from manifested phases of disuse atrophy in human skeletal muscle,
- mapping of specific markers (NOS, MMP, AChR) from major cellular and molecular compartments including the motor end plate susceptible to fiber atrophy and plasticity,
- to provide a structure-related model of altered molecular expression in atrophying muscle to get closer insights into the molecular pathways for improved countermeasure design by e.g. exercise (this study) and/or pharmacological treatment with e.g. NOS/NO-inhibitors/donors or tissue inhibitors of MMPs (TIMPs) that are available (follow-up studies).

#### Identification of main parameters

1. Expression patterns of histochemical NADPH-diaphorase (NOS-activity) and protein immunolocalization of NOS-I, -II and –III at muscle fiber compartments (cytosolic vs. sarcolemmal) under normal (pre-bed rest, BDC-1) and atrophied conditions at the point of profound tissue reorganization just before end of bed-rest (HDT+84).
2. Quantitation of motor end plates in normal vs. atrophied muscle fibers in the control vs. the exercise group identified by alpha-bungarotoxin label (binds to acetylcholine receptor subunit at neuromuscular junctions)
3. Expression patterns of MMP-protein immunolocalization in endo-/perimysial connective tissue cell compartments (i.e., de novo MMP-expression around affected vs. non-affected fiber groups) in normal vs. atrophied fiber groups in pre-study and post-study biopsies.

#### Tested hypothesis

Expression and localization of NOS, MMPs and AChR are highly responsive to ”disuse-induced” muscle atrophy, the spatiotemporal localization pattern parallels structural signs of atrophy and reorganization in muscle fiber compartments, and thus may provide valuable parameters for effective countermeasure design (cf. Title of Proposal)

#### Expected results

- Maintenance and/or relocalization of NADPH-diaphorase/NOS and/or loss of NADPH-diaphorase/NOS that is paralleled by the overall structural changes of muscle fibers in prolonged BR. (NOS expression and distribution parallels manifested atrophy)
- Quantitative changes to occur in the amount of neuromotor endplates in atrophied fiber bundles in the non-exercise (control) versus the excercise group

Maintenance and/or *de novo* reexpression of MMP-2, -7 or -9 at endo-/perimysial connetive tissue in affected fiber fascicles following prolonged bed-rest. (MMP [re-]expression parallels manifested atrophy in the non-exercise bed-rest group as compared to the exercise bed-rest group

The results obtained by the BR study provide closer insights into the cellular and molecular events in ”immobilization-induced” atrophy that might have impact on applied clinical research. As BR provides a valuable model for ”microgravity-induced” atrophy, the results from BR studies (i.e. simulated microgravity) are most relevant for human space flights in applied space research.

Atrophy is particularly challenging for prolonged space flights and effective countermeasures are needed to overcome skeletal muscle waste in microgravity environments.

#### Secondary objectives (not determined)

The secondary objectives of the study aim at providing reliable anatomical markers (NOS, MMP and motor endplate numbers) in skeletal muscle fiber structures that may document the positive outcome of limb exercise (eccentric and concentric limb muscle contraction, cf. Dr. Tesch´s proposal) at a two-days interval during bed rest starting from BR-5 day onwards (i.e., countermeasure trial) to avoid major atrophy as documented by the non-exercise (i.e. control) group.

### Specific inclusion and non-inclusion criteria for test subjects

Healthy male subjects with a general ”body status” comparable to the one of astronauts prepared for a space mission.

### Precise description of the experimental protocol

#### Test descriptions

Tissue: Two punched needle biopsies from each M. vastus lateralis and M. soleus (preferred muscles) before and at the end of the bed-rest study

Number and sex of subjects (Campaigns 1 and 2):

Campaign 1: 10 males, 5 Controls (n=5) and 5 Exercise (n=5)

Campaign 2: 9 males; 5 Controls (n=5) and 4 Exercise (n=4)

Number and days of biopsy/subject during campaign:

Pre-bed rest biopsy: 1x BDC-1(i.e. Control),

bed-rest biopsy: 1x HDT84; (Experimental)

Methods: Histochemistry, Immunocytochemistry, ELISA

Fluorescence and Confocal Laser Microscopy

#### Measured parameters

- Compartmental-specific patterns of histochemical and immuno-cytochemical markers (NOS and MMPs)
- Quantification of motor endplates by counting of α-BGTx-positive structures
- quantification of tissue-specific protein expression (ELISA)

before (BDC-1, control) and at mainfested (HDT+84) stages of ”bed-rest”-induced immobilization in two non-exercise (control) versus two exercise groups.

#### Equipment used (all commercially available)

Freeze-sectioning device (Cryostat, REICHERT, Germany)

Light- and fluorescent microscopy (AXIOPLAN II, Zeiss, Germany)

Confocal Laser Microscopy (strongly proposed because of state-of-the-art ”high-power” resolution of cellular and subcellular marker molecules)

ELISA-Reader: SpectraFluor, TECAN Instruments, Austria

Software: MS-Office 98, WORKS 5.0, StatView-II

#### Description of the test termination criteria of the protocol

Documentation of histochemical and immunocytochemical expression patterns in tissue sections after identical staining protocols as compared to either positive and/or negative control sections.

Determination of motor endplate numbers as stained by acetylcholine-receptor (AChR)-marker alpha-bungarotoxin (Texas-Red-conjugated α-BGTx) in non-exercise (control) vs. excercise groups.

Quantitative determination of MMP by ELISA (control vs. excercise group)

#### Specific medical monitoring

Wound care and palpation at 0-48h postbiopsy

#### Other specific requirements

(not defined)

#### Biological samples handling, etc.

##### Measured parameters

Cellular localization patterns of NADPH-diaphorase, NOS and MMPs as specifically stained cellular structures in skeletal muscle fibers and its surrounding connective parenchyma (endo/perimysium)

Quantitative determination of Texas-Red-conjugated α-BGTx-labelled motor endplates (via AChR-labeling)

##### Methods, equipment and place of analysis

Muscle Biopsy at MEDES, Toulouse, France

##### Labeling, storage and packing of samples

Samples wrapped in thin plastic folio with labels: (Date/muscle/BDC-1, -/HDT+84)

Snap-freezing in prechilled isopentane (Dewar)

Storage in Dewar in liquid N^2^

Transport to PI´s Laboratory (via 24h-Express mail on dry-ice)

Storage: at –80°C

Cryosectioning (10-15µm) and mounting on protein-coated slides, coded slides are stored frozen (-30°C) until use:

- - 1. NADPH-diaphorase histochemistry, NOS-I, -II and –III immunocytochemistry of experimental and control samples, Photodocumentation (Fluorescence, FM, and Confocal Laser Scanning Microscopy, LS-M, strongly proposed)
    2. Labeling of motor endplates by fluorescent-labelled (TEXAS-RED) conjugated α-BGTx (labels AChR)
    3. MMP-immunocytochemistry, MMP-ELISAs, from the rest of the frozen-cut tissue blocks

Photodocumentation (FM at UKE, LS-M at FUB)

#### Data utilisation and analysis, specify

##### Parameters analysed

*Qualitative measurements:*

- Histo/immunohistochemical staining intensities of individual fibers and subcellular structures (graded as strong-moderate-week-negative)
- Quantitative measurements:
- Amount of α-BGTx-positively stained endplates/µm^2^/section
- Amount of stained myofibers/µm^2^, MMP-ELISA (protein expression)

##### Statistical tests

Student´s t-test for paired differences (p<0.05 level), variance analysis

##### Software used

MS-Office 98, Excel, StatView 4.5.,

#### Harzards, side effects, possible inconvieniences, discomforts and contraints to subjects

All voluntary subjects will be biopsied under local anesthesia by a well-trained medical surgeon and under routine medical care. A possible and only contra-indication, i.e., disturbed blood coagulation, will be excluded by blood coagulation tests in the frame of the routine primary diagnostics (e.g., blood status, ECG, internistic control). Due to muscle biopsy interventions, generalized circulation and cardial shock reactions are extremely rare but cannot be fully excluded. However, adverse health effects or any other side effects have not been observed nor reported during previous muscle biopsies in patients or from controlled voluntary bed rest studies.

Acute discomfort of mild local pain due to transient small hematomes (i.e. small effusion of blood) following the needle biopsy may cause typical wound pain which may occur for up to 48 h postbiopsy and may be minimized by adequate postsurgery analgesic oral therapy, if necessary.

### 2 Protocol summary

*Objectives of the study:*

- to investigate compartment-related changes under long-term disuse condition of human skeletal muscle in control versus an exercise group,
- mapping of specific markers (NOS, MMP, AChR) from major cellular compartments susceptible to fiber atrophy and its putative prevention by exercise ,
- to provide a structure-related model of altered expression of relevant molecules from putative signalling cascades and the neuromuscular junction in atrophying muscle to get closer insights into the molecular pathways for improved countermeasure design by e.g. exercise intervals (cf. Tesch proposal) and/or pharmacological treatment with e.g. NOS/NO-inhibitors/donors or tissue inhibitors of MMPs (TIMPs) in a follow-up-study.

*Brief protocol description:*

During the whole study skeletal one pre- and study muscle biopsy will be obtained from two muscle species each, M. vastus lateralis (upper limb knee extensor) and M. soleus (lower limb extensor, calf muscle) from two groups of volunteers (10 controls and 9 exercise) and immediately snap-frozen (-196°C). The frozen samples will be transferred on dry-ice to one of the PI´s laboratory. Frozen tissue blocks will be serially frozen-cut (10-15µm), sections will be mounted on protein-coated slides, postfixed in neutrally buffered formaldehyde, and stained according to the following protocol: (A) NADPH-diaphorase histochemistry (i.e., NOS enzyme activity); (B) NOS-I, NOS-II and NOS-III immunohistochemistry (fluorescent-labeled 2nd AB); and MMP-2, MMP-7 and MMP-9 immunohistochemistry, (C) α-BGTx-labeling to specifically stain AChR for motor endplates. (D) Quantitative MMP-ELISA assays (commercially available kits) from final pieces of cut blocks.

Protocols A-C (at one of PI´s Laboratory); protocol D (at the PI´s Laboratory at the FU-Berlin. Sections will be photodocumented by fluorescence and LS-microscopy at the PI´s laboratory at the FU-Berlin.

*Main medical risks:* Due to interventions by needle biopsies, possible haematome may cause typical wound pain post treatment for up to 48 h (pain treatment). The probability of any effects on the systemic or cardiac circulation (i.e. shock reactions) is extremely low. No adverse acute or side-effects are known or have been reported from comparable humans studies.

### Investigators

**Principal Investigators:** Pr. D. Blottner

**Co-Investigators**:

Mr. Appel R.

Mlle Lück G.H.

Mlle RINDERMANN C.

Mlle Schoser B.G.H.

## Respiratory, circulatory, muscular and metabolism consequences of inactivity in humans.

### Scientific Background

#### Effects of bed rest on muscle function.

The maximal O_2_ consumption (O_2_max) decreases during bed rest as a consequence of the lack of anti-gravitational muscle contractions and/or of prolonged immobilization. This reduction is the result of several physiological and morphological changes. These include a reduction in lower limb muscle mass and cross-sectional area (CSA), which is associated with smaller muscle fibres, and reduced mitochondrial volume and oxidative enzyme activity. Muscle hypotrophy and muscle fibre alterations affect not only the maximal isometric force, but also the maximal instantaneous muscular power. In fact both force and power are reduced to a greater extent than muscle CSA. This may be due to neuromuscular changes, as shown by greater motor unit activation at any given force level during submaximal voluntary isometric contractions, and lower EMG activity during maximal isometric contractions after muscle unloading. In addition, a greater decrease in maximal voluntary isometric force than in maximal electrically-evoked tetanic contraction force was found after one week of simulated space flight. The latter data imply that the maximum number of motor units simultaneously activated at the time when maximal isometric force or maximal instantaneous power are achieved could be decreased after bed rest. A more asynchronous recruitment of a larger number of motor units after bed rest at any given power or force may affect the way a human walks or runs, and thus the energy cost walking and running.

#### Effects of bed rest on cardiopulmonary function function.

Maximal cardiac output ($\dot{Q}$) and O_2_ delivery are reduced to a greater extent than O_2_max. The reduction in maximal $\dot{Q}$ after bed rest may be related to an impairment of the heart performance or to changes in cardiovascular control. Furthermore, the blood volume and fluid volume re distributions, which take place during bed rest, could affect the capacity for O_2_ transport, which is also reduced. Hypovolaemia contributes to cardiovascular deconditioning, together with sympathetic alterations and orthostatic hypotension. The latter may be related also to neuro-vestibular dysfunction and metabolic alterations. The relative importance of plasma volume, red cell mass, myocardial mass and neurocirculatory control in determining the impaired cardiovascular function after bed rest is still unclear. Changes in sympathetic activation occur. Many hormones and transmitters are involved in blood volume and pressure regulation as well (e.g. renin, catecholamines, atrial natriuretic peptide, arginin vasopressin, cyclic GMP, endotheline, nitric oxide). In particular, the role of nitric oxide is poorly understood. The dominating underlying factors may differ between rest and exercise, between supine and upright posture, and between the various phases of bed rest and recovery. A better understanding of the roles played by these factors in the genesis of cardiovascular intolerance after bed rest is a main objective of the proposed study.

It was recently proposed that the oxygen flow in mixed venous blood, or oxygen return reflects the level around which the oxygen transport system is controlled. We hypothesize that the resetting of arterial baroreflex, which is expected to be associated with the reduction of circulating blood volume and to be brought about by a change in the balance between sympathetic and vagal activities, entails a resetting of oxygen transport regulation, as witnessed by a reduction of the oxygen flow in mixed venous blood and by a reduction and a slowering of the first phase of the kinetics of oxygen consumption at exercise onset.

#### Muscle fatigue.

Fatigue is one of the most common and fundamental sensations yet it is one that is poorly understood and poses a major intellectual challenge in exercise physiology. Inappropriate feelings of fatigue are important features of a wide range of diseases and disorders, for example, following injury or surgery, in patients with chronic pain and following many viral infections. Exaggerated feelings of fatigue are also a side effect of a number of common drug treatments including β-blockers and lipid lowering drugs. In many of these conditions there is no adequate explanation for the symptoms in terms of altered skeletal muscle function, whence the creation of the concept of central fatigue. Central fatigue is the condition in which there is an inability to further activation of the musculature due to a deficit in the central nervous system. A better understanding of the nature and origin of fatigue would be an important step in developing treatment strategies including exercise. Evidence for the proposition that central fatigue is an important factor limiting exercise in a variety of situations comes mainly by default in as much as explanations for the sensations of fatigue based on changes in peripheral function are often inadequate. For example, a common side effect of β-adrenoreceptor blockers is the complaint of fatigue and an increased sensation of effort, even during everyday activities. However, detailed studies of muscle fatigue using electrical stimulation to assess peripheral muscle function have shown that β-adrenoceptor blockade have very little effects on the muscles, whence the conclusion that an important role is played by central fatigue mechanisms. Similar conclusions have been attained after studying fatigue in patients affected by chronic heart failure , rheumatoid arthritis and hypothyroidism.

Prolonged inactivity results in a condition where the subject finds even common daily activities very tiring. Many of the symptoms may be explained by changes in the peripheral musculature or the cardiovascular system, as discussed elsewhere. However it remains to be seen whether the peripheral changes are sufficient to fully explain the deficit in performance. By analogy with the clinical examples cited above, it is logical to hypothesize that at least part of the fatigue symptoms after prolonged bed rest may originate from the central nervous system, giving rise to excessive central fatigue.

#### The interest of the duration of the head-down bed rest has also to be justified, in particular for bone changes.

In 1994, many of us participated in the 42-day HDT bed rest campaign, jointly organised by ESA and CNES. At those times, that was the longest bed rest campaign organised under strictly controlled conditions. A comparison of the results with the data obtained in bed rest studies of shorter duration showed that a steady state condition might not have been attained yet, especially for parameters related to muscle mass changes. Although a consistent longitudinal study of the kinetics of muscular changes induced by bed rest could not be performed on that occasion, nor will it be in the present bed rest campaign (indeed the performance of such a study is a major objective of our original project), it is clear that significantly longer times than 42 days are needed to reach such an equilibrium. Three months appear to be, on the basis of present knowledge, a convenient time : we hope that a comparison with data obtained in the 42-day bed rest will allow to get at least an indication of the occurrence of an equilibrium, if not of its time course.

#### Rationale for drug (if used) and dosage selection

No drug requirement is part of our project.

### Objectives and hypothesis

#### Main objective of the protocol

The aims of our project are :

1. To study how the onset of cardiovascular intolerance, with the ensuing resetting of the baroreflex, affects cardiovascular oxygen transport and its regulation, as witnessed by changes in the oxygen flow in mixed venous blood.
2. To determine the effects of cardiovascular intolerance on the first phase kinetics of oxygen consumption and ventilation.
3. To determine the relations between the level of baroreflex setting, and the circulating blood volume or the sympatho-vagal activity.
4. To study the effects of altered force generation and motor unit recruitment after bed rest on the energetics and biomechanics of locomotion.
5. To determine the extent to which central fatigue mechanisms contribute to the loss of performance following bed rest. This will be done as follows : i) study of the alterations in peripheral muscle functions generated by prolonged submaximal exercise, and ii) assessment of the extent to which the activation of glycolysis may depend on catecholamine stimulation during exercise.

#### Identification of main parameters

1. Maximal oxygen consumption.
2. Cardiovascular oxygen transport (oxygen delivery and oxygen return), requiring measurement of cardiac output, heart rate, arterialized blood gas composition, and arterial oxygen saturation.
3. Gas exchange kinetics at the onset and offset of exercise, requiring measurement of breath-by-breath ventilation and expired gas composition.
4. Blood volume.
5. Plasma concentrations of arginin vasopressin, atrial natriuretic peptide, renin, endothelin, cyclic GMP and catecholamines. Urine concentrations of catecholamines, arginin vasopressin and cyclic GMP Blood concentration of nitric oxide.
6. Total energy expenditure, Lipid metabolism, body composition, water turnover and the formation of metabolic water.
7. Heart rate variability and post-ganglionic sympathetic nerve activity.
8. Arterial cardiac chronotropic baroreflex sensitivity and ventricular interdependence.
9. Ventricular mass and cardiac dimension.
10. Muscle architecture, including angle of pennation, fibre length, muscle thickness and muscle cross-sectional area.
11. Energetics and biomechanics of walking and running.

#### Specific hypotheses

1. Orthostatic intolerance implies a resetting of the control system of cardiovascular oxygen transport, as reflected by a lower oxygen flow in mixed venous blood, essentially brought about by a reduction of cardiac output.
2. The amplitude and duration of the first phase of the gas exchange kinetics at exercise onset are reduced after bed rest, because of the reduction in cardiac output (pulmonary blood flow).
3. The resetting of the baroreflexes is the major determinant of the downward resetting of the oxygen transport after bed rest.
4. The resetting of the baroreflex is associated with changes in the balance between sympathetic and vagal activities and with changes in blood volume distribution.
5. The resetting of the baroreflex and of the control system of cardovascular oxygen transport is of smaller amplitude if exercise countermeasures are applied.
6. Orthostatic intolerance is associated with hormonal and electrolyte alterations, which affect body energy expenditure.
7. The onset of muscle hypotrophy entails changes in muscle fibre length and angles of pennation.
8. The occurrence of muscle hypotrophy is associated with motor control alterations, with negative effects on the energy cost and mechanical efficiency of human locomotion.
9. Fatigue symptoms after prolonged bed rest originate, at least in part, from the central nervous system, giving rise to excessive central fatigue.

#### Expected results

Of course the results which we may expect from the performance of this project are related to the hypotheses which will be tested. Our hope is to shed light i) on the interrelations between baroreflex resetting and modifications of the control system of cardiovascular oxygen transport, and ii) on some structural and functional consequences of muscle hypotrophy and of the postulated association of motor control alterations.

From a medical and social perspective, the functional effects of prolonged bed rest simulate some of the physiological or pathological changes which occur in the aged population. For instance, bone deterioration bears an analogy with osteoporosis, muscle atrophy with senile sarcopenia. A deeper understanding of the mechanisms behind bone and muscle deterioration during may contribute to increase our knowledge of the ageing process in humans thereby promoting healthy ageing.

#### Secondary objectives ( if necessary)

Not applicable

### Specific inclusion and non-inclusion criteria for test subjects

Inclusion criteria : as defined in the call for proposals.

Exclusion criteria (positive familiar anamnesis included): Smoking. Cardiovascular diseases. Use of cardiovascular drugs during the last three months prior to the inclusion in the study. Phlebitis and other conditions susceptible to generate thrombosis. Renal or liver failure. Raynaud disease or phenomenon. Hepatitis and other liver pathologies. Diabetes. Asthma and other allergic disease or hypersensitivity. Other obstructive pneumopathies. Restrictive pneumopathies. Haematological diseases and coagulation disorders. Psychiatric pathologies and psychological troubles. Neurologic disorders. Drug and alcohol addiction. Orthostatic intolerance. Venous abnormalities. Infectious diseases. Muscle disorders. BMI < 20 or > 27. Any abnormal value (within –2 SD to +2 SD) of BMD or bone biochemical markers. Any trouble in food consumption

### Precise description of the experimental protocol

#### Tests description (methods, duration, time of day, protocol conditions, etc.)

1. *Gas exchange, maximal oxygen consumption, cardiac output and oxygen transport.* Maximal oxygen consumption ($\dot{V}$O_2_max), cardiac output () and cardiovascular oxygen transport will be determined during dynamic leg exercise on an electrically braked bicycle ergometers, in the course of a combined protocol. The measurements will be carried out at rest and during exercises of increasing intensities.

Starting from 50 watts, power will be progressively augmented by steps of 50 watts, reduced to 25 watts as the expected individual maximum power will be approached. The duration of each work load will be 6 - 7 min.

Oxygen consumption, carbon dioxide output and pulmonary ventilation will be measured continuously on a breath-by-breath basis during the entire protocol, from continuous recording of ventilatory flow by an ultrasonic flowmeter, and of O_2_, CO_2_ and N_2_ fractions measured at the mouth by means of a mass spectrometer. This will allow us to calculate O_2_ and CO_2_ alveolar transfer taking into account the changes of gas lung stores occurring over each breath. By this approach, we will be able to obtain at once i) the steady-state $\dot{V}$O_2_ values required for the analysis of oxygen transport and the determination of $\dot{V}$O_2_max, and ii) a precise description of the gas exchange kinetics at the onset and offset of exercise. For the purpose of the latter analysis, at min 5 of each work load, a micro blood sample (20 μl) will be obtained from the ear lobe for the measurement of blood lactate concentration (Lab), by means of an electro-enzymatic method. This allows to measure the anaerobic lactic contribution to the oxygen deficit incurred during the exercise transient.

Individual $\dot{V}$O_2_max will be established from the plateau attained by the relationship between O_2_ consumption and power above a given power. In case of absence of such a plateau, subsidiary criteria for $\dot{V}$O_2_max establishment will be: 1) a lack of increase in heart rate between two successive work loads; 2) gas exchange ratio values higher than 1.1; 3) Blood lactate values higher that 10 mM. The heart rate will be measured by electrocardiography.

Successive work loads will be separated by 5 min recovery intervals, during which micro-blood samples (20 μl) will be obtained from an ear lobe at min 1, 3 and 5 for determination of the peak lactate concentration attained after each work load.

The cardiac output () and the cardiovascular oxygen transport at the steady state will be determined during the same protocol, carried out after the O_2_max determination. at rest and at the steady state of each work load will be determined by an open circuit acetylene method. To this purpose, during the 6^th^ minute of exercise, the subject will be given to breath a gas mixture containing 1% acetylene, 5% helium, 21% oxygen, balanced with nitrogen, for a total of 20 – 25 breaths. Concentrations of CO_2_, helium and acetylene while breathing the mixture will be continuously measured by the mass spectrometer. While breathing the mixture, the measurements of ventilation and gas exchange will continue normally, as if the volunteer was breathing ambient air. Expired ventilation, expired and alveolar CO_2_ partial pressures, inspired and alveolar acetylene partial pressures (this last corrected for mixing with the ratio of inspired to end-tidal helium), and the blood-gas partition coefficient for acetylene λ. A detailed description of the method is enclosed. λ will be measured on each subject, on a unique occasion, at the beginning of the bed rest campaign, either by gas chromatography, or by mass spectrometry. In both cases, the performance of a 5 ml venous blood sample at rest is necessary.

Immediately after the end of each work load, a total of 60 μl arterialized blood will be sampled from the ear lobe and immediately analysed for arterialized blood pH, Po_2_ and Pco_2_ (50 μl) and for haemoglobin concentration (Hb, 10 μl). Hb will be measured by a photometric technique. Arterialized blood gas composition will be measured by microelectrodes. Arterial oxygen saturation (Sao_2_) will be measured continuously by finger tip infrared oxymetry. Continuous monitoring of the arterial pressure profile will be obtained by means of a Portapres device. The arterial pressure traces will be used to calculate on a beat-by-beat basis during the exercise transients, by the model flow method. Calibration of the will be done at the steady state, against the values obtained with the acetylene method. This data will be used to calculate arterial O_2_ concentration, O_2_ delivery, the stroke volume of the heart, the oxygen flow in mixed venous blood and the total peripheral resistance at the metabolic steady state.

The overall duration of each experimental session would be 1h45, including the tilting exercise described under paragraph 4. The experiment will be repeated twice, once before and once after (currently day R+3) the bed rest period. On each session, a total of 1 ml blood from micro blood samples will be taken. A unique venous blood sample of 5 ml will be also performed on the first occasion, for the determination of the gas-blood partition coefficient of acetylene.

1. *Blood volume regulation*. Blood volume will be measured with a dilution method. To this aim, 10 mg of indocyanine green will be injected in a peripheral vein. The changes in dye concentration will then be monitored by means of a non invasive method (Nihon-Kohden DDG analyser, Japan). A total of 6 ml venous blood samples will also be obtained on the same day. These samples will be analysed for plasma concentrations of arginin vasopressin, atrial natriuretic peptide, renin, endothelin and cyclic GMP, all by radioimmunoassay. In addition, plasma catecholamine concentrations will be measured by high pressure liquid chromatography. Moreover, a urine sample will be taken for determining the urine concentrations of catecholamines, arginin vasopressin and cyclic GMP. Blood nitric oxide will be determined on a further 4 ml blood samples by a specific analyser.

The duration of the blood volume experiment will be 30 min, plus blood sampling. A total of 10 ml of venous blood will be taken on each occasion. The experiment will be performed once before the bed rest, twice during bed rest (HDT-16, HDT-58), and three times during recovery (R+1, R+11, R+29). The performance of the second part of this protocol (total energy expenditure) requires 4h. On each occasion, a total of 5 ml venous blood samples will be taken. The experiment will be carried out once before the bed rest, three times during the bed rest (HDT-16, HDT-58, HDT-90), and once after the bed rest (R+11).

The other part of the study is described in paragraph 6.10.5.

1. *Sympathetic function analysis.* Sympathetic nerve activity will be measured in the peroneal nerve of the leg just below the knee. This is done by inserting a tiny needle directly into the nerve (nerve puncture). After having localized the nerve, we will introduce a tiny (0.2 mm in diameter), sterile Tungsten-wire needle (an electrode) through the skin. We connect the wire needle to the electrical stim­ulator and advance the needle into the nerve with the aid of week electrical stimulation (3 volts, which is equivalent to 2 small radio batteries). When the tip of the needle (5 µm) has entered the nerve, we will turn the electrical stimulator off and make minor adjustments in the position of the needle until characteristic sympathetic bursts appear on the monitors and the recordings can begin. The origin of the nerve-traffic will be verified by classical responses to a Valsalva maneuver and no response to arousal stimuli such as a loud noise. Once in a correct position, the recording needle will subsequently remain in this position for the duration of the study. A second needle serves as a reference electrode and is inserted just under the skin. At the end of the study, we simply remove the needles by pulling them out of the skin. The peroneal nerve is the most frequently used for microneurographic studies of sympathetic nerve activity and is also used in the present study because: 1) It is quite accesible and associated with an identifiable landmark (the peroneal head); 2) it is frequently possible to obtain a “pure” muscle nerve site with no admixture of impulses from cutaneous nerve fibres, which makes interpretation of results easier; 3) it is often possible to obtain a stable site, with relatively little intereference and artifact related to EMG-activity, as long as the leg is relaxed; and 4) the muscle sympathetic nerve activity from the peroneal nerve is representative. On the same occasion, the heart rate (by electrocardiogram), the arterial blood pressure (by Portapres) and the respiratory rate (by an elastic belt placed on the thorax) will be monitored in continuous. The experiment will be carried out at rest, with the subject in supine position on a tilting table. Occasionally, the subject will be tilted to 30° for a short time (2-to-5 min).

The duration of this experiment will be two hours. The experiment shall be performed on the same occasions and days as for the heart rate variability determinations (Pagani).

1. *Baroreflex and cardiac performance during rest and exercise.* **1)** Prior to the graded exercise protocol described here above, and in conjunction with it, the lowest work load will be performed in supine posture, and with a series of rapid tilts between upright and supine posture to determine baroreflex and ventricular interdependence. Continuous monitoring of alveolar gas exchange on a breath-by-breath basis (see under point 1), heart rate (electrocardiography), arterial blood pressure (Portapres) and estimated thoracic fluid volume (trans-thoracic electrical impedance) will be performed during repeated rapid whole-body tilts during rest and exercise at 1/3 of O_2_max. The tilting ergometer will be provided by the Laboratory of Environmental Physiology, Department of Physiology and Pharmacology, Karolinska Institute : indeed it is the same home-made piece of equipment which was used in the 1994 42-day bed rest. Tilt interval is 1-2 minutes. Arterial cardiac chronotropic baroreflex sensitivity is determined from the relationship between associated heart-rate and baroreceptor arterial pressure (98-100). Ventricular interdependence is estimated from tilt-induced pulse-pressure transients (98-100). **2)** In a separate resting session, ventricular interdependence is also assessed from echocardiographic determinations of septal movements during a Müller manoeuver at rest (14, 54). In addition, ventricular interdependence is assessed from beat-by-beat aortic blood flow velocity, as determined by Doppler ultrasound during Müller manoeuvers at rest (103, 53). **3)** In a third separate session left ventricular mass and cardiac dimensions are determined with echocardiography.

The first part of this protocol will be performed in conjunction with experiment 1. The second and third part will be performed jointly on a separate occasion, once before, and once after (R+4?) the bed rest. The duration of the joint part 2+3 will be 40 min.

1. *Muscle architecture.* Angle of pennation, fibre length and distance between aponeuroses (muscle thickness) of the quadriceps femoris and gastrocnemius muscles will be determined from images obtained with a real-time computerized sonograph using a 7.5 MHz probe (Acuson 128 XP, USA). The physiological cross-sectional area (*A_p_*) of these muscles will be calculated as previously described (77). These measurements will be carried out at rest and during isometric contractions at 20, 40, 60, 80 and 100% of the maximal voluntary contraction force (MVC). Force will be measured by connecting the knee or the footplate to a force transducer (FN 3030, FGP Instrumentation, France).

The duration of this protocol will be 30 min. It will be performed once before, six times during (HDT-2, HDT-7, HDT-14, HDT-28, HDT-58, HDT-87) and 4 times after (R+2, R+12, R+30, R+90) the bed rest.

1. *Energetics and biomechanics of locomotion.* The experiments will be carried out on a treadmill at three speeds during walking (3.0, 4.5, 6.0 km/h) and running (8.0, 9.5, 11.0 km/h). The measurements will be carried out before and after the bed-rest. The energy cost of locomotion will be determined as the ratio of the measured net steady state $\dot{V}$O_2_ to the speed. At each speed, $\dot{V}$O_2_ will be measured during the 5th min of exercise, either by means of a metabolic cart (Cosmed K4b2, Italy), or by the standard open circuit method. In the latter case, expired gas will be collected into Douglas bags and subsequently analysed for gas composition and volume. A paramagnetic O_2_ analyser, an infrared CO_2_ analyser and a dry gas meter will be employed. Blood lactate will also be determined from ear lobe micro-samples, and heart rate monitored, as previously described (See section 1). Motion capture will be made by using an opto-electronic device composed by 4 CCD cameras capable to detect and measure at 100 Hz the 3D co ordinates of 18 reflective markers put on the relevant joints of both sides of the subjects. After calculation of the path of the body centre of mass, the kinetic (KE) and potential (PE) energy will be obtained. The mechanical external work will be obtained from the sum of KE and PE. The energy recovery, a parameter accounting for the exchange of potential and kinetic energy, will be measured to evaluate the pendulum-like mechanics of walking. An estimate of the efficiency of locomotion will be obtained by dividing the mechanical work by the metabolic energy cost. The stride frequency will also be computed.

The duration of this experiment will be one hour. A total of 0.1 ml of blood will be sampled. The experiment will be performed twice, once before and once after the bed rest (R+4).

#### Measured parameters

**Protocol 1 :** Breath-by-breath alveolar gas exchange; heart rate; arterial oxygen saturation; steady-state cardiac output by acetylene; beat-by-beat arterial blood pressure; blood lactate concentration; arterialized blood gas composition; blood haemoglobin concentration; blood-gas partition coefficient for acetylene. **Protocol 2** : Blood volume; plasma concentrations of arginin vasopressin, atrial natriuretic peptide, renin, endothelin and cyclic GMP; plasma catecholamine concentration; urine concentrations of catecholamines, arginin vasopressin and cyclic GMP; blood nitric oxide; total energy expenditure, body composition, water turnover and the formation of metabolic water; blood glucose, non-esterified fatty acids, keton bodies, leptin and insulin plasma concentrations; daily hormonal state (growth hormone, cortisol); kinetics of glucose metabolism; resting energy expenditure. **Protocol 3** : Sympathetic nerve activity in the peroneal nerve. **Protocol 4** : Breath-by-breath basis alveolar gas exchange, heart rate (electrocardiography), arterial blood pressure (Portapres) and estimated thoracic fluid volume (trans-thoracic electrical impedance). **Protocol 5** : Angle of pennation, fibre length and distance between aponeuroses by computerized sonography. **Protocol 6** : Oxygen consumption at the steady state; mechanical work during walking and running.

#### Equipment used

Infrared CO_2_ analyser (LB-2, Leybold Haereus, Germany); Electrically-braked cycle ergometer (Ergoline 800S, Germany); Paramagnetic O_2_ analyser (Oxynos 1C, Leybold-Haereus, Germany); Electroenzymatic lactate analyser (ESAT 6661 Lactat, Eppendorf, Germany); Dry gas meter (Singer DTM 15, USA); Cardiotachograph (Polar, Finland); Mass spectrometer (Balzers Prisma, Lichtenstein); Haemoglobin analyser (Haemophot, space adapted device after Hemocue, Sweden); Oxygen saturation meter (MicrO2, space adapted device after Siemens, Germany); Continuous blood pressure monitor (Portapres, TNO, The Netherlands); Blood gas analyser (TBD, commercial item); Ultrasonic flowmeter (Tuba, GHG, Switzerland); Electrocardiograph (Schiller Cardiovit A2-plus, Switzerland, or TBD); Blood volume system (Nihon-Kohden DDG analyser, Japan); Nerve traffic analyser (Iowa Medical Instruments, USA); Square pulse stimulator and isolation unit (Grass Instruments, USA, or similar); Isolated tungsten micro-electrodes and preamplifier (Iowa Medical Instruments, USA); Pen electrode (Iowa Medical Instruments, USA); Respiration belt (PowerLab, USA); Tilting ergometer (home made, from Karolinska Institute, Stockholm); Doppler ultrasound system (TBD, commercial item); Transthoracic electrical impedance meter (TBD, commercial item); Real-time computerized sonograph with a 7.5 MHz probe (Esaote Biomedica, Italy); Force transducer (FN 3030, FGP Instrumentation, France); Motor driven treadmill (TBD, commercial item); Motion capture analyser (Elite System, BTS, Italy);

Various types of classical software packages will be used, including AcqKnowledge Biopac, PowerLab, and Labview. Public domain available software will be used for motion analysis. Specific softwares for equipment running is delivered with the mass spectrometer, the microneurography system, the Portapres, the EMG system, the Doppler ultrasound, and the ECG acquisition system.

#### Description of the test termination criteria of the protocol

This paragraph applies to exercise protocols. The locomotion protocol will be only submaximal, and, unless premature ending is necessary, will be completed with the performance of three walking and three running speeds. All other protocols will be ended at volitional exhaustion. Nevertheless, the following criteria of premature exercise ending will be applied : 1) strong dyspnea, paleness and confusion; ii) precordial pain; iii) arrhythmias; iv) PQ interval increase; v) ST displacement by >2 mm downward and > 1.5 mm upward; vi) systolic blood pressure above 230 mmHg and/or diastolic blood pressure >120 mmHg; vii) systolique blood pressure decrease upon an increase in work load. For these reasons, the ECG and the arterial blood pressure will be anyway measured during each exercise protocol which will normally be ended at volitional exhaustion.

#### Specific medical monitoring (ECG, blood pressure monitoring, etc.)(if necessary)

The monitoring of ECG and arterial blood pressure is carried out during exercise testing. In most cases, this monitoring is part of the experimental protocol as well. If not, it will be implemented, in order to check for the test termination criteria.

#### Other specific requirements (dietary, conditions of the experiment sessions, etc.)

None

#### Biological samples handling, preservation conditions, transport

In general, blood samples will be immediately analysed on the spot. When different, please refer to specific paragraphs (see chapter 6.10.4.1, para 3).

#### Data utilisation and analysis, specify

The data will be presented in Tables and Figures. Paired t-test or ANOVA will be performed, when necessary. The Wilcoxon rank test or the Bonferroni test will be used to test the significance of intragroup differences. The level of significance will be set at p<0.05. When needed, linear regressions will be calculated by the least square method. Comparison among regression lines will be carried out by analysis of covariance.

The results of the study will be used for academic purposes : redaction of scientific articles, reports to the supporting agencies and conferences or oral and poster presentations at scientific meetings. No other use of the results will be made.

#### Hazards, side effects, possible inconveniences, discomfort and constraints to subjects

1. *Hazard cause :* Occurrence of heart ischaemia and cardiac arrhythmia during exercise, especially in maximal exercise tests. *Effects :* Acute heart attack. Resuscitation of the subjects may be necessary with subsequent hospitalisation in critical care unit. *Severity :* High. *Probability :* Low, may become very low if inclusion/exclusion criteria are strictly respected in the selection of volunteers . *Protection measures :* Exclusion criteria; premature exercise end as defined under 6.10.4.4. and 6.10.4.5. A reanimation kit should be made available by the medical follow-up responsibles.
2. *Hazard cause :* Transmission of microbial or bacterial infection due to either spontaneous growths in the breathing apparatus or to contamination which may be introduced by subjects who are, or become, infected during the course of the study. *Effects :* Subjects’ illness due to the microbial or fungal agent inhaled from the breathing apparatus.*Severity :* Low. *Probability :* Very low. *Protection measures :* Personal mouthpiece are provided for each subjects. These are interchangeable on the breathing apparatus and they will be sterilised after each experiment.
3. *Hazard cause :* Flammability of acetylene, and its sedative effects at high concentrations. *Effects :* Fire or explosion. Inert gas sedation at high concentration. *Severity :* Reasonable. *Probability :* Very low. *Protection measures :* Acetylene will be used at a concentration which is not flammable (1%). Also, sedative effects of acetylene are likely to occur only at concentrations above 10%. Pre-mixed certified cylinders will be used.
4. *Hazard cause :* Fall from an ergometer. *Effects :* Traumas. *Severity :* Low. *Probability :* Very low. *Protection measures :* None, except for fixing the subject by belts on the tilting ergometer and on the Cybex ergometer.
5. *Hazard cause :* Electric shock. *Effects :* Electric shock hurts and traumas *Severity :* Reasonable. *Probability :* Very low. *Protection measures :* All the equipment to be utilized will be CE certified. Moreover, i) I/O devices, either battery or main-power supplied, will be fully electrically isolated; ii) leakage current will be less than 100 microamps when surface skin electrodes are used; iii) leakage current of metallic parts of the devices directly accessible by subject or operator will be less than 100 microamps; iv) devices will be protected against the damages that may be caused by earth loops; v) general power network will be protected against the possible overtensions generated by the devices themselves.
6. *Hazard cause :* Hurting during blood sampling. *Effects :* Bleeding. *Severity :* Very low. *Probability:* Very low. *Protection measures :* None.

### Protocol summary (one page), written on a separate sheet

The general aims of this project is to investigate various consequences of prolonged bed rest, mediated by cardiovascular deconditioning or by muscle hypotrophy. The following hypotheses will be tested : Specific aims are i) to study how the onset of cardiovascular intolerance, with the ensuing resetting of the baroreflex, affects cardiovascular oxygen transport and its regulation, as witnessed by changes in the oxygen flow in mixed venous blood and in the first phase kinetics of oxygen consumption and ventilation upon exercise onset; ii) to determine the relations between the level of baroreflex setting, and the circulating blood volume or the sympatho-vagal activity; iii) to study the effects of altered force generation and motor unit recruitment on the energetics and biomechanics of locomotion. It consists of 6 experimental protocols, divided into two groups : cardiopulmonary and muscle protocols. The former group includes the assessment of maximal oxygen consumption; gas exchange, cardiac output and cardiovascular oxygen transport during exercise of increasing loads; fluid regulation, water balance and lipids metabolism; sympathetic nerve activity; baroreflex and cardiac performance during rest and exercise. The latter group of experiments includes a study of muscle architectural changes following the onset of hypotrophy; a biomechanical and energetic analysis of walking and running. In the course of this protocols, the following major parameters will be determined : i) maximal oxygen consumption and cardiovascular oxygen transport (oxygen delivery and oxygen return), requiring measurement of cardiac output, heart rate, arterialized blood gas composition, and arterial oxygen saturation; ii) gas exchange kinetics at the onset and offset of exercise, requiring measurement of breath-by-breath ventilation and expired gas composition (these parameters will be measured jointly in the course of experiment 1); iii) blood volume and the plasma concentrations of arginin vasopressin, atrial natriuretic peptide, renin, endothelin, cyclic GMP and catecholamines plus the blood concentration of nitric oxide; iv) urine concentrations of catecholamines, arginin vasopressin and cyclic GMP; v) total energy expenditure, body composition, water turnover and the formation of metabolic water; vi) post-ganglionic sympathetic nerve activity; vii) arterial cardiac chronotropic baroreflex sensitivity; viii) ventricular interdependenceand ventricular mass and cardiac dimension. In addition, the study of muscle architectural changes include an analysis of the angle of pennation, fibre length, muscle thickness and muscle cross-sectional area of lower limb muscles. Finally, the energy cost of and the mechanical work during of walking and running, will be studied. These protocols will be performed before and after the bed rest period. However, protocols not involving exercise performance will also be carried out on some occasions during the bed rest.

### Body composition and Lipid metabolism (Annex of point 3 of Protocol 6.10)

### Scientific Background

#### Problem presentation, Literature data including references, Protocol justification according to current scientific knowledge

Head-down bed rest induces endocrine and metabolic changes that affect the body composition and the energy requirements necessary to maintain health (1,2,3). Recent data of our group suggests, but does not demonstrate, that fat metabolism is strongly altered (4) as in flight (5.6). Because fat is the highest energy density tissue of the organism, a better understanding of its adaptation to simulated weightlessness is required to plan long-term space flight and make accurate estimates of the astronaut's energy requirements (7).

1) Blanc S, Normand S, Ritz P, Pachiaudi C, Vico L, Gharib C, Gauquelin-Koch G. Energy and water metabolism, body composition, and hormonal changes induced by 42 days of enforced inactivity and simulated weightlessness. J. Clin. Endocrinol. Metab. 83 (12): 4289-4297, 1998.

2) Blanc S, Normand S, Pachiaudi C, Duvareille M, Gharib C. Leptin responses to physical inactivity induced by bed rest. Am J Physiol 279(3):R891-8, 2000.

3) Gretebeck RJ, Schoeller DA, Gibson EK, Lane HW. Energy expenditure during antiorthostatic bed rest (simulated microgravity). J. Appl. Physiol. 78(6) : 2207-2211, 1995.

4) Blanc S, Normand S, Pachiaudi C, Laville M, Fortrat JO, Gharib C. Fuel homeostasis during physical inactivity induced by bed-rest. J Clin Endocrinol Metab 85(6): 2223-33, 2000.

5) Lane HW, Gretebeck RJ, Schoeller DA, Davis-Street J, Socki RA, Gibson EK. Comparison of ground-based and space-flight energy expenditure and water turnover in middle-aged healthy male US astronauts. Am. Clin. Nutr. 65 : 4-12, 1997.

6) Stein TP, Leskiv MJ, Schulter MD, Hoyt RW, Lane HW, Gretebeck RE, LeBlanc AD. Energy expenditure and balance during spaceflight on the space shuttle. Am. J. Physiol. 276(6) : R1739-R1748, 1999a.

7) Lane HW, Gretebeck RJ, Smith SC. Nutrition, endocrinology, and body composition during space flight. Nutr. Res. 18 (11) : 1923-1934, 1998.

#### The interest of the duration of the head-down bed rest has also to be justified, in particular for bone changes.

Despite the fact that we observed perturbations in lipid metabolism as assessed through indirect calorimetry after only 7 days of bed rest, changes in the whole body composition requires long term experiments. In other words, the period of the proposed bed rest is highly suitable to observe changes in body composition, which are beyond analytical considerations.

#### Rationale for drug (if used) and dosage selection

No drug used

### Objectives and hypothesis

#### Main objective of the protocol

The objectives of the experiment are to better understand the mechanisms of body composition changes observed during space flight and simulated microgravity by providing new insights in the partitioning of dietary fats between oxidation and storage. The role of length vs. saturation will be tested. A corolla to this protocol is to validate bio-impedance spectroscopy to ensure accurate estimates of body composition for further use in actual microgravity.

#### Identification of main parameters

- Energy expenditure
- Substrate oxidation
- Dietary fat oxidation
- Sympathetic nervous system activity
- Body composition

#### Tested hypothesis

Based on the above considerations and on our experience in stable isotope methodologies we hypothetized:

1) that the physical inactivity induced by bed rest leads to a positive fat balance through a partitioning of dietary fat toward storage rather than oxidation,

2) that the magnitude of dietary fat stored is dependant on the saturation degree and length of the fatty acids,

3) that exercise may counteract the changes induced by bed rest and can be used as a reliable countermeasure,

3) that bio-impedance spectroscopy is an accurate tool to estimate body composition during bed rest.

#### Expected results

Beside the importance of bed rest as a model of weightlessness, it is the only model to achieve reliable physical inactivity. There is now evidence that inactivity plays a key role in the development of obesity and insulin-resistance. However, such an implication is indirect and based on the positive effect of exercise. The proposed experiment may help to understand how physical inactivity interferes with the substrate oxidation and leads to fat accumulation. Moreover, by comparing two different fatty acids (length / saturation: oleate vs. palmitate), we will be able to potentially demonstrate if the type of lipid affects the partitioning between storage and oxidation. Specific diet and exercise protocols may be derived as countermeasures to prevent or reduce the deleterious effects of inactivity.

#### Secondary objectives ( if necessary)

The concomitant use of bio-impedance spectroscopy with the tracer determination of body composition will allow us to validate a reliable technique to measure body composition in flight as to estimate the effectiveness of the countermeasures achieved in space.

#### Specific inclusion and non-inclusion criteria for test subjects

No other specific criteria than those details in the main protocol are needed. We just required no claustrophobia because of the canopy used in indirect calorimetry and a physical activity level similar between the subjects.

#### Precise description of the experimental protocol

### Tests description (methods, duration, time of day, protocol conditions, etc.)

##### Body composition:

The body composition will be determined five times (twice in the control period and during the bed rest, once during the recovery period). The total duration of the protocol is 4 hours in the morning. Total body water, and extra-cellular volume will be determined by isotope dilution of 18-oxygen labeled water and bromide (doses mentioned in the figure below). After a baseline blood sample of 3-mL, these tracers will be orally ingested. Thereafter, two other blood samples will be performed after 3 and 4 hours of equilibration. At the last blood sampling a bio-impedance scan will be performed to measure non-invasively the same parameters by placing electrodes at the knee and elbow levels. In parallel, blood volume will be determined through the injection of the indothiocyanin green (detailed elsewhere). During the four hours of the protocol, the subjects will not be allowed to drink water or eat.

ISNERE BODY COMPOSITION

##### Fatty acid metabolism:

The protocol, which is completely described in the figure below, will be conducted twice in each subject: before and at the end of the bed rest. Two other tests will be performed one week before the others to achieve an acetate correction factor justified below. The protocol complete duration is 9 hours (1 hour in baseline and 8 during the test) and is based on an oral load of lipids, oleate and palmitate labeled with stable isotopes (^13^C and ^2^H, respectively) mixed with the meal. The oxidation of ^13^C oleate is determined by the ^13^C enrichment of expired CO_2_, sampled every 30-min. However, due to sequestration of the label in the tricarboxylic cycle, a correction factor is needed to account for such an underestimation of the fatty acid oxidation rate. The correction factor is deduced by performing the same test using a ^13^C acetate load one week before the lipid test. During beta-oxidation, the deuterium of the ^2^H palmitate will be lost as deutered water, which will mix the total body water pool. Therefore the recovery of ^2^H_2_O in urine is a measure of the palmitate oxidation.

During the duration of the protocols, indirect calorimetry will be performed to measure the whole body substrate oxidation and heart rate variability will allow us to determine the sympathetic nervous system.

Excepted for baseline blood samples, the protocols are totally non-invasive and based on stable isotopes i.e. not radio-labeled.

##### Measured parameters

With the above protocol we will determined the following parameters:

Body composition:

- Total body water
- Extra-cellular water volume
- Intra-cellular water volume
- Blood volume
- Fat mass
- Lean body mass

Lipid metabolism:

- Energy expenditure
- Thermogenic effect of the meal
- Respiratory quotient
- Net oxidation of lipid, carbohydrate and protein
- Oxidation of exogenous lipids
- Basal levels of insulin, glucose NEFA triglycerides and ketone bodies

#### Equipment used- Hardware description (trademark, model, …), specify if experimental equipment is commercially available or self built,

- A metabolic card (Deltatrac, Ohmeda, Helsinki, Finland)
- A impedancemeter (Xitron Inc, California, USA)
- A Finapress card to record continuous blood pressure

#### Description of the test termination criteria of the protocol

No other specific requirements than those explained in the general protocol are needed. It is just required that the subjects eat the same meal each evening preceding the test.

#### Biological samples handling, preservation conditions, transport

##### Measured parameters

Are described below only the samples that need storage for further analysis

**Body composition**

-Isotopic enrichment of plasma in 18-oxygen water, bromide and indothiocyanin green.

**Lipid metabolism**

-Isotopic enrichment in ^13^C of CO_2_ and deuterium enrichment in urine.

-24-hour urine : catecholamines, cortisol, C-peptide, urea and creatinine

-blood samples only before the lipid oral loads (not acetate tests) for triglycerides, NEFA, glucose, glycerol, beta-hydroxybutyrate, insulin.

##### Methods, equipment and place of analysis

-Deuterium, 18-oxygen water and ^13^CO_2_ enrichments will be determined by isotope ratio mass spectrometry, both at Lyon, France and Madison WI USA (laboratoire Pr Laville et Pr Schoeller).

-Bromide will be determined by HPLC at Madison WI USA (laboratoire Pr Schoeller)

-All metabolites and hormones will be determined by classical methods used in our laboratory by RIA, HPLC or enzymology in Lyon, France (Pr Gharib).

##### Labelling, storage and packaging of the samples

-Breath tests are stored in Vacutainers

-Blood will be centrifuged and plasma freeze at –80°C until analysis.

#### Data utilisation and analysis, specify

##### Parameters analysed

All the parameters described before will be analyzed.

##### Statistical tests

**Body composition**

The variables will be analyzed by a multi-way analysis of variance with repeated measures with time (before and after bed rest) exercise (control vs. exercise) as main effects.

The validation of bio impedance spectroscopy against direct isotope determination of body composition will be determimed by a classical Bland & Altman test (Bland JM, Altman DG. Statistical methods for assessing agreement between two methods of clinical measurement. Lancet i : 307-10, 1986) to assess agreement between two parameters.

**Lipid metabolism**

The variables will be analyzed by a multi-way analysis of variance with repeated measures with time (before and after bed rest) exercise (control vs. exercise) as main effects. Fatty acid type (oleate vs. palmitate) will be included when required in the above analysis.

##### Software used

StatView 5.01 (SAS institute, CA USA)

#### Hazards, side effects, possible inconveniences, discomfort and constraints to subjects

- Any potential hazard, adverse event, discomfort or constraint for the volunteers during the study (BDC, HDT and post RDC) has to be clearly described as well as the precautions that will be taken to avoid them. Please assess the severity (minimal or reasonable) and the probability (high, medium, low, extremely low) of each of them.
- In particular, if radiation will be received by the experimental subject due to either X-rays or use of isotopes, please indicate dose per session, number of sessions and total dose per subject for the whole experiment. If appropriate, a table summarizing venipuncture and blood volume (amount per session, number of sessions and total per subject for the whole experiment) should be provided. Test termination criteria will be specified for specific tests.
- All serious side effects should be reported immediately to the responsible medical offices and promptly be followed by detailed written reports.

The protocol proposed is totally non-invasive except for the blood samples performed and for which the potential risks are explained in the main protocol of the bed rest.

The tracer orally ingested (^13^C oleate, ^2^H plamitate, H_2_^18^O, bromide) are stable isotopes and therefore are not radiolabeled materials. Actually, no toxicity is known (Jones PJ et al. Stable isotopes in clinical research: safety reaffirmed. Clin; Sci. 80: 277-280, 1991).

Indirect calorimetry measurements can result in small disconfort due to confinement under the canopy. However, we use this method for numerous years and no problems have been encountered.

Concerning the sympathetic nervous system recording as the impedancemeter system, all the measurements are non-invasive and rely on electrode placements. These can results in small and temporary irritations, which are rare.

#### Protocol summary (one page), written on a separate sheet

Objectives, brief protocol description, main medical risks

Space flight induces deleterious changes in muscles, fat, bone and fluids that affect astronaut’s body composition and the energy requirements necessary to maintain their health.

The objectives of this protocol are a better understanding of the body composition changes occurring in space. To this purpose we propose 1) to give new insights in the role of physical inactivity achieved during bed rest in the partitioning of dietary fat between storage and oxidation, 2) to determine if such partitioning is function of the length and saturation of the fatty acids, 3) to test the effectiveness of physical exercise as a reliable countermeasure and 3) to validate bioimpedance spectroscopy a reliable tool to measure body composition in flight.

Oral loads of lipids labeled with stable isotopes (^13^C oleate and ^2^H palmitate) mixed within the breakfast will be conducted before and during the bed rest. During 8 hours following the load, breath tests, indirect calorimetry, urine collection, basal blood sample and heart rate variability will allow us to determine the oxidation of exogenous fat, the net oxidation of substrates, hormones (insulin) and metabolites (ketone bodies, glucose, NEFA, triglyderides), and the sympathetic nervous system activity. Two other tests will be performed one week before the lipid loads and in the same condition but using a ^13^C acetate load in order to correct the loss of ^13^C in the tricarboxylic cycle which underestimate the true oxidation rate of oleate and palmitate. Body composition will be determined two times in control period, two times during bed rest and once during recovery. Total body water and extra-cellular water volume will be assessed through H_2_^18^O and bromide dilution (oral ingestion). Blood volume will be assessed by indothyocianin die dilution. 3 and 4 hours after the ingestion a blood sample of 3-mL will be collected. These variables will be confirmed by bio-impedance spectroscopy.

Except one blood sample per lipid test (5-mL, not for acetate test) and three per body composition test (9-mL total), the protocol is totally non-invasive and is based on stable isotopes which are non radio-labeled materials and totally safe. No medical risks others than those linked to venous puncture can be drawn.

### Investigators

**Principal investigator :** Dr. Guido Ferretti

**Co-investigators**

Pr. Antonutto G. Mlle Normand S.

Dr. Azabi M.K. Pr. Dave R. Pendergast

Dr. Binzoni T. Dr. Mikael Sanders

Mr. Blanc S. Pr. Scholler D.A.

D. Capelli C Me Zamparo P.

Dr. Cautero M.

Dr. Custaud MA.

Dr. D'Atona G

Pr. di Prampero P.E

Dr. Fortrat J.O.

Dr. Fusi S.

Dr. Claude Gharib

Dr. Lador F.

Dr. Laville M

Pr. Dag Linnarsson

Dr. Maganaris C.N

Dr. Millet C

Pr. Albero Minetti

Mr. Moia C

Dr Marco V. Narici

## Assessment of lower and upper limbs vascular hemodynamics during long term bed rest.

### Scientific Background

#### Problem presentation

Exposure to actual or simulated weightlessness results in changes in cardiovascular hemodynamics (cardiovascular deconditioning) which contribute to orthostatic intolerance (OI) observed after space flights or ground simulation studies. It is important to know what cardiac or vascular factors mainly contribute to post-flight OI, because this knowledge will allow the definition of accurate, specific and adapted in-flight and post-flight countermeasure techniques to apply to spationauts, in order to protect their health and to enhance their safety.

The aim of the present project is to carry on previous studies made in our laboratory which tend to demonstrate that vascular deconditioning, especially venous deconditioning, is a key contributory factor to post-flight or post-bed rest OI. The aim of this AO is also to propose research projects which will meet the main objectives which are as follows :

- collecting standard baseline data before, during and after long term bed rest
- validation of physical and/or pharmacological countermeasures.

#### Literature data including references

In the following, we will present a state of art picture of the problem of the definition of countermeasures in relation with vascular deconditioning in microgravity, i.e. 1/ vascular factors contributing to post-flight or post-bed rest OI, 2/ physiological factors accounting for venous deconditioning in microgravity, and 3/ characteristics of vascular deconditioning in weightlessness.

*Vascular factors contributing to post-flight or post-bed rest OI.*

Among the factors which possibly contribute to OI after space flights or bed rests, peripheral vascular deconditioning, especially venous deconditioning, has recently been shown to play a key role in the occurrence of OI after bed rest (Pavy Le Traon et al 1999). The authors tested in 47 healthy subjects who participated in bed rest experiments different physical, physiological and experimental parameters thought to be associated with orthostatic cardiovascular responses, and correlated these parameters with their ability to complete or not complete the post-bed rest orthostatic tests. They concluded, by using logistic regression analysis, that the occurrence of OI was associated with greater height, low resting blood pressure, absence of countermeasures and greater changes in resting lower limb venous distensibility throughout the bed rest. In addition, in the framework of the experiments performed onboard the Mir station, with the space functional exploratory laboratory PHYSIOLAB, we evidenced (results not yet published) that the cosmonauts which exhibited greater lower limb venous compliance changes during flight also presented longer periods of OI after flight. Therefore, an obvious link exists between alterations of lower limb venous distensibility and the occurrence of post-bed rest or post-flight OI.

Other vascular and non vascular factors are able to contribute to post-flight OI. The inability of the cardiovascular regulation to maintain an adequate brain perfusion may be related to different phenomenons: Lack of lower limb arterial vasoconstriction, reduction of the venous return (muscle atrophy, increase venous compliance), hypovolemia, loss of sensitivity of the baroreflex increased (Arbeille et al 1998, and 1999, Buckey et al 1996, Convertino et al 1990, Fritsch et al 1992, Sigaudo et al 1996, Robertson et al 1996, Monos et al 1997, Herault et al 2000).

Lower limb arteries and splanchnic vein compartment responses to orthostatic stress. The lack of arterial vasoconstriction in the lower limbs was found in most of the intolerant subjects or cosmonauts even though there was no evident signs (clinic, HR, BP) of orthostatic intolerance. On the other hand some tolerant subjects do not show a marked increase of the femoral vascular resistance (nor pre neither post HDT or flight) whereas the cerebral to femoral flow ratio increases markedly as expected in normal subjects. The reduction of the femoral flow still exists but is no more associated with a marked increase in femoral resistance. We suggest that the reduction of the femoral flow may be more related to the increase in flow at the splanchnic level (acting like a by pass) than to the increase in lower limb vascular resistance. In both cases the cerebral to femoral flow ratio allows to quantify the efficiency of the peripheral flow redistribution and allows to identify the intolerant subjects even in absence of blood pressure drop.

When changing the position of the body (sitting to standing position) there is an important fluid shift toward the legs which may reduce the brain perfusion. The adaptation of the cardiovascular system consists of an increase of the sympathetic nervous system stimulation on the heart, peripheral vessels, and adrenal glands via rapidly acting reflexes. Nevertheless the lack of lower limb arterial vasoconstriction that appears (Arbeille et al 98) despite the increase in sympathetic activity, suggests that there could be a modification of the distal perivascular nervous network or a change in the number or sensitivity of the neuroreceptors at this level. Such hypothesis was tested on rats suspended by the tail (for microgravity simulation) in which the density of perivascular nervous fibers reduced in the lower limbs, and rats suspended by the head (for hypergravity simulation) in which the density of perivascular nervous fibers increased in the lower limbs (Ma et al 1997, Radionov et al 1997). Finally the major consequence of the cardiovascular disadaptation is the reduction of the proportion of the stroke volume directed towards the brain compared to the flow directed towards the legs. The measurement of the cerebral to femoral flow ratio by Doppler (Arbeille et al 97) enables to quantify the flow redistribution during orthostatic stress. Due to the lack of pressure gradient between different parts of the body, (at rest and when changing the position in 0 g) the baroreflex is no more stimulated as in one g, and is supposed to change its sensitivity for controling the peripheral flow distribution. Various grades of orthostatic intolerances have been identified (abnormal heart rate increase, drop in blood pressure higher than 2 mmHg, presyncopal symptoms or syncope), but it is still difficult to predict the occurence of such events and to quantify the degree of orthostatic intolerance.

Several countermeasures have been proposed to (a) make the adaptation to zero g more confortable and maintain the volemia (Russian thigh cuffs), (b) to improve the cardiac and peripheral response to orthostatic stresses (repeated LBNP inflight), (c) to reduce the hypovolemia before reentry (saline load) and the muscle atrophy (exercise) which contribute to orthostatic intolerance but the specific effect of each of these countermeasures on the cardiovascular have not been yet completly clarified.

Lower limb muscle and vein interactions. The leg vein compliance increases after some days in HDT which may be related to the absence of stimulation of these vessels (no fluid shift as the subject do not change his position), the reduction of the muscle pressure (muscle atrophy) or the modification of the vein wall mechanical properties. HDT and inflight studies of the calf veins showed opposite results: increase distensibility in HDT, reduced distensibility in flight. This may be due to the fact that HDT position do not well reproduce the effects of microgravity at the lower limb venous level as well as at the arterial level. Moreover the main proximal veins (femoral, popliteal veins) and the small intramuscular distal veins do not respond identically to fluid shift as their environment (muscle, tissue) is different. The muscle mass is known to reduce in absence of physical activity. The lower limb muscles acts as an active compressing system on the vein which favour the venous return. Thus the muscle atrophy may reduce this action which is very important during orthostatic tests. We propose to quantify the muscle volume reduction by using an ultrasound scanner which provide 3D reconstruction of the main lower limb muscles. In addition to the muscle mass reduction the muscle structure may change as there is transfer of liquid from the interstitial to the vascular compartment in HDT. One can suspect that the hydration of the muscle changes during the HDT and thus the stiffness of the muscle. Moreover one can suspect that the quick filtration of liquid into or from the tissue during orthostatic tests may be different pre and post HDT. At last we have to consider that may be the distensibility of the small veins inside the muscle may change in relation with the pressure of the environment or with their wall property change. We propose to monitor the change on muscle and veins cross section area at a pre-selected level during the orthostatic tests pre and post HDT and also to evaluate the water tissue content changes by processing the ultrasound image content in the same cross section

*Physiological factors accounting for venous deconditioning in microgravity.*

It is interesting to consider what factors may account for the changes in lower limb venous compliance following exposure to microgravity. In fact, the etiological factors of lower limb venous alterations are important to consider because they may be concerned by the countermeasures implemented to prevent orthostatic intolerance. First, we have to specify what veins are particularly concerned in the leg volume variations caused by orthostatic stress or any similar stress. Vein contribution to the increase in calf volume during venous occlusion perhaps comparable to an orthostatic stress was shown by Buckey *et al*. (1988) by using Nuclear Magnetic Resistance (NMR) and plethysmography. They showed that at 40 mm Hg of venous occlusion at the root of the lower limb, the increase in volume of deep veins contributed by 90 % to the increase in calf volume. These deep veins have few smooth muscles in their walls and practically no sympathetic nerves. They are not very sensitive to carotid and aortic baroreflexes nor to cardio-pulmonary and thus to sympathoadrenergic activity. Their capacitance is mainly controlled by the degree of extra-mural counter-pressure exerted by nearby skeletal muscles (Pollack & Wood, 1949)**.** These data imply that the characteristics of skeletal muscles which control the distensibility of these deep veins are a major factor of leg volume distensibility. The increase in lower limb venous distensibility has been shown during long as well as short duration or ground-based simulations (Convertino *et al* 1989; Louisy *et al* , 1990, 1995, 1997; Thornton & Hoeffler 1977). During long duration simulations (28, 30 and 42 days), the increase in venous distensibility was maximum only after 2 weeks. Louisy *et al*. (1995, 1997) and Convertino *et al*. (1989) evidenced that atrophy and loss of lower limb skeletal muscles partly explain the increase in venous distensibility observed during space flight simulations. We can therefore speculate that muscle atrophy plays an important part in the mechanisms of orthostatic intolerance during long-duration simulations and space flights. Other factors have been suggested to account for the venous over-distensibility and hence the orthostatic intolerance following exposure to real or simulated microgravity. In fact, it has been shown, in some instances, that lower limb venous distensibility could significantly increase as early as in the first 24 hours or the very first days of weightlessness simulation (Louisy *et al*., 1997). This rapid change in venous distensibility which is paralleled by rapid changes in leg volume cannot be attributed to muscle atrophy. Therefore, other mechanisms, with rapid time constant, may be hypothesized to explain early venous distensibility alterations (Louisy *et al*., 1997) , for example fluid shifts with subsequent changes in intravascular and interstitial volumes and pressures, altering venous transmural pressure and decreasing skeletal muscle fluid volume (Berg *et al*., 1993), alteration of venous smooth muscle tone or baseline tone of skeletal muscles surrounding the veins, alteration of veno-somatic or veno-arteriolar reflexes. The hypothesis on the influence of this last mechanism on orthostatic intolerance changes may be supported by studies which demonstrated in healthy subjects during epidural blockade that the local veno-arteriolar reflex, together with myogenic mechanisms, contribute to the maintenance of arterial blood pressure in the upright position (Henricksen, 1986).

Therefore, the mechanisms accounting for the alteration of lower limb venous distensibility and hence the OI after exposure to real or simulated weightlessness are dependent upon the duration of exposure. During long duration missions or bed-rests, skeletal muscle atrophy is the major determinant whereas during shorter missions or bed-rests, factors with rapid implementation (alteration of vascular and extravascular hemodynamics, neurogenic mechanisms) may be mainly involved. These points are important to consider as they bear an influence on the nature of countermeasures to implement as a function of the duration of weightlessness exposure.

*Characteristics of vascular deconditioning under weightlessness.*

We have demonstrated in studies performed in our laboratory that venous compliance or distensibility is not the only factor to be studied to characterize changes in limb vascular hemodynamics. During a long term (42 days) head-down bed rest (Louisy et al 1997) as well as during space flights (Louisy et al 1998), we evidenced an alteration of parameters of venous filling (venous filling index, arterial flow index), of venous emptying (half-emptying time), of calf muscle pump efficacy (ejection fraction, residual volume fraction), and of capillary function (capillary filtration coefficient). This denotes the complexity of microgravity vascular deconditioning which involves the overall vascular system of the limb, including extravascular factors (e.g. muscular factors). It is hypothesized that factors other than simple venous compliance or distensibility may be correlated with the occurrence of OI after space flights or ground simulation studies.

On the other hand It was demonstrated that after HDT and Space flight longer than 4 days orthostatic intolerance developed. We found in various HDT (4, 5, 7, 28, and 42 days) and space flights (7, 14 ,21, 30days, and 6 months) that orthostatic intolerance as associated with abnormal lower limb arterial vasoconstriction and reduced cerebral to femoral flow ratio and also alteration of the sympathetic/parasympathetic balance, and major vein (femoral) higher distensibility (Arbeille et al, 1994, 1998, 1999; Herault et al, 2000). We propose to evaluate the respective role of the following elements supposed to be involved into the flow redistribution towards the brain during orthostatic tests: volemia (left ventricle volume), sympathetic/parasympathetic balance (BP & HR), splanchnic flow (portal vein), lower limb arterial vasoconstriction (femoral A), leg muscle atrophy and structural changes (calf), and the calf vein distensibility. The leg vein and muscle measurements will be interpreted together with the plethysmographic data.

In conclusion to this background review relating studies performed in our laboratory and studies of other authors, we can state that it is pertinent to assess changes in limb vascular hemodynamics, particularly venous hemodynamics, in studies whose aim is to collect standard baseline data before, during and after long term bed rest and to validate physical and/or pharmacological countermeasures. The efficiency of countermeasures in attenuating cardiovascular deconditioning and OI during and after bed rest may be accounted for by positive effects on some aspects of limb, and particular lower limb vascular and/or venous hemodynamics. Studies on upper limbs could complement this approach. They could confirm that changes observed in lower limbs during exposure to actual or simulated weightlessness are actually due to fluid shifts and hypokinesia instead of to a more general vascular adaptation affecting overall body circulation. In addition, it is important to correlate changes in limb vascular hemodynamics to changes in other physiological parameters suspected to play a complementary role in cardiovascular deconditioning and in the occurrence of OI after bed rest or space flight.

*References*

**Arbeille Ph, D.Sigaudo, A.Pavy-le-Traon, S.Herault, M.Porcher, C.Gharib** Femoral to cerebral arterial flow redistribution and femoral vein distension during orthostatic tests after 4 days in HDT or confinement. Eur J Applied Physiol 78: 210-218; 1998.

**Arbeille Ph, V.Eder, S. Herault, G.Roumy**. Cardiovascular echographic and Doppler parameters for the assessment of orthostatic intolerance. Eur J Ultrasound, 7: 53-71. 1998.

**Arbeille Ph, S Diridillou, S Herault, G.Fomina, J.Roumy, I.Alferova.** Effect of the Thigh cuffs on the Carotid artery diameter, Jugular vein section, and Facial skin edema: a HDT study. J Gravitational Physiology; 6: 39-40. 1999.

**Arbeille Ph, S.Herault, G.Fomina, J Roumy, I.Alferova; C Gharib**. Influence of thigh cuffs on the cardiovascular system during a 7 day bed rest. J Applied Physiol ; 78 : 210-218 ;1999.

**Arbeille Ph, V.Eder, Casset.D, L.Quillet, C.Hudelo, S.Herault**. Realtime three dimensional ultrasound acquisition and display for cardiac volume and ejection fraction evaluation. Ultrasound Med Biol. 26: 201-208. 2000.

**Berg HE, Tedner B and Tesch PA.** Changes in lower limb muscle cross sectional area and tissue fluid volume after transition from standing to supine. Acta Physiol Scand 1993, 148, 379 – 385.

**Buckey JC, Peshock R and Blomqvist CG.** Deep venous contribution to hydrostatic blood volume change in human leg. Am J cardiol 1988, 62, 449 – 453.

**Convertino VA, Doerr DF and Stein SL.** Changes in size and compliance of the calf after 30 days of simulated microgravity. J Appl Physiol 1989, 66 (3) : 1509 – 1512.

**Henricksen O.** local sympathetic veno-arteriolar axon reflex. in : the sympathoadrenal system. alfred bozon symposium 23 (eds christiensen nj, henricksen o and lassen na), pp 67 – 76, munksgaard, copenhagen.

**Herault S, G.Fomina, I.Alferova, J.Roumy, A.Kotovskaya, V.Poliakov, Ph Arbeille**. Cardiac arterial and venous adaptation to 0g during 6 month MIR Space flights with and without "Thigh cuffs". Eur J Applied Physiol, 81 :384-390 ; 2000.

**Louisy F, Gaudin C, Oppert JM, Güell A and Guezennec CY.** Hemodynamics of leg veins during a 30-day -6° head-down bed rest with and without lower body negative pressure. Eur J Appl Physiol 1990, 6, 349 – 355.

**Louisy F, Berry P, Marini JF, Güell A and Guezennec CY.** Characteristics of the venous hemodynamics of the leg under simulated weightlessness. Physical exercise as countermeasure. Aviat Space Environ Med 1995, 66 (6), 542 – 549.

**Louisy F, Schroiff P and Güell A.** Changes in leg vein filling and emptying characteristics and leg volumes during long term head down bed rest. J Appl Physiol 1997, 82, 1726 – 1733.

**Louisy F, André-Deshays C, Cauquil D, Lazerges M, Lafaye A, Camus AL, Schroiff P, Fomina G.** Use of air plethysmography during the French - Russian mission EO 22 on board the Mir Space Station. In : Frontiers in computer-aided visualization of vascular functions. Proceedings of the Seventh International Symposium CNVD '97January 10 - 12, Paris, France. Blazek V and Schulz-Ehrenburg U (Eds.), Düsseldorf 1998.

**Pavy Le Traon A, Louisy F, Vasseur-Clausen P, Güell A and Gharib C.** Contributory factors to orthostatic intolerance after simulated weightlessness. Clinical Physiology 1999, 19, 5, 360 – 368.

**Pollack AA and Wood EH**. Venous pressure in the saphenous vein at the ankle in man during exercise and changes in posture. J.Appl Physiol 1949*,* 1, 649 - 662.

**Thornton WE and GW Hoeffler**. Hemodynamic studies of the leg under weightlessness. In : RS Johnson and LF Dietlein, Eds. Biomedical results from Skylab, NASA SP - 377, US Government printing Office, Washington DC, 1977.

#### Protocol justification according to current scientific knowledge

Exhaustive assessment of vascular hemodynamics including peripheral as well as central hemodynamics have never been performed during a 3-month long term bed rest. This is supposed to bring a lot of informations upon the way humans adapt their cardiovascular system to prolonged exposure to microgravity.

#### The interest of the duration of the head-down bed rest has also to be justified

- to simulate duration of future space missions on board the International Space Station
- to have a better understanding of long term medico-physiological adaptation process to microgravity, which are hypothetically different from those involved in short weightlessness adaptation. The consequence is that medico-physiological countermeasures will be different in nature after long term space missions.

#### Rationale for drug (if used) and dosage selection

Aimless

### Objectives and hypothesis

#### Main objective of the protocol

- to know what cardiac or vascular factors mainly contribute to post-flight OI, because this knowledge will allow the definition of accurate, specific and adapted in-flight and post-flight countermeasure techniques to apply to astronauts, in order to protect their health and enhance their safety.
- to propose research projects which will meet the mains objectives which are as follows: collecting standard baseline data before, during and after long term bed rest validation of physical and/or pharmacological countermeasures.

#### Identification of main parameters

- Parameters of vascular peripheral hemodynamics : limb arterial and venous flows and velocities, peripheral vascular diameters and resistances, venous compliance, venous inflows and outflows, efficacy of the calf muscle pump, muscle volumes, capillary filtration coefficient.
- Parameters of central hemodynamics : cardiac hemodynamics, kidney hemodynamics, splanchnic hemodynamics, hemodynamics at the level of jugular veins.

#### Tested hypothesis

Exposure to long term bed rest will be characterized by peripheral and central vascular deconditioning of greater intensities than those noted during short term exposure.

#### Expected results

This will be dependent upon hydro-electrolytic and volemic changes (hypovolemia), as well as anatomo-histologic changes (for example changes in thickness of heart and vessels, or changes in heart size and vascular section area, decrease in volume and performance of calf muscle pump, new redistribution of local circulations, particularly splanchnic and renal ones, etc.).

#### Secondary objectives ( if necessary)

Aimless

### Specific inclusion and non-inclusion criteria for test subjects

*Inclusion criteria :*

- Age : 25 – 45 years
- Recognized as fitted subjects after selection tests

*Exclusion criteria :*

- History of cardiac or vascular pathology, particularly :
- Venous insufficiency
- Varicose veins
- Thrombo-phlebitis
- Post-phlebitic disease
- Atheroma
- High blood pressure
- Smokers
- Consumers of alcohol, xanthines, narcotics etc.

### Precise description of the experimental protocol

#### Tests description (methods, duration, time of day, protocol conditions, etc.)

Central and peripheral vascular hemodynamics will be assessed by the following non invasive techniques :

- Ultra-sound techniques : Echography and Doppler
- Plethysmographic techniques : air plethysmography and opto-electronic plethysmography (volometry)

Measurements will be performed in supine position (before, during and after bed rest), and during orthostatic stress (stand test) (before and after bed rest).

##### Plethysmographic techniques

*Preliminary measurements.*

Absolute leg volumes (V_0_) will be measured prior to each plethysmographic examination at supine rest and during postural plethysmography by using volometry.

Session duration: 5 min

*Venous occlusion plethysmography at supine rest*

In the venous occlusion protocol (referred to as occlusion plethysmography), a wide occlusion cuff is placed at the root of the measured limb, just above the knee or the elbow. The limb is raised above the bed, slightly flexed, and externally rotated. This allows muscular relaxation and adequate limb venous emptying before starting the measuring procedure. After 20 minutes in the resting position a 50 mmHg venous counterpressure is applied at the root of the limb. This counterpressure, largely below the arterial diastolic pressure, allows limb venous filling from the arterial and microcirculatory side of the vascular network until the leg venous pressure equals then exceeds the pressure in the occlusion cuff. At this moment, the limb volume change curve becomes a plateau. After the plateau, cuff counterpressure is suddenly released, venous drainage is restored and the limb gradually recovers its initial volume, first rapidly then slowly. An example of plethysmographic curve is shown in figure 1.

In this protocol, changes in forearm hemodynamics will be assessed by volometry and air plethysmography with venous occlusion (30 and 50 mmHg counterpressure). The same parameters (but calf muscle pump eficacy and residual volume) as for the legs will be measured and calculated.

Session duration: 45 min

*Measurement during stand test (postural plethysmography)*

In the stand test procedure, which will be referred to as postural plethysmography, the subject first lays supine on the bed, with his legs elevated and relaxed. Plethysmographic recording is initiated after 20 min of rest in order to obtain a control graph. The subject is then asked to stand, with his weight on both legs. In this position, an increase in leg volume is recorded as a result of venous filling (usual anterograde filling from capillary bed plus pathological retrograde filling if venous reflux exists). The subject is asked to stand still until a plateau is reached indicating that the veins are full (figure 2).

Session duration: 15 min for preparation per subject

Continuous measurement during stand test

**INSERER FIG 1**

**IBSERER FIG 2**

##### Ultra-sound techniques, Measurement of central and peripheral hemodynamics

*Ultrasound parameters*

*a) Cardiac parameters :* The cardiac function will be assessed by echocardiography B and TM modes. Parasternal long-axis echocardiograms will be recorded with a commercially available echocardiograph using two working modes : a 3-MHz real-time imaging mode (Sector scan) and a time-motion (TM) mode. Such a system had previously been used for cardiovascular investigation during space flight and simulated weightlessness. It allows visualization of the cardiac chambers and displays the ventricular and auricular wall movements during the cardiac cycle. The subjects will be lying on their left side to increase the intercostal space window through which the cardiac chambers will be investigated. Echocardiographic images and TM traces will be recorded during held expiration. Under such conditions, the echocardiographic data are of good quality in most of the subjects. The end-diastolic diameter will be selected on the TM trace as the largest ventricular diameter and the end-systolic diameter as the smallest one. Ventricular diameters and HR will be both measured on the same TM trace. Ten cardiac cycles will be analysed and averaged for each data point. The left ventricular volume will be estimated from the previously measured diameters by using the Teichholz formula (Teichholz et al 1974). The following parameters will be measured or calculated : left ventricular end-diastolic volume LVDV, left ventricular end-systolic volume LVSV, stroke volume SV (calculated as LVDV-LVSV), heart rate HR, cardiac output CO (calculated as the product of HR and SV), ejection fraction EF (calculated as [LVDV-LVSV]/LVDV). These cardiac investigation at rest will be performed by a sonographer using a 3 Mhz sector scan probe.

*b) Arterial vascular resistances****.***Total peripheral resistances (TPR) will be calculated from the mean pressure (MBP) and cardiac output (CO). The vascular resistances in particular areas (brain, lung, lower limbs...) will be evaluated from the Doppler spectrum of the artery supplying the area of interest. At rest the vascular resistance will be measured from the Doppler spectrum obtained with a duplex (Echo-Doppler) probe. During dynamic test the Doppler spectrum will be recorded from flat skin probes maintained in a fixed position in front of the artery of interest by a harness (cerebral, femoral) (Aaslid et al 1982; Arbeille et al 1997).

*-Low resistance circulation (middle cerebral, renal, mesenteric arteries):* For the assessment of the cerebral vascular resistances, we shall use the resistance index Rca= (Sca-Dca)/Sca where Sca and Dca are the maximum systolic and end-diastolic Doppler frequencies. As the resistance increases, the end-diastolic component decreases and the Rca increases. Although Rca does not provide an absolute value of the vascular resistance, it changes in proportion to the vascular resistance (Aaslid et al 1982; Adamson et al 1990; Maulik et al 1989).

*-High resistance circulation (femoral artery):* The Doppler frequency spectrum of normal lower limb arteries shows a positive systolic frequency peak (Sfa), followed by a negative frequency peak at the beginning of diastole (Dfa) generated by the rebound of the systolic pressure wave from the distal arterio-capillary junction, which presents a high resistance to flow. The vascular resistance in the lower limb will be evaluated by using the femoral high resistance index expressed as Rfa=Dfa/Sfa, with Sfa the systolic maximum forward flow frequency and Dfa the diastolic maximum reverse flow frequency on the femoral artery Doppler spectrum. This index has been validated in an animal ewe model (Arbeille et al 1995) and on a human model (Arbeille et al 1998) by comparing the absolute values of lower limb Rfa with those of the classic vascular resistances calculated from the mean pressure and flow at the Doppler recording point (mmHg.ml^-1.^min^-1^).

*c) Vein cross section area (calf, portal, jugular, femoral):* The jugular vein section (Aj.v) will be measured on B mode echographic transversal views of the neck at the level of the Adam's apple. The cross-sectional area of the portal vein (Apt.v) will be also measured on longitudinal and transversal echographic views, of the main trunk inside the liver. The cross-sectional area of the superficial femoral vein (Afv) will be measured on a transversal echographic view of the vein, 1 cm below the femoral arterial bifurcation. The cross-sectional area of the sural veins (Asu.v) will be also measured on a transversal echographic view, 1 cm down from the anterior tibial artery.

At rest the vein will be investigated using an echographic probe handed by a sonographer. During stand test the echographic probe will be fixed on the skin in front of the area of interest only the portal vein will be explored with the probe handed by the operator.

*d) Muscle volume and structure:* The structure of the muscle will be evaluate on the same transverse echographic view as for the sural veins measurements. The area (cm²) and thickness (cm) of the muscle will be measured as well as the area of the veins (cm²) inside the muscle image. The muscle volume will be measured by using an « ultrasound scanner » designed by our lab. It consist of a linear support on which the ultrasound probe moves and acquires a serie of parallel transverse views of the muscle. An electrical low voltage micro engine allows to adjust the probe speed and the sequence for image acquisition. The muscle volume will be reconstructed from the 20 successive views recorded, using a 3D program (Autocad 12). The duration of the acquisition is approximately 5 min. The scanner was tested in vitro in our lab and the error on the volume measurements was lower than 3%.

At rest the sural veins and muscle cross section areas and the muscle volume will be measured from the data collected with the ultrasound scanner pre and in HDT. During stand and LBNP tests the sural veins and muscle cross section areas changes will be assessed using a flat Echographic probe sticked on the calf skin during the test.

The arterial, venous, and muscular morphological investigations will be performed using a conventional 7.5-MHz ultrasound (B-mode) probe. The diameter values will be expressed in mm, the vein areas will be expressed in mm² and the volume muscle in cm3.

***Session of measurement AT REST:***

1. Left ventricle long axis view (4 min)
2. Portal vein flow (6 min)
3. Renal vein & artery (4 min)
4. Cerebral artery flow (3 min)
5. Femoral artery flow (3 min)
6. Jugular vein section (3 min)
7. Sural vein section (3 min)
8. Femoral vein section (6 min)
9. Calf muscle volume (4 min)
10. Instrumentation – preparation (7 min) **total 40 - 45 min per subject**

***Session of measurement during STAND TEST***

1. Instrumentation for Sural vein and muscle section (3 min)
2. Instrumentation for Cerebral artery flow (3 min)
3. Instrumentation for Femoral artery flow (3 min)
4. Instrumentation for Portal vein flow (3min): 1 meas pre-Test and every 3 min in test
5. Jugular vein section (1 min): 1 time pre-stand and at the last min of the test
6. Renal vein & artery (2 min): 1 time pre-stand and at the last min of the test

**total 14 min for preparation per subject, continuous measurement during the test.**

*Hemodynamic and morphological parameters*

**At REST:**

* Systemic parameters: Blood pressure and ECG (Portapress).

* Cardiac function: Left ventricule, stroke volume, cardiac output, ejection fraction, myocardium thickness.

* Peripheral arteries: Cerebral artery: Blood flow and vascular resistance. - Renal artery: Vascular resistance - Fémoral artery: Blood flow and vascular resistance

* Veins: Jugular vein. Cross section area, wall kinetic - Portal vein: Diameter, flow- Femoral vein. Cross section area, Renal vein diameter- Sural veins. cross section area

* Muscle volume - Thigh and Calf muscle. cross section area, volume in cm3

**During STAND TEST**

* peripheral arteries: -Cerebral resistance and flow (vasodilation) - Fémoral artery: Blood flow and vascular resistance.

*Venous return: - Portal vein: Diameter & flow - Sural veins. cross section area

*Calf muscle. cross section area structural change.

#### Protocole work schedule

*At supine rest:*

- Pre-HDT: BDC -5, BDC -2 (before stand test procedure)
- During HDT: HDT 21, HDT55, HDT 83
- Post-HDT: R+1 (before stand test procedure)

R+7 (before stand test procedure)

*During stand test:*

- Pre-HDT : BDC -2
- Post-HDT: R+1, R+7

#### Measured parameters and conditions of measurements

See above.

Complementary informations : Specific conditions requested for the experiment: temperature of the room in which measurements will be performed must be between 22°C and 27°C.

#### Equipment used

##### Ultra-sound techniques (Hardware description (trademark, model, …), specify if experimental equipment is commercially available or self built.

Measurements at rest:

- Echocardiograph with pulsed Doppler (Unite Med Physiol Spatiales -Tours)
- Echograph for superficial organs (muscle, veins - UMPS Tours)
- Motorized muscle scanner (UMPS Tours)
- Computer for image processing (UMPS Tours)

Measures during tests (LBNP, Stand tests, head flexions)

- Echograph for superficial organs (muscle, veins)
- Echograph for deep organs (portal vein)
- Multichannel Doppler unit for arterial monitoring (DMS)
- Computer and spectrum analyser (UMPS-Tours)

All the equipment mentioned above consists in commercial Echograph and Doppler devices. (Esaote, Aloka and Dynamic imaging, and DMS) They were already used for HDT 7days 1998 and HDT woman 1999.

##### Plethysmographic techniques.

*Opto-electronic plethysmography or volometry : measurement of absolute leg and forearm volumes.*

The volometer (figure 3) (Bösl, Medizintechnik, Aachen, Germany) is an optoelectronic measuring device for determining the volume of an extremity from the measurements of its diameters (horizontal and vertical diameters). It comprises clamps for limb fixation, both proximal and distal, with respect to the measured limb segment, a sliding frame equipped with light transmitters and receivers, and a microprocessor. After applying pressure to the measuring head, the frame is moved from a pre-established initial position to the area to be measured, in approximately two seconds. During this time, one vertical and one horizontal diameters are measured at 225 successive points and stored in a microcomputer. A sensor incorporated in the frame determines the distance traveled and subsequently the location at which each measurement was made. The computer terminates the measuring operation after a distance of 36 or 40 cm or any other selected travel distance. The principle of limb volume measurement by optoelectronic plethysmography is illustrated in figure 4 showing a diagram of the frame equipped with the optoelectronic sensors with the cross section of a limb in its center. To the right of the limb and below the limb the frame includes 120 phototransistors

**Figure 3**. A photo of the optoelectronic plethysmograph or volometer to measure limb volumes.


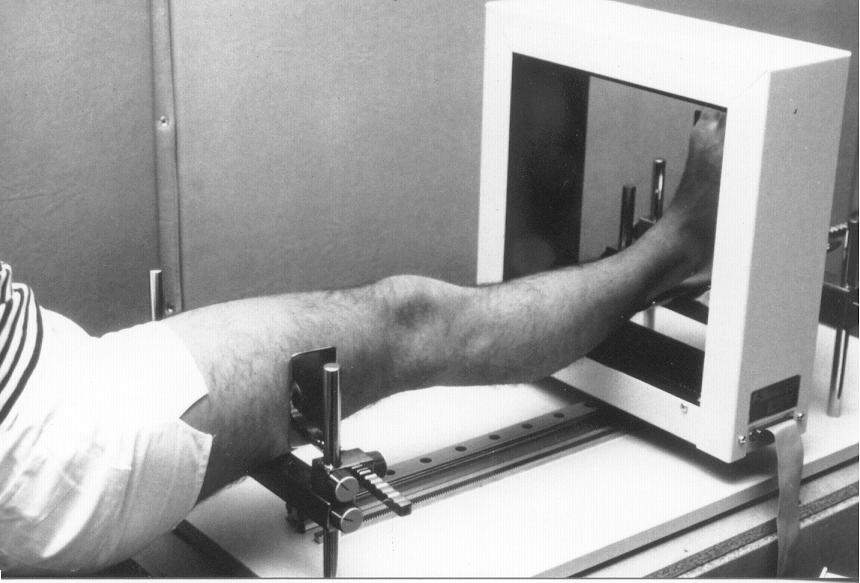


**Figure 4.** Flow diagram of the optoelectronic sensor plethysmograph (volometer).

**a**: measuring frame and connected equipment; **b**: diameters measured by volometer on a limb section.

placed in staggered rows ("receivers") spaced every 2.5 mm along the line. Opposite each one of these rows of receivers is a corresponding row of infra-red emitting semi-conductor diodes ("transmitters"). All light transmitters and receivers can be individually controlled by the microprocessor in accordance with its program to obtain 120 vertical and 120 horizontal light barriers. Limb diameters are measured by automatically switching on vertical and horizontal barriers successively in their respective rows. The number of light barriers interrupted by the limb is the measure of the limb diameter. For each point of the limb the computer measures two perpendicular diameters, a horizontal and a vertical diameter, stores them, and records their distance from the reference (initial) point on the limb within approximately 0.01 second. Knowing the successive cross-section areas over a given distance, calculated from the two measured diameters, the computer determines the entire volume of the measured stretch.

*Air plethysmography.*

The APG-1000 (ACI medical Inc., Sun Valley, CA) is a calibratable pneumatic volume plethysmograph. The principle of the device is to measure pressure changes in a cuff bulging with air at a constant pressure of 6 mmHg (referred to as bias pressure). The cuff consists of a 14-inch tubular polyvinyl chloride (PVC) air chamber (capacity 5 liters) that surrounds the leg to be measured. The pressure of 6 mmHg is the lowest that ensures good contact between the air chamber and the limb and that does not interfere with superficial circulation. A calibration test performed before measurements allows the conversion of pressure changes in the cuff into volume changes in the limb segment. The cuff is connected to a pressure transducer. The signal is transmitted to an amplifier, and then processed as an analog display on a PC.

#### Description of the test termination criteria of the protocol

Test termination criteria of the stand test (cf description in the overall protocol)

#### 6.11.4.6 Specific medical monitoring (ECG, blood pressure monitoring, etc.) (if necessary)

ECG and blood pressure monitoring during stand test

#### 6.11.4.7 Other specific requirements (dietary, conditions of the experiment sessions, etc.)

Aimless

#### 6.11.4.8 Biological samples handling, preservation conditions, transport

Aimless

#### 6.11.4.9 Data utilisation and analysis, specify

##### 6.11.4.9.1 Parameters analysed.

See above

##### 6.11.4.9.2 Statistical tests

Measurements performed in the supine position, before, during and after bed rest: comparative measurements of mean values assessed before, during and after bed rest by ANOVA: pair comparisons by post-hoc Fisher's t test (with measurements performed before bed rest as controls)

Measurements performed during stand tests : paired comparisons (t-tests) between :

- Parameters measured in the supine position before and after bed rest
- % responses in the standing position (expressed in % as compared to supine) before and after bed rest

Correlations tested between variables measured at the end of bed rest (venous compliance, venous capacity, venous surface area, muscle volumes, etc.) and tolerance time to post-bed rest orthostatic stress.

##### 6.11.4.9.3 Software used

Statview (Abacus concepts).

### 6.11.5 Hazards, side effects, possible inconveniences, discomfort and constraints to subjects

Methods used in this proposal are non-invasive and do not give rise to safety hazards. The only hazards that possibly may occur are syncope and loss of consciousness during stand test. These are rapidly resolved by laying the subject down horizontally.

### 6.11.6 Protocol summary (one page), written on a separate sheet

**Objectives of the study**

The aim of the present project is to carry on previous studies made in our laboratory which tend to demonstrate that vascular deconditioning (arterial, venous and microcirculatory) deconditioning, is a key contributory factor to post-flight or post-bed rest OI. The aim of this AO is also to propose research projects which will meet the main objectives which are as follows :

- collecting standard baseline data before, during and after long term bed rest
- validation of physical and/or pharmacological countermeasures.

Exhaustive assessment of vascular hemodynamics including peripheral as well as central hemodynamics have never been performed during a 3-month long term bed rest. This is supposed to bring a lot of informations upon the way humans adapt their cardiovascular system to prolonged exposure to microgravity.

**Protocol description**

Central and peripheral vascular hemodynamics will be assessed by the following non invasive techniques: 1/ Ultra-sound techniques : echography and Doppler; 2/ Plethysmographic techniques : air plethysmography and opto-electronic plethysmography (volometry). Measurements will be performed at rest (supine position) (before, during and after bed rest), and during orthostatic stress (stand test) (before and after bed rest).

Plethysmographic techniques (air plethysmography) (duration: 45 min) at rest consist in measuring leg and forearm volume changes under the effect of venous occlusion (sub-diastolic pressure) applied at the root of the limb. Opto-electronic plethysmography (volometry) (duration: 5 min) allows measurement of absolute leg and forearm volumes. During the stand test procedure, air plethysmographic techniques will be used without venous occlusion (gravity is the stimulus which provokes leg volume changes). Ultra-sound techniques (duration: 45 min) use the capabilities for ultra-sound waves to detect red blood cell movements (Doppler procedures), or to visualize body structures with different echogenicity (echotomography). Therefore, they allow measurement of cardiac chamber dimensions, arterial and venous velocities, vascular cross section areas, arterial peripheral resistances, and even muscle volume and structure.

The following parameters will be measured at supine rest :

1/ peripheral parameters in the limbs: arterial flow, venous filling parameters, venous compliance, venous capacity, venous emptying parameters, efficacy of calf muscle pump, capillary filtration, femoral vascular flow and resistances, vein cross section areas (femoral, popliteal), muscle volume and structure

2/ middle cerebral artery flow and resistance

3/ central parameters: cardiac function, flow and resistance of renal and mesenteric arteries, portal and jugular vein cross section.

Similar parameters will be measured during a 10 min stand test procedure.

**Protocole work schedule**

*At supine rest:*

- Pre-HDT: BDC -5, BDC -2 (before stand test procedure)
- During HDT: HDT 21, HDT55, HDT 83
- Post-HDT:	R+1 (before stand test procedure)

R+7 (before stand test procedure)

*During stand test:*

- Pre-HDT : BDC -2
- Post-HDT: R+1, R+7

**Main medical risks**

Methods used in this proposal are non-invasive and do not give rise to safety hazards. The only hazards that possibly may occur are syncope and loss of consciousness during stand test. These are rapidly resolved by laying the subject down horizontally.

### 6.11.7 Investigators

**Principal Investigators:** Dr Louisy F.

**Co-Investigators**:

Pr. Arbeille P

Dr. Roumy J.
