# Supplementary material for: Effect of Physical Inactivity on the Oxidation of Saturated and Monounsaturated Dietary Fatty Acids: Results of a Randomized Trial
Source: PLoS Clin Trials. 2006 Sep 29;1(5):e27. doi: 10.1371/journal.pctr.0010027 (PMC1584255; doi:10.1371/journal.pctr.0010027)
Supplement: Protocol S1 — (67 KB DOC) [file pctr.0010027.sd004.doc]

**Detailed methods**

***Primary outcome measures***

Dietary fat oxidation was measured before (control period day 11) and at the end of the bed rest (bed rest day 78), 36 hours after the last bout of exercise, through the combination of indirect calorimetry and a recently developed method of double labeling of fatty acids by stable isotopes, mixed in a standard breakfast. Oleic (18:1) and palmitic (16:0) acids were selected for the study. Those fatty acids represent the main monounsaturated and saturated fatty acids of the human diet (38 and 20% of total intake, respectively).

Two tests were conducted in identical conditions during the control period and at the end of the bed rest. The volunteers woke up at 0600h. Baseline breath and urine samples and fasting blood sample were collected. The participants ingested 0.4 g/kg of H_2_^18^O (10% enriched, CIL Andover MA, USA) to measure total body water. RMR was measured for 1 hour using indirect calorimetry (Deltatrac II, GE, USA) under the supervision of the investigators. The first 5-min of data were excluded as were 2-min of data following any movement or loss of wakefulness. Then, a breakfast representing 50% of the RMR (expressed as MJ/24h) in energy (55% carbohydrates, 15% protein and 30% fat) was offered to the participants. Part of this breakfast was composed of a liquid replacement meal (Boost High protein, Mead Johnson USA) in which 15mg/kg of [d_31_]palmitic acid (>98% enriched, CIL Andover MA USA) and 10mg/kg of [1-^13^C]oleic acid (>99% enriched, CIL Andover MA USA) were homogenized at least at 65°C (melting point of palmitate). One participant of each group had the same breakfast without tracers to estimate the natural background isotopic variations induced by the meal. Then, hourly breath and fresh urine samples were collected for 7 hours. Additional fresh urine samples were collected 12, 24 and 36 hours post-dose. Equilibration time for H_2_^18^O was taken at 3 and 4-hr post-dose. During the 7 hours of the test, total substrate use, energy expenditure, diet-induced thermogenesis (DIT) and non-protein respiratory quotient (NPRQ) were hourly measured by continuous indirect calorimetry. At the end of the test, the participants were escorted back to their room and had dinner. To correct oxidation values of oleate for the loss of ^13^C in the TCA cycle, additional tests were performed in all participants 7 days prior to the oral lipid loads, in strict similar conditions but oleate and palmitate tracers were replaced by 2mg/kg of [1-^13^C]acetate (>99% enriched, CIL Andover MA USA).

Breath ^13^CO_2_/^12^CO_2_ ratios were measured in duplicate on an Isoprime isotope ratio mass spectrometer (GV Instruments, Villeurbanne, France) through a continuous flow inlet system using Isoprime Multiflow interface (GV Instruments, Lyon, France). ^2^H/^1^H from urine samples were analyzed in triplicate on the same interface/IRMS system using the equilibration method catalyzed by platinum. ^13^CO_2_ and ^2^H_2_O measures were done at the Centre de Recherche en Nutrition Humaine de Lyon (France). ^18^O enrichments of H_2_^18^O from urine samples were equilibrated with CO_2_ and analyzed in quadruplet on a Delta-S isotope ratio mass spectrometer (Finnigan MAT, San Jose, CA, USA) through a continuous flow inlet system [1].

Recovery of [1-^13^C]oleic acid and [1-^13^C]acetate were calculated as the instantaneous recovery of ^13^C in expired CO_2_ hourly sampled over the 7 hours of the test and expressed as a percentage of the dose, according to the formula:

% dose recovery = [VCO_2_ • ∆δ_13CO2_ • R_STD_ / 1000] / [D • P • n/(MW • 100)] • 100

where δ_13CO2_ represents delta per mil = (R_sample_ / R_standard_ – 1) * 1000 with R being the ratio ^13^C/^12^C, ∆ represents δ_13CO2_ values corrected for each individual’s baseline and for the meal related natural background variation obtained from the two participants not dosed, R_STD_ represents ^13^C/^12^C of the standard CO_2_, P represents ^13^C isotope atom percent ([1-^13^C]acetate, Na salt 99%; [1-^13^C]oleate 98%), n represents the number of labeled atoms per molecule (1 for both tracers), MW is the molecular weight ([1-^13^C]acetate, Na salt = 83; [1-^13^C]oleate = 282), D the dose (in grams), and VCO_2_ the carbon dioxide production in ml/h. Cumulative recovery was calculated using the trapezoid rule. The background changes in ^13^CO_2_ following the meal were less than 0.15 permil, which was small relative to the 15 permil changes observed following ^13^C oleate administration. Oxidation of [1-^13^C]oleate was calculated by dividing the individuals oleate % cumulated recovery of by their own acetate % cumulated recovery factor to account for isotopic exchange in the TCA cycle [2]. Recovery of [d_31_]palmitate was calculated as the cumulated recovery of ^2^H in TBW hourly sampled [3] through urine voids using the formula:

% dose recovery = [TBW • 2 • ∆δ_2H_  • R_STD_ / 1000] / [D • P • n/(MW • 100)] • 100

where δ_2H_ represents delta per mil = (R_sample_ / R_standard_ – 1) * 1000 with R being the ratio ^2^H/^1^H, ∆ represents δ_2H_ values corrected for each individual’s baseline and for the meal related natural background variation, R_STD_ represents ^2^H/^1^H of the standard H_2_, P represents ^2^H isotope atom percent ([d_31_]palmitate, 98%), n represents the number of labeled atoms per molecule (i.e. 31), MW is the molecular weight (i.e. 287), TBW the total body water in moles, and D the dose ingested (in grams). ∆δ_2H_ values were corrected for insensible water loss using the formula:

∆δ_2H_ at time t (corrected) = ∆δ_2H_ at time t (uncorrected) • (1 - exp(-k_D_ • ∆t / 60 / 24)),

where ∆t represents the time between two samples and k_D_ the deuterium daily turnover rate taken as 0.07900 and 0.08200 day^-1^ during the bed rest and control periods, respectively. These values represent the average elimination rates measured in eight participants before and after a 42-day bed rest [4]. TBW (g) was calculated according to α • D / (∆ (^18^Oppm) • 1.007) where D is dose (in grams), ∆ (^18^Oppm) is the H_2_^18^O enrichment of body water in part per million at equilibration, α is the enrichment of the injected solution and determined by the slope of a standard curve in ppm•g/mg and 1.007 a correction factor for isotope exchange [5].

***Secondary outcome measures***

Body weight was measured daily on a special supine weighting device. Total body water was measured by hydrometry based on the isotope dilution of H_2_^18^O in body water. Fat (FM) and FFM were measured by dual-energy X-ray absorptiometry on QDR 4500 W (Hologic Massy France) scanner using the QDR System Software for version 11.2.

Energy expenditure, total fat, carbohydrate and protein oxidation rates as well as the NPRQ, were calculated from indirect calorimetry data using VCO_2_ (L.min^-1^), VO_2_ (L.min^-1^) and urinary nitrogen (N in g.min^-1^) and the following equations [6]:

Glucose oxidation (g.min^-1^) = 4.55VCO_2_ - 3.21VO_2_ – 2.87N

Lipids oxidation (g.min^-1^) = 1.67(VO_2_ - VCO_2_) – 1.92N

Protein oxidation (g.min^-1^) = 6.25N

Energy expenditure (kcal.min^-1^) = 3.91VO_2_ + 1.1VCO_2_ – 3.34N

QRNP = (VCO_2_ – 4.84N) / (VO_2_ – 6.04N)

When NPRQ is higher than 1, the lipid oxidation equation yields negative numbers. Because the apparent rate of fat oxidation is the difference between fat oxidation and synthesis, calculated rates of negative fat oxidation quantitatively represents net rates of fat synthesis. In such situations glucose oxidation increases. This represents the sum of net glucose oxidation plus glucose disappearance that is converted to lipids. During lipogenesis, glucose oxidation is thus overestimated and net glucose oxidation is calculated by [6]:

Glucose oxidation _lipogenesis_ (g.min^-1^) = 1.34VCO_2_ – 6.49VO_2_

Diet-induced thermogenesis was measured for 5 hours as the post-breakfast suprabasal energy expenditure and expressed as a percentage of the intake. Fasting hormones and metabolites were measured the day of the oral lipid load. Classical and widely described methods were used to determine insulin by RIA, glucose, free fatty acid, glycerol, triglycerides and ß-hydroxybutirate by enzymatic methods. Urinary nitrogen was measured by chemoluminescence (Antek 703C, Sopares, Paris, France).

**References**

1. Schoeller DA, Luke AH (1997) Rapid 18O analysis of CO2 samples by continuous-flow isotope ratio mass spectrometry. J Mass Spectrom 32: 1332-1336.

2. Votruba SB, Atkinson RL, Schoeller DA (2003) Prior exercise increases dietary oleate, but not palmitate oxidation. Obes Res 11: 1509-1518.

3. Raman A, Blanc S, Adams A, Schoeller DA (2004) Validation of deuterium-labeled fatty acids for the measurement of dietary fat oxidation during physical activity. J Lipid Res 45: 2339-2344.

4. Blanc S, Normand S, Ritz P, Pachiaudi C, Vico L, et al. (1998) Energy and water metabolism, body composition, and hormonal changes induced by 42 days of enforced inactivity and simulated weightlessness. J Clin Endocrinol Metab 83: 4289-4297.

5. Racette SB, Schoeller DA, Luke AH, Shay K, Hnilicka J, et al. (1994) Relative dilution spaces of 2H- and 18O-labeled water in humans. Am J Physiol 267: E585-590.

6. Simonson DC, DeFronzo RA (1990) Indirect calorimetry: methodological and interpretative problems. Am J Physiol 258: E399-412.
